# Supplementary material for: The SnaFab versus the Razi antivenom for treatment of snakebite envenomation: A randomized, double-blind (investigator and victims), active controlled, non-inferiority clinical trial
Source: PLOS Glob Public Health. 2025 Nov 24;5(11):e0004281. doi: 10.1371/journal.pgph.0004281 (PMC12643280; doi:10.1371/journal.pgph.0004281)
Supplement: S1 Table — (DOCX) [file pgph.0004281.s001.docx]

S1 Table- Supportive treatments and other necessary medical care in clinical trial of SnaFab vs. Razi antivenom in snakebite victims

| Treatment | Diagnosis | Adverse effect |
| --- | --- | --- |
| Antibiotic based on Infectious expert consult | Clinical | Cellulitis |
| Unfractionated Heparin 5000 IU QID | Doppler Sonography | Deep Vein Thrombosis (DVT) |
| Iso-group & Iso-Rh Packed cell(2 U stat) | Hemoglubin <7 | Active bleeding |
| Amp Ondansetron 4 mg IV | Clinical | Naseau and vomiting |
| Amp MgSO4 2g IV infusion | ECG analysis | QTc prolongation |
| Fluid therapy | CPK>1000 U/L | Rhabdomyolysis |
| Dialysis based on nephrology consult | Increase in SCr>0.3 mg/dl in 48 hours or urine output<0.5 ml/kg/h | Acute Kidney Injury |
| Fasciotomy  Based on orthopedic consult | Clinical | Compartment syndrome |
