# Supplementary material for: The SnaFab versus the Razi antivenom for treatment of snakebite envenomation: A randomized, double-blind (investigator and victims), active controlled, non-inferiority clinical trial
Source: PLOS Glob Public Health. 2025 Nov 24;5(11):e0004281. doi: 10.1371/journal.pgph.0004281 (PMC12643280; doi:10.1371/journal.pgph.0004281)
Supplement: S1 Protocol — (DOCX) [file pgph.0004281.s002.docx]

Objectives

The aim of this study is to investigate the efficacy and safety of the antivenom produced by Padra Serum Alborz Company compared to the antivenom produced by Razi Vaccine and Serum Research Institute in snakebite patients in Imam Reza Hospital in Mashhad, Ayatollah Taleghani Hospital in Urmia and Razi Hospital in Ahvaz.

Primary objective

To assess the non-inferiority of efficacy of the snakebite antivenom manufactured by Padra Serum Alborz compared to the antivenom produced by Razi Vaccine and Serum Research Institute, in snakebite patients.

Secondary objective

To assess the safety of the snakebite antivenom manufactured by Padra Serum Alborz compared to the antivenom manufactured by Razi Vaccine and Serum Research Institute in snakebite patients by comparing all adverse events during the study based on occurrence, severity and duration of the adverse effect.

Trial design

This is a randomized (1:1 ratio), multicenter, two-arm, parallel, double-blind (victim and results analysis team), active-controlled trial with a non-inferiority approach.

Methods

Phase 3 (victims aged 2 to 60 years) in which antivenom from Padra Serum or Razi Vaccine and Serum Research Institute is randomly received based on randomization code:

After providing complete declaration to the victims (over 18 years old) or the victim's guardian (2 to 14 years old) or both (14 to 18 years old), when they consent to enter the study (adhering the situation in urgent patient setting); each victim will be evaluated for inclusion and exclusion criteria, and then at admission, routine tests including the following will be taken from all victims:

CBC, diff, PT, PTT, INR, Fibrinogen, Blood Sugar (BS), Blood U rea Nitrogen (BUN), Creatinin (Cr), Na (sodium), K (Potassium), CPK (Creatinine Phospate Kinase), AST (Aspartate transaminase), ALT (alanine aminotranferase), LDH (Lactate dehydrogenase), Bilirubin (total, direct), ECG (electrocardiogram), U/A (Urine Analysis).

Victims with moderate and severe manifestation will be administered five and ten vials of antivenom, respectively. In cobra-bitten victims with no sign and symptoms regardless of bite site, five vials of antivenom will be administered.

If the snake species is not known and the victim has no obvious clinical manifestation (other than the bite site) and cobras are inhabitant in the geographical area (Mashhad), five vials of antivenom will be administered. Subsequent doses will be based on the patient's subsequent clinical status.

If there are no cobras in the geographical area, victims will only receive antivenom when they develop moderate or severe symptoms and signs.

The victim's clinical signs and symptoms and paraclinical tests will be evaluated after the initial dose of antivenom and then 30 minutes later and at 1, 6, 12, 24, 48 and 72 hours after antivenom administration.

If after one and six hours of the initial dose patient develops either 1.2<INR<3 IU ,25<PTT<50 sec, 25000<Plt<150.000/mm3, 200<CPK<1000 IU/L, the patient will be visited by the attending physician every hour and evaluated every six hours for lab data abnormalities. Lab data examiniation will be repeated every 6 hours if they do not return to normal values, otherwise, they will be evaluated daily.

The serum sickness questionnaire will be completed by the medical staff through a phone call one and two weeks after antivenom administration.

| **Serum Sickness Questionnare** | |
| --- | --- |
| Have you experienced abnormal and unintentional itching? | No🞎 Yes🞎 |
| Have experienced red spots (rash) anywhere on your body? | No🞎 Yes🞎 |
| Have you detected high temperature? | Yes, before developing the rashes🞎  Yes, after developing the rashes🞎  Yes, it persists before and/or after the rash development🞎  No🞎 |
| Have you experienced any chills and shaking? | No🞎 Yes 🞎 |
| Have you experienced any headache episodes? | No🞎 Yes 🞎 |
| Have experienced re-swelling after the swelling subsided? | No🞎 Yes 🞎 |
| Have you experienced swelling in your groin and armpit? | No🞎 🞎Yes |
| Have you experienced joint pain? | No🞎 Yes 🞎  If yes, distinguish the pain location:  Foot toe joints🞎  Hand finger joints🞎  Knee and thigh joints🞎  Wrist, elbow and arm joints🞎  Neck joints🞎 |
| Have you experienced muscle pain? | No🞎 🞎Yes |
| Have you experienced redness and flushing on your face? | No🞎 🞎Yes |
| Have you been feeling unpleasant recently? | No🞎  Bored🞎  Weakness and malaise🞎  Dizziness🞎  Nauseous🞎  Flushing🞎  Stomach pain🞎 |

After completing each questionnaire, if fever, malaise and itchy rash (urticaria or maculopapular) occur concurrently one to two weeks after antivenom administration, serum sickness is suspected, in which case the victim will be referred to a medical center and undergo work-up examinations including ESR, CBC and diff, C3, C4, CRP

The above tests are non-specific but leukocytosis, increased ESR and CRP, and decreased C3 and C4 are expected in this reaction.

Appropriate medical measures based on serum sickness protocol will be conducted (Hospitalization of the victim and initiation of systemic steroids and if other symptoms occur, supportive treatment with NSAIDs or antihistamines).

Inclusion criteria

- History of snakebite
- Consent to participate in the study
- Victims aged 18 to 60 years in the safety phase (Phase II) and victims aged 2 to 60 years in the efficacy phase (Phase III)
- Victim who has reached the relevant treatment center within 12 hours after the bite.
- Victim who needs antivenom treatment according to the snakebite severity scale.

Exclusion criteria

- Victims with a history of horse serum sensitivity
- Victims with a previous history of snakebite or scorpion sting who have previously received antivenom (snake or scorpion)
- Two or more bites at the time of admission
- Victims who have received antivenom before reaching the relevant center
- Victims who have manipulated the wound (cutting, sucking, burning, etc.) before reaching the treatment center.
- Victims with life-threatening hemorrhages (such as oral cavity and upper respiratory tract hemorrhages)
- Past drug history of anticoagulants (heparin or warfarin)
- Victims with a history of coagulation disorders, heart disease, neuromuscular disorders, renal failure, liver failure
- Pregnant or lactating women
- Victims bitten by a sea snake.
- Victims requiring mechanical ventilation at the time of admission.

Interventions

The exact specifications of the test product (Padra Serum Alborz snakebite antivenom) and control (Razi Vaccine and Serum Research Institute snakebite antivenom) are as follows:

|  | Test Product | Control Product |
| --- | --- | --- |
| Ingredients | Snake antivenom immunoglobulin, buffer, phenol | Snake antivenom immunoglobulin, buffer, phenol |
| Dosage form | Vial | Vial |
| Volume (mL) | 10 | 10 |
| Storage | Avoid direct sunlight, refrigerated in 2-8°C | Avoid direct sunlight, refrigerated in 2-8°C |

After randomization, victims are placed in one of the following groups:

Group 1: Snakebite antivenom (Padra Serum Alborz Company)

Dose received according to the protocol provided in "Dosing" from the polyvalent antivenom vial of Padra Serum Alborz Company

Group 2: Snakebite antivenom (Razi Vaccine and Serum Research Institute)

Dose received according to the protocol provided in "Dosing" from the polyvalent antivenom ampule of Razi Vaccine and Serum Research Institute

Dosing

Since the main factor in the deterioration of the victim's condition is the amount of toxin entering the body, and since the amount of toxin entered is a factor independent of the patient's age, the therapeutic dose is no different between children and adults. The required amount of antivenom initially depends on the severity of the bite and snake family, and subsequently on the victim's clinical symptoms and paraclinical tests. In the preclinical tests of the product manufactured by Padra Serum Alborz, up to 12 vials per kilogram of mice body weight, no lethality or side effects were observed, but usually up to a maximum of 25 antivenoms can be administered per victim.

The method of antivenom administration and dosing in snakebite victims, according to previous studies and snakebite severity scale, is as follows:

| Severity | Viper bite | Cobra bite |
| --- | --- | --- |
| Mild | **Local:** local swelling of less than 2.5 cm around the bite site  **Systemic:** None  **Number of antivenom needed:** None (Excluded from the study) | **Local**: None  **Systemic:** None  **Number of antivenom needed:** 5 vials of 6-valent |
| Moderate | **Local:**  Complete swelling of each finger, Local swelling of more than 2.5 cm around the bite site, progressive swelling, Considerable pain, tissue destruction (skin or muscle necrosis), Blister formation, Cervical bite, Regional lymphadenitis  **Systemic:**  Intractable vomiting, General weakness, Spontaneous hemorrhage, Cardiac dysrhythmia and abnormal ECG, Increase in serum creatinine, Thrombocytopenia (platelet count less than 150 thousand per cubic millimeter), Increasing coagulation tests (International Normalized Ratio (INR) more than 1.2, PT (Prothrombine Time) more than 20 seconds, PTT (Partial Prothrombine Time) more than 50 seconds)  **Number of antivenom needed:** 5 vials of 5-valent | **Local:**  Any amount of swelling, Pain of any intensity, tissue destruction (skin or muscle necrosis), Blister formation, Cervical bite, Regional lymphadenitis  **Systemic:**  Ptosis, Fasciculation, Paralysis of the external muscles of the eye, Limb paralysis, diplopia  **Number of antivenom needed:** 5 vials of 6-valent |
| Severe | **Local:**  Cervical bite with the possibility of upper airway obstruction, Compartment syndrome  **Systemic:**  Cardiovascular collapse (cardiac arrest, overt shock*, decreased peripheral perfusion), Rhabdomyolysis, Severe active bleeding  **Number of antivenom needed:** 10 vials of 5-valent | **Local:**  Cervical bite with the possibility of upper airway obstruction, Compartment syndrome  **Systemic:**  Medulla paralysis, Respiratory paralysis, Rhabdomyolysis  **Number of antivenom needed:** 10 vials of 6-valent |

If the snake species is not identified and the victim has no obvious clinical manifestation (other than the bite site) and cobras also exist in the geographical area: five antivenoms

If there are no cobras in the geographical area, victims will only receive antivenom when they develop moderate or severe symptoms and signs.

The calculated total antivenoms should be diluted in 5 to 10 milliliters per kilogram of body weight (250 to 500 milliliters) of normal saline and then administered intravenously through a vein in one of the intact limbs (not bitten).

To avoid serious unintended consequences, the initial dose of antivenom is started at a rate of 10 mL per hour, and if no signs and symptoms of hypersensitivity are observed, the dose rate is doubled every few minutes, so that the entire determined antivenom is administered within one hour.

Repeat dosing:

In situations where INR was more than 3, PT more than 20 seconds, PTT more than 50 seconds or platelets less than 25,000 (six hours after administration of the initial dose), or bite site swelling was progressed, systemic symptoms were continued, or neurotoxic, and cardiovascular symptoms were worsened (one hour after administration of the initial dose), five antivenoms were re-administered.

Maintenance dosing should commence upon reaching therapeutic response (cessation of swelling, improvement in neurological symptoms, and resolution of coagulation panel) or after receiving 20 vials of antivenom (whichever comes first). Two vials of antivenom were diluted into 200 ml of sodium chloride 0.9% and infused every 6 hours for three consecutive doses. All adverse events were carefully recorded during the study. The delayed complications (Serum Sickness) were evaluated up to 14 days after the first antivenom infusion.

The patient's vital signs are recorded at the specified study visits and all adverse events (even if unrelated to the study intervention drug) are carefully entered into the eCRF.

Expected adverse reactions from therapeutic antivenoms include early reactions after administration (anaphylactic shock, etc.), reactions more likely to occur within 24 hours (urticaria, itching, fever, hypotension, bronchospasm, etc.) and delayed reactions (serum sickness). The occurrence of any event will be carefully recorded in the victim's eCRF. If an adverse reaction occurs after antivenom administration, necessary measures will be taken by the principal investigator team.

| Treatment | Diagnosis | Adverse effect |
| --- | --- | --- |
| Antibiotic based on Infectious expert consult | Clinical | Cellulitis |
| Unfractionated Heparin 5000 IU QID | Doppler Sonography | Deep Vein Thrombosis (DVT) |
| Iso-group & Iso-Rh Packed cell(2 U stat) | Hemoglubin <7 | Active bleeding |
| Amp Ondansetron 4 mg IV | Clinical | Naseau and vomiting |
| Amp MgSO4 2g IV infusion | ECG analysis | QTc prolongation |
| Fluid therapy | CPK>1000 U/L | Rhabdomyolysis |
| Dialysis based on nephrology consult | Increase in SCr>0.3 mg/dl in 48 hours or urine output<0.5 ml/kg/h | Acute Kidney Injury |
| Fasciotomy  Based on orthopedic consult | Clinical | Compartment syndrome |

Treatment measures in the event of anaphylactic shock:

Immediately stop antivenom injection after observing signs of anaphylactic shock

Intramuscular injection of 0.3-0.5 mg of epinephrine in adults and 0.01 mg/kg in children, if necessary, repeated every 5 to 15 minutes until reaching a favorable hemodynamic status

Administration of H1 and H2 antihistamines (such as chlorpheniramine maleate 10 mg in adults and 0.2 mg/kg in children, cimetidine 200 mg in adults and 4 mg/kg in children)- if using cimetidine, infuse slowly in 20 ml normal saline over 2 minutes

Corticosteroids (intravenous hydrocortisone 100 mg in adults and 2 mg/kg in children)

Treatment should start very quickly at the first signs and symptoms of a reaction such as itching, tachycardia, restlessness or even a few hives.

Continuing antivenom administration after relief of anaphylactic shock symptoms

Outcomes

Primary outcome:

Comparison of the percentage of victims with improved snakebite symptoms up to 48 hours after receiving antivenom based on patients' clinical and paraclinical status including:

a) Stopping the progression of swelling up to 48 hours

b) Normalization of coagulation test status up to 48 hours

c) Stopping the progression of neurological symptoms up to 48 hours.

Each of the above will be evaluated based on the victim's baseline status.

Secondary outcomes:

1. Percentage of adverse reactions in snakebite victims
   - Anaphylactic reactions (48-hour follow-up period): Two or more of the following signs occurring rapidly (within minutes to hours) after antivenom administration in the victim:
     - Skin and mucosal involvement (such as generalized urticaria, itching or flushing, lip swelling, tongue swelling and swelling of the uvula)
     - Respiratory symptoms (such as dyspnea, wheezing, bronchospasm, stridor, hypoxemia)
     - Hypotension (systolic blood pressure less than 90 mmHg in adults) or signs associated with organ dysfunction (such as hypotonia, collapse, syncope, urinary incontinence)
     - Prolonged gastrointestinal symptoms (such as cramping abdominal pain, vomiting)
   - Serum sickness, which is a type III hypersensitivity vasculitis characterized by fever, malaise and itchy rash (urticarial or maculopapular) one to two weeks after antivenom administration. Associated findings include gastrointestinal symptoms, lymphadenopathy, arthralgia, arthritis and peripheral neuropathy, with nonspecific laboratory findings.
   - Any adverse event during the 14-day follow-up period
2. Comparison of antivenom dose received between the two intervention groups

Study Schedule

|  | **Study duration** | | | | | | | | | | |
| --- | --- | --- | --- | --- | --- | --- | --- | --- | --- | --- | --- |
|  | | **Screening and victim classification** |  | **Post-classification interval** | | | | | | | **Study termination** |
| **Visits** | | **Visit 1** | **Visit 2** | **Visit 3** | **Visit 4** | **Visit 5** | **Visit 6** | **Visit 7** | **Visit 8** | **Visit 9** | **Visit 10** |
| ***Time*** | | *At admission* | *30 minutes after injection* | *1 hour±15minutes* | *6 hours±15 minutes* | *12 hours±15minutes* | *24hours±1hour* | *48hours±1hour* | *72hours±1hour or at discharge* | *7 days±1 day* | *14 days±1 day* |
| Screening | | × |  |  |  |  |  |  |  |  |  |
| Consent form | | × |  |  |  |  |  |  |  |  |  |
| Randomization | | × |  |  |  |  |  |  |  |  |  |
| Attending physician vist | | × | × | × | × | × | × | × | × |  |  |
| Snakebite severity symptoms record | | × | × | × | × | × | × | × | × |  |  |
| Paraclinical tests | | × |  | × | × |  | × | × | × | × | × |
| Antivenom administration | | × |  |  |  |  |  |  |  |  |  |
| Supportive care measures | | × |  | × | × | × | × | × | × | × | × |
| Adverse effect record | | × |  | × | × | × | × | × | × | × | × |
| Serum sickness follow-up by phone call | |  |  |  |  |  |  |  |  | × | × |

Paraclinical Examination Schedule

| **Paraclinical Test** | **At admission** | **One hour after antivenom injection** | **Six-hour- post-admission** | **Daily** | **Three-day-post-admission** | **One-week post-admission (as needed)** | **Two-week-post-admission (as needed)** |
| --- | --- | --- | --- | --- | --- | --- | --- |
| **CBC, diff****^+^** | ✓ | ✓ | ✓ | ✓ | ✓ | ✓ | ✓ |
| **‍PT, PTT, INR** | ✓ |  | ✓ | ✓ | ✓ |  |  |
| **Fibrinogen** | ✓ |  | ✓ | ✓ | ✓ |  |  |
| **BS** | ✓ |  |  |  |  |  |  |
| **BUN, Cr** | ✓ |  | ✓ | ✓ | ✓ |  |  |
| **Na, K** | ✓ |  | ✓ | ✓ | ✓ |  |  |
| **CPK** | ✓ |  | ✓ | ✓ | ✓ |  |  |
| **AST,ALT, LDH** | ✓ |  |  | ✓ | ✓ |  |  |
| **Bilirubin(total&direct)** | ✓ |  |  | ✓ | ✓ |  |  |
| **ECG** | ✓ |  | ✓ | ✓ | ✓ |  |  |
| **Urine analysis** | ✓ |  |  |  |  |  |  |
| **C3 , C4 , ESR, CRP^+^** |  |  |  |  |  | ✓ | ✓ |
| **Doppler sonography** | 48 hours after snakebite | | | | | | |

+in case of serum sickness

**Description of study visit events:**

Visit 1 (At admission)

- Evaluate and select victims for the study
- Transfer victim with snakebite claim to emergency department
- Explain the study after reassuring the victim and obtain verbal consent (and ultimately signed consent form)
- Hospital admission
- Examination by physician
- Assess victim for inclusion and exclusion criteria
- Allocate randomized code to victim based on Randomization Sheet available at study site
- Initiate treatment process and administer antivenom according to treatment protocol under attending physician supervision
- Prepare antivenoms by study site nurse and start infusion (attending physician and victim are unaware of the type of antivenom received)
- Accurately record all initial symptoms observed in examinations and paraclinical tests in eCRF
- Record all concurrent care provided to manage bite complications
- Report adverse events and record potential adverse reactions after administration

Visit 2 (30 minutes after injection)

- Examination by physician
- Accurately record all symptoms observed in examinations in eCRF

Visit 3 (1 hour ±15 minutes)

- Examination by physician
- Accurately record all symptoms observed in examinations and paraclinical tests in eCRF
- Assess need for repeat antivenom administration and give antivenom if needed
- Record all concurrent care provided
- Report adverse events and record adverse reactions
- The patient should be visited by the physician every hour until clinical and paraclinical symptoms stop progressing.

Visit 4 (6 hours±15 minutes)

- Examination by physician
- Accurately record all symptoms observed in examinations and paraclinical tests in eCRF
- Assess need for repeat antivenom administration and give antivenom if needed
- Record all concurrent care provided
- If the victim was examined by the physician from 1 to 6 hours after injection (every hour), the symptoms observed and measures taken will be entered in the eCRF
- Report adverse events and record adverse reactions

Visit 5 (12 hours±15 minutes)

- - - - Examination by physician
      - Accurately record all symptoms observed in examinations and paraclinical tests in eCRF
      - Assess need for repeat antivenom administration and give antivenom if needed
      - Record all concurrent care provided
      - If the victim was examined by the physician from 6 to 12 hours after injection (every hour), the symptoms observed and measures taken will be entered in the eCRF
      - Report adverse events and record adverse reactions

Visit 6 (24 hours±1 hour)

- - - - Examination by physician
      - Accurately record all symptoms observed in examinations and paraclinical tests in eCRF
      - Record all concurrent care provided
      - If the victim was examined by the physician from 12 to 24 hours after injection (every hour), the symptoms observed and measures taken will be entered in the eCRF
      - Report adverse events and record adverse reactions

Visit 7 (48 hours ±1 hour)

- Examination by physician
- Accurately record all symptoms observed in examinations and paraclinical tests in eCRF
- Record all concurrent care provided
- If the victim was examined by the physician from 24 to 48 hours after injection (every hour), the symptoms observed and measures taken will be entered in the eCRF
- Report adverse events and record adverse reactions

Visit 8 (72 hours ±1 hour or at discharge)

- Examination by physician
- Accurately record all symptoms observed in examinations and paraclinical tests in eCRF
- Record all concurrent care provided
- If the victim was examined by the physician from 48 to 72 hours after injection (every hour), the symptoms observed and measures taken will be entered in the eCRF
- Report adverse events and record adverse reactions

For victims requiring longer hospitalization (more than 72 hours), the last visit performed in the hospital for eCRF recording is 72 hours after receiving the first dose of antivenom.

Visit 9 (7 day ±1 day)

- - - - Phone call
      - Ask questions related to serum sickness and refer victim to treatment center for paraclinical tests such as CBC, diff, ESR, CRP, C3 and C4 if it has occurred.
      - Record concurrent care
      - Report adverse events and record adverse reactions

Visit 10 (Day 14 ±1 day)

- - - - Phone call
      - Ask questions related to serum sickness and refer victim to treatment center for paraclinical tests such as CBC, diff, ESR, CRP, C3 and C4 if it has occurred.
      - Record concurrent care
      - Report adverse events and record adverse reactions

**Sample size:**

98 victims (with an expected dropout rate of about 15% in data collection related to the primary outcome of the study) are equally (1:1) randomized into two intervention arms. They will achieve 80% statistical power to determine non-inferiority using a one-sided score test (Farrington & Manning). The significance level (alpha) of the statistical test is 0.025. The non-inferiority margin, according to the principal investigator, is set at 0.2, assuming this is 0.9 in the control group. The calculations are attached in Appendix 1.

Randomization

Randomization sequences have been made online (<https://www.sealedenvelope.com/>) using the quadruple blocks for the total sample size of 98 victims (ratio of 1:1). The produced randomization sequences would be located in the study site. Each randomization code have been already labeled on each related anti-venom (2 groups of intervention) and be presented in the study site drug stock. After assurance of victim eligibility and receiving the informed consent, according to the randomization sequence, the specific antivenom would be injected to victims.

Blinding description

All victims after meeting the eligibility criteria are examined by physician. After allocating of randomization code to each victim, the nurse with using the drug stock in the study site (with the research tag on them) prepare the infusion bag for Intravenous infusion. Due to identical appearance of infusion bags and administration process, none of the victims would be aware of the group of intervention. It is tried to minimize the awareness of physician from the type of intervention but it's unavoidable. Importance of blindness is emphasized while education the nurses and physicians. In addition, since the data are documented in eCRF as untitled codes, the data management team would receive the information without the identification of victims. Therefore, blindness in victims and outcome assessor team would be obtained completely and for physician would be partial.

Statistical Methods

To determine the primary effectiveness, the main study approach is the per-protocol approach. Of course, the primary outcome (non-inferiority of the efficacy of Padra serum compared to Razi serum in the intention-to-treat (ITT) population will also be investigated and their results will be reported and compared with each other.

Statistical analysis

Trial profile:

All the participants who provide the informed consent form will be included in the final statistical report. A CONSORT-style plot shows the progression of victims during the trial, from initial screening for eligibility to completion of the final assessment of the primary endpoint. For the treatment group in the per-protocol population, reasons for withdrawal, and major protocol deviations and violations, will be reported as numbers (percentages).

Characteristics of the participants and baseline comparisons:

The baseline characteristics of the victims in two groups are presented descriptively and the possible differences depending on the desired variable are compared using parametric and non-parametric tests (depending on the type of variable and data collected as basic data). In case of a large difference (for example, more than 20% difference in a two-state variable), this imbalance is controlled in random allocation in the final statistical model.

Primary outcome analysis

The study approach in the effectiveness analysis in this study is a non-inferiority approach, so that the lower limit of the 95% confidence limit of the difference in the desired response (improvement of snakebite symptoms) in the antiserum group compared to the Razi group is expected not to exceed the determined margin of 10%. Of course, the study data is also analyzed with the intention-to-treat approach and its results are reported.

Complications analyses

All complication data for each treatment group were analyzed descriptively. The number, percentage, and duration of treatment-related adverse events are calculated by classification based on system organ or type of complication for all adverse events (AEs) and severe adverse events (SAEs).

Adverse events

An adverse event (AE) is described as any medical event that occurs in patients participating in the study and is different from the clinical manifestations of the progression of the underlying injury and does not necessarily have a cause-and-effect relationship with the treatment used in the study. Clinical manifestations that will be reported as adverse effects include any sign, symptom (such as any abnormal laboratory result), or temporary illness that is related to the use of the study drug, whether these effects have a cause-and-effect relationship with study drugs.

Classification of adverse events based on severity:

Adverse events will be classified according to their severity according to the approved guidelines (Common Toxicity Criteria, version 5.0, published on November 27, 2017). For all events not mentioned in the guidelines, mild, moderate, and severe classifications will be used, based on the following definitions.

mild: any reaction, sign or symptom that a person can recognize but does not interfere with his normal activities.

Moderate: Any reaction, sign or symptom that is bothersome enough to significantly interfere with any of the person's usual activities. For this case, medical intervention may be needed.

Severe: Any reaction, sign and symptom that is significantly bothersome enough to significantly interfere with a person's usual activities and cause disability and/or a distinct health risk. For this case, medical intervention is usually required.

Classification of adverse events based on the relationship with the treatment used in the study:

To determine the relationship between the adverse event or adverse drug reaction with the treatment used in the study, the following definitions will be considered:

Certain: A clinical event, including changes in laboratory tests that are related to a logical time order to drug administration, and its occurrence cannot be explained by the person's current illness or other drugs and substances. Response to drug reduction (discontinuation) should be clinically acceptable. This event should be definite from the point of view of pharmacology, and if necessary, the process of re-exposure to the drug should be used.

Probable/likely: A clinical event, including changes in laboratory tests that are related to a reasonable time order to the administration of drugs, and its occurrence is unlikely to be related to the person's current illness or other drugs and substances. And when stopping the drug, a reasonable clinical response is seen. Information on reexposure to the drug is not required to place an event in this group.

Possible: A clinical event, including changes in laboratory tests that are related to the drug administration with a logical time order, but also its occurrence can be explained through the person's current illness or other drugs and substances. Information on drug discontinuation may be missing or unclear.

Unlikely: A clinical event, including changes in laboratory tests that are related to a logical time order to drug administration, but the explanation of its occurrence through the person's current illness or other drugs and substances is more acceptable.

Conditional/unclassified: A clinical event, including changes in laboratory tests, that is reported as an adverse reaction, and it is necessary to obtain more data to properly evaluate it, or additional data in are under review.

Unassessable/Unclassifiable: A warning that suggests the occurrence of an adverse reaction but cannot be judged because the information is insufficient or inconsistent and cannot be complete or confirmed in terms of data.

In case of severe life-threatening adverse events, it must be reported to the sponsor/ethics committee within 24 hours after the researcher becomes aware of the adverse event. In the event of severe adverse events that are not life-threatening, it must be reported to the sponsor/ethics committee within 7 days after the researcher becomes aware of the adverse event. All severe adverse events and the results of follow-ups should be reported to the General Directorate of Medicine within 15 days after being informed to the sponsor.

Conflict of interest statement

Funding organization

All costs related to the study, including the cost of snakebite treatment, including all blood tests, urine tests, possible Doppler ultrasound and ECG, etc.) will be borne by Padra Serum Alborz Company.

Data access:

Only the principal investigator (and, if necessary, the steering committee), monitors, auditors and inspectors of the General Department of Drug and Substance Affairs will have access to all data and databases.

Post-study patient cares:

Secondary care (if necessary and related to the study) will be provided to the participants by the principal investigator and sponsored by the sponsor after the study.
