# Supplementary material for: The SnaFab versus the Razi antivenom for treatment of snakebite envenomation: A randomized, double-blind (investigator and victims), active controlled, non-inferiority clinical trial
Source: PLOS Glob Public Health. 2025 Nov 24;5(11):e0004281. doi: 10.1371/journal.pgph.0004281 (PMC12643280; doi:10.1371/journal.pgph.0004281)
Supplement: S1 Checklist — (DOC) [file pgph.0004281.s003.doc]

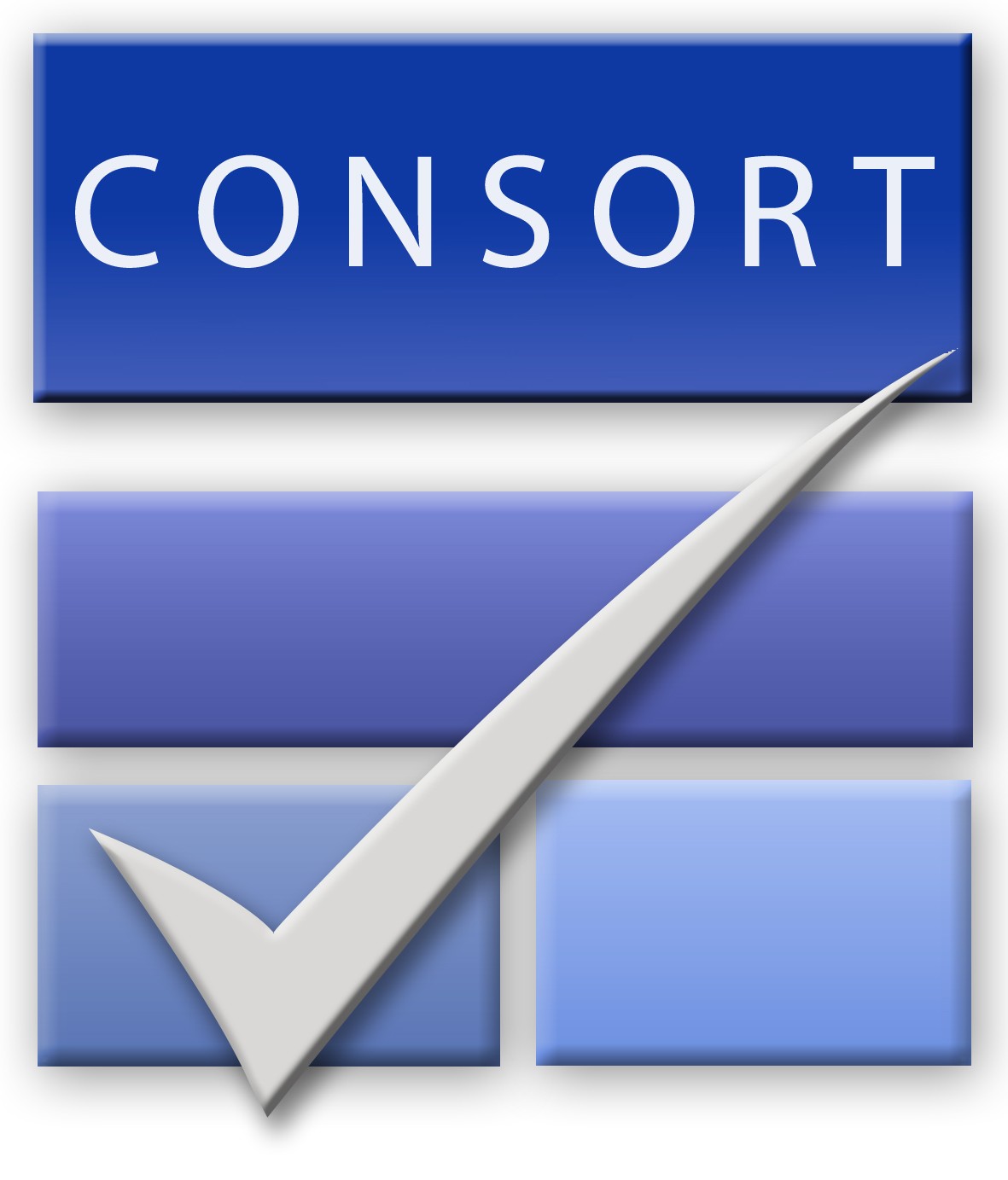
CONSORT 2010 checklist of information to include when reporting a randomised trial*

| Section/Topic | Item No | | Checklist item | Reported on page No |
| --- | --- | --- | --- | --- |
| Title and abstract | | | | |
|  | 1a | | **The SnaFab™ Versus the Razi Antivenom for Treatment of Snakebite Envenomation: A Randomized, Double-Blind (investigator and victims), Active Controlled, Non-inferiority Clinical Trial** |  |
|  | 1b | | **Background**: Snakebite envenoming is a significant public health issue in Iran, and it is crucial to have effective and easily accessible antivenom treatments. This study aimed to evaluate the non-inferiority of the SnaFab™ and the Razi antivenoms for treating snakebite envenoming.  **Methods**: The study was a randomized, double-blind, multicenter, active-controlled, non-inferiority trial involving 98 snakebite victims commenced on April 17th, 2020, and concluded on May 15th, 2021. The patients received either the SnaFab™ or the Razi antivenoms, with the primary endpoint being the recovery rate within 48 hours after antivenom infusion. The secondary endpoints included adverse events over a 14-day monitoring period and total antivenom consumption. The non-inferiority margin (NIM) was set at 20 percent.  **Results**: The recovery rate was 100 percent in the SnaFab™ group, which was non-inferior to the Razi antivenom group (98%). The mean number of antivenom units administered was 8.95 ± 4.40 units in the SnaFab™ group, compared to 9.04 ± 4.49 units in the Razi group (P value: 0.92). Adverse events were reported by 10.2% of individuals in the SnaFab™ group and 20.4% in the Razi group. The most commonly reported adverse event was muscle weakness in the Razi group (8%). Moreover, there were no reports of anaphylactic shock or serum sickness.  **Conclusion**: In summary, this study found that SnaFab™ antivenom is non-inferior to Razi antivenom for treating snakebite envenoming in Iran. SnaFab™ antivenom was also well-tolerated and safe.  Key words: Antivenom, Snakebite, Neglected Tropical Disease, Non-inferiority Trial | 1 |
| Introduction | | | | |
| Background and objectives | 2a | | The global impact of snakebites is substantial, with annual estimates indicating 4.5-5.4 million bite incidents, of which 1.8-2.7 million results in envenomation. According to domestic reports, snakebite is a concerning health issue in Iran affecting 4500-6500 individuals annually. In Iran, snakebites lead to approximately 3 to 12 deaths annually, which translates to a rate of 0.003 to 0.01 snakebite deaths per 100,000 individuals each year. Non-inferiority trials, such as the one being discussed, aim to determine if a new treatment is at least as effective as the established one by a predetermined margin. | 2 |
|  | 2b | | This clinical trial aims to evaluate the non-inferiority of the SnaFab™ compared to the Razi antivenom in treating snakebite envenoming in Iran. By setting a clear non-inferiority margin, this trial aims to determine whether SnaFab™ can be considered a non-inferior alternative to the existing standard of care. Should SnaFab™ meet this benchmark, it could offer other benefits such as enhanced accessibility, fewer adverse effects, and greater cost-effectiveness, making it a superior choice in certain circumstances. | 3 |
| Methods | | | | |
| Trial design | 3a | | This was a randomized, double-blind (investigator and victim), active-controlled, parallel non-inferiority trial | 4 |
|  | 3b | | None |  |
| Participants | 4a | | Participants aged 2-60 years who had a history of snakebite, required antivenom based on the severity scale (Table 1), presented within 12 hours of the bite, and provided informed consent (adhering the situation in urgent patients settings) were included to the study and victims who had a history of horse serum allergy, previous snakebite or scorpion sting with antivenom administration, multiple bites, pre-hospital wound manipulation, life-threatening bleeding, marine snakebite, mechanical ventilation requirement, anticoagulant usage, coagulation disorders, significant comorbidities (cardiac, neuromuscular, renal, hepatic), pregnancy or lactation, or prior antivenom administration before reaching the hospital were excluded from the study. | 4 |
|  | 4b | | It was conducted at Imam Reza Hospital in Mashhad, Ayatollah Taleghani Hospital in Urmia, and Razi Hospital in Ahvaz. | 4 |
| Interventions | 5 | | Antivenom dosing was determined based on the snakebite severity scale. Victims were given 5-valent antivenom in Urmia and Ahvaz covering vipers and 6-valent antivenom in Mashhad due to the existence of both vipers and cobras. According to the snakebite severity scale, all victims with moderate and severe symptoms were prescribed 5 and 10 vials of antivenom, respectively. Cobra bite victims, which might have occurred in Imam Reza Hospital in Mashhad, who did not exhibit clinical signs and symptoms (mild symptoms) were prescribed five vials of 6-valent antivenoms. All prescribed vials were diluted in sodium chloride 0.9% and infused intravenously. The victims were closely monitored 30 minutes, 1, 6, 12, 24, 48, and 72 hours after the initial administration. In situations where INR was more than 3, PT more than 20 seconds, PTT more than 50 seconds or platelets less than 25,000 (six hours after administration of the initial dose), or bite site swelling was progressed, systemic symptoms were continued, or neurotoxic, and cardiovascular symptoms were worsened (one hour after administration of the initial dose), five antivenoms were re-administered. Maintenance dosing should commence upon reaching therapeutic response (cessation of swelling, improvement in neurological symptoms, and resolution of coagulation panel) or after receiving 20 vials of antivenom (whichever comes first). Two vials of antivenom were diluted into 200 ml of sodium chloride 0.9% and infused every 6 hours for three consecutive doses. All adverse events were carefully recorded during the study. The delayed complications (Serum Sickness) were evaluated up to 14 days after the first antivenom infusion. | 4 |
| Outcomes | 6a | | The primary objective was to determine the proportion of victims who had recovered within 48 hours post-antivenom infusion. Recovery was defined as the cessation of swelling progression, coagulation abnormalities recovery (PT<20, INR<1.2, PTT<50, PLT>150000), or the cessation of neurotoxicity progression. Responders were those who met at least three recovery criteria. Secondary outcomes included the proportion of patients who experienced adverse events and the total amount of antivenom administration. | 5 |
|  | 6b | | Not Applicable |  |
| Sample size | 7a | | To ensure 81% power to detect the defined non-inferiority margin difference (0.20), 42 patients in each group would be recruited, assuming a 15% dropout rate. Therefore, 98 victims were participated in the trial. | 5 |
|  | 7b | | Not applicable |  |
| Randomisation: |  | |  |  |
| Sequence generation | 8a | | A computer-generated block randomization method was used in the trial (<https://www.sealedenvelope.com/>). | 5 |
|  | 8b | | Block randomization | 5 |
| Allocation concealment mechanism | 9 | | The randomization sequence was concealed by an independent contract research organization (CRO). Neither the participants nor the investigators knew which intervention groups the participants were assigned. | 5 |
| Implementation | 10 | | Independent CRO concealed sequence | 5 |
| Blinding | 11a | | In the efficacy study, all victims were examined by a physician at the study site after assessing their eligibility for inclusion in the study. Once the intervention group for each victim was determined using a pre-prepared randomization chain, the responsible nurse prepared the infusion bag using the study site’s drug stock and administered the intravenous infusion.  Given the identical appearance of the infusion bags and the administration process, none of the victims were aware of their treatment group. Additionally, efforts were made to ensure that the treating physician was as uninformed as possible about the antivenom group received. This was emphasized in the training provided to the nurses and the physician (thus, although there was a possibility of the physician being aware of the assigned treatment, this risk was minimized due to the presence of a treating physician and prior training before the study began). Finally, since the study information recorded in the eCRF was provided to the data management team in the form of codes lacking identifying information about the victims, the blinding process was fully implemented at the subject level and the study result analysis team, and as much as possible, it was also implemented at the treating physician level in a relative manner | 5 |
|  | 11b | | Not Aoolicable |  |
| Statistical methods | 12a | | The primary analysis was based on a per-protocol (PP) approach, which included only patients who completed the study without significant protocol deviations. The non-inferiority margin (NIM) was set at 0.20, and the difference in treatment response rates between the SnaFab™ and Razi antivenom groups would be calculated with a 95% confidence interval (CI). | 6 |
|  | 12b | | Secondary endpoints, such as the total number of administered antivenom and adverse event rates, were analyzed using appropriate statistical tests (e.g., chi-square test for categorical variables, t-test, or Mann-Whitney U test for continuous variables) with significance set at p< 0.05. All analyses were conducted using "SPSS". | 6 |
| Results | | | | |
| Participant flow (a diagram is strongly recommended) | 13a | | 147 individuals were screened, with only 98 eligible with 1:1 ratio randomisation. | 6 |
|  | 13b | | CONSORT flow diagram included | 7 |
| Recruitment | 14a | | Recruitment for the study commenced on April 17th, 2020, and concluded on May 15th, 2021. | 6 |
|  | 14b | | Due to study conclusion | 6 |
| Baseline data | 15 | | Provided in Table 2 | 17 |
| Numbers analysed | 16 | | 147 individuals were screened, with only 98 eligible randomised in 1:1 ratio | 6 |
| Outcomes and estimation | 17a | | Forty-eight out of 49 participants in the Razi arm (%98) and 49 out of 49 participants in the SnaFab™ arm (%100) showed recovery within 48 hours after antivenom infusion and not exceeding the NIM. The 95% confidence interval showed that the percentage of patients who recovered in the Razi arm was 0.02 higher (diff: 0.02 [95% CI: -0.02 to 0.06]).  All patients in both SnaFab and groups presented with local symptoms including pain and swelling. Upon admission, 35 patients (71.4%) in the Razi group and 26 patients (53.1%) in the SnaFab group exhibited systemic manifestations in addition to local symptoms, including coagulation disorders, thrombocytopenia, and general weakness. Severe symptoms, such as active severe hemorrhage and compartment syndrome, were observed in 3 patients (6.12%) from the SnaFab group and 2 patients (4.08%) from the Razi group. Additionally, according to the snakebite severity scale, moderate symptoms were documented in 46 patients (93.8%) from the Padra Serum Alborz group and 47 patients (95.9%) from the Razi Serum group. | 7 |
|  | 17b | | - Primary Outcome (Recovery Rate):   Absolute numbers and percentages:  SnaFab™ group: 49/49 (100%)  Razi group: 48/49 (98%)  Relative difference:  Difference of 0.02 [95% CI: -0.02 to 0.06]   - Adverse Events:   Absolute numbers and percentages:  SnaFab™ group: 5/49 (10.20%)  Razi group: 10/49 (20.40%) | 7-8 |
| Ancillary analyses | 18 | | *Secondary Endpoints*: According to the study, the average amount of antivenom administered to victims in the SnaFab™ group (8.95 ± 4.40 units) and the Razi group (9.04 ± 4.49 units) did not show a significant difference (P value: 0.92). Of the 93 patients presenting with moderate envenomation symptoms according to the snakebite severity scale, 46 patients were treated in the SnaFab group and 47 patients in the Razi group. The total antivenom consumption was 394 vials in the SnaFab group and 402 ampoules in the Razi group (P value: 0.71). Among the study population, severe envenomation was documented in 5 cases at presentation. The SnaFab cohort included three such cases (patients #1, #4, and #44) requiring 20, 15, and 10 vials respectively, while the Razi cohort included two cases (patients #50 and #63) requiring 10 and 15 vials respectively. The difference in antivenom requirements between groups was not statistically significant (P=0.59).  The adverse events reported during the follow-up period are presented in Table 3. During the follow-up period, 10 out of 49 individuals (20.40%) in the Razi group reported adverse events, while 5 out of 49 individuals (10.20%) in the SnaFab™ group reported adverse events. However, there were no cases of anaphylactic shock reported in either group. The Razi group reported muscle weakness as the most common adverse event, while no delayed hypersensitivity reactions, including serum sickness, were reported in either group. | 8 |
| Harms | 19 | | Adverse events detailed in Table 3 | 18 |
| Discussion | | | | |
| Limitations | 20 | | Pandemic-related: Implementing the trial coincided with the COVID-19 pandemic, which took longer than expected to reach the desired sample size.  Methodological-related: Most participants were male, which may reflect the higher risk of snakebite among males due to their increased exposure to outdoor activities. However, it is essential to note that gender differences were not statistically significant between the two groups, indicating that the randomization process successfully created comparable groups regarding gender distribution. The SnaFab™ group's mean age was significantly higher than the Razi group. While this difference may be a potential confounding factor, it is unlikely to impact the study results significantly. | 10 |
| Generalisability | 21 | | The average amount of antivenom in both groups showed no significant differences (P value=0.92). It suggests both antivenom require similar dosing regimens and may imply comparable potency between products. Moreover, the interchangeability of products is supported. This data is particularly valuable for: healthcare policy makers, clinical protocol development, hospital formulary decisions, resource allocation planning and cost-effectiveness analyses. | 10 |
| Interpretation | 22 | | The interpretation is consistent with the study results, showing:  Benefits:  SnaFab™ demonstrated non-inferiority to Razi antivenom (recovery rates: 100% vs 98%)  Lower adverse event rate with SnaFab™ (10.2% vs 20.4%)  Similar antivenom dosing requirements between groups (8.95 vs 9.04 units, p=0.92)  Harms:  No severe adverse events (anaphylactic shock or serum sickness) in either group  Minor adverse events were reported in both groups but were less frequent with SnaFab™  Other Evidence:  Results align with existing literature on caprylic acid fractionation benefits  Adverse event rates were lower than reported in comparable trials  Findings support WHO’s goals for improving snakebite treatment access  The interpretation appropriately balances the demonstrated non-inferiority with safety considerations while acknowledging the need for post-marketing surveillance. |  |
| Other information | | | |  |
| Registration | 23 | | It received approval from the Iranian Food and Drug Administration (IFDA) and Ethical Committees and was registered at the Iranian Registration of Clinical Trials (IRCT20180515039672N2, <https://irct.behdasht.gov.ir/trial/41983>). | 5 |
| Protocol | 24 | | The datasets generated during and/or analyzed during the current study are available from the corresponding author on reasonable request. |  |
| Funding | 25 | This research was funded by “Padra Serum Alborz Company” grant number 99/20880 and the APC was funded by “Padra Serum Alborz Company” as well. | |  |

Citation: Schulz KF, Altman DG, Moher D, for the CONSORT Group. CONSORT 2010 Statement: updated guidelines for reporting parallel group randomised trials. BMC Medicine. 2010;8:18.
© 2010 Schulz et al. This is an Open Access article distributed under the terms of the Creative Commons Attribution License (<http://creativecommons.org/licenses/by/2.0>), which permits unrestricted use, distribution, and reproduction in any medium, provided the original work is properly cited.

*We strongly recommend reading this statement in conjunction with the CONSORT 2010 Explanation and Elaboration for important clarifications on all the items. If relevant, we also recommend reading CONSORT extensions for cluster randomised trials, non-inferiority and equivalence trials, non-pharmacological treatments, herbal interventions, and pragmatic trials. Additional extensions are forthcoming: for those and for up-to-date references relevant to this checklist, see [www.consort-statement.org](http://www.consort-statement.org/).
