# Supplementary material for: The SnaFab versus the Razi antivenom for treatment of snakebite envenomation: A randomized, double-blind (investigator and victims), active controlled, non-inferiority clinical trial
Source: PLOS Glob Public Health. 2025 Nov 24;5(11):e0004281. doi: 10.1371/journal.pgph.0004281 (PMC12643280; doi:10.1371/journal.pgph.0004281)
Supplement: S1 Data — (DOCX) [file pgph.0004281.s004.docx]

## Table 1 – Age, gender, and time of admission distribution of victims

| ID | Code | Group | Center | Gender | Age | Date of biting | Admission Date |
| --- | --- | --- | --- | --- | --- | --- | --- |
| 1 | YUAB | SnaFab | AHV | M | 31 | Apr 2020 | Same day |
| 2 | MAGH | SnaFab | AHV | M | 13 | Apr 2020 | Same day |
| 3 | ALSA | SnaFab | AHV | M | 31 | May 2020 | Same day |
| 4 | GHMA | SnaFab | AHV | M | 30 | May 2020 | Same day |
| 5 | ABAR | SnaFab | AHV | M | 37 | May 2020 | Same day |
| 6 | HABA | SnaFab | AHV | M | 23 | May 2020 | Same day |
| 7 | FASO | SnaFab | AHV | M | 38 | May 2020 | Same day |
| 8 | RABA | SnaFab | AHV | M | 24 | May 2020 | Same day |
| 9 | HAHO | SnaFab | AHV | M | 30 | May 2020 | Same day |
| 10 | REAB | SnaFab | AHV | M | 19 | May 2020 | Same day |
| 11 | ALSE | SnaFab | MAS | M | 60 | June 2020 | Same day |
| 12 | BAEB | SnaFab | URM | M | 35 | June 2020 | Same day |
| 13 | FAMA | SnaFab | AHV | F | 40 | July 2020 | Same day |
| 14 | ALHA | SnaFab | AHV | M | 32 | July 2020 | Same day |
| 15 | SASH | SnaFab | AHV | M | 30 | July 2020 | Same day |
| 16 | EEZ9 | SnaFab | MAS | M | 27 | July 2020 | Same day |
| 17 | DDH8 | Razi | MAS | F | 45 | July 2020 | Same day |
| 18 | QQG5 | Razi | MAS | F | 30 | July 2020 | Same day |
| 19 | XXA1 | SnaFab | MAS | M | 15 | Aug 2020 | Same day |
| 20 | SSV7 | Razi | MAS | M | 39 | Aug 2020 | Same day |
| 21 | BBM6 | Razi | MAS | M | 17 | Aug 2020 | Same day |
| 22 | YYY4 | SnaFab | MAS | M | 53 | Aug 2020 | Same day |
| 23 | ZZI1 | SnaFab | URM | M | 49 | Aug 2020 | Same day |
| 24 | SSU8 | SnaFab | MAS | M | 25 | Aug 2020 | Same day |
| 25 | NNZ3 | Razi | URM | M | 35 | Aug 2020 | Same day |
| 26 | FFG3 | SnaFab | AHV | M | 52 | Aug 2020 | Same day |
| 27 | IIE0 | Razi | AHV | M | 27 | Sep 2020 | Same day |
| 28 | EEG0 | Razi | AHV | F | 21 | Aug 2020 | Same day |
| 29 | JJW4 | SnaFab | AHV | M | 27 | Sep 2020 | Same day |
| 30 | BBI6 | SnaFab | AHV | M | 21 | Sep 2020 | Same day |
| 31 | AAH7 | SnaFab | AHV | M | 17 | Sep 2020 | Same day |
| 32 | AAF9 | Razi | AHV | M | 27 | Sep 2020 | Same day |
| 33 | WWR1 | Razi | AHV | M | 27 | Sep 2020 | Same day |
| 34 | RRS3 | SnaFab | AHV | F | 36 | Sep 2020 | Same day |
| 35 | JJI8 | Razi | AHV | F | 29 | Sep 2020 | Same day |
| 36 | QQW3 | SnaFab | AHV | M | 15 | Sep 2020 | Same day |
| 37 | AAT1 | SnaFab | MAS | F | 52 | Sep 2020 | Same day |
| 38 | VVI7 | Razi | AHV | F | 27 | Sep 2020 | Same day |
| 39 | UUL5 | SnaFab | AHV | M | 31 | Sep 2020 | Same day |
| 40 | QQQ3 | Razi | AHV | M | 29 | Sep 2020 | Same day |
| 41 | UUN5 | Razi | AHV | M | 32 | Sep 2020 | Same day |
| 42 | XXN0 | SnaFab | AHV | M | 58 | Sep 2020 | Same day |
| 43 | RRA4 | Razi | AHV | F | 46 | Sep 2020 | Same day |
| 44 | DDN5 | SnaFab | AHV | M | 30 | Sep 2020 | Same day |
| 45 | OOZ0 | Razi | AHV | M | 27 | Sep 2020 | Same day |
| 46 | UUT4 | SnaFab | AHV | M | 37 | Sep 2020 | Same day |
| 47 | GGM9 | SnaFab | AHV | F | 37 | Sep 2020 | Same day |
| 48 | SSH4 | SnaFab | AHV | M | 30 | Sep 2020 | Same day |
| 49 | FFW7 | Razi | AHV | M | 45 | Sep 2020 | Same day |
| 50 | MMM9 | Razi | AHV | M | 18 | Sep 2020 | Same day |
| 51 | KKZ6 | SnaFab | AHV | M | 28 | Sep 2020 | Same day |
| 52 | KKW6 | Razi | AHV | M | 35 | Sep 2020 | Same day |
| 53 | EEP5 | Razi | AHV | M | 54 | Sep 2020 | Same day |
| 54 | FFR9 | SnaFab | AHV | M | 22 | Sep 2020 | Same day |
| 55 | UUN6 | Razi | AHV | F | 3 | Sep 2020 | Same day |
| 56 | BBP7 | SnaFab | MAS | F | 38 | Sep 2020 | Same day |
| 57 | OOE4 | Razi | AHV | F | 6 | Sep 2020 | Same day |
| 58 | QQA5 | SnaFab | AHV | M | 46 | Sep 2020 | Same day |
| 59 | FFT8 | Razi | MAS | M | 15 | Sep 2020 | Same day |
| 60 | JJY3 | SnaFab | AHV | M | 25 | Sep 2020 | Same day |
| 61 | GGN0 | Razi | AHV | F | 46 | Sep 2020 | Same day |
| 62 | zzQ0 | SnaFab | AHV | M | 48 | Sep 2020 | Same day |
| 63 | JJK9 | Razi | AHV | M | 21 | Oct 2020 | Same day |
| 64 | YYE2 | SnaFab | AHV | M | 16 | Oct 2020 | Same day |
| 65 | TTJ2 | Razi | MAS | M | 18 | Oct 2020 | Same day |
| 66 | GG0 | SnaFab | AHV | F | 39 | Oct 2020 | Same day |
| 67 | NN5 | Razi | AHV | M | 29 | Oct 2020 | Same day |
| 68 | JX8 | Razi | AHV | M | 33 | Oct 2020 | Same day |
| 69 | QU4 | Razi | AHV | M | 21 | Oct 2020 | Same day |
| 70 | EEJ8 | Razi | URM | M | 44 | Oct 2020 | Same day |
| 71 | WT3 | Razi | AHV | M | 8 | Oct 2020 | Same day |
| 72 | DC7 | SnaFab | AHV | M | 36 | Oct 2020 | Same day |
| 73 | BK8 | Razi | AHV | M | 30 | Oct 2020 | Same day |
| 74 | ZK8 | Razi | AHV | F | 30 | Nov 2020 | Same day |
| 75 | FM4 | Razi | AHV | M | 43 | Nov 2020 | Same day |
| 76 | YP2 | SnaFab | AHV | M | 48 | Dec 2020 | Same day |
| 77 | GK9 | Razi | AHV | F | 38 | Dec 2020 | Same day |
| 78 | FC3 | Razi | AHV | F | 47 | Dec 2020 | Same day |
| 79 | UT8 | Razi | AHV | M | 34 | Feb 2021 | Same day |
| 80 | QS6 | Razi | AHV | M | 15 | Feb 2021 | Same day |
| 81 | RL9 | Razi | AHV | M | 31 | Apr 2021 | Same day |
| 82 | YF3 | SnaFab | AHV | M | 50 | Apr 2021 | Same day |
| 83 | NNK8 | SnaFab | MAS | M | 40 | Apr 2021 | Same day |
| 84 | KL5 | Razi | AHV | F | 17 | Apr 2021 | Same day |
| 85 | RU5 | SnaFab | AHV | M | 39 | Apr 2021 | Same day |
| 86 | OG6 | Razi | AHV | M | 18 | Apr 2021 | Same day |
| 87 | RU0 | Razi | AHV | M | 21 | Apr 2021 | Same day |
| 88 | CH8 | Razi | AHV | M | 6 | Apr 2021 | Same day |
| 89 | VL2 | SnaFab | AHV | M | 23 | Apr 2021 | Same day |
| 90 | DC8 | Razi | AHV | M | 14 | Apr 2021 | Same day |
| 91 | HV6 | Razi | AHV | M | 60 | Apr 2021 | Same day |
| 92 | FL2 | Razi | AHV | M | 31 | Apr 2021 | Same day |
| 93 | RS1 | SnaFab | AHV | F | 51 | Apr 2021 | Same day |
| 94 | AJ6 | Razi | AHV | M | 43 | Apr 2021 | Same day |
| 95 | ZY8 | Razi | AHV | M | 30 | Apr 2021 | Same day |
| 96 | YB6 | SnaFab | AHV | F | 42 | Apr 2021 | Same day |
| 97 | GS2 | Razi | AHV | M | 18 | Apr 2021 | Same day |
| 98 | WZ7 | Razi | AHV | F | 25 | May 2021 | Same day |

## Table 2 – Sequence of patient admissions at the Ahvaz center

| ID | Code | Group | Center | Gender | Age | Date of biting | Admission Date | Phase |
| --- | --- | --- | --- | --- | --- | --- | --- | --- |
| 1 | YUAB | SnaFab | AHV | M | 31 | Apr 2020 | Same day | Safety |
| 2 | MAGH | SnaFab | AHV | M | 13 | Apr 2020 | Same day | Safety |
| 3 | ALSA | SnaFab | AHV | M | 31 | May 2020 | Same day | Safety |
| 4 | GHMA | SnaFab | AHV | M | 30 | May 2020 | Same day | Safety |
| 5 | ABAR | SnaFab | AHV | M | 37 | May 2020 | Same day | Safety |
| 6 | HABA | SnaFab | AHV | M | 23 | May 2020 | Same day | Safety |
| 7 | FASO | SnaFab | AHV | M | 38 | May 2020 | Same day | Safety |
| 8 | RABA | SnaFab | AHV | M | 24 | May 2020 | Same day | Safety |
| 9 | HAHO | SnaFab | AHV | M | 30 | May 2020 | Same day | Safety |
| 10 | REAB | SnaFab | AHV | M | 19 | May 2020 | Same day | Safety |
| 13 | FAMA | SnaFab | AHV | F | 40 | July 2020 | Same day | Safety |
| 14 | ALHA | SnaFab | AHV | M | 32 | July 2020 | Same day | Safety |
| 15 | SASH | SnaFab | AHV | M | 30 | July 2020 | Same day | Safety |
| 26 | FFG3 | SnaFab | AHV | M | 52 | Aug 2020 | Same day | Safety and Efficacy |
| 27 | IIE0 | Razi | AHV | M | 27 | Sep 2020 | Same day | Safety and Efficacy |
| 28 | EEG0 | Razi | AHV | F | 21 | Aug 2020 | Same day | Safety and Efficacy |
| 29 | JJW4 | SnaFab | AHV | M | 27 | Sep 2020 | Same day | Safety and Efficacy |
| 30 | BBI6 | SnaFab | AHV | M | 21 | Sep 2020 | Same day | Safety and Efficacy |
| 31 | AAH7 | SnaFab | AHV | M | 17 | Sep 2020 | Same day | Safety and Efficacy |
| 32 | AAF9 | Razi | AHV | M | 27 | Sep 2020 | Same day | Safety and Efficacy |
| 33 | WWR1 | Razi | AHV | M | 27 | Sep 2020 | Same day | Safety and Efficacy |
| 34 | RRS3 | SnaFab | AHV | F | 36 | Sep 2020 | Same day | Safety and Efficacy |
| 35 | JJI8 | Razi | AHV | F | 29 | Sep 2020 | Same day | Safety and Efficacy |
| 36 | QQW3 | SnaFab | AHV | M | 15 | Sep 2020 | Same day | Safety and Efficacy |
| 38 | VVI7 | Razi | AHV | F | 27 | Sep 2020 | Same day | Safety and Efficacy |
| 39 | UUL5 | SnaFab | AHV | M | 31 | Sep 2020 | Same day | Safety and Efficacy |
| 40 | QQQ3 | Razi | AHV | M | 29 | Sep 2020 | Same day | Safety and Efficacy |
| 41 | UUN5 | Razi | AHV | M | 32 | Sep 2020 | Same day | Safety and Efficacy |
| 42 | XXN0 | SnaFab | AHV | M | 58 | Sep 2020 | Same day | Safety and Efficacy |
| 43 | RRA4 | Razi | AHV | F | 46 | Sep 2020 | Same day | Safety and Efficacy |
| 44 | DDN5 | SnaFab | AHV | M | 30 | Sep 2020 | Same day | Safety and Efficacy |
| 45 | OOZ0 | Razi | AHV | M | 27 | Sep 2020 | Same day | Safety and Efficacy |
| 46 | UUT4 | SnaFab | AHV | M | 37 | Sep 2020 | Same day | Safety and Efficacy |
| 47 | GGM9 | SnaFab | AHV | F | 37 | Sep 2020 | Same day | Safety and Efficacy |
| 48 | SSH4 | SnaFab | AHV | M | 30 | Sep 2020 | Same day | Safety and Efficacy |
| 49 | FFW7 | Razi | AHV | M | 45 | Sep 2020 | Same day | Safety and Efficacy |
| 50 | MMM9 | Razi | AHV | M | 18 | Sep 2020 | Same day | Safety and Efficacy |
| 51 | KKZ6 | SnaFab | AHV | M | 28 | Sep 2020 | Same day | Safety and Efficacy |
| 52 | KKW6 | Razi | AHV | M | 35 | Sep 2020 | Same day | Safety and Efficacy |
| 53 | EEP5 | Razi | AHV | M | 54 | Sep 2020 | Same day | Safety and Efficacy |
| 54 | FFR9 | SnaFab | AHV | M | 22 | Sep 2020 | Same day | Safety and Efficacy |
| 55 | UUN6 | Razi | AHV | F | 3 | Sep 2020 | Same day | Safety and Efficacy |
| 57 | OOE4 | Razi | AHV | F | 6 | Sep 2020 | Same day | Safety and Efficacy |
| 58 | QQA5 | SnaFab | AHV | M | 46 | Sep 2020 | Same day | Safety and Efficacy |
| 60 | JJY3 | SnaFab | AHV | M | 25 | Sep 2020 | Same day | Safety and Efficacy |
| 61 | GGN0 | Razi | AHV | F | 46 | Sep 2020 | Same day | Safety and Efficacy |
| 62 | zzQ0 | SnaFab | AHV | M | 48 | Sep 2020 | Same day | Safety and Efficacy |
| 63 | JJK9 | Razi | AHV | M | 21 | Oct 2020 | Same day | Safety and Efficacy |
| 64 | YYE2 | SnaFab | AHV | M | 16 | Oct 2020 | Same day | Safety and Efficacy |
| 66 | GG0 | SnaFab | AHV | F | 39 | Oct 2020 | Same day | Safety and Efficacy |
| 67 | NN5 | Razi | AHV | M | 29 | Oct 2020 | Same day | Safety and Efficacy |
| 68 | JX8 | Razi | AHV | M | 33 | Oct 2020 | Same day | Safety and Efficacy |
| 69 | QU4 | Razi | AHV | M | 21 | Oct 2020 | Same day | Safety and Efficacy |
| 71 | WT3 | Razi | AHV | M | 8 | Oct 2020 | Same day | Safety and Efficacy |
| 72 | DC7 | SnaFab | AHV | M | 36 | Oct 2020 | Same day | Safety and Efficacy |
| 73 | BK8 | Razi | AHV | M | 30 | Oct 2020 | Same day | Safety and Efficacy |
| 74 | ZK8 | Razi | AHV | F | 30 | Oct 2020 | Same day | Safety and Efficacy |
| 75 | FM4 | Razi | AHV | M | 43 | Oct 2020 | Same day | Safety and Efficacy |
| 76 | YP2 | SnaFab | AHV | M | 48 | Dec 2020 | Same day | Safety and Efficacy |
| 77 | GK9 | Razi | AHV | F | 38 | Dec 2020 | Same day | Safety and Efficacy |
| 78 | FC3 | Razi | AHV | F | 47 | Dec 2020 | Same day | Safety and Efficacy |
| 79 | UT8 | Razi | AHV | M | 34 | Feb 2021 | Same day | Safety and Efficacy |
| 80 | QS6 | Razi | AHV | M | 15 | Feb 2021 | Same day | Safety and Efficacy |
| 81 | RL9 | Razi | AHV | M | 31 | Apr 2021 | Same day | Safety and Efficacy |
| 82 | YF3 | SnaFab | AHV | M | 50 | Apr 2021 | Same day | Safety and Efficacy |
| 84 | KL5 | Razi | AHV | F | 17 | Apr 2021 | Same day | Safety and Efficacy |
| 85 | RU5 | SnaFab | AHV | M | 39 | Apr 2021 | Same day | Safety and Efficacy |
| 86 | OG6 | Razi | AHV | M | 18 | Apr 2021 | Same day | Safety and Efficacy |
| 87 | RU0 | Razi | AHV | M | 21 | Apr 2021 | Same day | Safety and Efficacy |
| 88 | CH8 | Razi | AHV | M | 6 | Apr 2021 | Same day | Safety and Efficacy |
| 89 | VL2 | SnaFab | AHV | M | 23 | Apr 2021 | Same day | Safety and Efficacy |
| 90 | DC8 | Razi | AHV | M | 14 | Apr 2021 | Same day | Safety and Efficacy |
| 91 | HV6 | Razi | AHV | M | 60 | Apr 2021 | Same day | Safety and Efficacy |
| 92 | FL2 | Razi | AHV | M | 31 | Apr 2021 | Same day | Safety and Efficacy |
| 93 | RS1 | SnaFab | AHV | F | 51 | Apr 2021 | Same day | Safety and Efficacy |
| 94 | AJ6 | Razi | AHV | M | 43 | Apr 2021 | Same day | Safety and Efficacy |
| 95 | ZY8 | Razi | AHV | M | 30 | Apr 2021 | Same day | Safety and Efficacy |
| 96 | YB6 | SnaFab | AHV | F | 42 | Apr 2021 | Same day | Safety and Efficacy |
| 97 | GS2 | Razi | AHV | M | 18 | Apr 2021 | Same day | Safety and Efficacy |
| 98 | WZ7 | Razi | AHV | F | 25 | May 2021 | Same day | Safety and Efficacy |

## Table 3 – Sequence of patient admissions at the Mashhad center

| ID | Code | Group | Center | Gender | Age | Date of biting | Admission Date | Phase |
| --- | --- | --- | --- | --- | --- | --- | --- | --- |
| 11 | ALSE | SnaFab | MAS | M | 60 | June 2020 | Same day | Safety |
| 16 | EEZ9 | SnaFab | MAS | M | 27 | July 2020 | Same day | Safety and Efficacy |
| 17 | DDH8 | Razi | MAS | F | 45 | July 2020 | Same day | Safety and Efficacy |
| 18 | QQG5 | Razi | MAS | F | 30 | July 2020 | Same day | Safety and Efficacy |
| 19 | XXA1 | SnaFab | MAS | M | 15 | Aug 2020 | Same day | Safety and Efficacy |
| 20 | SSV7 | Razi | MAS | M | 39 | Aug 2020 | Same day | Safety and Efficacy |
| 21 | BBM6 | Razi | MAS | M | 17 | Aug 2020 | Same day | Safety and Efficacy |
| 22 | YYY4 | SnaFab | MAS | M | 53 | Aug 2020 | Same day | Safety and Efficacy |
| 24 | SSU8 | SnaFab | MAS | M | 25 | Aug 2020 | Same day | Safety and Efficacy |
| 37 | AAT1 | SnaFab | MAS | F | 52 | Sep 2020 | Same day | Safety and Efficacy |
| 56 | BBP7 | SnaFab | MAS | F | 38 | Sep 2020 | Same day | Safety and Efficacy |
| 59 | FFT8 | Razi | MAS | M | 15 | Sep 2020 | Same day | Safety and Efficacy |
| 65 | TTJ2 | Razi | MAS | M | 18 | Oct 2020 | Same day | Safety and Efficacy |
| 83 | NNK8 | SnaFab | MAS | M | 40 | Apr 2021 | Same day | Safety and Efficacy |

## Table 4 – Sequence of patient admissions at the Urmia center

| ID | Code | Group | Center | Gender | Age | Date of biting | Admission Date | Phase |
| --- | --- | --- | --- | --- | --- | --- | --- | --- |
| 12 | BAEB | SnaFab | URM | M | 35 | June 2020 | Same day | Safety |
| 23 | ZZI1 | SnaFab | URM | M | 49 | Aug 2020 | Same day | Safety and Efficacy |
| 25 | NNZ3 | Razi | URM | M | 35 | Aug 2020 | Same day | Safety and Efficacy |
| 70 | EEJ8 | Razi | URM | M | 44 | Oct 2020 | Same day | Safety and Efficacy |

## Table 5 – Snakebite distribution site

| ID | Code | Group | Center | Bite Site |
| --- | --- | --- | --- | --- |
| 1 | YUAB | SnaFab | AHV | Left upper limbs |
| 2 | MAGH | SnaFab | AHV | Left lower limbs |
| 3 | ALSA | SnaFab | AHV | Right lower limbs |
| 4 | GHMA | SnaFab | AHV | Left lower limbs |
| 5 | ABAR | SnaFab | AHV | Left lower limbs |
| 6 | HABA | SnaFab | AHV | Left upper limbs |
| 7 | FASO | SnaFab | AHV | Left lower limbs |
| 8 | RABA | SnaFab | AHV | Right lower limbs |
| 9 | HAHO | SnaFab | AHV | Left lower limbs |
| 10 | REAB | SnaFab | AHV | Left lower limbs |
| 11 | ALSE | SnaFab | MAS | Right upper limbs |
| 12 | BAEB | SnaFab | URM | Left upper limbs |
| 13 | FAMA | SnaFab | AHV | Right upper limbs |
| 14 | ALHA | SnaFab | AHV | Right upper limbs |
| 15 | SASH | SnaFab | AHV | Right lower limbs |
| 16 | EEZ9 | SnaFab | MAS | Left lower limbs |
| 17 | DDH8 | Razi | MAS | Right lower limbs |
| 18 | QQG5 | Razi | MAS | Left lower limbs |
| 19 | XXA1 | SnaFab | MAS | Right upper limbs |
| 20 | SSV7 | Razi | MAS | Right lower limbs |
| 21 | BBM6 | Razi | MAS | Right lower limbs |
| 22 | YYY4 | SnaFab | MAS | Left upper limbs |
| 23 | ZZI1 | SnaFab | URM | Right upper limbs |
| 24 | SSU8 | SnaFab | MAS | Right upper limbs |
| 25 | NNZ3 | Razi | URM | Right lower limbs |
| 26 | FFG3 | SnaFab | AHV | Right upper limbs |
| 27 | IIE0 | Razi | AHV | Left upper limbs |
| 28 | EEG0 | Razi | AHV | Left lower limbs |
| 29 | JJW4 | SnaFab | AHV | Left lower limbs |
| 30 | BBI6 | SnaFab | AHV | Left lower limbs |
| 31 | AAH7 | SnaFab | AHV | Right lower limbs |
| 32 | AAF9 | Razi | AHV | Left lower limbs |
| 33 | WWR1 | Razi | AHV | Left lower limbs |
| 34 | RRS3 | SnaFab | AHV | Left upper limbs |
| 35 | JJI8 | Razi | AHV | Left lower limbs |
| 36 | QQW3 | SnaFab | AHV | Right upper limbs |
| 37 | AAT1 | SnaFab | MAS | Left lower limbs |
| 38 | VVI7 | Razi | AHV | Right lower limbs |
| 39 | UUL5 | SnaFab | AHV | Left lower limbs |
| 40 | QQQ3 | Razi | AHV | Left upper limbs |
| 41 | UUN5 | Razi | AHV | Left upper limbs |
| 42 | XXN0 | SnaFab | AHV | Head & Neck |
| 43 | RRA4 | Razi | AHV | Right upper limbs |
| 44 | DDN5 | SnaFab | AHV | Right lower limbs |
| 45 | OOZ0 | Razi | AHV | Right lower limbs |
| 46 | UUT4 | SnaFab | AHV | Right upper limbs |
| 47 | GGM9 | SnaFab | AHV | Left lower limbs |
| 48 | SSH4 | SnaFab | AHV | Right upper limbs |
| 49 | FFW7 | Razi | AHV | Right lower limbs |
| 50 | MMM9 | Razi | AHV | Right lower limbs |
| 51 | KKZ6 | SnaFab | AHV | Right lower limbs |
| 52 | KKW6 | Razi | AHV | Right lower limbs |
| 53 | EEP5 | Razi | AHV | Right lower limbs |
| 54 | FFR9 | SnaFab | AHV | Right lower limbs |
| 55 | UUN6 | Razi | AHV | Right lower limbs |
| 56 | BBP7 | SnaFab | MAS | Left lower limbs |
| 57 | OOE4 | Razi | AHV | Right lower limbs |
| 58 | QQA5 | SnaFab | AHV | Left lower limbs |
| 59 | FFT8 | Razi | MAS | Right lower limbs |
| 60 | JJY3 | SnaFab | AHV | Left lower limbs |
| 61 | GGN0 | Razi | AHV | Right lower limbs |
| 62 | zzQ0 | SnaFab | AHV | Right lower limbs |
| 63 | JJK9 | Razi | AHV | Left lower limbs |
| 64 | YYE2 | SnaFab | AHV | Right upper limbs |
| 65 | TTJ2 | Razi | MAS | Left upper limbs |
| 66 | GG0 | SnaFab | AHV | Right upper limbs |
| 67 | NN5 | Razi | AHV | Right upper limbs |
| 68 | JX8 | Razi | AHV | Left lower limbs |
| 69 | QU4 | Razi | AHV | Left lower limbs |
| 70 | EEJ8 | Razi | URM | Right lower limbs |
| 71 | WT3 | Razi | AHV | Right upper limbs |
| 72 | DC7 | SnaFab | AHV | Left lower limbs |
| 73 | BK8 | Razi | AHV | Right lower limbs |
| 74 | ZK8 | Razi | AHV | Left upper limbs |
| 75 | FM4 | Razi | AHV | Left upper limbs |
| 76 | YP2 | SnaFab | AHV | Right lower limbs |
| 77 | GK9 | Razi | AHV | Left lower limbs |
| 78 | FC3 | Razi | AHV | Right upper limbs |
| 79 | UT8 | Razi | AHV | Left upper limbs |
| 80 | QS6 | Razi | AHV | Left lower limbs |
| 81 | RL9 | Razi | AHV | Right lower limbs |
| 82 | YF3 | SnaFab | AHV | Right lower limbs |
| 83 | NNK8 | SnaFab | MAS | Right lower limbs |
| 84 | KL5 | Razi | AHV | Left lower limbs |
| 85 | RU5 | SnaFab | AHV | Left lower limbs |
| 86 | OG6 | Razi | AHV | Left lower limbs |
| 87 | RU0 | Razi | AHV | Right upper limbs |
| 88 | CH8 | Razi | AHV | Right lower limbs |
| 89 | VL2 | SnaFab | AHV | Left lower limbs |
| 90 | DC8 | Razi | AHV | Left lower limbs |
| 91 | HV6 | Razi | AHV | Left lower limbs |
| 92 | FL2 | Razi | AHV | Left lower limbs |
| 93 | RS1 | SnaFab | AHV | Left lower limbs |
| 94 | AJ6 | Razi | AHV | Right lower limbs |
| 95 | ZY8 | Razi | AHV | Left lower limbs |
| 96 | YB6 | SnaFab | AHV | Right lower limbs |
| 97 | GS2 | Razi | AHV | Left upper limbs |
| 98 | WZ7 | Razi | AHV | Left lower limbs |

## Table 6 – Vital signs

| Visit 8 | | | | Visit 7 | | | | Visit 6 | | | | Visit 5 | | | | Visit 4 | | | | Visit 2&3 | | | | Visit 1 | | | | Code | ID |
| --- | --- | --- | --- | --- | --- | --- | --- | --- | --- | --- | --- | --- | --- | --- | --- | --- | --- | --- | --- | --- | --- | --- | --- | --- | --- | --- | --- | --- | --- |
| *HR* | ***BP*** | ***RR*** | ***T*** | ***HR*** | ***BP*** | ***RR*** | ***T*** | ***HR*** | ***BP*** | ***RR*** | ***T*** | ***HR*** | ***BP*** | ***RR*** | ***T*** | ***HR*** | ***BP*** | ***RR*** | ***T*** | ***HR*** | ***BP*** | ***RR*** | ***T*** | ***HR*** | ***BP*** | ***RR*** | ***T*** |  |  |
| *82* | ***140/80*** | ***18*** | ***36.7*** | ***88*** | ***134/95*** | ***20*** | ***--*** | ***84*** | ***110/80*** | ***20*** | ***-*** | ***88*** | ***120/80*** | ***20*** | ***-*** | ***80*** | ***130/80*** | ***20*** | ***37*** | ***82*** | ***110/70*** | ***20*** | ***37*** | ***80*** | ***120/80*** | ***18*** | ***36.3*** | **YUAB** | 1 |
| *80* | ***110/70*** | ***20*** | ***36.4*** | ***80*** | ***110/70*** | ***20*** | ***36.4*** | ***82*** | ***110/70*** | ***18*** | ***36*** | ***80*** | ***120/70*** | ***20*** | ***36.8*** | ***83*** | ***110/70*** | ***20*** | ***36.7*** | ***82*** | ***110/70*** | ***20*** | ***36.4*** | ***80*** | ***120/80*** | ***20*** | ***36.8*** | **MAGH** | 2 |
| *DIS* | ***DIS*** | ***DIS*** | ***DIS*** | ***DIS*** | ***DIS*** | ***DIS*** | ***DIS*** | ***DIS*** | ***DIS*** | ***DIS*** | ***DIS*** | ***DIS*** | ***DIS*** | ***DIS*** | ***DIS*** | ***DIS*** | ***DIS*** | ***DIS*** | ***DIS*** | ***82*** | ***120/80*** | ***18*** | ***36.6*** | ***83*** | ***110/75*** | ***20*** | ***36.7*** | **ALSA** | 3 |
| *78* | ***110/70*** | ***18*** | ***36.9*** | ***88*** | ***130/70*** | ***20*** | ***36*** | ***86*** | ***110/60*** | ***20*** | ***37*** | ***80*** | ***120/70*** | ***18*** | ***36*** | ***84*** | ***110/60*** | ***18*** | ***37*** | ***80*** | ***120/80*** | ***18*** | ***36.5*** | ***80*** | ***120/80*** | ***18*** | ***36.4*** | **GHMA** | 4 |
| *DIS* | ***120/70*** | ***DIS*** | ***DIS*** | ***DIS*** | ***DIS*** | ***DIS*** | ***DIS*** | ***DIS*** | ***DIS*** | ***DIS*** | ***DIS*** | ***80*** | ***120/60*** | ***20*** | ***37*** | ***82*** | ***110/70*** | ***20*** | ***36.8*** | ***89*** | ***120/70*** | ***20*** | ***37*** | ***92*** | ***120/75*** | ***19*** | ***36.5*** | **ABAR** | 5 |
| *DIS* | ***120/60*** | ***DIS*** | ***DIS*** | ***DIS*** | ***DIS*** | ***DIS*** | ***DIS*** | ***DIS*** | ***DIS*** | ***DIS*** | ***DIS*** | ***-*** | ***-*** | ***-*** | ***-*** | ***-*** | ***-*** | ***-*** | ***-*** | ***69*** | ***160/90*** | ***20*** | ***36.2*** | ***69*** | ***160/90*** | ***20*** | ***36.2*** | **HABA** | 6 |
| *76* | ***120/77*** | ***20*** | ***37*** | ***80*** | ***110/70*** | ***20*** | ***36.5*** | ***85*** | ***135/85*** | ***18*** | ***36.3*** | ***78*** | ***120/80*** | ***20*** | ***36.5*** | ***80*** | ***100/60*** | ***22*** | ***37*** | ***82*** | ***110/70*** | ***20*** | ***37*** | ***85*** | ***100/60*** | ***18*** | ***36*** | **FASO** | 7 |
| *80* | ***120/60*** | ***20*** | ***37*** | ***72*** | ***120/70*** | ***20*** | ***36.4*** | ***88*** | ***110/70*** | ***20*** | ***37*** | ***78*** | ***110/60*** | ***18*** | ***36*** | ***86*** | ***110/60*** | ***20*** | ***36.5*** | ***82*** | ***110/80*** | ***20*** | ***37*** | ***84*** | ***120/70*** | ***22*** | ***35.9*** | **RABA** | 8 |
| *78* | ***120/77*** | ***18*** | ***36.9*** | ***82*** | ***110/80*** | ***20*** | ***36.5*** | ***78*** | ***120/70*** | ***20*** | ***36.3*** | ***80*** | ***110/80*** | ***20*** | ***36.6*** | ***84*** | ***125/85*** | ***16*** | ***36.5*** | ***85*** | ***111/65*** | ***18*** | ***37*** | ***79*** | ***120/70*** | ***18*** | ***36.2*** | **HAHO** | 9 |
| *DIS* | ***DIS*** | ***DIS*** | ***DIS*** | ***DIS*** | ***DIS*** | ***DIS*** | ***DIS*** | ***DIS*** | ***DIS*** | ***DIS*** | ***DIS*** | ***-*** | ***-*** | ***-*** | ***-*** | ***-*** | ***-*** | ***-*** | ***-*** | ***-*** | ***-*** | ***-*** | ***-*** | ***99*** | ***130/90*** | ***20*** | ***36*** | **REAB** | 10 |
| *DIS* | ***DIS*** | ***DIS*** | ***DIS*** | ***70*** | ***110/80*** | ***18*** | ***36.9*** | ***73*** | ***135/80*** | ***17*** | ***36.9*** | ***70*** | ***130/80*** | ***12*** | ***36.9*** | ***80*** | ***110/70*** | ***14*** | ***36.9*** | ***80*** | ***130/59*** | ***14*** | ***37*** | ***75*** | ***135/55*** | ***13*** | ***36.9*** | **ALSE** | 11 |
| *DIS* | ***DIS*** | ***DIS*** | ***DIS*** | ***80*** | ***110/65*** | ***15*** | ***-*** | ***80*** | ***100/70*** | ***14*** | ***-*** | ***74*** | ***110/70*** | ***14*** | ***-*** | ***74*** | ***110/70*** | ***14*** | ***36*** | ***88*** | ***120/80*** | ***18*** | ***36.5*** | ***88*** | ***110/70*** | ***12*** | ***37*** | **BAEB** | 12 |
| *DIS* | ***DIS*** | ***DIS*** | ***DIS*** | ***82*** | ***120/80*** | ***20*** | ***36.8*** | ***80*** | ***110/60*** | ***20*** | ***37*** | ***70*** | ***110/70*** | ***20*** | ***36.5*** | ***80*** | ***120/60*** | ***20*** | ***36*** | ***76*** | ***120/80*** | ***20*** | ***36.5*** | ***86*** | ***120/80*** | ***20*** | ***36.5*** | **FAMA** | 13 |
| *DIS* | ***DIS*** | ***DIS*** | ***DIS*** | ***DIS*** | ***DIS*** | ***DIS*** | ***DIS*** | ***DIS*** | ***DIS*** | ***DIS*** | ***DIS*** | ***-*** | ***-*** | ***-*** | ***-*** | ***-*** | ***-*** | ***-*** | ***-*** | ***-*** | ***-*** | ***-*** | ***-*** | ***100*** | ***135/80*** | ***20*** | ***36.9*** | **ALHA** | 14 |
| *DIS* | ***DIS*** | ***DIS*** | ***DIS*** | ***DIS*** | ***DIS*** | ***DIS*** | ***DIS*** | ***DIS*** | ***DIS*** | ***DIS*** | ***DIS*** | ***-*** | ***-*** | ***-*** | ***-*** | ***-*** | ***-*** | ***-*** | ***-*** | ***-*** | ***-*** | ***-*** | ***-*** | ***86*** | ***110/70*** | ***16*** | ***36.3*** | **SASH** | 15 |
| *DIS* | ***DIS*** | ***DIS*** | ***DIS*** | ***80*** | ***120/80*** | ***12*** | ***37*** | ***80*** | ***120/80*** | ***14*** | ***37*** | ***80*** | ***120/80*** | ***16*** | ***37*** | ***76*** | ***125/80*** | ***18*** | ***37*** | ***94*** | ***120/79*** | ***12*** | ***37*** | ***108*** | ***120/80*** | ***12*** | ***37.1*** | **EEZ9** | 16 |
| *90* | ***110/70*** | ***16*** | ***37*** | ***88*** | ***105/60*** | ***17*** | ***37*** | ***65*** | ***105/65*** | ***16*** | ***36.7*** | ***86*** | ***110/70*** | ***16*** | ***36.8*** | ***85*** | ***120/80*** | ***17*** | ***37.3*** | ***110*** | ***130/80*** | ***24*** | ***36.4*** | ***100*** | ***130/85*** | ***24*** | ***36.7*** | **DDH8** | 17 |
| *DIS* | ***DIS*** | ***DIS*** | ***DIS*** | ***78*** | ***110/70*** | ***17*** | ***37*** | ***60*** | ***110/60*** | ***16*** | ***37*** | ***85*** | ***110/70*** | ***18*** | ***37*** | ***85*** | ***130/90*** | ***20*** | ***37*** | ***86*** | ***130/80*** | ***28*** | ***36.7*** | ***95*** | ***100/65*** | ***29*** | ***36.5*** | **QQG5** | 18 |
| *DIS* | ***DIS*** | ***DIS*** | ***DIS*** | ***-*** | ***-*** | ***-*** | ***-*** | ***70*** | ***115/70*** | ***16*** | ***36.6*** | ***80*** | ***105/65*** | ***16*** | ***36.5*** | ***82*** | ***110/70*** | ***16*** | ***36.5*** | ***80*** | ***100/70*** | ***15*** | ***37*** | ***83*** | ***100/70*** | ***15*** | ***37*** | **XXA1** | 19 |
| *DIS* | ***DIS*** | ***DIS*** | ***DIS*** | ***-*** | ***-*** | ***-*** | ***-*** | ***-*** | ***-*** | ***-*** | ***-*** | ***82*** | ***130/80*** | ***17*** | ***37*** | ***85*** | ***140/80*** | ***17*** | ***37*** | ***100*** | ***140/90*** | ***17*** | ***37*** | ***100*** | ***140/90*** | ***16*** | ***37*** | **SSV7** | 20 |
| *DIS* | ***DIS*** | ***DIS*** | ***DIS*** | ***DIS*** | ***DIS*** | ***DIS*** | ***DIS*** | ***80*** | ***125/80*** | ***16*** | ***37*** | ***80*** | ***120/70*** | ***14*** | ***37*** | ***-*** | ***-*** | ***-*** | ***-*** | ***70*** | ***120/80*** | ***14*** | ***37*** | ***65*** | ***120/70*** | ***16*** | ***37.3*** | **BBM6** | 21 |
| *DIS* | ***DIS*** | ***DIS*** | ***DIS*** | ***84*** | ***110/70*** | ***18*** | ***37*** | ***70*** | ***110/70*** | ***16*** | ***36.5*** | ***80*** | ***110/70*** | ***16*** | ***37*** | ***86*** | ***120/80*** | ***18*** | ***37*** | ***75*** | ***130/85*** | ***19*** | ***37.2*** | ***78*** | ***120/80*** | ***16*** | ***37.3*** | **YYY4** | 22 |
| *DIS* | ***DIS*** | ***DIS*** | ***DIS*** | ***90*** | ***110/70*** | ***16*** | ***36.8*** | ***80*** | ***100/80*** | ***14*** | ***36.7*** | ***-*** | ***-*** | ***-*** | ***-*** | ***84*** | ***115/75*** | ***20*** | ***36.9*** | ***85*** | ***110/70*** | ***18*** | ***36.8*** | ***77*** | ***100/70*** | ***18*** | ***36.7*** | **ZZI1** | 23 |
| *DIS* | ***DIS*** | ***DIS*** | ***DIS*** | ***DIS*** | ***DIS*** | ***DIS*** | ***DIS*** | ***DIS*** | ***DIS*** | ***DIS*** | ***DIS*** | ***DIS*** | ***DIS*** | ***DIS*** | ***DIS*** | ***-*** | ***-*** | ***-*** | ***-*** | ***-*** | ***-*** | ***-*** | ***-*** | ***63*** | ***90/60*** | ***17*** | ***37*** | **SSU8** | 24 |
| *DIS* | ***DIS*** | ***DIS*** | ***DIS*** | ***DIS*** | ***DIS*** | ***DIS*** | ***DIS*** | ***DIS*** | ***DIS*** | ***DIS*** | ***DIS*** | ***DIS*** | ***DIS*** | ***DIS*** | ***DIS*** | ***56*** | ***100/47*** | ***18*** | ***36.9*** | ***-*** | ***-*** | ***-*** | ***-*** | ***56*** | ***100/60*** | ***17*** | ***37*** | **NNZ3** | 25 |
| *78* | ***120/70*** | ***17*** | ***36.8*** | ***80*** | ***120/70*** | ***18*** | ***37*** | ***80*** | ***120/70*** | ***17*** | ***36.8*** | ***81*** | ***125/80*** | ***18*** | ***36.7*** | ***89*** | ***120/80*** | ***17*** | ***36.7*** | ***81*** | ***130/75*** | ***18*** | ***36.8*** | ***82*** | ***130/80*** | ***18*** | ***37.3*** | **FFG3** | 26 |
| *75* | ***110/80*** | ***19*** | ***37*** | ***78*** | ***120/80*** | ***18*** | ***37*** | ***78*** | ***120/78*** | ***18*** | ***36.5*** | ***81*** | ***120/80*** | ***18*** | ***37*** | ***80*** | ***115/75*** | ***18*** | ***37*** | ***82*** | ***115/75*** | ***19*** | ***36.5*** | ***82*** | ***130/80*** | ***18*** | ***37.5*** | **IIE0** | 27 |
| *78* | ***120/70*** | ***17*** | ***36.8*** | ***90*** | ***110/70*** | ***17*** | ***37*** | ***95*** | ***120/80*** | ***17*** | ***37.3*** | ***90*** | ***110/80*** | ***18*** | ***37.2*** | ***85*** | ***110/80*** | ***20*** | ***37.1*** | ***95*** | ***120/70*** | ***17*** | ***36.9*** | ***90*** | ***110/80*** | ***18*** | ***37*** | **EEG0** | 28 |
| *DIS* | ***DIS*** | ***DIS*** | ***DIS*** | ***82*** | ***120/80*** | ***20*** | ***37*** | ***75*** | ***120/70*** | ***18*** | ***36.3*** | ***86*** | ***110/80*** | ***18*** | ***37*** | ***85*** | ***120/70*** | ***17*** | ***37*** | ***100*** | ***110/80*** | ***18*** | ***37*** | ***95*** | ***120/70*** | ***17*** | ***36.8*** | **JJW4** | 29 |
| *DIS* | ***DIS*** | ***DIS*** | ***DIS*** | ***88*** | ***125/80*** | ***19*** | ***37*** | ***90*** | ***120/90*** | ***20*** | ***37*** | ***78*** | ***110/70*** | ***20*** | ***36.4*** | ***77*** | ***100/60*** | ***18*** | ***364*** | ***78*** | ***115/70*** | ***18*** | ***36.5*** | ***87*** | ***120/80*** | ***17*** | ***37*** | **BBI6** | 30 |
| *88* | ***110/80*** | ***19*** | ***36.5*** | ***88*** | ***100/60*** | ***20*** | ***36.5*** | ***82*** | ***120/70*** | ***20*** | ***36.5*** | ***80*** | ***130/70*** | ***18*** | ***37.2*** | ***86*** | ***110/70*** | ***18*** | ***36.5*** | ***84*** | ***100/70*** | ***22*** | ***37*** | ***83*** | ***110/80*** | ***18*** | ***37*** | **AAH7** | 31 |
| *DIS* | ***DIS*** | ***DIS*** | ***DIS*** | ***DIS*** | ***DIS*** | ***DIS*** | ***DIS*** | ***70*** | ***120/70*** | ***17*** | ***37.1*** | ***90*** | ***110/90*** | ***20*** | ***37*** | ***75*** | ***100/70*** | ***19*** | ***36.8*** | ***87*** | ***120/70*** | ***18*** | ***36.5*** | ***90*** | ***110/80*** | ***20*** | ***37*** | **AAF9** | 32 |
| *DIS* | ***DIS*** | ***DIS*** | ***DIS*** | ***74*** | ***110/70*** | ***18*** | ***37*** | ***75*** | ***120/70*** | ***20*** | ***36.1*** | ***85*** | ***110/60*** | ***17*** | ***37*** | ***95*** | ***120/70*** | ***19*** | ***37*** | ***70*** | ***110/60*** | ***18*** | ***36.8*** | ***90*** | ***100/70*** | ***20*** | ***37*** | **WWR1** | 33 |
| *DIS* | ***DIS*** | ***DIS*** | ***DIS*** | ***DIS*** | ***DIS*** | ***DIS*** | ***DIS*** | ***75*** | ***110/70*** | ***17*** | ***37*** | ***70*** | ***120/70*** | ***18*** | ***37*** | ***78*** | ***130/80*** | ***16*** | ***36.6*** | ***80*** | ***100/60*** | ***16*** | ***36.5*** | ***82*** | ***150/90*** | ***18*** | ***36.8*** | **RRS3** | 34 |
| *70* | ***120/80*** | ***19*** | ***37*** | ***90*** | ***110/80*** | ***19*** | ***37*** | ***78*** | ***110/70*** | ***20*** | ***36.9*** | ***80*** | ***120/90*** | ***17*** | ***37*** | ***79*** | ***120/80*** | ***18*** | ***36.5*** | ***90*** | ***110/80*** | ***18*** | ***37.5*** | ***75*** | ***120/70*** | ***20*** | ***37*** | **JJI8** | 35 |
| *DIS* | ***DIS*** | ***DIS*** | ***DIS*** | ***DIS*** | ***DIS*** | ***DIS*** | ***DIS*** | ***DIS*** | ***DIS*** | ***DIS*** | ***DIS*** | ***73*** | ***120/80*** | ***15*** | ***36.7*** | ***75*** | ***125/85*** | ***18*** | ***36.5*** | ***78*** | ***120/80*** | ***15*** | ***36.3*** | ***80*** | ***120/80*** | ***16*** | ***36.5*** | **QQW3** | 36 |
| *DIS* | ***DIS*** | ***DIS*** | ***DIS*** | ***78*** | ***110/70*** | ***15*** | ***37.5*** | ***76*** | ***100/60*** | ***15*** | ***37.5*** | ***80*** | ***90/60*** | ***15*** | ***37*** | ***80*** | ***100/60*** | ***15*** | ***37.5*** | ***60*** | ***90/60*** | ***16*** | ***37*** | ***88*** | ***80/60*** | ***19*** | ***36.5*** | **AAT1** | 37 |
| *80* | ***120/80*** | ***18*** | ***37*** | ***80*** | ***125/75*** | ***19*** | ***37*** | ***85*** | ***120/80*** | ***18*** | ***37.5*** | ***86*** | ***120/70*** | ***18*** | ***38*** | ***85*** | ***110/80*** | ***19*** | ***37*** | ***87*** | ***120/90*** | ***20*** | ***36*** | ***90*** | ***110/70*** | ***18*** | ***37*** | **VVI7** | 38 |
| *85* | ***120/70*** | ***19*** | ***37*** | ***84*** | ***110/70*** | ***18*** | ***37*** | ***80*** | ***120/70*** | ***20*** | ***37*** | ***85*** | ***110/70*** | ***19*** | ***37*** | ***84*** | ***120/70*** | ***20*** | ***37*** | ***75*** | ***120/75*** | ***20*** | ***37*** | ***98*** | ***120/80*** | ***17*** | ***36.5*** | **UUL5** | 39 |
| *DIS* | ***DIS*** | ***DIS*** | ***DIS*** | ***90*** | ***120/70*** | ***17*** | ***37*** | ***80*** | ***125/85*** | ***18*** | ***36.6*** | ***82*** | ***110/70*** | ***18*** | ***36.7*** | ***82*** | ***125/80*** | ***16*** | ***36.7*** | ***80*** | ***120/80*** | ***16*** | ***36.5*** | ***80*** | ***120/80*** | ***18*** | ***36.5*** | **QQQ3** | 40 |
| *83* | ***120/85*** | ***17*** | ***37.5*** | ***85*** | ***120/80*** | ***17*** | ***37*** | ***90*** | ***130/70*** | ***15*** | ***37*** | ***81*** | ***120/80*** | ***16*** | ***37.2*** | ***80*** | ***110/70*** | ***16*** | ***37*** | ***75*** | ***185/80*** | ***15*** | ***36.7*** | ***77*** | ***120/80*** | ***15*** | ***36.8*** | **UUN5** | 41 |
| *DIS* | ***DIS*** | ***DIS*** | ***DIS*** | ***DIS*** | ***DIS*** | ***DIS*** | ***DIS*** | ***DIS*** | ***DIS*** | ***DIS*** | ***DIS*** | ***85*** | ***110/70*** | ***18*** | ***37*** | ***87*** | ***110/70*** | ***20*** | ***36.8*** | ***90*** | ***130/70*** | ***19*** | ***36.9*** | ***85*** | ***120/70*** | ***18*** | ***37*** | **XXN0** | 42 |
| *DIS* | ***DIS*** | ***DIS*** | ***DIS*** | ***70*** | ***110/70*** | ***20*** | ***36.5*** | ***75*** | ***110/70*** | ***20*** | ***36.8*** | ***80*** | ***125/85*** | ***20*** | ***37*** | ***78*** | ***120/85*** | ***18*** | ***36.5*** | ***78*** | ***125/75*** | ***20*** | ***36.5*** | ***75*** | ***120/80*** | ***19*** | ***37*** | **RRA4** | 43 |
| *DIS* | ***DIS*** | ***DIS*** | ***DIS*** | ***DIS*** | ***DIS*** | ***DIS*** | ***DIS*** | ***DIS*** | ***DIS*** | ***DIS*** | ***DIS*** | ***85*** | ***120/70*** | ***17*** | ***37*** | ***90*** | ***110/70*** | ***20*** | ***38*** | ***85*** | ***120/80*** | ***19*** | ***36.9*** | ***80*** | ***110/70*** | ***18*** | ***37*** | **DDN5** | 44 |
| *DIS* | ***DIS*** | ***DIS*** | ***DIS*** | ***95*** | ***110/70*** | ***19*** | ***37*** | ***95*** | ***110/70*** | ***20*** | ***37.5*** | ***110*** | ***100/60*** | ***18*** | ***37.3*** | ***100*** | ***110/70*** | ***20*** | ***37.2*** | ***84*** | ***110/80*** | ***19*** | ***36.8*** | ***90*** | ***100/60*** | ***18*** | ***37*** | **OOZ0** | 45 |
| *85* | ***110/80*** | ***19*** | ***37*** | ***85*** | ***120/70*** | ***19*** | ***37*** | ***80*** | ***120/70*** | ***19*** | ***37*** | ***80*** | ***110/70*** | ***18*** | ***36.9*** | ***86*** | ***120/80*** | ***18*** | ***37*** | ***83*** | ***110/70*** | ***20*** | ***37*** | ***83*** | ***120/80*** | ***18*** | ***37.1*** | **UUT4** | 46 |
| *70* | ***110/80*** | ***19*** | ***37*** | ***75*** | ***110/70*** | ***19*** | ***37.5*** | ***80*** | ***120/80*** | ***20*** | ***37*** | ***80*** | ***110/80*** | ***19*** | ***37*** | ***90*** | ***120/70*** | ***20*** | ***36.9*** | ***80*** | ***120/80*** | ***18*** | ***37*** | ***85*** | ***120/80*** | ***18*** | ***36*** | **GGM9** | 47 |
| *DIS* | ***DIS*** | ***DIS*** | ***DIS*** | ***88*** | ***110/80*** | ***19*** | ***37*** | ***88*** | ***110/80*** | ***18*** | ***36.9*** | ***85*** | ***120/70*** | ***19*** | ***37*** | ***90*** | ***110/80*** | ***20*** | ***36.5*** | ***80*** | ***120/70*** | ***18*** | ***37*** | ***85*** | ***120/80*** | ***19*** | ***36.5*** | **SSH4** | 48 |
| *70* | ***120/85*** | ***19*** | ***37*** | ***80*** | ***120/80*** | ***17*** | ***36.7*** | ***82*** | ***120/80*** | ***18*** | ***36.6*** | ***78*** | ***115/75*** | ***16*** | ***36.5*** | ***80*** | ***110/70*** | ***18*** | ***36.7*** | ***78*** | ***120/85*** | ***18*** | ***36.5*** | ***68*** | ***120/80*** | ***20*** | ***37*** | **FFW7** | 49 |
| *82* | ***120/80*** | ***18*** | ***36.8*** | ***84*** | ***115/80*** | ***20*** | ***36.9*** | ***82*** | ***115/80*** | ***18*** | ***36.8*** | ***84*** | ***115/80*** | ***18*** | ***37*** | ***88*** | ***120/80*** | ***20*** | ***36.9*** | ***82*** | ***120/80*** | ***18*** | ***37*** | ***84*** | ***120/80*** | ***20*** | ***36.7*** | **MMM9** | 50 |
| *DIS* | ***DIS*** | ***DIS*** | ***DIS*** | ***80*** | ***110/70*** | ***20*** | ***37*** | ***75*** | ***120/80*** | ***18*** | ***37*** | ***75*** | ***120/70*** | ***19*** | ***37*** | ***80*** | ***120/70*** | ***18*** | ***36.9*** | ***85*** | ***130/70*** | ***17*** | ***37*** | ***90*** | ***120/80*** | ***18*** | ***37*** | **KKZ6** | 51 |
| *DIS* | ***DIS*** | ***DIS*** | ***DIS*** | ***DIS*** | ***DIS*** | ***DIS*** | ***DIS*** | ***80*** | ***120/80*** | ***20*** | ***36.5*** | ***70*** | ***110/70*** | ***20*** | ***37*** | ***75*** | ***120/80*** | ***19*** | ***37*** | ***82*** | ***120/75*** | ***19*** | ***36.5*** | ***90*** | ***120/80*** | ***18*** | ***36.5*** | **KKW6** | 52 |
| *DIS* | ***DIS*** | ***DIS*** | ***DIS*** | ***DIS*** | ***DIS*** | ***DIS*** | ***DIS*** | ***DIS*** | ***DIS*** | ***DIS*** | ***DIS*** | ***DIS*** | ***DIS*** | ***DIS*** | ***DIS*** | ***80*** | ***110/80*** | ***17*** | ***36.9*** | ***80*** | ***110/70*** | ***17*** | ***37*** | ***85*** | ***120/70*** | ***18*** | ***37*** | **EEP5** | 53 |
| *DIS* | ***DIS*** | ***DIS*** | ***DIS*** | ***75*** | ***120/80*** | ***19*** | ***37*** | ***85*** | ***120/80*** | ***19*** | ***37*** | ***70*** | ***120/75*** | ***18*** | ***36*** | ***85*** | ***120/75*** | ***20*** | ***37.5*** | ***80*** | ***120/80*** | ***19*** | ***37*** | ***84*** | ***110/70*** | ***17*** | ***36.4*** | **FFR9** | 54 |
| *DIS* | ***DIS*** | ***DIS*** | ***DIS*** | ***101*** | ***120/70*** | ***19*** | ***37.1*** | ***103*** | ***110/70*** | ***18*** | ***37*** | ***105*** | ***110/70*** | ***20*** | ***36.8*** | ***100*** | ***110/80*** | ***18*** | ***37*** | ***89*** | ***100/70*** | ***20*** | ***36.8*** | ***100*** | ***100/70*** | ***19*** | ***36.9*** | **UUN6** | 55 |
| *DIS* | ***DIS*** | ***DIS*** | ***DIS*** | ***DIS*** | ***DIS*** | ***DIS*** | ***DIS*** | ***80*** | ***120/60*** | ***18*** | ***37.5*** | ***80*** | ***110/70*** | ***17*** | ***37*** | ***89*** | ***110/70*** | ***17*** | ***37*** | ***82*** | ***120/75*** | ***16*** | ***37*** | ***88*** | ***110/70*** | ***18*** | ***36.8*** | **BBP7** | 56 |
| *DIS* | ***DIS*** | ***DIS*** | ***DIS*** | ***DIS*** | ***DIS*** | ***DIS*** | ***DIS*** | ***102*** | ***110/75*** | ***19*** | ***37*** | ***103*** | ***110/70*** | ***19*** | ***37.2*** | ***99*** | ***100/60*** | ***23*** | ***37.1*** | ***105*** | ***110/70*** | ***22*** | ***36.9*** | ***95*** | ***120/70*** | ***18*** | ***37*** | **OOE4** | 57 |
| *DIS* | ***DIS*** | ***DIS*** | ***DIS*** | ***DIS*** | ***DIS*** | ***DIS*** | ***DIS*** | ***72*** | ***120/70*** | ***20*** | ***36.5*** | ***75*** | ***120/75*** | ***20*** | ***37.5*** | ***78*** | ***120/80*** | ***19*** | ***37*** | ***75*** | ***115/75*** | ***18*** | ***36.3*** | ***72*** | ***115/75*** | ***18*** | ***36.2*** | **QQA5** | 58 |
| *DIS* | ***DIS*** | ***DIS*** | ***DIS*** | ***81*** | ***100/60*** | ***17*** | ***37.2*** | ***90*** | ***110/70*** | ***16*** | ***37.3*** | ***95*** | ***100/60*** | ***16*** | ***37.5*** | ***100*** | ***100/60*** | ***17*** | ***37.5*** | ***90*** | ***140/100*** | ***19*** | ***37.1*** | ***78*** | ***120/70*** | ***18*** | ***37*** | **FFT8** | 59 |
| *DIS* | ***DIS*** | ***DIS*** | ***DIS*** | ***86*** | ***110/70*** | ***16*** | ***37*** | ***85*** | ***120/70*** | ***17*** | ***36.9*** | ***80*** | ***110/70*** | ***18*** | ***37*** | ***85*** | ***120/70*** | ***17*** | ***37*** | ***90*** | ***120/70*** | ***18*** | ***37*** | ***80*** | ***110/70*** | ***14*** | ***36.6*** | **JJY3** | 60 |
| *DIS* | ***DIS*** | ***DIS*** | ***DIS*** | ***DIS*** | ***DIS*** | ***DIS*** | ***DIS*** | ***75*** | ***125/80*** | ***20*** | ***37*** | ***80*** | ***120/75*** | ***19*** | ***37*** | ***85*** | ***125/80*** | ***20*** | ***36.5*** | ***75*** | ***120/85*** | ***19*** | ***37*** | ***80*** | ***120/80*** | ***14*** | ***36.3*** | **GGN0** | 61 |
| *80* | ***120/75*** | ***17*** | ***37*** | ***87*** | ***120/70*** | ***18*** | ***37*** | ***80*** | ***110/75*** | ***17*** | ***36.7*** | ***100*** | ***110/80*** | ***17*** | ***36.9*** | ***85*** | ***120/80*** | ***16*** | ***36.9*** | ***90*** | ***130/70*** | ***17*** | ***36.8*** | ***95*** | ***120/70*** | ***18*** | ***37*** | **ZZQ0** | 62 |
| *70* | ***120/80*** | ***17*** | ***37*** | ***79*** | ***120/70*** | ***18*** | ***36.8*** | ***81*** | ***120/80*** | ***19*** | ***37*** | ***78*** | ***110/70*** | ***18*** | ***36.9*** | ***90*** | ***120/80*** | ***20*** | ***37*** | ***80*** | ***120/80*** | ***19*** | ***37.2*** | ***85*** | ***110/70*** | ***18*** | ***37.2*** | **JJK9** | 63 |
| *DIS* | ***DIS*** | ***DIS*** | ***DIS*** | ***DIS*** | ***DIS*** | ***DIS*** | ***DIS*** | ***81*** | ***110/70*** | ***18*** | ***37*** | ***80*** | ***120/70*** | ***17*** | ***36.9*** | ***85*** | ***110/70*** | ***19*** | ***37*** | ***80*** | ***120/70*** | ***17*** | ***37*** | ***89*** | ***120/80*** | ***19*** | ***35.5*** | **YYE2** | 64 |
| *DIS* | ***DIS*** | ***DIS*** | ***DIS*** | ***DIS*** | ***DIS*** | ***DIS*** | ***DIS*** | ***DIS*** | ***DIS*** | ***DIS*** | ***DIS*** | ***78*** | ***110/70*** | ***17*** | ***37*** | ***81*** | ***110/70*** | ***17*** | ***37*** | ***80*** | ***110/70*** | ***17*** | ***37*** | ***74*** | ***119/80*** | ***18*** | ***37.4*** | **TTJ2** | 65 |
| *82* | ***120/70*** | ***20*** | ***36.4*** | ***76*** | ***110/70*** | ***20*** | ***36.6*** | ***76*** | ***110/60*** | ***18*** | ***36.6*** | ***75*** | ***100/60*** | ***18*** | ***36*** | ***80*** | ***110/60*** | ***20*** | ***36.5*** | ***75*** | ***110/70*** | ***18*** | ***36.5*** | ***70*** | ***120/70*** | ***20*** | ***36.5*** | **GG0** | 66 |
| *DIS* | ***DIS*** | ***DIS*** | ***DIS*** | ***95*** | ***110/75*** | ***19*** | ***37.1*** | ***90*** | ***120/70*** | ***17*** | ***36.9*** | ***89*** | ***120/75*** | ***18*** | ***37.3*** | ***84*** | ***130/70*** | ***20*** | ***37.2*** | ***80*** | ***120/80*** | ***17*** | ***36.9*** | ***90*** | ***110/70*** | ***18*** | ***37*** | **NN5** | 67 |
| *DIS* | ***DIS*** | ***DIS*** | ***DIS*** | ***DIS*** | ***DIS*** | ***DIS*** | ***DIS*** | ***77*** | ***130/70*** | ***18*** | ***36.5*** | ***76*** | ***100/60*** | ***20*** | ***36.5*** | ***80*** | ***125/85*** | ***18*** | ***36.5*** | ***85*** | ***120/90*** | ***18*** | ***36.8*** | ***75*** | ***120/70*** | ***17*** | ***37.4*** | **JX8** | 68 |
| *DIS* | ***DIS*** | ***DIS*** | ***DIS*** | ***DIS*** | ***DIS*** | ***DIS*** | ***DIS*** | ***DIS*** | ***DIS*** | ***DIS*** | ***DIS*** | ***80*** | ***120/70*** | ***19*** | ***37*** | ***85*** | ***120/80*** | ***18*** | ***36.8*** | ***90*** | ***110/80*** | ***17*** | ***37*** | ***80*** | ***120/70*** | ***18*** | ***37*** | **QU4** | 69 |
| *76* | ***100/70*** | ***14*** | ***36.5*** | ***72*** | ***100/70*** | ***14*** | ***36*** | ***92*** | ***110/70*** | ***16*** | ***36.5*** | ***96*** | ***110/75*** | ***16*** | ***36.5*** | ***92*** | ***110/70*** | ***18*** | ***37*** | ***96*** | ***110/75*** | ***18*** | ***37.2*** | ***104*** | ***125/75*** | ***18*** | ***37.2*** | **EEJ8** | 70 |
| *88* | ***110/80*** | ***18*** | ***36*** | ***70*** | ***110/80*** | ***18*** | ***37*** | ***70*** | ***110/70*** | ***13*** | ***36.9*** | ***75*** | ***120/80*** | ***17*** | ***37*** | ***80*** | ***110/70*** | ***18*** | ***37*** | ***81*** | ***110/80*** | ***17*** | ***36.9*** | ***93*** | ***120/70*** | ***18*** | ***37*** | **WT3** | 71 |
| *DIS* | ***DIS*** | ***DIS*** | ***DIS*** | ***DIS*** | ***DIS*** | ***DIS*** | ***DIS*** | ***DIS*** | ***DIS*** | ***DIS*** | ***DIS*** | ***DIS*** | ***DIS*** | ***DIS*** | ***DIS*** | ***90*** | ***120/70*** | ***18*** | ***37.2*** | ***85*** | ***110/80*** | ***17*** | ***37.1*** | ***90*** | ***120/70*** | ***18*** | ***37*** | **DC7** | 72 |
| *78* | ***110/78*** | ***17*** | ***36.5*** | ***76*** | ***110/80*** | ***17*** | ***37*** | ***75*** | ***120/70*** | ***16*** | ***37*** | ***80*** | ***120/80*** | ***18*** | ***36.8*** | ***75*** | ***120/70*** | ***17*** | ***37*** | ***74*** | ***110/70*** | ***16*** | ***36.8*** | ***75*** | ***110/60*** | ***18*** | ***36.7*** | **BK8** | 73 |
| *82* | ***120/80*** | ***18*** | ***37*** | ***80*** | ***110/80*** | ***17*** | ***37.1*** | ***87*** | ***120/70*** | ***16*** | ***37*** | ***80*** | ***120/80*** | ***17*** | ***37.1*** | ***85*** | ***120/70*** | ***18*** | ***37.1*** | ***82*** | ***120/80*** | ***16*** | ***37*** | ***80*** | ***120/70*** | ***17*** | ***37*** | **ZK8** | 74 |
| *DIS* | ***DIS*** | ***DIS*** | ***DIS*** | ***DIS*** | ***DIS*** | ***DIS*** | ***DIS*** | ***85*** | ***120/78*** | ***19*** | ***37*** | ***83*** | ***120/80*** | ***15*** | ***36.8*** | ***90*** | ***120/70*** | ***20*** | ***37*** | ***80*** | ***120/80*** | ***17*** | ***37*** | ***85*** | ***120/70*** | ***18*** | ***36.8*** | **FM4** | 75 |
| *DIS* | ***DIS*** | ***DIS*** | ***DIS*** | ***75*** | ***120/70*** | ***16*** | ***37*** | ***78*** | ***120/70*** | ***16*** | ***37.2*** | ***80*** | ***120/70*** | ***16*** | ***37.1*** | ***78*** | ***120/90*** | ***15*** | ***37*** | ***85*** | ***120/80*** | ***17*** | ***36.9*** | ***90*** | ***110/70*** | ***18*** | ***37*** | **YP2** | 76 |
| *DIS* | ***DIS*** | ***DIS*** | ***DIS*** | ***DIS*** | ***DIS*** | ***DIS*** | ***DIS*** | ***DIS*** | ***DIS*** | ***DIS*** | ***DIS*** | ***DIS*** | ***DIS*** | ***DIS*** | ***DIS*** | ***83*** | ***110/70*** | ***17*** | ***36.5*** | ***85*** | ***120/70*** | ***19*** | ***36.9*** | ***85*** | ***120/80*** | ***18*** | ***37*** | **GK9** | 77 |
| *DIS* | ***DIS*** | ***DIS*** | ***DIS*** | ***80*** | ***120/85*** | ***17*** | ***37*** | ***72*** | ***120/80*** | ***18*** | ***37*** | ***72*** | ***120/80*** | ***17*** | ***36*** | ***80*** | ***110/70*** | ***18*** | ***37*** | ***80*** | ***120/70*** | ***19*** | ***36*** | ***85*** | ***120/80*** | ***18*** | ***37*** | **FC3** | 78 |
| *70* | ***110/70*** | ***18*** | ***36.5*** | ***75*** | ***120/75*** | ***19*** | ***37*** | ***75*** | ***120/70*** | ***19*** | ***36.5*** | ***78*** | ***120/70*** | ***19*** | ***36.5*** | ***75*** | ***120/80*** | ***19*** | ***37*** | ***80*** | ***120/75*** | ***18*** | ***36.5*** | ***75*** | ***120/75*** | ***20*** | ***37*** | **UT8** | 79 |
| *85* | ***120/70*** | ***18*** | ***37*** | ***85*** | ***120/70*** | ***18*** | ***37*** | ***95*** | ***110/90*** | ***18*** | ***37.1*** | ***90*** | ***120/80*** | ***17*** | ***37*** | ***85*** | ***120/70*** | ***17*** | ***37*** | ***95*** | ***120/80*** | ***20*** | ***36*** | ***90*** | ***110/70*** | ***18*** | ***37*** | QS6 | 80 |
| *DIS* | *DIS* | *DIS* | *DIS* | ***75*** | ***120/80*** | ***15*** | ***37*** | ***75*** | ***120/80*** | ***15*** | ***37.6*** | ***75*** | ***110/80*** | ***14*** | ***36.8*** | ***76*** | ***120/80*** | ***16*** | ***37.2*** | ***75*** | ***120/80*** | ***16*** | ***37.2*** | ***75*** | ***120/80*** | ***14*** | ***37.6*** | RL9 | 81 |
| *DIS* | *DIS* | *DIS* | *DIS* | *DIS* | *DIS* | *DIS* | *DIS* | ***84*** | ***110/70*** | ***16*** | ***37.2*** | ***86*** | ***120/80*** | ***16*** | ***37.6*** | ***85*** | ***120/80*** | ***16*** | ***37.2*** | ***85*** | ***110/80*** | ***18*** | ***37.6*** | ***86*** | ***110/80*** | ***18*** | ***37.2*** | YF3 | 82 |
| *DIS* | *DIS* | *DIS* | *DIS* | *DIS* | *DIS* | *DIS* | *DIS* | *DIS* | *DIS* | *DIS* | *DIS* | ***85*** | ***115/70*** | ***15*** | ***37*** | ***88*** | ***115/70*** | ***15*** | ***37*** | ***88*** | ***110/70*** | ***15*** | ***37*** | ***78*** | ***120/80*** | ***17*** | ***36.8*** | NNK8 | 83 |
| *DIS* | *DIS* | *DIS* | *DIS* | *DIS* | *DIS* | *DIS* | *DIS* | ***78*** | ***120/80*** | ***16*** | ***36.6*** | ***75*** | ***110/70*** | ***14*** | ***37.2*** | ***75*** | ***110/80*** | ***14*** | ***36.8*** | ***80*** | ***120/80*** | ***16*** | ***37.2*** | ***75*** | ***120/80*** | ***16*** | ***37.2*** | KL5 | 84 |
| *DIS* | *DIS* | *DIS* | *DIS* | *DIS* | *DIS* | *DIS* | *DIS* | ***75*** | ***120/70*** | ***16*** | ***37.2*** | ***75*** | ***120/80*** | ***16*** | ***36.8*** | ***72*** | ***120/80*** | ***15*** | ***36.8*** | ***75*** | ***130/80*** | ***16*** | ***36.2*** | ***76*** | ***130/80*** | ***14*** | ***36.2*** | RU5 | 85 |
| *DIS* | *DIS* | *DIS* | *DIS* | *DIS* | *DIS* | *DIS* | *DIS* | ***85*** | ***120/80*** | ***15*** | ***36.8*** | ***80*** | ***110/80*** | ***16*** | ***37.2*** | ***80*** | ***110/80*** | ***16*** | ***37.2*** | ***85*** | ***120/80*** | ***18*** | ***36.2*** | ***86*** | ***120/80*** | ***18*** | ***36.8*** | OG6 | 86 |
| *DIS* | *DIS* | *DIS* | *DIS* | ***82*** | ***110/75*** | ***16*** | ***36.2*** | ***80*** | ***120/70*** | ***16*** | ***36.2*** | ***78*** | ***120/80*** | ***16*** | ***37.2*** | ***75*** | ***120/80*** | ***18*** | ***36.8*** | ***80*** | ***130/80*** | ***18*** | ***37*** | ***75*** | ***130/80*** | ***16*** | ***37.2*** | RU0 | 87 |
| *DIS* | *DIS* | *DIS* | *DIS* | ***95*** | ***110/80*** | ***19*** | ***37.1*** | ***90*** | ***100/70*** | ***17*** | ***37*** | ***85*** | ***110/80*** | ***20*** | ***37*** | ***90*** | ***100/70*** | ***22*** | ***37*** | ***89*** | ***110/80*** | ***21*** | ***36.5*** | ***83*** | ***100/70*** | ***19*** | ***37*** | CH8 | 88 |
| *DIS* | *DIS* | *DIS* | *DIS* | *DIS* | *DIS* | *DIS* | *DIS* | ***80*** | ***120/80*** | ***15*** | ***36.8*** | ***88*** | ***120/80*** | ***15*** | ***37.2*** | ***85*** | ***120/80*** | ***16*** | ***37.2*** | ***80*** | ***130/80*** | ***16*** | ***37.6*** | ***80*** | ***130/80*** | ***18*** | ***37.2*** | VL2 | 89 |
| *84* | ***120/80*** | ***18*** | ***36.8*** | ***83*** | ***120/80*** | ***18*** | ***36.8*** | ***79*** | ***110/80*** | ***18*** | ***37*** | ***80*** | ***110/70*** | ***18*** | ***37*** | ***85*** | ***110/70*** | ***18*** | ***36.9*** | ***70*** | ***110/80*** | ***17*** | ***37*** | ***100*** | ***110/70*** | ***18*** | ***37*** | DC8 | 90 |
| *DIS* | *DIS* | *DIS* | *DIS* | ***80*** | ***130/80*** | ***18*** | ***37.2*** | ***82*** | ***130/80*** | ***18*** | ***37.2*** | ***80*** | ***120/70*** | ***16*** | ***37.6*** | ***86*** | ***130/70*** | ***16*** | ***37.2*** | ***85*** | ***130/80*** | ***18*** | ***37.6*** | ***86*** | ***130/80*** | ***18*** | ***37.6*** | HV6 | 91 |
| *DIS* | *DIS* | *DIS* | *DIS* | *DIS* | *DIS* | *DIS* | *DIS* | ***75*** | ***130/70*** | ***16*** | ***37.2*** | ***75*** | ***120/80*** | ***18*** | ***37.2*** | ***72*** | ***120/80*** | ***16*** | ***36.8*** | ***75*** | ***130/80*** | ***18*** | ***37.6*** | ***75*** | ***130/80*** | ***18*** | ***37.2*** | FL2 | 92 |
| *DIS* | *DIS* | *DIS* | *DIS* | *DIS* | *DIS* | *DIS* | *DIS* | *DIS* | *DIS* | *DIS* | *DIS* | *DIS* | *DIS* | *DIS* | *DIS* | *DIS* | *DIS* | *DIS* | *DIS* | ***85*** | ***120/80*** | ***17*** | ***37.6*** | ***86*** | ***130/80*** | ***18*** | ***37.2*** | RS1 | 93 |
| *DIS* | *DIS* | *DIS* | *DIS* | *DIS* | *DIS* | *DIS* | *DIS* | ***78*** | ***125/75*** | ***16*** | ***37.2*** | ***86*** | ***120/75*** | ***18*** | ***36.8*** | ***75*** | ***130/80*** | ***17*** | ***37.2*** | ***85*** | ***130/80*** | ***16*** | ***36.2*** | ***86*** | ***130/80*** | ***18*** | ***36.8*** | AJ6 | 94 |
| *DIS* | *DIS* | *DIS* | *DIS* | ***86*** | ***120/80*** | ***16*** | ***37*** | ***86*** | ***120/80*** | ***16*** | ***36.6*** | ***85*** | ***110/80*** | ***17*** | ***36.8*** | ***86*** | ***120/80*** | ***16*** | ***36.8*** | ***88*** | ***120/80*** | ***18*** | ***36.2*** | ***86*** | ***130/80*** | ***18*** | ***36.8*** | ZY8 | 95 |
| *DIS* | *DIS* | *DIS* | *DIS* | *DIS* | *DIS* | *DIS* | *DIS* | *DIS* | *DIS* | *DIS* | *DIS* | ***75*** | ***120/80*** | ***15*** | ***37.1*** | ***80*** | ***130/80*** | ***16*** | ***37.1*** | ***77*** | ***130/80*** | ***15*** | ***36.7*** | ***78*** | ***130/80*** | ***15*** | ***36.6*** | YB6 | 96 |
| *DIS* | *DIS* | *DIS* | *DIS* | *DIS* | *DIS* | *DIS* | *DIS* | ***84*** | ***125/75*** | ***14*** | ***37*** | ***82*** | ***120/75*** | ***16*** | ***36.7*** | ***80*** | ***120/70*** | ***15*** | ***37.1*** | ***85*** | ***130/80*** | ***16*** | ***36.8*** | ***82*** | ***120/70*** | ***16*** | ***37*** | GS2 | 97 |
| *DIS* | ***DIS*** | ***DIS*** | ***DIS*** | ***DIS*** | ***DIS*** | ***DIS*** | ***DIS*** | ***DIS*** | ***DIS*** | ***DIS*** | ***DIS*** | ***88*** | ***130/80*** | ***15*** | ***37*** | ***86*** | ***130/70*** | ***15*** | ***36.9*** | ***84*** | ***120/70*** | ***13*** | ***37.2*** | ***86*** | ***120/70*** | ***14*** | ***37.1*** | WZ7 | 98 |

T: Temperature, RR: Respiratory Rate, BP: Blood pressure, HR: Heart Rate, DIS: Discharged

## Table 7 – Sign and symptoms, severity, and number of antivenoms administered

| ID | Code | Group | Center | Local 1 | Local 2 | Local 3 | Local 4 | Local 5 | Systemic 1 | Systemic 2 | Systemic 3 | Systemic 4 | Severity | Total AV used |
| --- | --- | --- | --- | --- | --- | --- | --- | --- | --- | --- | --- | --- | --- | --- |
| 1 | YUAB | SnaFab | AHV | Swelling>2.5 cm | - | - | - | - | Severe active bleeding | Coagulation test abnormality | - | - | Severe | 20 |
| 2 | MAGH | SnaFab | AHV | Significant pain | Swelling>2.5 cm | - | - | - | - | - | - | - | Moderate | 5 |
| 3 | ALSA | SnaFab | AHV | Significant pain | Swelling>2.5 cm | - | - | - | - | - | - | - | Moderate | 5 |
| 4 | GHMA | SnaFab | AHV | Significant pain | Swelling>2.5 cm | Compartment Syndrome | - | - | Coagulation test abnormality | - | - | - | Severe | 15 |
| 5 | ABAR | SnaFab | AHV | Pregressive swelling | - | - | - | - | Coagulation test abnormality | - | - | - | Moderate | 5 |
| 6 | HABA | SnaFab | AHV | Significant pain | - | - | - | - | - | - | - | - | Moderate | 5 |
| 7 | FASO | SnaFab | AHV | Significant pain | Pregressive swelling | - | - | - | - | - | - | - | Moderate | 7 |
| 8 | RABA | SnaFab | AHV | Significant pain | Pregressive swelling | - | - | - | - | - | - | - | Moderate | 9 |
| 9 | HAHO | SnaFab | AHV | Significant pain | complete swelling of each finger | - | - | - | Thrombocytopenia | - | - | - | Moderate | 5 |
| 10 | REAB | SnaFab | AHV | Significant pain | Swelling>2.5 cm | - | - | - | Coagulation test abnormality | - | - | - | Moderate | 5 |
| 11 | ALSE | SnaFab | MAS | Pregressive swelling | - | - | - | - | - | - | - | - | Moderate | 21 |
| 12 | BAEB | SnaFab | URM | Significant pain | Swelling>2.5 cm | - | - | - | - | - | - | - | Moderate | 11 |
| 13 | FAMA | SnaFab | AHV | Pregressive swelling | Swelling>2.5 cm | - | - | - | - | - | - | - | Moderate | 5 |
| 14 | ALHA | SnaFab | AHV | Swelling>2.5 cm | - | - | - | - | - | - | - | - | Moderate | 5 |
| 15 | SASH | SnaFab | AHV | Swelling>2.5 cm | - | - | - | - | Coagulation test abnormality | - | - | - | Moderate | 10 |
| 16 | EEZ9 | SnaFab | MAS | Swelling>2.5 cm | Pregressive swelling | Significant pain | Blister formation | Tissue destruction (Skin or muscle necrosis) | Coagulation test abnormality | Thrombocytopenia | - | - | Moderate | 16 |
| 17 | DDH8 | Razi | MAS | Swelling>2.5 cm | Pregressive swelling | Significant pain | Blister formation | - | Generalized weakness | Spontaneous bleeding | Refractry vomitting | - | Moderate | 16 |
| 18 | QQG5 | Razi | MAS | Any severity pain | Any severity swelling | - | - | - | - | - | - | - | Moderate | 11 |
| 19 | XXA1 | SnaFab | MAS | Any severity pain | Any severity swelling | Blister formation | - | - | - | - | - | - | Moderate | 11 |
| 20 | SSV7 | Razi | MAS | Any severity pain | Any severity swelling | - | - | - | - | - | - | - | Moderate | 11 |
| 21 | BBM6 | Razi | MAS | Any severity pain | Any severity swelling | - | - | - | Coagulation test abnormality |  |  | - | Moderate | 11 |
| 22 | YYY4 | SnaFab | MAS | Any severity pain | Any severity swelling | - | - | - | - | - | - | - | Moderate | 11 |
| 23 | ZZI1 | SnaFab | URM | Significant pain | Pregressive swelling | Swelling>2.5 cm | Blister formation | - | Thrombocytopenia | - | - | - | Moderate | 11 |
| 24 | SSU8 | SnaFab | MAS | Any severity pain | Any severity swelling | - | - | - | - | - | - | - | Moderate | 11 |
| 25 | NNZ3 | Razi | URM | Significant pain | Pregressive swelling | Swelling>2.5 cm | - | - | Thrombocytopenia | - | - | - | Moderate | 6 |
| 26 | FFG3 | SnaFab | AHV | Significant pain | Swelling>2.5 cm | - | - | - | Coagulation test abnormality | - | - | - | Moderate | 15 |
| 27 | IIE0 | Razi | AHV | Significant pain | complete swelling of each finger | - | - | - | Coagulation test abnormality | - | - | - | Moderate | 15 |
| 28 | EEG0 | Razi | AHV | Significant pain | complete swelling of each finger | - | - | - | Coagulation test abnormality | - | - | - | Moderate | 10 |
| 29 | JJW4 | SnaFab | AHV | Significant pain | Swelling>2.5 cm | - | - | - | - | - | - | - | Moderate | 5 |
| 30 | BBI6 | SnaFab | AHV | Significant pain | Swelling>2.5 cm | - | - | - | Coagulation test abnormality | - | - | - | Moderate | 15 |
| 31 | AAH7 | SnaFab | AHV | Significant pain | - | - | - | - | Coagulation test abnormality | Generalized weakness | - | - | Moderate | 5 |
| 32 | AAF9 | Razi | AHV | Significant pain | Swelling>2.5 cm | - | - | - | Coagulation test abnormality | - | - | - | Moderate | 5 |
| 33 | WWR1 | Razi | AHV | Significant pain | Swelling>2.5 cm | - | - | - | Thrombocytopenia | - | - | - | Moderate | 5 |
| 34 | RRS3 | SnaFab | AHV | Significant pain | Swelling>2.5 cm | - | - | - | - | - | - | - | Moderate | 5 |
| 35 | JJI8 | Razi | AHV | Significant pain | Swelling>2.5 cm | - | - | - | Coagulation test abnormality | - | - | - | Moderate | 5 |
| 36 | QQW3 | SnaFab | AHV | Significant pain | Swelling>2.5 cm | - | - | - | Coagulation test abnormality | - | - | - | Moderate | 5 |
| 37 | AAT1 | SnaFab | MAS | Significant pain | Swelling>2.5 cm | - | - | - | - | - | - | - | Moderate | 16 |
| 38 | VVI7 | Razi | AHV | Significant pain | Swelling>2.5 cm | - | - | - | Coagulation test abnormality | - | - | - | Moderate | 10 |
| 39 | UUL5 | SnaFab | AHV | Significant pain | Swelling>2.5 cm | - | - | - | - | - | - | - | Moderate | 15 |
| 40 | QQQ3 | Razi | AHV | Significant pain | Swelling>2.5 cm | - | - | - | Coagulation test abnormality | Generalized weakness | - | - | Moderate | 10 |
| 41 | UUN5 | Razi | AHV | Significant pain | Swelling>2.5 cm | - | - | - | Coagulation test abnormality | Generalized weakness | - | - | Moderate | 10 |
| 42 | XXN0 | SnaFab | AHV | Significant pain | Head & neck bite | - | - | - | - | - | - | - | Moderate | 5 |
| 43 | RRA4 | Razi | AHV | Significant pain | Swelling>2.5 cm | - | - | - | - | - | - | - | Moderate | 5 |
| 44 | DDN5 | SnaFab | AHV | Significant pain | Swelling>2.5 cm | - | - | - | Generalized weakness | Refractry vomitting | Severe active bleeding | - | Severe | 10 |
| 45 | OOZ0 | Razi | AHV | Significant pain | Swelling>2.5 cm | - | - | - | Coagulation test abnormality | - | - | - | Moderate | 5 |
| 46 | UUT4 | SnaFab | AHV | Significant pain | complete swelling of each finger | - | - | - | Coagulation test abnormality | Spontaneous bleeding | - | - | Moderate | 10 |
| 47 | GGM9 | SnaFab | AHV | Significant pain | Swelling>2.5 cm | - | - | - | Generalized weakness | - | - | - | Moderate | 5 |
| 48 | SSH4 | SnaFab | AHV | complete swelling of each finger | Swelling>2.5 cm | - | - | - | Coagulation test abnormality | - | - | - | Moderate | 10 |
| 49 | FFW7 | Razi | AHV | Significant pain | Swelling>2.5 cm | - | - | - | Spontaneous bleeding | Generalized weakness | Thrombocytopenia | - | Moderate | 5 |
| 50 | MMM9 | Razi | AHV | Significant pain | Swelling>2.5 cm | - | - | - | Severe active bleeding | Coagulation test abnormality | - | - | Severe | 10 |
| 51 | KKZ6 | SnaFab | AHV | Significant pain | Swelling>2.5 cm | - | - | - | - | - | - | - | Moderate | 5 |
| 52 | KKW6 | Razi | AHV | Significant pain | Swelling>2.5 cm | - | - | - | Coagulation test abnormality | - | - | - | Moderate | 10 |
| 53 | EEP5 | Razi | AHV | Significant pain | Swelling>2.5 cm | - | - | - | - | - | - | - | Moderate | 5 |
| 54 | FFR9 | SnaFab | AHV | Significant pain | Swelling>2.5 cm | - | - | - | Coagulation test abnormality | Spontaneous bleeding | Refractry vomitting | - | Moderate | 10 |
| 55 | UUN6 | Razi | AHV | Significant pain | Swelling>2.5 cm | - | - | - | Coagulation test abnormality | - | - | - | Moderate | 10 |
| 56 | BBP7 | SnaFab | MAS | Significant pain | Swelling>2.5 cm | - | - | - | - | - | - | - | Moderate | 11 |
| 57 | OOE4 | Razi | AHV | Significant pain | - | - | - | - | Generalized weakness | - | - | - | Moderate | 5 |
| 58 | QQA5 | SnaFab | AHV | Significant pain | Swelling>2.5 cm | - | - | - | Coagulation test abnormality | Generalized weakness | Thrombocytopenia | Refractry vomitting | Moderate | 10 |
| 59 | FFT8 | Razi | MAS | Any severity pain | Any severity swelling | - | - | - | Thrombocytopenia | - | - | - | Moderate | 21 |
| 60 | JJY3 | SnaFab | AHV | Significant pain | Swelling>2.5 cm | - | - | - | Generalized weakness | - | - | - | Moderate | 5 |
| 61 | GGN0 | Razi | AHV | Significant pain | Swelling>2.5 cm | - | - | - | - | - | - | - | Moderate | 5 |
| 62 | ZZQ0 | SnaFab | AHV | Significant pain | Swelling>2.5 cm | - | - | - | Thrombocytopenia | Coagulation test abnormality | - | - | Moderate | 15 |
| 63 | JJK9 | Razi | AHV | Significant pain | Swelling>2.5 cm | - | - | - | Spontaneous bleeding | - | - | - | Severe | 15 |
| 64 | YYE2 | SnaFab | AHV | Significant pain | Swelling>2.5 cm | - | - | - | Generalized weakness | - | - | - | Moderate | 5 |
| 65 | TTJ2 | Razi | MAS | Significant pain | Swelling>2.5 cm | - | - | - | - | - | - | - | Moderate | 11 |
| 66 | GG0 | SnaFab | AHV | Swelling>2.5 cm | - | - | - | - | Coagulation test abnormality | - | - | - | Moderate | 10 |
| 67 | NN5 | Razi | AHV | Significant pain | Swelling>2.5 cm | - | - | - | Coagulation test abnormality | - | - | - | Moderate | 15 |
| 68 | JX8 | Razi | AHV | Swelling>2.5 cm | - | - | - | - | - | - | - | - | Moderate | 5 |
| 69 | QU4 | Razi | AHV | Swelling>2.5 cm | - | - | - | - | Coagulation test abnormality | - | - | - | Moderate | 10 |
| 70 | EEJ8 | Razi | URM | Significant pain | Swelling>2.5 cm | - | - | - | - | - | - | - | Moderate | 16 |
| 71 | WT3 | Razi | AHV | Significant pain | Swelling>2.5 cm | - | - | - | Coagulation test abnormality | - | - | - | Moderate | 5 |
| 72 | DC7 | SnaFab | AHV | Significant pain | Swelling>2.5 cm | - | - | - | - | - | - | - | Moderate | 5 |
| 73 | BK8 | Razi | AHV | Significant pain | Swelling>2.5 cm | - | - | - | - | - | - | - | Moderate | 10 |
| 74 | ZK8 | Razi | AHV | Significant pain | Swelling>2.5 cm | - | - | - | Coagulation test abnormality | - | - | - | Moderate | 20 |
| 75 | FM4 | Razi | AHV | Significant pain | Swelling>2.5 cm | - | - | - | Coagulation test abnormality | - | - | - | Moderate | 15 |
| 76 | YP2 | SnaFab | AHV | Significant pain | Swelling>2.5 cm | - | - | - | Coagulation test abnormality | - | - | - | Moderate | 10 |
| 77 | GK9 | Razi | AHV | Swelling>2.5 cm | - | - | - | - | - | - | - | - | Moderate | 5 |
| 78 | FC3 | Razi | AHV | Significant pain | Swelling>2.5 cm | - | - | - | - | - | - | - | Moderate | 5 |
| 79 | UT8 | Razi | AHV | Significant pain | Swelling>2.5 cm | - | - | - | Coagulation test abnormality | - | - | - | Moderate | 5 |
| 80 | QS6 | Razi | AHV | Significant pain | Swelling>2.5 cm | - | - | - | Coagulation test abnormality | - | - | - | Moderate | 10 |
| 81 | RL9 | Razi | AHV | Significant pain | Swelling>2.5 cm | - | - | - | Coagulation test abnormality | - | - | - | Moderate | 5 |
| 82 | YF3 | SnaFab | AHV | Significant pain | Swelling>2.5 cm | - | - | - | Coagulation test abnormality | - | - | - | Moderate | 10 |
| 83 | NNK8 | SnaFab | MAS | Significant pain | Swelling>2.5 cm | - | - | - | - | - | - | - | Moderate | 9 |
| 84 | KL5 | Razi | AHV | Significant pain | Swelling>2.5 cm | - | - | - | - | - | - | - | Moderate | 5 |
| 85 | RU5 | SnaFab | AHV | Significant pain | Swelling>2.5 cm | - | - | - | Thrombocytopenia | - | - | - | Moderate | 5 |
| 86 | OG6 | Razi | AHV | Significant pain | Swelling>2.5 cm | - | - | - | - | - | - | - | Moderate | 5 |
| 87 | RU0 | Razi | AHV | Significant pain | Swelling>2.5 cm | - | - | - | Coagulation test abnormality | - | - | - | Moderate | 15 |
| 88 | CH8 | Razi | AHV | Significant pain | Swelling>2.5 cm | - | - | - | Coagulation test abnormality | - | - | - | Moderate | 5 |
| 89 | VL2 | SnaFab | AHV | Significant pain | Swelling>2.5 cm | - | - | - | - | - | - | - | Moderate | 5 |
| 90 | DC8 | Razi | AHV | Significant pain | Swelling>2.5 cm | - | - | - | Coagulation test abnormality | - | - | - | Moderate | 5 |
| 91 | HV6 | Razi | AHV | Significant pain | Swelling>2.5 cm | - | - | - | Coagulation test abnormality | - | - | - | Moderate | 5 |
| 92 | FL2 | Razi | AHV | Significant pain | Swelling>2.5 cm | - | - | - | Generalized weakness | Thrombocytopenia | - | - | Moderate | 10 |
| 93 | RS1 | SnaFab | AHV | - | - | - | - | - | Coagulation test abnormality | - | - | - | Moderate | 5 |
| 94 | AJ6 | Razi | AHV | Significant pain | Swelling>2.5 cm | - | - | - | Generalized weakness | Vomitting | - | - | Moderate | 5 |
| 95 | ZY8 | Razi | AHV | Significant pain | Swelling>2.5 cm | - | - | - | - | - | - | - | Moderate | 5 |
| 96 | YB6 | SnaFab | AHV | Significant pain | Swelling>2.5 cm | - | - | - | - | - | - | - | Moderate | 5 |
| 97 | GS2 | Razi | AHV | Significant pain | Swelling>2.5 cm | - | - | - | Vomitting | Generalized pain | - | - | Moderate | 10 |
| 98 | WZ7 | Razi | AHV | Significant pain | Swelling>2.5 cm | - | - | - | Vomitting | Coagulation test abnormality | - | - | Moderate | 15 |

## Table 8 – Baseline lab results

| ID | Code | Group | Center | PT | PTT | INR | WBC | RBC | Hb | HCT | PLT | BUN/ Cr | Na/ K | AST/  ALT/LDH | BIL total/ direct | ECG |
| --- | --- | --- | --- | --- | --- | --- | --- | --- | --- | --- | --- | --- | --- | --- | --- | --- |
| 1 | **YUAB** | SnaFab | AHV | 36* | 47 | 7* | 10.2 | 5.29 | 16.4 | 46.7 | 208 | 19/1 | 137/4 | 42/30/- | 1/- | N |
| 2 | **MAGH** | SnaFab | AHV | - | - | - | - | - | - | - | - | - | - | - | - | - |
| 3 | **ALSA** | SnaFab | AHV | - | - | - | - | - | - | - | - | - | - | - | - | - |
| 4 | **GHMA** | SnaFab | AHV | 36* | 120 | 6* | 13.7 | 4.75 | 13.9 | 38.6 | 261 | 31/1.6 | 135/3.7 | - | 1/- | - |
| 5 | **ABAR** | SnaFab | AHV | 23.7 | 37 | 3.5 | 8.1 | 5.63 | 15.6 | 45.7 | 185 | 23/0.7 | 138/3.3 | - | - | N |
| 6 | **HABA** | SnaFab | AHV | - | - | - | - | - | - | - | - | - | - | - | - | - |
| 7 | **FASO** | SnaFab | AHV | - | - | - | - | - | - | - | - | - | - | - | - | N |
| 8 | **RABA** | SnaFab | AHV | 13.7 | 47 | 1.2 | 7.3 | 5.27 | 16.6 | 47 | 291 | 12/0.5 | 140/3.8 | 30/28 | 1.5/- | N |
| 9 | **HAHO** | SnaFab | AHV | 13.3 | 37 | 1.13 | 10 | 5.89 | 16.2 | 47.8 | 101 | 20/1 | 145/4.2 | - | - | - |
| 10 | **REAB** | SnaFab | AHV | 36 | 121 | 6 | 11.6 | 5.36 | 15.7 | 47.1 | 193 | 23/1.1 | 136/3.9 | 24/45 | - | N |
| 11 | **ALSE** | SnaFab | MAS | 13.3 | 28.5 | 1.13 | 13.6 | 4.97 | 14.5 | 41.6 | 143 | 22.8/0.7 | 141/4 | 16/14/395 | 1.2/0.2 | N |
| 12 | **BAEB** | SnaFab | URM | 12 | 28 | 0.88 | 10.1 | 5.62 | 17.1 | 50.5 | 199 | 15.4/0.9 | 137/4 | - | - | N |
| 13 | **FAMA** | SnaFab | AHV | 15 | 25 | 1.4 | 7.6 | 4.64 | 9.7 | 30.3 | 266 | 17/1 | 136/3.6 | **-** | 0.8/- | N |
| 14 | **ALHA** | SnaFab | AHV | 14.7 | 37 | 1.3 | 7.5 | 4.24 | 10.8 | 33.1 | 247 | - | - | **-** | **-/**1 | N |
| 15 | **SASH** | SnaFab | AHV | 36* | 54 | 6* | 6.5 | 5.06 | 14.5 | 40.7 | 193 | 13/0.8 | 136/4.3 | -/27/- | 0.5/- | N |
| 16 | **EEZ9** | SnaFab | MAS | 15.8 | 45.4 | 1.4 | 13.4 | 5.56 | 16.1 | 46.9 | 96 | 19/1 | 140/4.6 | 21/21/298 | 0.8/0.5 | N |
| 17 | **DDH8** | Razi | MAS | 13.8 | 29.9 | 1.25 | 13 | 4 | 12.3 | 35.8 | 218 | 20.4/1 | 141/3.6 | 16/11/283 | 0.5/0.1 | تاکیکاردی سینوسی |
| 18 | **QQG5** | Razi | MAS | 13 | 29 | 1.14 | 8.6 | 4.79 | 13.8 | 35.1 | 206 | 7/0.7 | 140/3.7 | 24/14/474 | 0.4/0.2 | N |
| 19 | **XXA1** | SnaFab | MAS | 13.4 | 37.2 | 1.19 | 8.6 | 4.47 | 12.4 | 35.1 | 222 | 11.2/0.6 | 139/4.3 | 18/9/347 | 0.5/0.1 | N |
| 20 | **SSV7** | Razi | MAS | 11.9 | 30 | 0.99 | 20 | 5.27 | 16 | 45 | 237 | 27/1.1 | 142/3.8 | 34/44/- | 0.8/0.3 | N |
| 21 | **BBM6** | Razi | MAS | 20.4 | 31.1 | 2.35 | 14 | 4.92 | 15 | 42.2 | 159 | 15.8/0.9 | 137/3.4 | 19/14/397 | 1.2/0.3 | N |
| 22 | **YYY4** | SnaFab | MAS | 33.2 | 28.9 | 1 | 10.6 | 5.53 | 16.2 | 49.9 | 143 | 18.3/0.7 | 138/4 | 20/18/435 | 0.6/0.2 | N |
| 23 | **ZZI1** | SnaFab | URM | 13.5 | 28 | 1.06 | 14.5 | 5.09 | 15.8 | - | 107 | 31/1 | 136/3.9 | - | - | N |
| 24 | **SSU8** | SnaFab | MAS | 13.3 | 29.9 | 1.18 | 9.3 | 4.39 | 13.2 | 39.1 | 181 | 7.5/0.7 | 139/4.2 | 17/11/427 | 0.3/01 | N |
| 25 | **NNZ3** | Razi | URM | 13 | 34.5 | 1 | 11.4 | 5.34 | 17.7 | - | 128 | 16/1.2 | 141/3.94 | 43/29.3/482 | - | N |
| 26 | **FFG3** | SnaFab | AHV | 108 | 121* | 11.4 | 8.61 | 5.13 | 15.6 | 46.3 | 222 | 12/1 | 139/3.6 | - | - | N |
| 27 | **IIE0** | Razi | AHV | 36* | 120* | 6* | 11.76 | 5.42 | 16.5 | 48 | 264 | 14/0.7 | 145/4 | - | - | N |
| 28 | **EEG0** | Razi | AHV | 36* | 121* | 6* | 11.7 | 5.97 | 17 | 50.2 | 222 | 21/1.4 | 134/4 | - | - | N |
| 29 | **JJW4** | SnaFab | AHV | 14 | 25 | 1.2 | 10.92 | 7.12 | 14.4 | 47 | 165 | 17/1.1 | 140/3.2 | - | - | N |
| 30 | **BBI6** | SnaFab | AHV | 36* | 121* | - | 9.54 | 5.03 | 13.3 | 39.8 | 177 | - | - | - | - | N |
| 31 | **AAH7** | SnaFab | AHV | 36* | 120* | 6* | 8.2 | 5.74 | 15.6 | 48.9 | 234 | 21/1.2 | 145/4.1 | - | - | N |
| 32 | **AAF9** | Razi | AHV | 21 | 32 | 2.5 | 7.8 | 6.13 | 12.4 | 38.8 | 193 | 12/0.8 | 135/3.6 | - | - | N |
| 33 | **WWR1** | Razi | AHV | 13 | 25 | 1.1 | 6.28 | 4.75 | 13.6 | 40.8 | 110 | 23/1.1 | 135/3.8 | - | - | N |
| 34 | **RRS3** | SnaFab | AHV | 12.4 | 29 | 1.04 | 7.6 | 4.47 | 12.6 | 38.3 | 336 | 16/0.7 | 142/3.9 | 35/40/- | 1.2/0.2 | N |
| 35 | **JJI8** | Razi | AHV | 37* | 41 | 6.1 | 16.3 | 5.89 | 12 | 38.2 | 358 | 19/1 | 136/5 | - | - | N |
| 36 | **QQW3** | SnaFab | AHV | 36* | 121* | 6* | 6.8 | 5.08 | 13.4 | 41.7 | 313 | 11/0.9 | 145/3.8 | - | 0.6/- | N |
| 37 | **AAT1** | SnaFab | MAS | 12 | 29 | 1 | 5.8 | 5.7 | 12.9 | 42.9 | 228 | 11.2/0.7 | 136/4.8 | 12/14/239 | 0.5/0.1 | N |
| 38 | **VVI7** | Razi | AHV | * | * | * | 22.37 | 6.03 | 17.4 | 53.6 | 236 | 13/1 | 139/4 | 35/-/- | 1.5/0.8 | N |
| 39 | **UUL5** | SnaFab | AHV | 14 | 121 | 1.2 | 8.5 | 6.32 | 18.9 | 55.1 | 237 | 12/1.4 | 142/3.4 | - | - | N |
| 40 | **QQQ3** | Razi | AHV | 18 | 32 | 1.8 | 10.7 | 6.03 | 16.4 | 50.7 | 304 | 16/1.4 | 140/3.1 | - | 1.1/- | N |
| 41 | **UUN5** | Razi | AHV | 19 | 37 | 2.1 | 12.5 | 5.63 | 14.9 | 46.8 | 262 | 17/1.2 | 138/4.6 | - | - | N |
| 42 | **XXN0** | SnaFab | AHV | 12 | 25 | 1 | 8.6 | 5.17 | 14.6 | 44.1 | 246 | 12/1.2 | 146/4.2 | - | - | N |
| 43 | **RRA4** | Razi | AHV | 13 | 25 | 1.1 | 4.8 | 5.09 | 12.8 | 42 | 279 | 19/0.8 | 143/4.2 | - | - | N |
| 44 | **DDN5** | SnaFab | AHV | 12 | 26 | 1 | 8.3 | 4.76 | 15 | 42.8 | 205 | 14/1.1 | 140/4.1 | - | - | N |
| 45 | **OOZ0** | Razi | AHV | 37* | 121* | 6* | 10.6 | 5.1 | 14.5 | 45 | 181 | 16/1 | 140/3.5 | - | - | N |
| 46 | **UUT4** | SnaFab | AHV | 36* | 121* | -* | 18.01 | 5.48 | 16.4 | 48.1 | 223 | 17/1 | 141/4 | 37/-/566 | 2/0.2 | N |
| 47 | **GGM9** | SnaFab | AHV | 13 | 25 | 1.1 | 8.3 | 4.93 | 13.4 | 39.7 | 260 | -/1.1 | 140/4.2 | - | 0.5/- | N |
| 48 | **SSH4** | SnaFab | AHV | 16 | 40 | 1.6 | - | - | - | - | - | -/1.3 | 136/4 | - | - | N |
| 49 | **FFW7** | Razi | AHV | * | * | * | 5.3 | 5.43 | 15.6 | 47.9 | 103 | 19/1.3 | 139/3.7 | - | - | N |
| 50 | **MMM9** | Razi | AHV | 37 | 50 | 6.1 | 27.7 | 5.33 | 15.3 | 47.2 | 217 | 22/1.3 | 142/3.3 | -/-/683 | 1.9 | N |
| 51 | **KKZ6** | SnaFab | AHV | 13 | 25 | 1.1 | 15.4 | 5.34 | 15.5 | 46.9 | 313 | 13/1 | 138/3.7 | - | - | N |
| 52 | **KKW6** | Razi | AHV | 14 | 121 | 1.3 | - | - | - | - | - | 19/1.5 | 136/3.5 | - | - | N |
| 53 | **EEP5** | Razi | AHV | 12 | 24 | 1 | 6.16 | 4.93 | 15.6 | 45.8 | 190 | 12/1 | 136/3.4 | - | - | N |
| 54 | **FFR9** | SnaFab | AHV | 16 | 30 | 1.6 | 8 | 5.76 | 16.7 | 47.4 | 246 | 12/1 | 136/3.9 | - | - | N |
| 55 | **UUN6** | Razi | AHV | - | - | - | - | - | - | - | - | - | - | - | - | N |
| 56 | **BBP7** | SnaFab | MAS | 11 | 30 | 0.9 | 15.3 | 4.57 | 14.2 | 42.4 | 182 | 32/0.8 | 139/4.1 | 24/46/319 | 0.4/0.1 | N |
| 57 | **OOE4** | Razi | AHV | 12 | 32 | 1 | 8.4 | 4.42 | 12.2 | 35.5 | 197 | 8/0.7 | 140/3.6 | - | - | N |
| 58 | **QQA5** | SnaFab | AHV | 36* | 121* | 6* | 5.1 | 6.79 | 19.8 | 60.1 | 60* | 13/1.2 | 140/3.8 | 23/-/1028 | 3.6/1.7 | N |
| 59 | **FFT8** | Razi | MAS | 12 | 23 | 1 | 16.7 | 5.52 | 15.5 | 42.5 | 34 | 13.8/1.11 | 142/3.4 | 23/12/473 | 0.7/0.2 | N |
| 60 | **JJY3** | SnaFab | AHV | 13 | 25 | 1.1 | 5.7 | 5.92 | 17.6 | 50.4 | 301 | 12/1.1 | 139/4.3 | - | - | N |
| 61 | **GGN0** | Razi | AHV | 12 | 24 | 1 | 5.02 | 4.21 | 13 | 38.9 | 287 | 13/0.7 | 136/4.1 | 20/-/- | 1.6/0.2 | N |
| 62 | **ZZQ0** | SnaFab | AHV | 36 | 121 | 6 | 9.2 | 5.94 | 16.7 | 48 | 129 | 10/0.8 | 137/4.4 | -/-/394 | 1.5/- | N |
| 63 | **JJK9** | Razi | AHV | - | - | - | - | - | - | - | - | - | - | - | - | N |
| 64 | **YYE2** | SnaFab | AHV | 12 | 25 | 1 | 9.3 | 4.5 | 13.5 | 38.5 | 323 | 12/1 | 137/3.8 | - | - | N |
| 65 | **TTJ2** | Razi | MAS | 11.4 | 47 | 1.04 | 9.6 | 5.1 | 15.7 | 45.2 | 262 | 16.8/0.9 | 140/4.1 | 18/9/290 | 2.3/0.5 | N |
| 66 | **GG0** | SnaFab | AHV | 37 | 121 | 6 | 15.9 | 4.54 | 13 | 39.1 | 361 | 13/0.8 | 138/3.8 | - | 0.8/- | N |
| 67 | **NN5** | Razi | AHV | 36 | 32 | - | 6.8 | 6.58 | 13.4 | 41.8 | 190 | 15/0.7 | 140/3.6 | 20/-/- | 2.7/0.4 | N |
| 68 | **JX8** | Razi | AHV | 14 | 34 | 1.3 | 8.8 | 4.96 | 14.5 | 43.8 | 234 | 17/1 | 139/3.9 | - | 0.5/- | N |
| 69 | **QU4** | Razi | AHV | 37 | 121 | 6 | 10.8 | 6.05 | 17.3 | 49.6 | 259 | 35/1.1 | 144/4.1 | - | - | N |
| 70 | **EEJ8** | Razi | URM | 13 | 40 | 1 | 11.9 | - | 15.2 | - | 350 | 36.5/1.2 | 140/3.8 | 14/16/306 | 0.9/0.3 | N |
| 71 | **WT3** | Razi | AHV | 38 | 120 | 6 | 14.3 | 4.11 | 11.6 | 34.8 | 215 | 19/0.8 | 137/2.8 | - | - | N |
| 72 | **DC7** | SnaFab | AHV | 12 | 27 | 1 | 10.5 | 5.07 | 15.9 | 45.1 | 219 | 12/0.7 | 141/4.3 | 46/63/- | 1.2/0.3 | N |
| 73 | **BK8** | Razi | AHV | 13 | 38 | 1.1 | 11.1 | 5.97 | 17.6 | 50.6 | 180 | -/0.9 | 138/3.4 | - | 1.6/0.3 | N |
| 74 | **ZK8** | Razi | AHV | 36 | 121 | 6 | 9.5 | 4.46 | 11.4 | 33.8 | 219 | 14/0.8 | 143/4.5 | **-** | - | N |
| 75 | **FM4** | Razi | AHV | 36 | 120 | 6 | 11.5 | 5.24 | 14.6 | 40 | 279 | 9/1.2 | 136/3.9 | **-** | **-** | N |
| 76 | **YP2** | SnaFab | AHV | 37 | 121 | 6 | 10.3 | 5.68 | 17.9 | 48.2 | 221 | 9/1.4 | 137/4.1 | - | - | N |
| 77 | **GK9** | Razi | AHV | 12 | 35 | 1 | 11.1 | 5.06 | 13.2 | 38.5 | 259 | 12/0.9 | 139/3.9 | - | - | N |
| 78 | **FC3** | Razi | AHV | 12 | 25 | 1 | 11.9 | 4.87 | 12.5 | 36 | 264 | 10/1 | 147/4.4 | - | - | N |
| 79 | **UT8** | Razi | AHV | 60 | 120 | 6 | 11.3 | 4.93 | 14.6 | 44.7 | 283 | 6/0.9 | 135/3.7 | - | - | N |
| 80 | **QS6** | Razi | AHV | 37* | 121* | 6* | 7.8 | 4.34 | 12.2 | 36.8 | 290 | 26/0.9 | 144/3.9 | -/-/780 | - | N |
| 81 | **RL9** | Razi | AHV | 17 | 120 | 1.8 | - | - | - | - | - | 26/1.1 | - | - | - | N |
| 82 | **YF3** | SnaFab | AHV | 15 | 121 | 1.4 | 13.1 | 4.93 | 16 | 46 | 266 | 24/1.3 | 136/3.2 | - | - | N |
| 83 | **NNK8** | SnaFab | MAS | - | - | - | 12.7 | 4.78 | 15.2 | 42.6 | 174 | 38/0.9 | 140/4.4 | 34/24/347 | 0.6/0.2 | N |
| 84 | **KL5** | Razi | AHV | 13.1 | 27 | 1.16 | 9.5 | 4.61 | 13.4 | 38.7 | 357 | 17/.09 | 140/3.8 | - | - | N |
| 85 | **RU5** | SnaFab | AHV | 14 | 25 | 1.2 | 9.3 | 5.15 | 15.3 | 44.8 | 122 | 24/1 | 147/3.1 | - | - | N |
| 86 | **OG6** | Razi | AHV | 14 | 30 | 1.2 | 8.5 | 5.07 | 17.5 | 50.9 | 238 | 18/1 | 141/3.4 | -/14/408 | 1.1/- | N |
| 87 | **RU0** | Razi | AHV | 12 | 121 | 1.6 | 11.9 | 4.45 | 13.2 | 40 | 205 | 20/1 | 143/3.6 | -/20/- | 1.3/- | N |
| 88 | **CH8** | Razi | AHV | 23 | 28 | 2.4 | 5.5 | 4.48 | 11.7 | 35.3 | 337 | 15/0.8 | 144/3.4 | 36/27/- | - | N |
| 89 | **VL2** | SnaFab | AHV | 12 | 31 | 1 | 7.2 | 4.95 | 14.3 | 44.1 | 330 | 10/1 | 142/3.3 | -/10/390 | 0.5/- | N |
| 90 | **DC8** | Razi | AHV | 60 | 120 | 6 | 8.9 | 5.28 | 13.8 | 43 | 228 | 5/0.6 | 143/3.7 | - | - | N |
| 91 | HV6 | Razi | AHV | 15 | 35 | 1.4 | 12.1 | 4.39 | 14.9 | 45.8 | 245 | 21/1.1 | 140/4 | -/35/- | 0.8/- | N |
| 92 | FL2 | Razi | AHV | 13 | 28 | 1 | 12.1 | 4.97 | 15.7 | 45.5 | 71 | 24/1 | 142/4.2 | - | - | N |
| 93 | RS1 | SnaFab | AHV | 36 | 121 | 6 | 8.4 | 4.68 | 12.7 | 39.8 | 309 | 15/0.9 | 143/4 | - | - | N |
| 94 | AJ6 | Razi | AHV | 12 | 26 | 1 | 7.8 | 4.89 | 14.4 | 43.8 | 177 | 19/0.7 | 144/3.9 | - | - | N |
| 95 | ZY8 | Razi | AHV | 13 | 31 | 1.1 | 14.3 | 4.25 | 13 | 40.5 | 314 | 21/0.7 | 139/3.7 | - | 0.5/- | N |
| 96 | YB6 | SnaFab | AHV | 12 | 25 | 1 | 9.8 | 4.02 | 12.6 | 38.4 | 224 | 16/0.5 | 143/3.3 | - | 0.4/- | N |
| 97 | GS2 | Razi | AHV | 12.9 | 30 | 1.13 | 7.2 | 4.51 | 13.8 | 42.7 | 155 | 12/0.6 | 148/3.9 | - | - | N |
| 98 | WZ7 | Razi | AHV | 37* | 121* | 6 | 7.7 | 4.11 | 12.7 | 35.5 | 270 | 13/0.7 | 141/3.3 | -/-/454 | 0.7/- | N |

N= normal, * = result disrupted

## Table 9 - Test results of victims 6 hour post-administration

| ID | Code | Group | Center | PT | PTT | INR | WBC | RBC | Hb | HCT | PLT | BUN/ Cr | Na/ K | CPK | AST/  ALT/LDH | BIL total/ direct | ECG |
| --- | --- | --- | --- | --- | --- | --- | --- | --- | --- | --- | --- | --- | --- | --- | --- | --- | --- |
| 1 | **YUAB** | SnaFab | AHV | 16 | 37 | 1.6 | - | - | - | - | - | - | - | - | - | - | N |
| 2 | **MAGH** | SnaFab | AHV | - | - | - | - | - | - | - | - | - | - | - | - | - | - |
| 3 | **ALSA** | SnaFab | AHV | DIS | DIS | DIS | DIS | DIS | DIS | DIS | DIS | DIS | DIS | DIS | DIS | DIS | DIS |
| 4 | **GHMA** | SnaFab | AHV | 25.1 | 64.6 | 3.9 | 10.7 | 4.48 | 13.3 | 39.4 | 225 | - | - | - | - | - | N |
| 5 | **ABAR** | SnaFab | AHV | - | - | - | - | - | - | - | - | - | - | - | - | - | - |
| 6 | **HABA** | SnaFab | AHV | - | - | - | - | - | - | - | - | - | - | - | - | - | - |
| 7 | **FASO** | SnaFab | AHV | 18 | 55 | 2.05 | 13 | 4.93 | 14.8 | 44.5 | 183 | - | - | - | - | - | - |
| 8 | **RABA** | SnaFab | AHV | 20.4 | 55 | 2.62 | 9.6 | 5.14 | 16.2 | 46.1 | 256 | - | - | - | - | - | - |
| 9 | **HAHO** | SnaFab | AHV | 14.7 | 39 | 1.38 | - | - | - | - | - | - | - | - | - | - | N |
| 10 | **REAB** | SnaFab | AHV | - | - | - | 10.9 | 4.85 | 14.5 | 44.2 | 163 | - | - | - | - | - | - |
| 11 | **ALSE** | SnaFab | MAS | 15 | 30 | 1.31 | 10.6 | 4.25 | 12.6 | 35.7 | 221 | 21.4/0.7 | 138/4.1 | - | - | - | N |
| 12 | **BAEB** | SnaFab | URM | - | - | - | - | - | - | - | - | - | - | - | - | - | - |
| 13 | **FAMA** | SnaFab | AHV | 18 | 37 | 1.84 | - | - | - | - | - | - | - | **-** | **-** | - | - |
| 14 | **ALHA** | SnaFab | AHV | - | - | - | 13.5 | 4.25 | 10.2 | 32.5 | 236 | - | - | **-** | **-** | **-** | - |
| 15 | **SASH** | SnaFab | AHV | 17.2 | 31 | 1.5 | - | - | - | - | - | - | - | - | - | - | - |
| 16 | **EEZ9** | SnaFab | MAS | 16.1 | 27.1 | 1.43 | 13.5 | 4.78 | 14.1 | 40.7 | 82 | 17.9/0.9 | 140/4.2 | - | - | - | N |
| 17 | **DDH8** | Razi | MAS | 13.2 | 27 | 1.17 | 11.2 | 3.74 | 11.3 | 33.5 | 193 | 12.5/1 | 144/3.5 | 43 | - | - | N |
| 18 | **QQG5** | Razi | MAS | 12.9 | 30 | 1.12 | 7.1 | 4.32 | 12.5 | 35.9 | 180 | 6.25/0.7 | 139/3.2 | - | - | - | N |
| 19 | **XXA1** | SnaFab | MAS | 13.4 | 38.9 | 1.19 | 9.4 | 4.51 | 12.7 | 35.8 | 233 | 10/0.6 | 137/4.6 | - | - | - | N |
| 20 | **SSV7** | Razi | MAS | 11 | 24.2 | 0.83 | 21.8 | 5.3 | 15.4 | 45 | 152 | 29/0.9 | - | - | - | - | N |
| 21 | **BBM6** | Razi | MAS | 14.3 | 34 | 1.33 | 10 | 5.04 | 14.6 | 44.6 | 166 | 12.9/1.1 | 148/4.4 | - | - | - | N |
| 22 | **YYY4** | SnaFab | MAS | 12.9 | 28 | 1.12 | 8.6 | 5 | 14.4 | 45.8 | 188 | 17.5/0.8 | 137/4.2 | - | - | - | N |
| 23 | **ZZI1** | SnaFab | URM | - | - | - | - | - | - | - | - | - | - | - | - | - | N |
| 24 | **SSU8** | SnaFab | MAS | 13.1 | 32 | 1.15 | 7 | 4.67 | 14 | 40.6 | 189 | 8.3/0.9 | 141/4 | 112 | - | - | N |
| 25 | **NNZ3** | Razi | URM | 13 | 38 | 1 | 20 | 6.46 | 20.8 | - | 295 | 15/1.17 | 140/5.4 | - | - | - | N |
| 26 | **FFG3** | SnaFab | AHV | 37* | 42 | 6* | 12.7 | 4.94 | 14.6 | 45 | 227 | - | - | - | - | - | N |
| 27 | **IIE0** | Razi | AHV | 36* | 120* | 6* | 13.9 | 5.1 | 15 | 45.9 | 229 | - | - | - | - | - | N |
| 28 | **EEG0** | Razi | AHV | 26 | 36 | 3.4 | 4.97 | 3.65 | 9.8 | 30 | 125 | 20/1.6 | 135/4.1 | - | - | - | N |
| 29 | **JJW4** | SnaFab | AHV | 14.8 | 25 | 1.27 | 12 | 6.4 | 14.1 | 43.7 | 198 | - | - | - | - | - | N |
| 30 | **BBI6** | SnaFab | AHV | 37 | 66 | 7 | 13.5 | 5.2 | 13.2 | 41.7 | 186 | 8/1 | 141/4 | - | - | - | N |
| 31 | **AAH7** | SnaFab | AHV | 12 | 121* | 1 | 11.56 | 5.65 | 15.8 | 47.6 | 238 | - | - | - | - | - | N |
| 32 | **AAF9** | Razi | AHV | 16 | 30 | 1.5 | - | - | - | - | - | - | - | - | - | - | N |
| 33 | **WWR1** | Razi | AHV | 13 | 30 | 1.1 | 16.7 | 5.2 | 14.4 | 45.6 | 259 | - | - | - | - | - | N |
| 34 | **RRS3** | SnaFab | AHV | 18 | 25 | 1.9 | 16.8 | 5.39 | 14.7 | 46.5 | 386 | 16/1.2 | 134/3.6 | - | - | - | N |
| 35 | **JJI8** | Razi | AHV | 15 | 31 | 1.4 | - | - | - | - | - | 10/0.8 | 139/4.3 | - | - | - | N |
| 36 | **QQW3** | SnaFab | AHV | 12 | 25 | 1 | 8.3 | 4.8 | 12.7 | 40.6 | 265 | - | - | - | - | - | N |
| 37 | **AAT1** | SnaFab | MAS | 11.5 | 27 | 0.93 | 6.7 | 4.5 | 10.1 | 33.5 | 141 | 9.5/0.7 | 137/4 |  | - | - | N |
| 38 | **VVI7** | Razi | AHV | 16 | 33 | 1.6 | 24.1 | 6.85 | 20.1 | 61.7 | 247 | - | - | - | - | - | N |
| 39 | **UUL5** | SnaFab | AHV | 37 | 121 | 6.1 | 14.4 | 5.92 | 16.8 | 52.7 | 206 | - | - | - | - | - | N |
| 40 | **QQQ3** | Razi | AHV | 21 | 36 | 2.5 | 11.2 | 5.82 | 15.7 | 49.2 | 285 | - | - | - | - | - | N |
| 41 | **UUN5** | Razi | AHV | - | - | - | - | - | - | - | - | - | - | - | - | - | N |
| 42 | **XXN0** | SnaFab | AHV | - | - | - | - | - | - | - | - | 12/1.1 | 141/3.7 | **-** | - | 0.8/- | N |
| 43 | **RRA4** | Razi | AHV | 14 | 37 | 1.3 | - | - | - | - | - | - | - | - | - | - | N |
| 44 | **DDN5** | SnaFab | AHV | - | - | - | - | - | - | - | - | - | - | - | - | - | N |
| 45 | **OOZ0** | Razi | AHV | * | * | * | 8 | 4.15 | 11.4 | 36.2 | 166 | - | - | - | - | - | N |
| 46 | **UUT4** | SnaFab | AHV | 37 | 121 | - | 13.5 | 5.57 | 15.4 | 45.5 | 211 | - | - | - | - | - | N |
| 47 | **GGM9** | SnaFab | AHV | 14 | 32 | 1.3 | - | - | - | - | - | -/1 | 136/4 | - | - | - | N |
| 48 | **SSH4** | SnaFab | AHV | 37* | 55* | 7* | 9.7 | 5.13 | 16.2 | 47.3 | 212 | -/1.5 | 133/4.4 | - | - | - | N |
| 49 | **FFW7** | Razi | AHV | 14 | 28 | 1.3 | - | - | - | - | - | - | - | - | - | - | N |
| 50 | **MMM9** | Razi | AHV | 16 | 34 | 1.6 | 16.9 | 5.27 | 15.5 | 45.7 | 196 | 22/1.5 | 140/4.4 | - | - | 0.5 | N |
| 51 | **KKZ6** | SnaFab | AHV | 13 | 25 | 1.1 | - | - | - | - | - | - | - | - | - | - | N |
| 52 | **KKW6** | Razi | AHV | 25 | 50 | 3.3 | - | - | - | - | - | - | - | - | - | - | N |
| 53 | **EEP5** | Razi | AHV | - | - | - | - | - | - | - | - | - | - | - | - | - | N |
| 54 | **FFR9** | SnaFab | AHV | - | - | - | - | - | - | - | - | - | - | - | - | - | N |
| 55 | **UUN6** | Razi | AHV | 60* | 120* | 6* | 8.5 | 4 | 11.1 | 33.2 | 321 | 9/0.5 | 140/3.9 | - | - | - | N |
| 56 | **BBP7** | SnaFab | MAS | - | - | - | - | - | - | - | - | - | - | - | - | - | N |
| 57 | **OOE4** | Razi | AHV | 13 | 32 | 1 | - | - | - | - | - | - | - | - | - | - | N |
| 58 | **QQA5** | SnaFab | AHV | 36 | 121 | * | 22.1 | 6.32 | 18.9 | 55.9 | 60* | 17/- | - | - | - | - | N |
| 59 | **FFT8** | Razi | MAS | 17 | 25.5 | 1.75 | 5.4 | 4.47 | 12.6 | 37.4 | 119 | 13/1 | 138/4.3 | - | - | - | N |
| 60 | **JJY3** | SnaFab | AHV | 12 | 50 | 1 | 11.2 | 5.64 | 16.6 | 48.4 | 297 | - | - | - | - | - | N |
| 61 | **GGN0** | Razi | AHV | - | - | - | - | - | - | - | - | - | - | - | - | - | N |
| 62 | **ZZQ0** | SnaFab | AHV | 36 | 65 | 6 | 15.1 | 6.17 | 17.7 | 50.3 | 167 | - | - | - | - | - | N |
| 63 | **JJK9** | Razi | AHV | 12 | 43 | 1 | 10.4 | 4.46 | 13.4 | 40.4 | 200 | 20/1.3 | 138/3.7 | - | - | - | N |
| 64 | **YYE2** | SnaFab | AHV | - | - | - | - | - | - | - | - | - | - | - | - | - | N |
| 65 | **TTJ2** | Razi | MAS | 12.7 | 44 | 1.15 | 9.5 | 4.82 | 14.8 | 43.2 | 253 | 14.4/0.9 | 142/3.4 | - | - | - | N |
| 66 | **GG0** | SnaFab | AHV | 36* | 121* | 7* | 18.4 | 4.53 | 13 | 39.4 | 314 | - | - | - | - | - | N |
| 67 | **NN5** | Razi | AHV | - | - | - | 11.3 | 6.23 | 12.5 | 38.7 | 189 | - | - | - | - | - | N |
| 68 | **JX8** | Razi | AHV | 12 | 24 | 1 | - | - | - | - | - | 13/0.9 | 140/4.3 | - | - | - | N |
| 69 | **QU4** | Razi | AHV | 14.9 | 46 | 1.28 | 15.61 | 5.74 | 16.9 | 50.6 | 234 | - | - | - | - | - | N |
| 70 | **EEJ8** | Razi | URM | 13 | 42 | 1.2 | 12.4 | - | 15.8 | - | 280 | 32/1 | 144/4 | 262 | - | - | N |
| 71 | **WT3** | Razi | AHV | 13 | 25 | 1 | 7 | 3.64 | 10.2 | 31.3 | 145 | - | - | - | - | - | N |
| 72 | **DC7** | SnaFab | AHV | - | - | - | - | - | - | - | - | - | - | - | - | - | N |
| 73 | **BK8** | Razi | AHV | - | - | - | - | - | - | - | - | - | - | - | - | - | N |
| 74 | **ZK8** | Razi | AHV | - | - | - | - | - | - | - | - | - | - | - | - | - | N |
| 75 | **FM4** | Razi | AHV | 36 | 121 | 6 | 10.7 | 4.73 | 12.7 | 36 | 258 | - | - | **-** | - | - | N |
| 76 | **YP2** | SnaFab | AHV | 36 | 121 | 6 | - | - | - | - | - | - | - | - | - | - | N |
| 77 | **GK9** | Razi | AHV | 12 | 35 | 1 | 9.3 | 2.25 | 6.9 | 19.9 | 101 | 10/0.9 | 140/4.6 | - | 53/40/469 | 0.7/- | N |
| 78 | **FC3** | Razi | AHV | 13.3 | 28 | 1.2 | 11.7 | 4.42 | 13.1 | 38.6 | 250 | - | - | - | - | - | N |
| 79 | **UT8** | Razi | AHV | 17 | 31 | 1.8 | - | - | - | - | - | - | - | - | - | - | N |
| 80 | **QS6** | Razi | AHV | 23 | 60 | 2.7 | 7.4 | 4.05 | 11.5 | 34.3 | 242 | 7/0.8 | 136/3.6 | - | 38/15/- | 0.7/- | N |
| 81 | **RL9** | Razi | AHV | 15.6 | 32 | 1.58 | 4.04 | 4.88 | 14.7 | 43.6 | 283 | 18/0.8 | 140/4 | - | 60/37/470 | 1.7/0.3 | N |
| 82 | **YF3** | SnaFab | AHV | 36 | 121 | 6 | 13.54 | 5.07 | 15.2 | 43.9 | 198 | 16/1.5 | 142/3.6 | 475 | 31/21/712 | 1.9/0.3 | N |
| 83 | **NNK8** | SnaFab | MAS | 8.9 | 27.6 | 0.89 | 10.3 | 4.7 | 14.3 | 41.7 | 192 | 33/0.8 | 138/4.5 | - | - | - | N |
| 84 | **KL5** | Razi | AHV | 13 | 25 | 1.1 | 6.6 | 3.92 | 12.4 | 36.1 | 334 | 10/0.7 | 138/3.9 | 50 | -/16/- | 0.5/- | N |
| 85 | **RU5** | SnaFab | AHV | 13.8 | 24 | 1.28 | 10.56 | 5.51 | 15.5 | 44.5 | 150 | 25/1.1 | 140/4 | - | - | - | N |
| 86 | **OG6** | Razi | AHV | 16 | 38 | 1.5 | 10.7 | 5.44 | 17.7 | 48.7 | 199 | 8/0.9 | 139/3.6 | 68 | -/22/359 | - | N |
| 87 | **RU0** | Razi | AHV | 36 | 36* | 6* | 8.4 | 4.7 | 13.4 | 40.8 | 184 | 13/1 | 138/3.9 | - | - | - | N |
| 88 | **CH8** | Razi | AHV | 60* | 120* | 6* | 8.4 | 4.44 | 11.8 | 34.1 | 274 | 9/0.4 | 142/3.2 | - | - | - | N |
| 89 | **VL2** | SnaFab | AHV | - | - | - | - | - | - | - | - | - | - | - | - | - | - |
| 90 | **DC8** | Razi | AHV | - | - | - | - | - | - | - | - | - | - | - | - | - | N |
| 91 | HV6 | Razi | AHV | 12.3 | 24 | 1.04 | - | - | - | - | - | 25/1.4 | 137/4 | - | -/51/- | 1.2/0.3 | N |
| 92 | FL2 | Razi | AHV | 14.7 | 29 | 1.42 | 11.6 | 5.25 | 16.1 | 47.4 | 210 | 21/0.8 | 136/3.6 | - | - | - | N |
| 93 | RS1 | SnaFab | AHV | DIS | DIS | DIS | DIS | DIS | DIS | DIS | DIS | DIS | DIS | DIS | DIS | DIS | DIS |
| 94 | AJ6 | Razi | AHV | 14 | 30 | 1.2 | 14.2 | 4.47 | 13.1 | 40.2 | 223 | 20/0.9 | 137/4.4 | 204 | -/-/401 | 0.8/- | N |
| 95 | ZY8 | Razi | AHV | 17.6 | 27 | 1.95 | 13.9 | 3.98 | 12.3 | 38 | 265 | - | - | - | - | - | N |
| 96 | YB6 | SnaFab | AHV | 12 | 41 | 1 | 9.6 | 3.75 | 11.4 | 36.3 | 223 | 13/0.6 | 149/3.9 | 98 | - | 0.8/- | N |
| 97 | GS2 | Razi | AHV | 12 | 30 | 1 | 6 | 4.54 | 13 | 39.9 | 177 | 11/0.7 | 132/3.9 | 200 | -/-/435 | - | N |
| 98 | WZ7 | Razi | AHV | 36* | 83 | 6* | 9.2 | 3.9 | 12.1 | 33.3 | 216 | 10/0.7 | 143/3.7 | 90 | -/25/404 | 1.2/0.3 | N |

DIS: Discharged, N= normal, * = result disrupted

## Table 10 - Test results of victims who had abnormal coagulation tests at visit 4 (6 hour post-administration) [recheck in next 12 hours]

| ID | Code | Group | Center | PT | PTT | INR | PLT |
| --- | --- | --- | --- | --- | --- | --- | --- |
| 1 | **YUAB** | SnaFab | AHV | 13.7 | 1.17 | 29 | - |
| 4 | **GHMA** | SnaFab | AHV | - | - | - | - |
| 8 | **RABA** | SnaFab | AHV | 14 | 27 | 1.2 | - |
| 9 | **HAHO** | SnaFab | AHV | 14 | 27 | 1.2 | - |
| 10 | **REAB** | SnaFab | AHV | 36* | 121* | 6* | - |
| 15 | **SASH** | SnaFab | AHV | 22 | 35 | 1.6 | - |
| 16 | **EEZ9** | SnaFab | MAS | - | - | - | - |
| 17 | **DDH8** | Razi | MAS | 11.6 | 25.1 | 0.95 | 173 |
| 27 | **IIE0** | Razi | AHV | 14.3 | 35 | 1.2 | - |
| 28 | **EEG0** | Razi | AHV | 15 | 35 | 1.4 | - |
| 30 | **BBI6** | SnaFab | AHV | 28 | 39 | 4 | - |
| 32 | **AAF9** | Razi | AHV | 14 | 28 | 1.2 | - |
| 34 | **RRS3** | SnaFab | AHV | 17 | 30 | 1.8 | 271 |
| 36 | **QQW3** | SnaFab | AHV | 14 | 31 | 1.3 | 314 |
| 37 | **AAT1** | SnaFab | MAS | - | - | - | - |
| 38 | **VVI7** | Razi | AHV | 22 | 44 | 2.7 | - |
| 39 | **UUL5** | SnaFab | AHV | - | - | - | 206 |
| 40 | **QQQ3** | Razi | AHV | 12.9 | 38 | 1.09 | 228 |
| 43 | **RRA4** | Razi | AHV | 16 | 25 | 1.5 | - |
| 45 | **OOZ0** | Razi | AHV | 16 | 35 | 1.5 | - |
| 50 | **MMM9** | Razi | AHV | 12.4 | 25 | 1.04 | - |
| 52 | **KKW6** | Razi | AHV | 14 | 27 | 1.2 | - |
| 54 | **FFR9** | SnaFab | AHV | 15 | 38 | 1.29 | - |
| 55 | **UUN6** | Razi | AHV | 16.5 | 22 | 1.5 | 319 |
| 58 | **QQA5** | SnaFab | AHV | 15 | 27 | 1.4 | - |
| 59 | **FFT8** | Razi | MAS | - | - | - | - |
| 60 | **JJY3** | SnaFab | AHV | 20 | 34 | 2.9 | - |
| 66 | **GG0** | SnaFab | AHV | - | - | - | - |
| 74 | **ZK8** | Razi | AHV | 36 | 121 | 6 | 236 |
| 75 | **FM4** | Razi | AHV | 19 | 90 | 2 | - |
| 76 | **YP2** | SnaFab | AHV | 24 | 34 | 3.1 | - |
| 79 | **UT8** | Razi | AHV | 16.7 | 30 | 1.78 | 224 |
| 80 | **QS6** | Razi | AHV | 19 | 35 | 2 | 216 |
| 81 | **RL9** | Razi | AHV | - | - | - | - |
| 82 | **YF3** | SnaFab | AHV | - | - | - | - |
| 86 | **OG6** | Razi | AHV | - | - | - | - |
| 87 | **RU0** | Razi | AHV | 25 | 48 | 3.5 | 198 |
| 88 | **CH8** | Razi | AHV | 17.5 | 25 | 1.5 | 264 |
| 90 | **DC8** | Razi | AHV | 15.4 | 35 | 1.3 | - |
| 92 | **FL2** | Razi | AHV | 12 | 30 | 1 | 213 |
| 95 | **ZY8** | Razi | AHV | 12 | 25 | 1 | 247 |
| 98 | **WZ7** | Razi | AHV | 36* | 36* | 6* | - |

DIS: Discharged, N= normal, * = result disrupted

## Table 11 - Test results of victims 24 hours after injection

| ID | Code | Group | Center | PT | PTT | INR | WBC | RBC | Hb | HCT | PLT | BUN/ Cr | Na/ K | CPK | AST/  ALT/LDH | BIL total/ direct | ECG |
| --- | --- | --- | --- | --- | --- | --- | --- | --- | --- | --- | --- | --- | --- | --- | --- | --- | --- |
| 1 | **YUAB** | SnaFab | AHV | 15 | 30 | 1.4 | - | - | - | - | - | - | - | - | - | - | - |
| 2 | **MAGH** | SnaFab | AHV | - | - | - | - | - | - | - | - | - | - | - | - | - | - |
| 3 | **ALSA** | SnaFab | AHV | DIS | DIS | DIS | DIS | DIS | DIS | DIS | DIS | DIS | DIS | DIS | DIS | DIS | DIS |
| 4 | **GHMA** | SnaFab | AHV | 12.8 | 30 | 1.08 | - | - | - | - | - | - | - | - | - | - | - |
| 5 | **ABAR** | SnaFab | AHV | DIS | DIS | DIS | DIS | DIS | DIS | DIS | DIS | DIS | DIS | DIS | DIS | DIS | DIS |
| 6 | **HABA** | SnaFab | AHV | - | - | - | - | - | - | - | - | - | - | - | - | - | - |
| 7 | **FASO** | SnaFab | AHV | - | - | - | - | - | - | - | - | - | - | - | - | - | - |
| 8 | **RABA** | SnaFab | AHV | 17.8 | 39 | 2.01 | 12.2 | 5.16 | 15.3 | 44.2 | 252 | - | - | - | - | - | - |
| 9 | **HAHO** | SnaFab | AHV | 13.7 | 38 | 1.2 | 13.7 | 5.63 | 14.4 | 43.5 | 163 | - | - | - | - | - | - |
| 10 | **REAB** | SnaFab | AHV | - | - | - | 8.42 | 5.31 | 15.1 | 45.3 | 161 | - | - | - | - | - | - |
| 11 | **ALSE** | SnaFab | MAS | 12.9 | 28.7 | 1.09 | 8.9 | 4.54 | 13 | 38.3 | 225 | 25/1.1 | 139/3.8 | 81 | 18/15/251.1 | 1.7/0.3 | N |
| 12 | **BAEB** | SnaFab | URM | 13 | 25.3 | 1 | 13.37 | 4.96 | 15 | 41.6 | 211 | 14.5/0.93 | 139/4.3 | 72 | 23/48/274 | - | - |
| 13 | **FAMA** | SnaFab | AHV | 17.0 | 25 | 1.6 | - | - | - | - | - | - | - | - | - | - | - |
| 14 | **ALHA** | SnaFab | AHV | - | - | - | - | - | - | - | - | - | - | - | - | - | - |
| 15 | **SASH** | SnaFab | AHV | 18.7 | 39 | 2.21 | - | - | - | - | - | - | - | - | - | - | N |
| 16 | **EEZ9** | SnaFab | MAS | 14.3 | 28.3 | 1.24 | 8.2 | 4.48 | 12.9 | 39.1 | 74 | - | - | - | - | - | N |
| 17 | **DDH8** | Razi | MAS | 11.6 | 25.1 | 0.95 | 9.3 | 3.28 | 10.1 | 30.4 | 173 | 6.6/0.8 | 145/3.7 | 157 | - | - | N |
| 18 | **QQG5** | Razi | MAS | 13.6 | 30 | 1.22 | 7.5 | 4.2 | 12.1 | 34.6 | 178 | 5.8/0.7 | 137/3.5 | - | 17/10 | 0.5/0.2 | N |
| 19 | **XXA1** | SnaFab | MAS | 12.5 | 33.2 | 1.07 | 6.6 | 4.77 | 13.3 | 40.3 | 235 | 5.8/0.7 | 139/4.7 | - | 17/9 | 0.2/0.1 | N |
| 20 | **SSV7** | Razi | MAS | 11.4 | 23.9 | 0.92 | - | - | - | - | - | - | - | - | - | - | N |
| 21 | **BBM6** | Razi | MAS | - | - | - | - | - | - | - | - | - | - | - | 15/16/252 | 1.1/0.4 | N |
| 22 | **YYY4** | SnaFab | MAS | - | - | - | 4.8 | 5 | 14 | 45.3 | 189 | - | - | - | 17/15/355 | 0.5/0.2 | N |
| 23 | **ZZI1** | SnaFab | URM | 17 | 34 | 1.48 | 18 | 5.24 | 16 | - | 136 | 32/1 | - | - | - | - | N |
| 24 | **SSU8** | SnaFab | MAS | 13.4 | 31 | 1.19 | 8 | 4.3 | 13 | 38 | 205 | 9.5/1.1 | 144/4 | 86 | 20/10/295 | 0.3/0.1 | N |
| 25 | **NNZ3** | Razi | URM | - | - | - | - | - | - | - | - | - | - | - | - | - | N |
| 26 | **FFG3** | SnaFab | AHV | 16 | 30 | 1.6 | 19.7 | 5.25 | 15.3 | 47.1 | 220 | 15/0.7 | 137/3.8 | - | 23/-/487 | 1.1/- | N |
| 27 | **IIE0** | Razi | AHV | 12 | 48 | 1 | - | - | - | - | - | 32/1.9 | 133/3.2 | - | - | - | N |
| 28 | **EEG0** | Razi | AHV | 15 | 34 | 1.4 | 14.2 | 5.45 | 14.8 | 45.4 | 202 | - | - | - | - | - | N |
| 29 | **JJW4** | SnaFab | AHV | 13 | 30 | 1.1 | - | - | - | - | - | - | - | - | - | - | N |
| 30 | **BBI6** | SnaFab | AHV | 19 | 31 | 2.1 | 18.4 | 5.16 | 12.7 | 40 | 224 | 13/1 | - | - | - | - | N |
| 31 | **AAH7** | SnaFab | AHV | 18 | 30 | 1.9 | - | - | - | - | - | - | - | - | - | - | N |
| 32 | **AAF9** | Razi | AHV | 15 | 33 | 1.4 | 7 | 6.78 | 12.5 | 43.3 | 199 | - | - | - | - | - | N |
| 33 | **WWR1** | Razi | AHV | 12.4 | 32 | 1.04 | - | - | - | - | - | - | - | - | - | - | N |
| 34 | **RRS3** | SnaFab | AHV | 14 | 27 | 1.3 | - | - | - | - | - | - | - | - | - | - | N |
| 35 | **JJI8** | Razi | AHV | 13 | 29 | 1.15 | - | - | - | - | - | - | - | - | - | - | N |
| 36 | **QQW3** | SnaFab | AHV | 15 | 35 | 1.4 | 6.9 | 4.71 | 12.5 | 39.9 | 303 | - | - | - | - | - | N |
| 37 | **AAT1** | SnaFab | MAS | 12 | 28 | 1 | 5.9 | 4.13 | 9.3 | 30.3 | 183 | 10/0.7 | 136.5/4.4 | - | 14/27/- | 0.9/0.5 | N |
| 38 | **VVI7** | Razi | AHV | 21 | 30 | 2.6 | 34.2 | 6.88 | 20.4 | 62.5 | 278 | - | - | - | - | - | N |
| 39 | **UUL5** | SnaFab | AHV | 14 | 27 | 1.3 | - | - | - | - | - | - | - | - | - | - | N |
| 40 | **QQQ3** | Razi | AHV | 16 | 34 | 1.6 | 21.4 | 5.48 | 15.3 | 46.3 | 267 | -/1.4 | 142/3 | - | - | - | N |
| 41 | **UUN5** | Razi | AHV | 14 | 34 | 1.2 | 17.77 | 4.68 | 13.3 | 38.7 | 247 | 16/0.9 | 138/3.9 |  | 26/40/- | 1.3/0.2 | N |
| 42 | **XXN0** | SnaFab | AHV | - | - | - | - | - | - | - | - | - | - | - | - | - | N |
| 43 | **RRA4** | Razi | AHV | - | - | - | - | - | - | - | - | - | - | - | - | - | N |
| 44 | **DDN5** | SnaFab | AHV | - | - | - | - | - | - | - | - | - | - | - | - | - | N |
| 45 | **OOZ0** | Razi | AHV | 15 | 25 | 1.4 | 11.25 | 3.83 | 11.3 | 33.7 | 156 | 13/1 | 136/4.1 | - | - | - | N |
| 46 | **UUT4** | SnaFab | AHV | 13 | 25 | 1.1 | - | - | - | - | - | - | - | - | - | - | N |
| 47 | **GGM9** | SnaFab | AHV | 13 | 25 | 1.1 | - | - | - | - | - | - | - | - | - | - | N |
| 48 | **SSH4** | SnaFab | AHV | 16 | 33 | 1.6 | 17.2 | 5.19 | 16.6 | 47.9 | 234 | - | - | **-** | - | - | N |
| 49 | **FFW7** | Razi | AHV | 14 | 35 | 1.3 | 12.6 | 5.42 | 15.8 | 48.4 | 240 | 22/1.6 | 133/4.1 | - | - | - | N |
| 50 | **MMM9** | Razi | AHV | 13 | 32 | 1.1 | 17.1 | 5.13 | 14.3 | 43.6 | 169 | 18/1.4 | 138/4.2 | **-** | - | 0.6/- | N |
| 51 | **KKZ6** | SnaFab | AHV | 14 | 25 | 1.3 | - | - | - | - | - | - | - | - | - | - | N |
| 52 | **KKW6** | Razi | AHV | 13 | 27 | 1.1 | - | - | - | - | - | 12/1.2 | 136/3.9 | - | - | - | N |
| 53 | **EEP5** | Razi | AHV | - | - | - | - | - | - | - | - | - | - | - | - | - | N |
| 54 | **FFR9** | SnaFab | AHV | 14 | 43 | 1.2 | 14.6 | 5.47 | 15.7 | 44.3 | 219 | - | - | - | - | - | N |
| 55 | **UUN6** | Razi | AHV | 14 | 29 | 1.1 | - | - | - | - | - | - | - | - | - | - | N |
| 56 | **BBP7** | SnaFab | MAS | 13.2 | 32 | 1.09 | 8.4 | 3.75 | 11.3 | 35.4 | 192 | 18/0.6 | 138/3.9 | - | - | - | N |
| 57 | **OOE4** | Razi | AHV | 13 | 32 | 1 | - | - | - | - | - | - | - | - | - | - | N |
| 58 | **QQA5** | SnaFab | AHV | 15 | 34 | 1.4 | - | - | - | - | - | - | - | - | - | - | N |
| 59 | **FFT8** | Razi | MAS | 14.3 | 32.6 | 1.33 | - | - | - | - | - | - | - | - | 14/8/- | 0.6/0.2 | N |
| 60 | **JJY3** | SnaFab | AHV | 15 | 30 | 1.4 | 22.3 | 5.21 | 15.5 | 43.6 | 258 | - | - | - | - | - | N |
| 61 | **GGN0** | Razi | AHV | 13 | 35 | 1.1 | - | - | - | - | - | - | - | - | - | - | N |
| 62 | **ZZQ0** | SnaFab | AHV | 14 | 26 | 1.2 | 12.4 | 5.35 | 14.6 | 44.3 | 133 | - | - | - | - | - | N |
| 63 | **JJK9** | Razi | AHV | 14 | 30 | 1.2 | - | - | - | - | - | 22/1.7 | 135/4.2 | - | - | - | N |
| 64 | **YYE2** | SnaFab | AHV | - | 24 | - | 17.13 | 4.23 | 13 | 38.5 | 240 | - | - | - | - | - | N |
| 65 | **TTJ2** | Razi | MAS | 12 | 30 | 1.09 | 8.9 | 4.95 | 15.4 | 42.6 | 214 | 12.6/0.8 | 138/3.9 | - | 22/6/- | 2.9/0.9 | N |
| 66 | **GG0** | SnaFab | AHV | 13 | 27 | 1.1 | - | - | - | - | - | - | - | - | - | - | N |
| 67 | **NN5** | Razi | AHV | 13.3 | 33 | 1.13 | - | - | - | - | - | - | - | - | - | - | N |
| 68 | **JX8** | Razi | AHV | 12 | 28 | 1 | 8.59 | 4.62 | 12.9 | 39 | 175 | - | - | - | - | - | N |
| 69 | **QU4** | Razi | AHV | DIS | DIS | DIS | DIS | DIS | DIS | DIS | DIS | DIS | DIS | DIS | DIS | DIS | DIS |
| 70 | **EEJ8** | Razi | URM | 13 | 35 | 1.1 | 12.6 | - | 16.1 | - | 200 | 38/0.8 | 142/3.9 | 242 | 14/16/317 | - | N |
| 71 | **WT3** | Razi | AHV | 14 | 35 | 1.1 | 7.1 | 3.56 | 9.9 | 30.3 | 139 | - | - | - | - | - | N |
| 72 | **DC7** | SnaFab | AHV | DIS | DIS | DIS | DIS | DIS | DIS | DIS | DIS | DIS | DIS | DIS | DIS | DIS | DIS |
| 73 | **BK8** | Razi | AHV | 17 | 55 | 1.6 | 12.9 | 5.34 | 15.1 | 45.4 | 176 | - | - | - | - | - | N |
| 74 | **ZK8** | Razi | AHV | 15 | 50 | 1.4 | 15.1 | 4.2 | 11.1 | 34.8 | 238 | - | - | - | - | - | N |
| 75 | **FM4** | Razi | AHV | 14.4 | 46 | 1.37 | 10.67 | 3.99 | 10.9 | 32.1 | 219 | - | - | - | -/-/407 | - | N |
| 76 | **YP2** | SnaFab | AHV | 14.1 | 36 | 1.32 | - | - | - | - | - | - | - | - | - | - | N |
| 77 | **GK9** | Razi | AHV | DIS | DIS | DIS | DIS | DIS | DIS | DIS | DIS | DIS | DIS | DIS | DIS | DIS | DIS |
| 78 | **FC3** | Razi | AHV | 13 | 25 | 1.1 | - | - | - | - | - | - | - | - | - | - | N |
| 79 | **UT8** | Razi | AHV | 14 | 30 | 1.2 | 14 | 5.14 | 15 | 46.5 | 263 | 10/1.2 | 137/3.6 | - | - | - | N |
| 80 | **QS6** | Razi | AHV | 14 | 36 | 1.3 | 5.2 | 4.08 | 11.6 | 34.7 | 205 | - | - | - | - | - | N |
| 81 | **RL9** | Razi | AHV | 14 | 39 | 1.6 | 9.1 | 4.6 | 15 | 44.7 | 304 | 17/0.9 | 137/3.2 | 161 | 18/-/486 | 0.5/- | N |
| 82 | **YF3** | SnaFab | AHV | 17 | 40 | 1.7 | 12.3 | 4.08 | 12.5 | 37.7 | 202 | 14/1 | 141/3.5 | - | -/20/695 | 0.5/- | N |
| 83 | **NNK8** | SnaFab | MAS | DIS | DIS | DIS | DIS | DIS | DIS | DIS | DIS | DIS | DIS | DIS | DIS | DIS | DIS |
| 84 | **KL5** | Razi | AHV | 13 | 32 | 1.1 | 12.4 | 3.42 | 10.3 | 32 | 328 | 15/0.7 | 139/3.9 | - | - | - | N |
| 85 | **RU5** | SnaFab | AHV | 13 | 28 | 1.1 | 12.3 | 4.53 | 13.1 | 39.5 | 165 | 15/0.9 | 143/3.4 | 209 | -/18/405 | 0.7/- | N |
| 86 | **OG6** | Razi | AHV | 12 | 26 | 1 | 12.1 | 5.05 | 17.4 | 50.5 | 237 | 9/1 | 140/3.6 | 61 | -/18/327 | 0.5/- | N |
| 87 | **RU0** | Razi | AHV | 18 | 32 | 1.9 | 11.2 | 4.36 | 12.9 | 39.5 | 192 | 14/0.9 | 138/3.5 | 134 | -/45/390 | 0.6/- | N |
| 88 | **CH8** | Razi | AHV | - | - | - | 6.4 | 4.53 | 11.6 | 35.1 | 280 | 5/0.4 | - | 135 | 41/43/420 | - | N |
| 89 | **VL2** | SnaFab | AHV | DIS | DIS | DIS | DIS | DIS | DIS | DIS | DIS | DIS | DIS | DIS | DIS | DIS | DIS |
| 90 | **DC8** | Razi | AHV | 12.7 | 35 | 1.1 | 4.7 | 4.96 | 13.2 | 40.3 | 198 | - | - | - | - | - | N |
| 91 | HV6 | Razi | AHV | 13 | 36 | 1.1 | 15.2 | 3.82 | 12.7 | 40.8 | 242 | 28/1.3 | 142/4.2 | 175 | - | 1/- | N |
| 92 | FL2 | Razi | AHV | DIS | DIS | DIS | DIS | DIS | DIS | DIS | DIS | DIS | DIS | DIS | DIS | DIS | DIS |
| 93 | RS1 | SnaFab | AHV | DIS | DIS | DIS | DIS | DIS | DIS | DIS | DIS | DIS | DIS | DIS | DIS | DIS | DIS |
| 94 | AJ6 | Razi | AHV | 14 | 31 | 1.3 | 13.8 | 4.26 | 12.1 | 38.6 | 259 | - | - | - | - | - | N |
| 95 | ZY8 | Razi | AHV | 14 | 35 | 1.3 | 17 | 3.79 | 11.5 | 36.3 | 277 | - | - | - | - | - | N |
| 96 | YB6 | SnaFab | AHV | DIS | DIS | DIS | DIS | DIS | DIS | DIS | DIS | DIS | DIS | DIS | DIS | DIS | DIS |
| 97 | GS2 | Razi | AHV | 13 | 25 | 1.1 | 12.9 | 4.7 | 13.7 | 41.5 | 209 | 8/0.7 | - | 134 | -/-/322 | 0.7/- | N |
| 98 | WZ7 | Razi | AHV | DIS | DIS | DIS | DIS | DIS | DIS | DIS | DIS | DIS | DIS | - | DIS | DIS | DIS |

DIS: Discharged, N= normal, * = result disrupted

## Table 12 - Lab results of victims 48 hours after injection

| ID | Code | Group | Center | PT | PTT | INR | WBC | RBC | Hb | HCT | PLT | BUN/ Cr | Na/ K | CPK | AST/  ALT/LDH | BIL total/ direct | ECG |
| --- | --- | --- | --- | --- | --- | --- | --- | --- | --- | --- | --- | --- | --- | --- | --- | --- | --- |
| 1 | **YUAB** | SnaFab | AHV | 17 | 30 | 1.7 | - | - | - | - | - | - | - | - | - | - | - |
| 2 | **MAGH** | SnaFab | AHV | - | - | - | - | - | - | - | - | - | - | - | - | - | - |
| 3 | **ALSA** | SnaFab | AHV | DIS | DIS | DIS | DIS | DIS | DIS | DIS | DIS | DIS | DIS | DIS | DIS | DIS | DIS |
| 4 | **GHMA** | SnaFab | AHV | 13.9 | 31 | 1.23 | 8.8 | 3.57 | 9.7 | 28.8 | 199 | - | - | - | - | - | - |
| 5 | **ABAR** | SnaFab | AHV | DIS | DIS | DIS | DIS | DIS | DIS | DIS | DIS | DIS | DIS | DIS | DIS | DIS | DIS |
| 6 | **HABA** | SnaFab | AHV | DIS | DIS | DIS | DIS | DIS | DIS | DIS | DIS | DIS | DIS | DIS | DIS | DIS | DIS |
| 7 | **FASO** | SnaFab | AHV | - | - | - | - | - | - | - | - | - | - | - | - | - | - |
| 8 | **RABA** | SnaFab | AHV | 16.9 | 33 | 1.81 | 11.8 | 5.15 | 16 | 46.6 | 228 | - | - | - | - | - | - |
| 9 | **HAHO** | SnaFab | AHV | 12.2 | 24 | 1.6 | 13.1 | 5.07 | 13.8 | 40.6 | 133 | - | - | - | - | - | - |
| 10 | **REAB** | SnaFab | AHV | DIS | DIS | DIS | DIS | DIS | DIS | DIS | DIS | DIS | DIS | DIS | DIS | DIS | DIS |
| 11 | **ALSE** | SnaFab | MAS | 12.6 | 30 | 1.06 | 8.1 | 4.47 | 13.4 | 38.8 | 198 | 25.2/0.9 | 138/3.9 | 88 | 18/15/251 | 1.7/0.3 | - |
| 12 | **BAEB** | SnaFab | URM | DIS | DIS | DIS | DIS | DIS | DIS | DIS | DIS | DIS | DIS | DIS | DIS | DIS | DIS |
| 13 | **FAMA** | SnaFab | AHV | 16 | 30 | 1.6 | DIS | DIS | DIS | DIS | DIS | DIS | DIS | DIS | DIS | DIS | DIS |
| 14 | **ALHA** | SnaFab | AHV | DIS | DIS | DIS | DIS | DIS | DIS | DIS | DIS | DIS | DIS | DIS | DIS | DIS | DIS |
| 15 | **SASH** | SnaFab | AHV | 19.7 | 37 | 2.45 | 7.94 | 5.01 | 13.1 | 42.1 | 188 | - | - | - | - | - | - |
| 16 | **EEZ9** | SnaFab | MAS | 14.1 | 30 | - | 5.7 | 4.43 | 13 | 38 | 71` | 6.6/0.9 | 140/3.6 | - | - | - | - |
| 17 | **DDH8** | Razi | MAS | 13.5 | 33 | 1.21 | 4.8 | 3.49 | 10.5 | 31 | 166 | 8.3/0.7 | 140/3.5 | 99 | - | - | - |
| 18 | **QQG5** | Razi | MAS | 13.2 | 30 | 1.16 | 8.1 | 4.68 | 13.8 | 38.4 | 190 | - | - | - | - | - | - |
| 19 | **XXA1** | SnaFab | MAS | DIS | DIS | DIS | DIS | DIS | DIS | DIS | DIS | DIS | DIS | DIS | DIS | DIS | DIS |
| 20 | **SSV7** | Razi | MAS | DIS | DIS | DIS | DIS | DIS | DIS | DIS | DIS | DIS | DIS | DIS | DIS | DIS | DIS |
| 21 | **BBM6** | Razi | MAS | DIS | DIS | DIS | DIS | DIS | DIS | DIS | DIS | DIS | DIS | DIS | DIS | DIS | DIS |
| 22 | **YYY4** | SnaFab | MAS | DIS | DIS | DIS | DIS | DIS | DIS | DIS | DIS | DIS | DIS | DIS | DIS | DIS | DIS |
| 23 | **ZZI1** | SnaFab | URM | - | - | - | 7.11 | 4.85 | 13.5 | - | 150 | 30/0.94 | 136/3.8 | 69 | - | - | - |
| 24 | **SSU8** | SnaFab | MAS | DIS | DIS | DIS | DIS | DIS | DIS | DIS | DIS | DIS | DIS | DIS | DIS | DIS | DIS |
| 25 | **NNZ3** | Razi | URM | DIS | DIS | DIS | DIS | DIS | DIS | DIS | DIS | DIS | DIS | DIS | DIS | DIS | DIS |
| 26 | **FFG3** | SnaFab | AHV | 17 | 25 | 1.8 | 14.7 | 4.93 | 14.6 | 44.7 | 206 | - | - | - | - | - | - |
| 27 | **IIE0** | Razi | AHV | 28 | 100 | 4 | 9.57 | 5.05 | 14.5 | 43.8 | 202 | 27/1.4 | 137/3.6 | - | - | - | - |
| 28 | **EEG0** | Razi | AHV | 14 | 36 | 1.3 | 12.5 | 5.25 | 14.7 | 44.5 | 200 | - | - | - | - | - | - |
| 29 | **JJW4** | SnaFab | AHV | 15 | 35 | 1.4 | - | - | - | - | - | - | - | - | - | - | - |
| 30 | **BBI6** | SnaFab | AHV | 15 | 31 | 1.4 | - | - | - | - | - | - | - | - | - | - | - |
| 31 | **AAH7** | SnaFab | AHV | 14 | 25 | 1.3 | 11.3 | 5.12 | 14.9 | 43.5 | 221 | - | - | - | - | - | - |
| 32 | **AAF9** | Razi | AHV | DIS | DIS | DIS | DIS | DIS | DIS | DIS | DIS | DIS | DIS | DIS | DIS | DIS | DIS |
| 33 | **WWR1** | Razi | AHV | 14 | 30 | 1.2 | - | - | - | - | - | - | - | - | - | - | - |
| 34 | **RRS3** | SnaFab | AHV | DIS | DIS | DIS | DIS | DIS | DIS | DIS | DIS | DIS | DIS | DIS | DIS | DIS | DIS |
| 35 | **JJI8** | Razi | AHV | 13 | 25 | 1.1 | - | - | - | - | - | - | - | - | - | - | - |
| 36 | **QQW3** | SnaFab | AHV | 18 | 32 | 1.8 | - | - | - | - | - | - | - | - | - | - | - |
| 37 | **AAT1** | SnaFab | MAS | DIS | DIS | DIS | DIS | DIS | DIS | DIS | DIS | DIS | DIS | DIS | DIS | DIS | DIS |
| 38 | **VVI7** | Razi | AHV | 13 | 28 | 1.1 | 22.6 | 5.47 | 15.7 | 49.3 | 240 | - | - | - | - | - | - |
| 39 | **UUL5** | SnaFab | AHV | 16 | 25 | 1.6 | 12.2 | 5.79 | 16.5 | 49.3 | 186 | - | - | - | - | - | - |
| 40 | **QQQ3** | Razi | AHV | 14 | 25 | 1.3 | 24.3 | 5.57 | 16.1 | 47.5 | 303 | 14/1.3 | 136/3.1 | - | - | - | N |
| 41 | **UUN5** | Razi | AHV | 17 | 30 | 1.8 | 16.5 | 4.38 | 11.9 | 36.5 | 220 | -/1.1 | 137/2.9 | - | - | 0.6/- | - |
| 42 | **XXN0** | SnaFab | AHV | DIS | DIS | DIS | DIS | DIS | DIS | DIS | DIS | DIS | DIS | DIS | DIS | DIS | DIS |
| 43 | **RRA4** | Razi | AHV | DIS | DIS | DIS | DIS | DIS | DIS | DIS | DIS | DIS | DIS | DIS | DIS | DIS | DIS |
| 44 | **DDN5** | SnaFab | AHV | DIS | DIS | DIS | DIS | DIS | DIS | DIS | DIS | DIS | DIS | DIS | DIS | DIS | DIS |
| 45 | **OOZ0** | Razi | AHV | 12.3 | 25 | 1.03 | 11.71 | 4.37 | 13 | 38.4 | 202 | 15/0.7 | 140/3.6 | - | - | - | - |
| 46 | **UUT4** | SnaFab | AHV | 12 | 25 | 1 | - | - | - | - | - | - | - | - | - | - | - |
| 47 | **GGM9** | SnaFab | AHV | 14 | 27 | 1.3 | - | - | - | - | - | - | - | - | - | - | - |
| 48 | **SSH4** | SnaFab | AHV | 13.1 | 24 | 1.1 | - | - | - | - | - | - | - | - | - | - | - |
| 49 | **FFW7** | Razi | AHV | 15 | 31 | 1.4 | 8.3 | 4.01 | 11.4 | 35.4 | 183 | -/1.4 | 125/3.1 | - | - | - | - |
| 50 | **MMM9** | Razi | AHV | 14 | 45 | 1.3 | - | - | - | - | - | - | - | - | - | - | - |
| 51 | **KKZ6** | SnaFab | AHV | 13 | 25 | 1.1 | - | - | - | - | - | - | - | - | - | - | - |
| 52 | **KKW6** | Razi | AHV | DIS | DIS | DIS | DIS | DIS | DIS | DIS | DIS | DIS | DIS | DIS | DIS | DIS | DIS |
| 53 | **EEP5** | Razi | AHV | DIS | DIS | DIS | DIS | DIS | DIS | DIS | DIS | DIS | DIS | DIS | DIS | DIS | DIS |
| 54 | **FFR9** | SnaFab | AHV | 13 | 39 | 1.1 | - | - | - | - | - | - | - | - | - | - | - |
| 55 | **UUN6** | Razi | AHV | 13 | 26 | 1 | - | - | - | - | - | - | - | - | - | - | - |
| 56 | **BBP7** | SnaFab | MAS | DIS | DIS | DIS | DIS | DIS | DIS | DIS | DIS | DIS | DIS | DIS | DIS | DIS | DIS |
| 57 | **OOE4** | Razi | AHV | DIS | DIS | DIS | DIS | DIS | DIS | DIS | DIS | DIS | DIS | DIS | DIS | DIS | DIS |
| 58 | **QQA5** | SnaFab | AHV | DIS | DIS | DIS | DIS | DIS | DIS | DIS | DIS | DIS | DIS | DIS | DIS | DIS | DIS |
| 59 | **FFT8** | Razi | MAS | 11 | 40 | 0.92 | - | - | - | - | - | - | - | - | - | - | - |
| 60 | **JJY3** | SnaFab | AHV | 12 | 39 | 1 | - | - | - | - | - | - | - | - | - | - | - |
| 61 | **GGN0** | Razi | AHV | - | - | - | - | - | - | - | - | - | - | - | - | - | N |
| 62 | **ZZQ0** | SnaFab | AHV | - | - | - | 10.9 | 4.98 | 14.4 | 43.9 | 114 | - | - | - | - | - | N |
| 63 | **JJK9** | Razi | AHV | 14 | 32 | 1.3 | 12.7 | 4.4 | 13.5 | 39.8 | 217 | 16/1 | 138/3.7 | - | - | - | N |
| 64 | **YYE2** | SnaFab | AHV | DIS | DIS | DIS | DIS | DIS | DIS | DIS | DIS | DIS | DIS | DIS | DIS | DIS | DIS |
| 65 | **TTJ2** | Razi | MAS | DIS | DIS | DIS | DIS | DIS | DIS | DIS | DIS | DIS | DIS | DIS | DIS | DIS | DIS |
| 66 | **GG0** | SnaFab | AHV | 14 | 28 | 1.3 | - | - | - | - | - | - | - | - | - | - | N |
| 67 | **NN5** | Razi | AHV | 12.8 | 26 | 1.08 | - | - | - | - | - | - | - | - | - | - | N |
| 68 | **JX8** | Razi | AHV | DIS | DIS | DIS | DIS | DIS | DIS | DIS | DIS | DIS | DIS | DIS | DIS | DIS | DIS |
| 69 | **QU4** | Razi | AHV | DIS | DIS | DIS | DIS | DIS | DIS | DIS | DIS | DIS | DIS | DIS | DIS | DIS | DIS |
| 70 | **EEJ8** | Razi | URM | 13 | 31 | 1.1 | 9.5 | - | 15.5 | - | 306 | 35/0.8 | 144/4.4 | 202 | 11/14/250 | 0.9/0.3 | N |
| 71 | **WT3** | Razi | AHV | DIS | DIS | DIS | DIS | DIS | DIS | DIS | DIS | DIS | DIS | DIS | DIS | DIS | DIS |
| 72 | **DC7** | SnaFab | AHV | DIS | DIS | DIS | DIS | DIS | DIS | DIS | DIS | DIS | DIS | DIS | DIS | DIS | DIS |
| 73 | **BK8** | Razi | AHV | 14 | 33 | 1.3 | 11.4 | 5.27 | 14.9 | 46.5 | 174 | - | - | - | - | - | N |
| 74 | **ZK8** | Razi | AHV | 27 | 72 | 3.8 | 10.5 | 4.08 | 10.5 | 30.6 | 210 | 10/0.8 | 137/3.9 | - | - | - | N |
| 75 | **FM4** | Razi | AHV | DIS | DIS | DIS | DIS | DIS | DIS | DIS | DIS | DIS | DIS | DIS | DIS | DIS | DIS |
| 76 | **YP2** | SnaFab | AHV | 12 | 33 | 1.3 | 24 | 5.11 | 14.4 | 41.1 | 190 | 15/1.3 | - | - | - | - | N |
| 77 | **GK9** | Razi | AHV | DIS | DIS | DIS | DIS | DIS | DIS | DIS | DIS | DIS | DIS | DIS | DIS | DIS | DIS |
| 78 | **FC3** | Razi | AHV | 12 | 24 | 1 | 8.13 | 4.33 | 12.9 | 38 | 239 | - | - | - | - | - | N |
| 79 | **UT8** | Razi | AHV | 14 | 25 | 1.3 | 14.2 | 4.68 | 13.4 | 42.1 | 274 | 7/1 | 136/3.5 | - | - | - | N |
| 80 | **QS6** | Razi | AHV | 15 | 33 | 1.4 | 6.1 | 4.23 | 12.1 | 36 | 224 | 13/0.7 | 148/4.2 | - | 55/13/- | 0.5/- | N |
| 81 | **RL9** | Razi | AHV | 13 | 30 | 1.3 | 9.5 | 4.16 | 13.6 | 40.3 | 318 | 19/1.2 | 137/3.5 | 119 | -/16/395 | 0.7/- | N |
| 82 | **YF3** | SnaFab | AHV | DIS | DIS | DIS | DIS | DIS | DIS | DIS | DIS | DIS | DIS | DIS | DIS | DIS | DIS |
| 83 | **NNK8** | SnaFab | MAS | DIS | DIS | DIS | DIS | DIS | DIS | DIS | DIS | DIS | DIS | DIS | DIS | DIS | DIS |
| 84 | **KL5** | Razi | AHV | 13.6 | 24 | 1.24 | - | - | - | - | - | - | - | - | - | - | N |
| 85 | **RU5** | SnaFab | AHV | DIS | DIS | DIS | DIS | DIS | DIS | DIS | DIS | DIS | DIS | DIS | DIS | DIS | DIS |
| 86 | **OG6** | Razi | AHV | DIS | DIS | DIS | DIS | DIS | DIS | DIS | DIS | DIS | DIS | DIS | DIS | DIS | DIS |
| 87 | **RU0** | Razi | AHV | 16 | 40 | 1.6 | 5.9 | 3.96 | 11 | 35.6 | 156 | 10/1.1 | 140/3.5 | - | -/27/365 | 0.7/- | N |
| 88 | **CH8** | Razi | AHV | 13 | 30 | 1 | - | - | - | - | - | - | - | - | - | - | N |
| 89 | **VL2** | SnaFab | AHV | DIS | DIS | DIS | DIS | DIS | DIS | DIS | DIS | DIS | DIS | DIS | DIS | DIS | DIS |
| 90 | **DC8** | Razi | AHV | 13.6 | 40 | 1.1 | 5.1 | 5.04 | 13.5 | 41.2 | 195 | 5/0.6 | - | - | - | - | N |
| 91 | HV6 | Razi | AHV | 14 | 38 | 1.3 | 8.8 | 3.71 | 11.7 | 35.8 | 191 | 22/0.9 | 139/3.9 | - | - | - | N |
| 92 | FL2 | Razi | AHV | DIS | DIS | DIS | DIS | DIS | DIS | DIS | DIS | DIS | DIS | DIS | DIS | DIS | DIS |
| 93 | RS1 | SnaFab | AHV | DIS | DIS | DIS | DIS | DIS | DIS | DIS | DIS | DIS | DIS | DIS | DIS | DIS | DIS |
| 94 | AJ6 | Razi | AHV | DIS | DIS | DIS | DIS | DIS | DIS | DIS | DIS | DIS | DIS | DIS | DIS | DIS | DIS |
| 95 | ZY8 | Razi | AHV | 15.5 | 24 | 1.56 | 19.6 | 4.33 | 12.7 | 39 | 287 | - | - | - | - | - | N |
| 96 | YB6 | SnaFab | AHV | DIS | DIS | DIS | DIS | DIS | DIS | DIS | DIS | DIS | DIS | DIS | DIS | DIS | DIS |
| 97 | GS2 | Razi | AHV | DIS | DIS | DIS | DIS | DIS | DIS | DIS | DIS | DIS | DIS | DIS | DIS | DIS | DIS |
| 98 | WZ7 | Razi | AHV | DIS | DIS | DIS | DIS | DIS | DIS | DIS | DIS | DIS | DIS | DIS | DIS | DIS | DIS |

DIS: Discharged, N= normal, * = result distrupted

## Table 13 - Lab results of victims 72 hours after injection

| ID | Code | Group | Center | PT | PTT | INR | WBC | RBC | Hb | HCT | PLT | BUN/ Cr | Na/ K | CPK | AST/  ALT/LDH | BIL total/ direct | ECG |
| --- | --- | --- | --- | --- | --- | --- | --- | --- | --- | --- | --- | --- | --- | --- | --- | --- | --- |
| 1 | **YUAB** | SnaFab | AHV | 16 | 29 | 1.6 | - | - | - | - | - | - | - | - | - | - | - |
| 2 | **MAGH** | SnaFab | AHV | DIS | DIS | DIS | DIS | DIS | DIS | DIS | DIS | DIS | DIS | DIS | DIS | DIS | DIS |
| 3 | **ALSA** | SnaFab | AHV | DIS | DIS | DIS | DIS | DIS | DIS | DIS | DIS | DIS | DIS | DIS | DIS | DIS | DIS |
| 4 | **GHMA** | SnaFab | AHV | 13.7 | 25 | 1.2 | 6.1 | 3.31 | 8.8 | 26.7 | 162 | - | - | - | - | - | - |
| 5 | **ABAR** | SnaFab | AHV | DIS | DIS | DIS | DIS | DIS | DIS | DIS | DIS | DIS | DIS | DIS | DIS | DIS | DIS |
| 6 | **HABA** | SnaFab | AHV | DIS | DIS | DIS | DIS | DIS | DIS | DIS | DIS | DIS | DIS | DIS | DIS | DIS | DIS |
| 7 | **FASO** | SnaFab | AHV | DIS | DIS | DIS | DIS | DIS | DIS | DIS | DIS | DIS | DIS | DIS | DIS | DIS | DIS |
| 8 | **RABA** | SnaFab | AHV | 16.9 | 28 | 1.81 | - | - | - | - | - | - | - | - | - | - | - |
| 9 | **HAHO** | SnaFab | AHV | 13.3 | 36 | 1.13 | 12.63 | 5.39 | 13.1 | 42.6 | 120 | - | - | - | - | - | - |
| 10 | **REAB** | SnaFab | AHV | DIS | DIS | DIS | DIS | DIS | DIS | DIS | DIS | DIS | DIS | DIS | DIS | DIS | DIS |
| 11 | **ALSE** | SnaFab | MAS | DIS | DIS | DIS | DIS | DIS | DIS | DIS | DIS | DIS | DIS | DIS | DIS | DIS | DIS |
| 12 | **BAEB** | SnaFab | URM | DIS | DIS | DIS | DIS | DIS | DIS | DIS | DIS | DIS | DIS | DIS | DIS | DIS | DIS |
| 13 | **FAMA** | SnaFab | AHV | 12.9 | 24 | 1.09 | - | - | - | - | - | - | - | - | - | - | - |
| 14 | **ALHA** | SnaFab | AHV | DIS | DIS | DIS | DIS | DIS | DIS | DIS | DIS | DIS | DIS | DIS | DIS | DIS | DIS |
| 15 | **SASH** | SnaFab | AHV | DIS | DIS | DIS | DIS | DIS | DIS | DIS | DIS | DIS | DIS | DIS | DIS | DIS | DIS |
| 16 | **EEZ9** | SnaFab | MAS | DIS | DIS | DIS | DIS | DIS | DIS | DIS | DIS | DIS | DIS | DIS | DIS | DIS | DIS |
| 17 | **DDH8** | Razi | MAS | 13.2 | 24 | 1.16 | 6.4 | 3.54 | 10.8 | 31.2 | 172 | - | - | - | - | - | - |
| 18 | **QQG5** | Razi | MAS | DIS | DIS | DIS | DIS | DIS | DIS | DIS | DIS | DIS | DIS | DIS | DIS | DIS | DIS |
| 19 | **XXA1** | SnaFab | MAS | DIS | DIS | DIS | DIS | DIS | DIS | DIS | DIS | DIS | DIS | DIS | DIS | DIS | DIS |
| 20 | **SSV7** | Razi | MAS | DIS | DIS | DIS | DIS | DIS | DIS | DIS | DIS | DIS | DIS | DIS | DIS | DIS | DIS |
| 21 | **BBM6** | Razi | MAS | DIS | DIS | DIS | DIS | DIS | DIS | DIS | DIS | DIS | DIS | DIS | DIS | DIS | DIS |
| 22 | **YYY4** | SnaFab | MAS | DIS | DIS | DIS | DIS | DIS | DIS | DIS | DIS | DIS | DIS | DIS | DIS | DIS | DIS |
| 23 | **ZZI1** | SnaFab | URM | DIS | DIS | DIS | DIS | DIS | DIS | DIS | DIS | DIS | DIS | DIS | DIS | DIS | DIS |
| 24 | **SSU8** | SnaFab | MAS | DIS | DIS | DIS | DIS | DIS | DIS | DIS | DIS | DIS | DIS | DIS | DIS | DIS | DIS |
| 25 | **NNZ3** | Razi | URM | DIS | DIS | DIS | DIS | DIS | DIS | DIS | DIS | DIS | DIS | DIS | DIS | DIS | DIS |
| 26 | **FFG3** | SnaFab | AHV | 12.4 | 32 | 1.04 | - | - | - | - | - | - | - | - | - | - | - |
| 27 | **IIE0** | Razi | AHV | 15.2 | 40 | 1.31 | 12.5 | 5.32 | 16.3 | 48 | 231 | 18/0.8 | 136/3.5 | - | 17/-/- | 1.3/0.3 | - |
| 28 | **EEG0** | Razi | AHV | DIS | DIS | DIS | DIS | DIS | DIS | DIS | DIS | DIS | DIS | DIS | DIS | DIS | DIS |
| 29 | **JJW4** | SnaFab | AHV | DIS | DIS | DIS | DIS | DIS | DIS | DIS | DIS | DIS | DIS | DIS | DIS | DIS | DIS |
| 30 | **BBI6** | SnaFab | AHV | DIS | DIS | DIS | DIS | DIS | DIS | DIS | DIS | DIS | DIS | DIS | DIS | DIS | DIS |
| 31 | **AAH7** | SnaFab | AHV | 14 | 37 | 1.3 | 8.4 | 5.8 | 16.1 | 49.1 | 212 | - | - | - | - | - | - |
| 32 | **AAF9** | Razi | AHV | DIS | DIS | DIS | DIS | DIS | DIS | DIS | DIS | DIS | DIS | DIS | DIS | DIS | DIS |
| 33 | **WWR1** | Razi | AHV | DIS | DIS | DIS | DIS | DIS | DIS | DIS | DIS | DIS | DIS | DIS | DIS | DIS | DIS |
| 34 | **RRS3** | SnaFab | AHV | DIS | DIS | DIS | DIS | DIS | DIS | DIS | DIS | DIS | DIS | DIS | DIS | DIS | DIS |
| 35 | **JJI8** | Razi | AHV | 13 | 28 | 1.1 | - | - | - | - | - | - | - | - | - | - | - |
| 36 | **QQW3** | SnaFab | AHV | DIS | DIS | DIS | DIS | DIS | DIS | DIS | DIS | DIS | DIS | DIS | DIS | DIS | DIS |
| 37 | **AAT1** | SnaFab | MAS | DIS | DIS | DIS | DIS | DIS | DIS | DIS | DIS | DIS | DIS | DIS | DIS | DIS | DIS |
| 38 | **VVI7** | Razi | AHV | 12 | 35 | 1 | 18.9 | 5 | 14.6 | 45.4 | 190 | - | - | - | - | - | - |
| 39 | **UUL5** | SnaFab | AHV | DIS | DIS | DIS | DIS | DIS | DIS | DIS | DIS | DIS | DIS | DIS | DIS | DIS | DIS |
| 40 | **QQQ3** | Razi | AHV | DIS | DIS | DIS | DIS | DIS | DIS | DIS | DIS | DIS | DIS | DIS | DIS | DIS | DIS |
| 41 | **UUN5** | Razi | AHV | 15 | 31 | 1.4 | 8.6 | 4.68 | 13.4 | 39.8 | 238 | - | -/3.3 | - | - | 0.7/- | - |
| 42 | **XXN0** | SnaFab | AHV | DIS | DIS | DIS | DIS | DIS | DIS | DIS | DIS | DIS | DIS | DIS | DIS | DIS | DIS |
| 43 | **RRA4** | Razi | AHV | DIS | DIS | DIS | DIS | DIS | DIS | DIS | DIS | DIS | DIS | DIS | DIS | DIS | DIS |
| 44 | **DDN5** | SnaFab | AHV | DIS | DIS | DIS | DIS | DIS | DIS | DIS | DIS | DIS | DIS | DIS | DIS | DIS | DIS |
| 45 | **OOZ0** | Razi | AHV | DIS | DIS | DIS | DIS | DIS | DIS | DIS | DIS | DIS | DIS | DIS | DIS | DIS | DIS |
| 46 | **UUT4** | SnaFab | AHV | 13 | 25 | 1.1 | - | - | - | - | - | - | - | - | - | - | - |
| 47 | **GGM9** | SnaFab | AHV | 13 | 33 | 1.1 | - | - | - | - | - | - | - | - | - | - | - |
| 48 | **SSH4** | SnaFab | AHV | DIS | DIS | DIS | DIS | DIS | DIS | DIS | DIS | DIS | DIS | DIS | DIS | DIS | DIS |
| 49 | **FFW7** | Razi | AHV | 14 | 32 | 1.3 | 11.4 | 4.58 | 13.8 | 41.1 | 196 | 15/1.2 | 135/3.7 | - | - | - | - |
| 50 | **MMM9** | Razi | AHV | 12 | 25 | 1 | 12.6 | 4.96 | 14.1 | 42.1 | 206 | 17/1 | 145/3.8 | - | - | - | - |
| 51 | **KKZ6** | SnaFab | AHV | DIS | DIS | DIS | DIS | DIS | DIS | DIS | DIS | DIS | DIS | DIS | DIS | DIS | DIS |
| 52 | **KKW6** | Razi | AHV | DIS | DIS | DIS | DIS | DIS | DIS | DIS | DIS | DIS | DIS | DIS | DIS | DIS | DIS |
| 53 | **EEP5** | Razi | AHV | DIS | DIS | DIS | DIS | DIS | DIS | DIS | DIS | DIS | DIS | DIS | DIS | DIS | DIS |
| 54 | **FFR9** | SnaFab | AHV | DIS | DIS | DIS | DIS | DIS | DIS | DIS | DIS | DIS | DIS | DIS | DIS | DIS | DIS |
| 55 | **UUN6** | Razi | AHV | DIS | DIS | DIS | DIS | DIS | DIS | DIS | DIS | DIS | DIS | DIS | DIS | DIS | DIS |
| 56 | **BBP7** | SnaFab | MAS | DIS | DIS | DIS | DIS | DIS | DIS | DIS | DIS | DIS | DIS | DIS | DIS | DIS | DIS |
| 57 | **OOE4** | Razi | AHV | DIS | DIS | DIS | DIS | DIS | DIS | DIS | DIS | DIS | DIS | DIS | DIS | DIS | DIS |
| 58 | **QQA5** | SnaFab | AHV | DIS | DIS | DIS | DIS | DIS | DIS | DIS | DIS | DIS | DIS | DIS | DIS | DIS | DIS |
| 59 | **FFT8** | Razi | MAS | DIS | DIS | DIS | DIS | DIS | DIS | DIS | DIS | DIS | DIS | DIS | DIS | DIS | DIS |
| 60 | **JJY3** | SnaFab | AHV | 14.3 | 25 | 1.2 | DIS | DIS | DIS | DIS | DIS | DIS | DIS | DIS | DIS | DIS | DIS |
| 61 | **GGN0** | Razi | AHV | - | - | - | - | - | - | - | - | - | - | - | - | - | N |
| 62 | **ZZQ0** | SnaFab | AHV | 12 | 33 | 1 | - | - | - | - | - | - | - | - | - | - | N |
| 63 | **JJK9** | Razi | AHV | - | - | - | - | - | - | - | - | 13/0.9 | 136/4.1 | - | - | - | N |
| 64 | **YYE2** | SnaFab | AHV | DIS | DIS | DIS | DIS | DIS | DIS | DIS | DIS | DIS | DIS | DIS | DIS | DIS | DIS |
| 65 | **TTJ2** | Razi | MAS | DIS | DIS | DIS | DIS | DIS | DIS | DIS | DIS | DIS | DIS | DIS | DIS | DIS | DIS |
| 66 | **GG0** | SnaFab | AHV | 14 | 28 | 1.3 | - | - | - | - | - | - | - | - | - | - | N |
| 67 | **NN5** | Razi | AHV | DIS | DIS | DIS | DIS | DIS | DIS | DIS | DIS | DIS | DIS | DIS | DIS | DIS | DIS |
| 68 | **JX8** | Razi | AHV | DIS | DIS | DIS | DIS | DIS | DIS | DIS | DIS | DIS | DIS | DIS | DIS | DIS | DIS |
| 69 | **QU4** | Razi | AHV | DIS | DIS | DIS | DIS | DIS | DIS | DIS | DIS | DIS | DIS | DIS | DIS | DIS | DIS |
| 70 | **EEJ8** | Razi | URM | 13 | 29 | 1.1 | 8.3 | - | 14.8 | - | - | 26/0.7 | 144/4 | 205 | 13/15/253 | 0.7/0.3 | - |
| 71 | **WT3** | Razi | AHV | DIS | DIS | DIS | DIS | DIS | DIS | DIS | DIS | DIS | DIS | DIS | DIS | DIS | DIS |
| 72 | **DC7** | SnaFab | AHV | DIS | DIS | DIS | DIS | DIS | DIS | DIS | DIS | DIS | DIS | DIS | DIS | DIS | DIS |
| 73 | **BK8** | Razi | AHV | 12 | 47 | 1 | 10.78 | 5.4 | 15.2 | 45.3 | 145 | - | - | - | - | - | N |
| 74 | **ZK8** | Razi | AHV | 15 | 35 | 1.4 | 8.3 | 4.09 | 10.7 | 30.6 | 173 | - | - | - | - | - | N |
| 75 | **FM4** | Razi | AHV | DIS | DIS | DIS | DIS | DIS | DIS | DIS | DIS | DIS | DIS | DIS | DIS | DIS | DIS |
| 76 | **YP2** | SnaFab | AHV | DIS | DIS | DIS | DIS | DIS | DIS | DIS | DIS | DIS | DIS | DIS | DIS | DIS | DIS |
| 77 | **GK9** | Razi | AHV | DIS | DIS | DIS | DIS | DIS | DIS | DIS | DIS | DIS | DIS | DIS | DIS | DIS | DIS |
| 78 | **FC3** | Razi | AHV | DIS | DIS | DIS | DIS | DIS | DIS | DIS | DIS | DIS | DIS | DIS | DIS | DIS | DIS |
| 79 | **UT8** | Razi | AHV | 14 | 28 | 1.3 | 12.9 | 4.39 | 13.3 | 39.2 | 236 | 5/0.9 | 142/3.3 | - | - | - | N |
| 80 | **QS6** | Razi | AHV | 14 | 30 | 1.3 | 9.2 | 3.99 | 11.5 | 33.5 | 224 | - | - | - | - | - | N |
| 81 | **RL9** | Razi | AHV | DIS | DIS | DIS | DIS | DIS | DIS | DIS | DIS | DIS | DIS | DIS | DIS | DIS | DIS |
| 82 | **YF3** | SnaFab | AHV | DIS | DIS | DIS | DIS | DIS | DIS | DIS | DIS | DIS | DIS | DIS | DIS | DIS | DIS |
| 83 | **NNK8** | SnaFab | MAS | DIS | DIS | DIS | DIS | DIS | DIS | DIS | DIS | DIS | DIS | DIS | DIS | DIS | DIS |
| 84 | **KL5** | Razi | AHV | DIS | DIS | DIS | DIS | DIS | DIS | DIS | DIS | DIS | DIS | DIS | DIS | DIS | DIS |
| 85 | **RU5** | SnaFab | AHV | DIS | DIS | DIS | DIS | DIS | DIS | DIS | DIS | DIS | DIS | DIS | DIS | DIS | DIS |
| 86 | **OG6** | Razi | AHV | DIS | DIS | DIS | DIS | DIS | DIS | DIS | DIS | DIS | DIS | DIS | DIS | DIS | DIS |
| 87 | **RU0** | Razi | AHV | DIS | DIS | DIS | DIS | DIS | DIS | DIS | DIS | DIS | DIS | DIS | DIS | DIS | DIS |
| 88 | **CH8** | Razi | AHV | - | - | - | - | - | - | - | - | - | - | - | - | - | N |
| 89 | **VL2** | SnaFab | AHV | DIS | DIS | DIS | DIS | DIS | DIS | DIS | DIS | DIS | DIS | DIS | DIS | DIS | DIS |
| 90 | **DC8** | Razi | AHV | 12.5 | 32 | 1 | 4.4 | 5.12 | 13.5 | 41.1 | 213 | 5/0.6 | - | - | - | - | N |
| 91 | HV6 | Razi | AHV | DIS | DIS | DIS | DIS | DIS | DIS | DIS | DIS | DIS | DIS | DIS | DIS | DIS | DIS |
| 92 | FL2 | Razi | AHV | DIS | DIS | DIS | DIS | DIS | DIS | DIS | DIS | DIS | DIS | DIS | DIS | DIS | DIS |
| 93 | RS1 | SnaFab | AHV | - | - | - | - | - | - | - | - | - | - | - | - | - | N |
| 94 | AJ6 | Razi | AHV | DIS | DIS | DIS | DIS | DIS | DIS | DIS | DIS | DIS | DIS | DIS | DIS | DIS | DIS |
| 95 | ZY8 | Razi | AHV | DIS | DIS | DIS | DIS | DIS | DIS | DIS | DIS | DIS | DIS | DIS | DIS | DIS | DIS |
| 96 | YB6 | SnaFab | AHV | DIS | DIS | DIS | DIS | DIS | DIS | DIS | DIS | DIS | DIS | DIS | DIS | DIS | DIS |
| 97 | GS2 | Razi | AHV | DIS | DIS | DIS | DIS | DIS | DIS | DIS | DIS | DIS | DIS | DIS | DIS | DIS | DIS |
| 98 | WZ7 | Razi | AHV | DIS | DIS | DIS | DIS | DIS | DIS | DIS | DIS | DIS | DIS | DIS | DIS | DIS | DIS |

DIS: Discharged, N= normal, * = result distrupted

## Table 14 - PT tests of victims categorized by visits

| Result | Visit 8 | Visit 7 | Visit 6 | Visit 5 | Visit 4 | Visit 1 | Group | Center | Code | ID |
| --- | --- | --- | --- | --- | --- | --- | --- | --- | --- | --- |
| Recovered | 16 | 17 | 15 | 13.7 | 16 | 36* | SnaFab | AHV | **YUAB** | 1 |
| - | DIS | - | - | - | - | - | SnaFab | AHV | **MAGH** | 2 |
| - | DIS | DIS | DIS | - | DIS | - | SnaFab | AHV | **ALSA** | 3 |
| Recovered | 13.7 | 13.9 | 12.8 | - | 25.1 | 36* | SnaFab | AHV | **GHMA** | 4 |
| Recovered | DIS | DIS | DIS | - | - | 23.7 | SnaFab | AHV | **ABAR** | 5 |
| - | DIS | DIS | - | - | - | - | SnaFab | AHV | **HABA** | 6 |
| - | DIS | - | - | - | 18 | - | SnaFab | AHV | **FASO** | 7 |
| Recovered | 16.9 | 16.9 | 17.8 | 14 | 20.4 | 13.7 | SnaFab | AHV | **RABA** | 8 |
| - | 13.3 | 12.2 | 13.7 | 14 | 14.7 | 13.3 | SnaFab | AHV | **HAHO** | 9 |
| - | DIS | DIS | - | 36* | - | 36 | SnaFab | AHV | **REAB** | 10 |
| Recovered | DIS | 12.6 | 12.9 | - | 15 | 13.3 | SnaFab | MAS | **ALSE** | 11 |
| - | DIS | DIS | 13 | - | - | 12 | SnaFab | URM | **BAEB** | 12 |
| - | 12.9 | 16 | 17.0 | - | 18 | 15 | SnaFab | AHV | **FAMA** | 13 |
| - | DIS | DIS | - | - | - | 14.7 | SnaFab | AHV | **ALHA** | 14 |
| Recovered | DIS | 19.7 | 18.7 | 22 | 17.2 | 36* | SnaFab | AHV | **SASH** | 15 |
| - | DIS | 14.1 | 14.3 | - | 16.1 | 15.8 | SnaFab | MAS | **EEZ9** | 16 |
| - | 13.2 | 13.5 | 11.6 | 11.6 | 13.2 | 13.8 | Razi | MAS | **DDH8** | 17 |
| - | DIS | 13.2 | 13.6 | - | 12.9 | 13 | Razi | MAS | **QQG5** | 18 |
| - | DIS | DIS | 12.5 | - | 13.4 | 13.4 | SnaFab | MAS | **XXA1** | 19 |
| - | DIS | DIS | 11.4 | - | 11.1 | 11.9 | Razi | MAS | **SSV7** | 20 |
| - | DIS | DIS | - | - | 14.3 | 20.4 | Razi | MAS | **BBM6** | 21 |
| Recovered | DIS | DIS | - | - | 12.9 | 33.2 | SnaFab | MAS | **YYY4** | 22 |
| - | DIS | - | 17 | - | - | 13.5 | SnaFab | URM | **ZZI1** | 23 |
| - | DIS | DIS | 13.4 | - | 13.1 | 13.3 | SnaFab | MAS | **SSU8** | 24 |
| - | DIS | DIS | - | - | 13 | 13 | Razi | URM | **NNZ3** | 25 |
| Recovered | 12.4 | 17 | 16 | - | 37* | 108 | SnaFab | AHV | **FFG3** | 26 |
| Not Recovered | 15.2 | 28 | 12 | 14.3 | 36* | 36* | Razi | AHV | **IIE0** | 27 |
| - | DIS | 14 | 15 | 15 | 26 | 36* | Razi | AHV | **EEG0** | 28 |
| - | DIS | 15 | 13 | - | 14.8 | 14 | SnaFab | AHV | **JJW4** | 29 |
| Recovered | DIS | 15 | 19 | 28 | 37 | 36* | SnaFab | AHV | **BBI6** | 30 |
| Recovered | 14 | 14 | 18 | - | 12 | 36* | SnaFab | AHV | **AAH7** | 31 |
| Recovered | DIS | DIS | 15 | 14 | 16 | 21 | Razi | AHV | **AAF9** | 32 |
| - | DIS | 14 | 12.4 | - | 13 | 13 | Razi | AHV | **WWR1** | 33 |
| - | DIS | DIS | 14 | 17 | 18 | 12.4 | SnaFab | AHV | **RRS3** | 34 |
| Recovered | 13 | 13 | 13 | - | 15 | 37* | Razi | AHV | **JJI8** | 35 |
| Recovered | DIS | 18 | 15 | 14 | 12 | 36* | SnaFab | AHV | **QQW3** | 36 |
| - | DIS | DIS | - | - | 11.5 | 12 | SnaFab | MAS | **AAT1** | 37 |
| Recovered | 12 | 13 | 21 | 22 | 16 | * | Razi | AHV | **VVI7** | 38 |
| Recovered | DIS | 16 | 14 | - | 37 | 14 | SnaFab | AHV | **UUL5** | 39 |
| Recovered | DIS | 14 | 16 | 12.9 | 21 | 18 | Razi | AHV | **QQQ3** | 40 |
| - | 15 | 17 | 14 | - | - | 19 | Razi | AHV | **UUN5** | 41 |
| - | DIS | DIS | - | - | - | 12 | SnaFab | AHV | **XXN0** | 42 |
| - | DIS | DIS | - | 16 | 14 | 13 | Razi | AHV | **RRA4** | 43 |
| - | DIS | DIS | - | - | - | 12 | SnaFab | AHV | **DDN5** | 44 |
| Recovered | DIS | 12.3 | 15 | 16 | * | 37* | Razi | AHV | **OOZ0** | 45 |
| Recovered | 13 | 12 | 13 | - | 37 | 36* | SnaFab | AHV | **UUT4** | 46 |
| - | 13 | 14 | 13 | - | 14 | 13 | SnaFab | AHV | **GGM9** | 47 |
| Recovered | DIS | 13.1 | 16 | - | 37* | 16 | SnaFab | AHV | **SSH4** | 48 |
| - | 14 | 15 | 14 | - | 14 | * | Razi | AHV | **FFW7** | 49 |
| Recovered | 12 | 14 | 13 | 12.4 | 16 | 37 | Razi | AHV | **MMM9** | 50 |
| - | DIS | 13 | 14 | - | 13 | 13 | SnaFab | AHV | **KKZ6** | 51 |
| Recovered | DIS | DIS | 13 | 14 | 25 | 14 | Razi | AHV | **KKW6** | 52 |
| - | DIS | DIS | - | - | - | 12 | Razi | AHV | **EEP5** | 53 |
| - | DIS | 13 | 14 | 15 | - | 16 | SnaFab | AHV | **FFR9** | 54 |
| Recovered | DIS | 13 | 14 | 16.5 | 60* | - | Razi | AHV | **UUN6** | 55 |
| - | DIS | DIS | 13.2 | - | - | 11 | SnaFab | MAS | **BBP7** | 56 |
| - | DIS | DIS | 13 | - | 13 | 12 | Razi | AHV | **OOE4** | 57 |
| Recovered | DIS | DIS | 15 | 15 | 36 | 36* | SnaFab | AHV | **QQA5** | 58 |
| Recovered | DIS | 11 | 14.3 | - | 17 | 12 | Razi | MAS | **FFT8** | 59 |
| Recovered | 14.3 | 12 | 15 | 20 | 12 | 13 | SnaFab | AHV | **JJY3** | 60 |
| - | - | - | 13 | - | - | 12 | Razi | AHV | **GGN0** | 61 |
| Recovered | 12 | - | 14 | - | 36 | 36 | SnaFab | AHV | **ZZQ0** | 62 |
| - | - | 14 | 14 | - | 12 | - | Razi | AHV | **JJK9** | 63 |
| - | DIS | DIS | - | - | - | 12 | SnaFab | AHV | **YYE2** | 64 |
| - | - | - | 12 | - | 12.7 | 11.4 | Razi | MAS | **TTJ2** | 65 |
| Recovered | 14 | 14 | 13 | - | 36* | 37 | SnaFab | AHV | **GG0** | 66 |
| Recovered | DIS | 12.8 | 13.3 | - | - | 36 | Razi | AHV | **NN5** | 67 |
| - | DIS | DIS | 12 | - | 12 | 14 | Razi | AHV | **JX8** | 68 |
| Recovered | DIS | DIS | DIS | - | 14.9 | 37 | Razi | AHV | **QU4** | 69 |
| - | 13 | 13 | 13 | - | 13 | 13 | Razi | URM | **EEJ8** | 70 |
| Recovered | DIS | DIS | 14 | - | 13 | 38 | Razi | AHV | **WT3** | 71 |
| - | DIS | DIS | DIS | - | - | 12 | SnaFab | AHV | **DC7** | 72 |
| - | 12 | 14 | 17 | - | - | 13 | Razi | AHV | **BK8** | 73 |
| Not Recovered | 15 | 27 | 15 | 36 | - | 36 | Razi | AHV | **ZK8** | 74 |
| Recovered | DIS | DIS | 14.4 | 19 | 36 | 36 | Razi | AHV | **FM4** | 75 |
| Recovered | DIS | 12 | 14.1 | 24 | 36 | 37 | SnaFab | AHV | **YP2** | 76 |
| - | DIS | DIS | DIS | - | 12 | 12 | Razi | AHV | **GK9** | 77 |
| - | DIS | 12 | 13 | - | 13.3 | 12 | Razi | AHV | **FC3** | 78 |
| Recovered | 14 | 14 | 14 | 16.7 | 17 | 60 | Razi | AHV | **UT8** | 79 |
| Recovered | 14 | 15 | 14 | 19 | 23 | 37* | Razi | AHV | **QS6** | 80 |
| - | DIS | 13 | 14 | - | 15.6 | 17 | Razi | AHV | **RL9** | 81 |
| - | DIS | DIS | 17 | - | 36 | 15 | SnaFab | AHV | **YF3** | 82 |
| - | - | - | - | - | 8.9 | - | SnaFab | MAS | **NNK8** | 83 |
| - | DIS | 13.6 | 13 | - | 13 | 13.1 | Razi | AHV | **KL5** | 84 |
| - | DIS | DIS | 13 | - | 13.8 | 14 | SnaFab | AHV | **RU5** | 85 |
| - | DIS | DIS | 12 | - | 16 | 14 | Razi | AHV | **OG6** | 86 |
| Recovered | DIS | 16 | 18 | 25 | 36 | 12 | Razi | AHV | **RU0** | 87 |
| Recovered | - | 13 | - | 17.5 | 60* | 23 | Razi | AHV | **CH8** | 88 |
| - | - | - | DIS | - | - | 12 | SnaFab | AHV | **VL2** | 89 |
| Recovered | 12.5 | 13.6 | 12.7 | 15.4 | - | 60 | Razi | AHV | **DC8** | 90 |
| - | DIS | 14 | 13 | - | 12.3 | 15 | Razi | AHV | **HV6** | 91 |
| - | DIS | DIS | DIS | 12 | 14.7 | 13 | Razi | AHV | **FL2** | 92 |
| - | - | - | - | - | - | 36 | SnaFab | AHV | **RS1** | 93 |
| - | DIS | DIS | 14 | - | 14 | 12 | Razi | AHV | **AJ6** | 94 |
| - | DIS | 15.5 | 14 | 12 | 17.6 | 13 | Razi | AHV | **ZY8** | 95 |
| - | DIS | - | DIS | - | 12 | 12 | SnaFab | AHV | **YB6** | 96 |
| - | 13 | 13 | 13 | - | 12 | 12.9 | Razi | AHV | **GS2** | 97 |
| Not Recovered | DIS | DIS | DIS | 36* | 36* | 37* | Razi | AHV | **WZ7** | 98 |

DIS: Discharged, N= normal, * = result disrupted

## Table 15 - PTT tests of victims categorized by visits

| Result | Visit 8 | Visit 7 | Visit 6 | Visit 5 | Visit 4 | Visit 1 | Group | Center | Code | ID |
| --- | --- | --- | --- | --- | --- | --- | --- | --- | --- | --- |
| - | 29 | 30 | 30 | 1.17 | 37 | 47 | SnaFab | AHV | **YUAB** | 1 |
| - | DIS | - | - | - | - | - | SnaFab | AHV | **MAGH** | 2 |
| - | DIS | DIS | DIS | - | DIS | - | SnaFab | AHV | **ALSA** | 3 |
| Recovered | 25 | 31 | 30 | - | 64.6 | 120 | SnaFab | AHV | **GHMA** | 4 |
| - | DIS | DIS | DIS | - | - | 37 | SnaFab | AHV | **ABAR** | 5 |
| - | DIS | DIS | - | - | - | - | SnaFab | AHV | **HABA** | 6 |
| Recovered | DIS | - | - | - | 55 | - | SnaFab | AHV | **FASO** | 7 |
| Recovered | 28 | 33 | 39 | 27 | 55 | 47 | SnaFab | AHV | **RABA** | 8 |
| Recovered | 36 | 24 | 38 | 27 | 39 | 37 | SnaFab | AHV | **HAHO** | 9 |
| - | DIS | DIS | - | 121* | - | 121 | SnaFab | AHV | **REAB** | 10 |
| - | DIS | 30 | 28.7 | - | 30 | 28.5 | SnaFab | MAS | **ALSE** | 11 |
| - | DIS | DIS | 25.3 | - | - | 28 | SnaFab | URM | **BAEB** | 12 |
| - | 24 | 30 | 25 | - | 37 | 25 | SnaFab | AHV | **FAMA** | 13 |
| - | DIS | DIS | - | - | - | 37 | SnaFab | AHV | **ALHA** | 14 |
| Recovered | DIS | 37 | 39 | 35 | 31 | 54 | SnaFab | AHV | **SASH** | 15 |
| - | DIS | 30 | 28.3 | - | 27.1 | 45.4 | SnaFab | MAS | **EEZ9** | 16 |
| - | 24 | 33 | 25.1 | 25.1 | 27 | 29.9 | Razi | MAS | **DDH8** | 17 |
| - | DIS | 30 | 30 | - | 30 | 29 | Razi | MAS | **QQG5** | 18 |
| - | DIS | DIS | 33.2 | - | 38.9 | 37.2 | SnaFab | MAS | **XXA1** | 19 |
| - | DIS | DIS | 23.9 | - | 24.2 | 30 | Razi | MAS | **SSV7** | 20 |
| - | DIS | DIS | - | - | 34 | 31.1 | Razi | MAS | **BBM6** | 21 |
| - | DIS | DIS | - | - | 28 | 28.9 | SnaFab | MAS | **YYY4** | 22 |
| - | DIS | - | 34 | - | - | 28 | SnaFab | URM | **ZZI1** | 23 |
| - | DIS | DIS | 31 | - | 32 | 29.9 | SnaFab | MAS | **SSU8** | 24 |
| - | DIS | DIS | - | - | 38 | 34.5 | Razi | URM | **NNZ3** | 25 |
| Recovered | 32 | 25 | 30 | - | 42 | 121* | SnaFab | AHV | **FFG3** | 26 |
| Not Recovered | 40 | 100 | 48 | 35 | 120* | 120* | Razi | AHV | **IIE0** | 27 |
| Recovered | DIS | 36 | 34 | 35 | 36 | 121* | Razi | AHV | **EEG0** | 28 |
| - | DIS | 35 | 30 | - | 25 | 25 | SnaFab | AHV | **JJW4** | 29 |
| Recovered | DIS | 31 | 31 | 39 | 66 | 121* | SnaFab | AHV | **BBI6** | 30 |
| Recovered | 37 | 25 | 30 | - | 121* | 120* | SnaFab | AHV | **AAH7** | 31 |
| - | DIS | DIS | 33 | 28 | 30 | 32 | Razi | AHV | **AAF9** | 32 |
| - | DIS | 30 | 32 | - | 30 | 25 | Razi | AHV | **WWR1** | 33 |
| - | DIS | DIS | 27 | 30 | 25 | 29 | SnaFab | AHV | **RRS3** | 34 |
| - | 28 | 25 | 29 | - | 31 | 41 | Razi | AHV | **JJI8** | 35 |
| Recovered | DIS | 32 | 35 | 31 | 25 | 121* | SnaFab | AHV | **QQW3** | 36 |
| - | DIS | DIS | - | - | 27 | 29 | SnaFab | MAS | **AAT1** | 37 |
| - | 35 | 28 | 30 | 44 | 33 | * | Razi | AHV | **VVI7** | 38 |
| Recovered | DIS | 25 | 27 | - | 121 | 121 | SnaFab | AHV | **UUL5** | 39 |
| - | DIS | 25 | 34 | 38 | 36 | 32 | Razi | AHV | **QQQ3** | 40 |
| - | 31 | 30 | 34 | - | - | 37 | Razi | AHV | **UUN5** | 41 |
| - | DIS | DIS | - | - | - | 25 | SnaFab | AHV | **XXN0** | 42 |
| - | DIS | DIS | - | 25 | 37 | 25 | Razi | AHV | **RRA4** | 43 |
| - | DIS | DIS | - | - | - | 26 | SnaFab | AHV | **DDN5** | 44 |
| Recovered | DIS | 25 | 25 | 35 | * | 121* | Razi | AHV | **OOZ0** | 45 |
| Recovered | 25 | 25 | 25 | - | 121 | 121* | SnaFab | AHV | **UUT4** | 46 |
| - | 33 | 27 | 25 | - | 32 | 25 | SnaFab | AHV | **GGM9** | 47 |
| Recovered | DIS | 24 | 33 | - | 55* | 40 | SnaFab | AHV | **SSH4** | 48 |
| - | 32 | 31 | 35 | - | 28 | * | Razi | AHV | **FFW7** | 49 |
| Recovered | 25 | 45 | 32 | 25 | 34 | 50 | Razi | AHV | **MMM9** | 50 |
| - | DIS | 25 | 25 | - | 25 | 25 | SnaFab | AHV | **KKZ6** | 51 |
| Recovered | DIS | DIS | 27 | 27 | 50 | 121 | Razi | AHV | **KKW6** | 52 |
| - | DIS | DIS | - | - | - | 24 | Razi | AHV | **EEP5** | 53 |
| - | DIS | 39 | 43 | 38 | - | 30 | SnaFab | AHV | **FFR9** | 54 |
| Recovered | DIS | 26 | 29 | 22 | 120* | - | Razi | AHV | **UUN6** | 55 |
| - | DIS | DIS | 32 | - | - | 30 | SnaFab | MAS | **BBP7** | 56 |
| - | DIS | DIS | 32 | - | 32 | 32 | Razi | AHV | **OOE4** | 57 |
| Recovered | DIS | DIS | 34 | 27 | 121 | 121* | SnaFab | AHV | **QQA5** | 58 |
| - | DIS | 40 | 32.6 | - | 25.5 | 23 | Razi | MAS | **FFT8** | 59 |
| - | 25 | 39 | 30 | 34 | 50 | 25 | SnaFab | AHV | **JJY3** | 60 |
| - | - | - | 35 | - | - | 24 | Razi | AHV | **GGN0** | 61 |
| Recovered | 33 | - | 26 | - | 65 | 121 | SnaFab | AHV | **ZZQ0** | 62 |
| - | - | 32 | 30 | - | 43 | - | Razi | AHV | **JJK9** | 63 |
| - | DIS | DIS | 24 | - | - | 25 | SnaFab | AHV | **YYE2** | 64 |
| - | - | - | 30 | - | 44 | 47 | Razi | MAS | **TTJ2** | 65 |
| Recovered | 28 | 28 | 27 | - | 121* | 121 | SnaFab | AHV | **GG0** | 66 |
| - | DIS | 26 | 33 | - | - | 32 | Razi | AHV | **NN5** | 67 |
| - | DIS | DIS | 28 | - | 24 | 34 | Razi | AHV | **JX8** | 68 |
| Recovered | DIS | DIS | DIS | - | 46 | 121 | Razi | AHV | **QU4** | 69 |
| - | 29 | 31 | 35 | - | 42 | 40 | Razi | URM | **EEJ8** | 70 |
| Recovered | DIS | DIS | 35 | - | 25 | 120 | Razi | AHV | **WT3** | 71 |
| - | DIS | DIS | DIS | - | - | 27 | SnaFab | AHV | **DC7** | 72 |
| - | 47 | 33 | 55 | - | - | 38 | Razi | AHV | **BK8** | 73 |
| Not Recovered | 35 | 72 | 50 | 121 | - | 121 | Razi | AHV | **ZK8** | 74 |
| Recovered | DIS | DIS | 46 | 90 | 121 | 120 | Razi | AHV | **FM4** | 75 |
| Recovered | DIS | 33 | 36 | 34 | 121 | 121 | SnaFab | AHV | **YP2** | 76 |
| - | DIS | DIS | DIS | - | 35 | 35 | Razi | AHV | **GK9** | 77 |
| - | DIS | 24 | 25 | - | 28 | 25 | Razi | AHV | **FC3** | 78 |
| Recovered | 28 | 25 | 30 | 30 | 31 | 120 | Razi | AHV | **UT8** | 79 |
| Recovered | 30 | 33 | 36 | 35 | 60 | 121* | Razi | AHV | **QS6** | 80 |
| Recovered | DIS | 30 | 39 | - | 32 | 120 | Razi | AHV | **RL9** | 81 |
| Recovered | DIS | DIS | 40 | - | 121 | 121 | SnaFab | AHV | **YF3** | 82 |
| - | - | - | - | - | 27.6 | - | SnaFab | MAS | **NNK8** | 83 |
| - | DIS | 24 | 32 | - | 25 | 27 | Razi | AHV | **KL5** | 84 |
| - | DIS | DIS | 28 | - | 24 | 25 | SnaFab | AHV | **RU5** | 85 |
| - | DIS | DIS | 26 | - | 38 | 30 | Razi | AHV | **OG6** | 86 |
| Recovered | DIS | 40 | 32 | 48 | 36* | 121 | Razi | AHV | **RU0** | 87 |
| Recovered | - | 30 | - | 25 | 120* | 28 | Razi | AHV | **CH8** | 88 |
| - | - | - | DIS | - | - | 31 | SnaFab | AHV | **VL2** | 89 |
| Recovered | 32 | 40 | 35 | 35 | - | 120 | Razi | AHV | **DC8** | 90 |
| - | DIS | 38 | 36 | - | 24 | 35 | Razi | AHV | **HV6** | 91 |
| - | DIS | DIS | DIS | 30 | 29 | 28 | Razi | AHV | **FL2** | 92 |
| - | - | - | - | - | - | 121 | SnaFab | AHV | **RS1** | 93 |
| - | DIS | DIS | 31 | - | 30 | 26 | Razi | AHV | **AJ6** | 94 |
| - | DIS | 24 | 35 | 25 | 27 | 31 | Razi | AHV | **ZY8** | 95 |
| - | DIS | - | DIS | - | 41 | 25 | SnaFab | AHV | **YB6** | 96 |
| - | 30 | 30 | 25 | - | 30 | 30 | Razi | AHV | **GS2** | 97 |
| - | DIS | DIS | DIS | 36* | 83 | 121* | Razi | AHV | **WZ7** | 98 |

DIS: Discharged, N= normal, * = result disrupted

## Table 16 - Platelet tests of victims categorized by visits

| Result | Visit 8 | Visit 7 | Visit 6 | Visit 5 | Visit 4 | Visit 1 | Group | Center | Code | ID |
| --- | --- | --- | --- | --- | --- | --- | --- | --- | --- | --- |
| - | - | - | - | - | - | 208 | SnaFab | AHV | **YUAB** | 1 |
| - | DIS | - | - | - | - | - | SnaFab | AHV | **MAGH** | 2 |
| - | DIS | DIS | DIS | - | DIS | - | SnaFab | AHV | **ALSA** | 3 |
| - | 162 | 199 | - | - | 225 | 261 | SnaFab | AHV | **GHMA** | 4 |
| - | DIS | DIS | DIS | - | - | 185 | SnaFab | AHV | **ABAR** | 5 |
| - | DIS | DIS | - | - | - | - | SnaFab | AHV | **HABA** | 6 |
| - | DIS | - | - | - | 183 | - | SnaFab | AHV | **FASO** | 7 |
| - | - | 228 | 252 | - | 256 | 291 | SnaFab | AHV | **RABA** | 8 |
| Not Recovered | 120 | 133 | 163 | - | - | 101 | SnaFab | AHV | **HAHO** | 9 |
| - | DIS | DIS | 161 | - | 163 | 193 | SnaFab | AHV | **REAB** | 10 |
| Recovered | DIS | 198 | 225 | - | 221 | 143 | SnaFab | MAS | **ALSE** | 11 |
| - | DIS | DIS | 211 | - | - | 199 | SnaFab | URM | **BAEB** | 12 |
| - | - | DIS | - | - | - | 266 | SnaFab | AHV | **FAMA** | 13 |
| - | DIS | DIS | - | - | 236 | 247 | SnaFab | AHV | **ALHA** | 14 |
| - | DIS | 188 | - | - | - | 193 | SnaFab | AHV | **SASH** | 15 |
| - | DIS | 71` | 74 | - | 82 | 96 | SnaFab | MAS | **EEZ9** | 16 |
| - | 172 | 166 | 173 | 173 | 193 | 218 | Razi | MAS | **DDH8** | 17 |
| - | DIS | 190 | 178 | - | 180 | 206 | Razi | MAS | **QQG5** | 18 |
| - | DIS | DIS | 235 | - | 233 | 222 | SnaFab | MAS | **XXA1** | 19 |
| - | DIS | DIS | - | - | 152 | 237 | Razi | MAS | **SSV7** | 20 |
| - | DIS | DIS | - | - | 166 | 159 | Razi | MAS | **BBM6** | 21 |
| - | DIS | DIS | 189 | - | 188 | 143 | SnaFab | MAS | **YYY4** | 22 |
| Not Recovered | DIS | 150 | 136 | - | - | 107 | SnaFab | URM | **ZZI1** | 23 |
| - | DIS | DIS | 205 | - | 189 | 181 | SnaFab | MAS | **SSU8** | 24 |
| Recovered | DIS | DIS | - | - | 295 | 128 | Razi | URM | **NNZ3** | 25 |
| - | - | 206 | 220 | - | 227 | 222 | SnaFab | AHV | **FFG3** | 26 |
| - | 231 | 202 | - | - | 229 | 264 | Razi | AHV | **IIE0** | 27 |
| Recovered | DIS | 200 | 202 | - | 125 | 222 | Razi | AHV | **EEG0** | 28 |
| - | DIS | - | - | - | 198 | 165 | SnaFab | AHV | **JJW4** | 29 |
| - | DIS | - | 224 | - | 186 | 177 | SnaFab | AHV | **BBI6** | 30 |
| - | 212 | 221 | - | - | 238 | 234 | SnaFab | AHV | **AAH7** | 31 |
| - | DIS | DIS | 199 | - | - | 193 | Razi | AHV | **AAF9** | 32 |
| Recovered | DIS | - | - | - | 259 | 110 | Razi | AHV | **WWR1** | 33 |
| - | DIS | DIS | - | 271 | 386 | 336 | SnaFab | AHV | **RRS3** | 34 |
| - | - | - | - | - | - | 358 | Razi | AHV | **JJI8** | 35 |
| - | DIS | - | 303 | 314 | 265 | 313 | SnaFab | AHV | **QQW3** | 36 |
| Not Recovered | DIS | DIS | - | - | 141 | 228 | SnaFab | MAS | **AAT1** | 37 |
| - | 190 | 240 | 278 | - | 247 | 236 | Razi | AHV | **VVI7** | 38 |
| - | DIS | 186 | - | 206 | 206 | 237 | SnaFab | AHV | **UUL5** | 39 |
| - | DIS | 303 | 267 | 228 | 285 | 304 | Razi | AHV | **QQQ3** | 40 |
| - | 238 | 220 | 247 | - | - | 262 | Razi | AHV | **UUN5** | 41 |
| - | DIS | DIS | - | - | - | 246 | SnaFab | AHV | **XXN0** | 42 |
| - | DIS | DIS | - | - | - | 279 | Razi | AHV | **RRA4** | 43 |
| - | DIS | DIS | - | - | - | 205 | SnaFab | AHV | **DDN5** | 44 |
| - | DIS | 202 | 156 | - | 166 | 181 | Razi | AHV | **OOZ0** | 45 |
| - | - | - | - | - | 211 | 223 | SnaFab | AHV | **UUT4** | 46 |
| - | - | - | - | - | - | 260 | SnaFab | AHV | **GGM9** | 47 |
| - | DIS | - | 234 | - | 212 | - | SnaFab | AHV | **SSH4** | 48 |
| Recovered | 196 | 183 | 240 | - | - | 103 | Razi | AHV | **FFW7** | 49 |
| - | 206 | - | 169 | - | 196 | 217 | Razi | AHV | **MMM9** | 50 |
| - | DIS | - | - | - | - | 313 | SnaFab | AHV | **KKZ6** | 51 |
| - | DIS | DIS | - | - | - | - | Razi | AHV | **KKW6** | 52 |
| - | DIS | DIS | - | - | - | 190 | Razi | AHV | **EEP5** | 53 |
| - | DIS | - | 219 | - | - | 246 | SnaFab | AHV | **FFR9** | 54 |
| - | DIS | - | - | 319 | 321 | - | Razi | AHV | **UUN6** | 55 |
| - | DIS | DIS | 192 | - | - | 182 | SnaFab | MAS | **BBP7** | 56 |
| - | DIS | DIS | - | - | - | 197 | Razi | AHV | **OOE4** | 57 |
| Not Recovered | DIS | DIS | - | - | 60* | 60* | SnaFab | AHV | **QQA5** | 58 |
| Not Recovered | DIS | - | - | - | 119 | 34 | Razi | MAS | **FFT8** | 59 |
| - | DIS | - | 258 | - | 297 | 301 | SnaFab | AHV | **JJY3** | 60 |
| - | - | - | - | - | - | 287 | Razi | AHV | **GGN0** | 61 |
| Not Recovered | - | 114 | 133 | - | 167 | 129 | SnaFab | AHV | **ZZQ0** | 62 |
| - | - | 217 | - | - | 200 | - | Razi | AHV | **JJK9** | 63 |
| - | DIS | DIS | 240 | - | - | 323 | SnaFab | AHV | **YYE2** | 64 |
| - | - | - | 214 | - | 253 | 262 | Razi | MAS | **TTJ2** | 65 |
| - | - | - | - | - | 314 | 361 | SnaFab | AHV | **GG0** | 66 |
| - | DIS | - | - | - | 189 | 190 | Razi | AHV | **NN5** | 67 |
| - | DIS | DIS | 175 | - | - | 234 | Razi | AHV | **JX8** | 68 |
| - | DIS | DIS | DIS | - | 234 | 259 | Razi | AHV | **QU4** | 69 |
| - | 306 | 306 | 200 | - | 280 | 350 | Razi | URM | **EEJ8** | 70 |
| Not Recovered | DIS | DIS | 139 | - | 145 | 215 | Razi | AHV | **WT3** | 71 |
| - | DIS | DIS | DIS | - | - | 219 | SnaFab | AHV | **DC7** | 72 |
| - | 145 | 174 | 176 | - | - | 180 | Razi | AHV | **BK8** | 73 |
| - | 174 | 210 | 238 | 236 | - | 219 | Razi | AHV | **ZK8** | 74 |
| - | DIS | DIS | 219 | 222 | 258 | 279 | Razi | AHV | **FM4** | 75 |
| - | DIS | 190 | - | - | - | 221 | SnaFab | AHV | **YP2** | 76 |
| - | DIS | DIS | DIS | - | 101 | 259 | Razi | AHV | **GK9** | 77 |
| - | DIS | 239 | - | - | 250 | 264 | Razi | AHV | **FC3** | 78 |
| - | 236 | 274 | 263 | 224 | - | 283 | Razi | AHV | **UT8** | 79 |
| - | 224 | 224 | 205 | 216 | 242 | 290 | Razi | AHV | **QS6** | 80 |
| - | DIS | 318 | 304 | - | 283 | - | Razi | AHV | **RL9** | 81 |
| - | DIS | DIS | 202 | - | 198 | 266 | SnaFab | AHV | **YF3** | 82 |
| - | - | - | - | - | 192 | 174 | SnaFab | MAS | **NNK8** | 83 |
| - | DIS | - | 328 | - | 334 | 357 | Razi | AHV | **KL5** | 84 |
| Recovered | DIS | DIS | 165 | - | 150 | 122 | SnaFab | AHV | **RU5** | 85 |
| - | DIS | DIS | 237 | - | 199 | 238 | Razi | AHV | **OG6** | 86 |
| - | DIS | 156 | 192 | 198 | 184 | 205 | Razi | AHV | **RU0** | 87 |
| - | - | - | 280 | 264 | 274 | 337 | Razi | AHV | **CH8** | 88 |
| - | - | - | DIS | - | - | 330 | SnaFab | AHV | **VL2** | 89 |
| - | 213 | 195 | 198 | - | - | 228 | Razi | AHV | **DC8** | 90 |
| - | DIS | 191 | 242 | - | - | 245 | Razi | AHV | HV6 | 91 |
| Recovered | DIS | DIS | DIS | 213 | 210 | 71 | Razi | AHV | FL2 | 92 |
| - | - | - | - | - | - | 309 | SnaFab | AHV | RS1 | 93 |
| - | DIS | DIS | 259 | - | 223 | 177 | Razi | AHV | AJ6 | 94 |
| - | DIS | 287 | 277 | 247 | 265 | 314 | Razi | AHV | ZY8 | 95 |
| - | DIS | - | DIS | - | 223 | 224 | SnaFab | AHV | YB6 | 96 |
| - | 318 | 318 | 209 | - | 177 | 155 | Razi | AHV | GS2 | 97 |
| - | DIS | DIS | DIS | - | 216 | 270 | Razi | AHV | WZ7 | 98 |

DIS: Discharged, N= normal, * = result disrupted

## Table 17 - INR tests of victims categorized by visits

| Result | Visit 8 | Visit 7 | Visit 6 | Visit 5 | Visit 4 | Visit 1 | Group | Center | Code | ID |
| --- | --- | --- | --- | --- | --- | --- | --- | --- | --- | --- |
| Not Recovered | 1.6 | 1.6 | 1.4 | 29 | 1.6 | 7* | SnaFab | AHV | **YUAB** | 1 |
| - | DIS | DIS | - | - | - | - | SnaFab | AHV | **MAGH** | 2 |
| - | DIS | DIS | DIS | - | DIS | - | SnaFab | AHV | **ALSA** | 3 |
| Recovered | 1.2 | 1.2 | 1.08 | - | 3.9 | 6* | SnaFab | AHV | **GHMA** | 4 |
| Not Recovered | DIS | DIS | DIS | - | - | 3.5 | SnaFab | AHV | **ABAR** | 5 |
| - | DIS | DIS | - | - | - | - | SnaFab | AHV | **HABA** | 6 |
| - | DIS | DIS | - | - | 2.05 | - | SnaFab | AHV | **FASO** | 7 |
| Not Recovered | 1.81 | 1.81 | 2.01 | 1.2 | 2.62 | 1.2 | SnaFab | AHV | **RABA** | 8 |
| Not Recovered | 1.13 | 1.13 | 1.2 | 1.2 | 1.38 | 1.13 | SnaFab | AHV | **HAHO** | 9 |
| Not Recovered | DIS | DIS | - | 6* | - | 6 | SnaFab | AHV | **REAB** | 10 |
| - | DIS | DIS | 1.09 | - | 1.31 | 1.13 | SnaFab | MAS | **ALSE** | 11 |
| - | DIS | DIS | 1 | - | - | 0.88 | SnaFab | URM | **BAEB** | 12 |
| Not Recovered | 1.09 | 1.09 | 1.6 | - | 1.84 | 1.4 | SnaFab | AHV | **FAMA** | 13 |
| - | DIS | DIS | - | - | - | 1.3 | SnaFab | AHV | **ALHA** | 14 |
| Not Recovered | DIS | DIS | 2.21 | 1.6 | 1.5 | 6* | SnaFab | AHV | **SASH** | 15 |
| - | DIS | 1.21 | 1.24 | - | 1.43 | 1.4 | SnaFab | MAS | **EEZ9** | 16 |
| - | DIS | 1.21 | 0.95 | 0.95 | 1.17 | 1.25 | Razi | MAS | **DDH8** | 17 |
| - | DIS | 1.16 | 1.22 | - | 1.12 | 1.14 | Razi | MAS | **QQG5** | 18 |
| - | DIS | DIS | 1.07 | - | 1.19 | 1.19 | SnaFab | MAS | **XXA1** | 19 |
| - | DIS | DIS | 0.92 | - | 0.83 | 0.99 | Razi | MAS | **SSV7** | 20 |
| Recovered | DIS | DIS | - | - | 1.33 | 2.35 | Razi | MAS | **BBM6** | 21 |
| - | DIS | DIS | - | - | 1.12 | 1 | SnaFab | MAS | **YYY4** | 22 |
| - | DIS | DIS | 1.48 | - | - | 1.06 | SnaFab | URM | **ZZI1** | 23 |
| - | DIS | DIS | 1.19 | - | 1.15 | 1.18 | SnaFab | MAS | **SSU8** | 24 |
| - | DIS | DIS | - | - | 1 | 1 | Razi | URM | **NNZ3** | 25 |
| Not Recovered | 1.04 | 1.8 | 1.6 | - | 6* | 11.4 | SnaFab | AHV | **FFG3** | 26 |
| Not Recovered | 1.31 | 4 | 1 | 1.2 | 6* | 6* | Razi | AHV | **IIE0** | 27 |
| Not Recovered | DIS | 1.3 | 1.4 | 1.4 | 3.4 | 6* | Razi | AHV | **EEG0** | 28 |
| Not Recovered | DIS | 1.4 | 1.1 | - | 1.27 | 1.2 | SnaFab | AHV | **JJW4** | 29 |
| Not Recovered | DIS | 1.4 | 2.1 | 4 | 7 | - | SnaFab | AHV | **BBI6** | 30 |
| Not Recovered | 1.3 | 1.3 | 1.9 | - | 1 | 6* | SnaFab | AHV | **AAH7** | 31 |
| Not Recovered | DIS | DIS | 1.4 | 1.2 | 1.5 | 2.5 | Razi | AHV | **AAF9** | 32 |
| - | DIS | 1.2 | 1.04 | - | 1.1 | 1.1 | Razi | AHV | **WWR1** | 33 |
| - | DIS | DIS | 1.3 | 1.8 | 1.9 | 1.04 | SnaFab | AHV | **RRS3** | 34 |
| Recovered | 1.1 | 1.1 | 1.15 | - | 1.4 | 6.1 | Razi | AHV | **JJI8** | 35 |
| Not Recovered | DIS | DIS | 1.4 | 1.3 | 1 | 6* | SnaFab | AHV | **QQW3** | 36 |
| - | DIS | DIS | - | - | 0.93 | 1 | SnaFab | MAS | **AAT1** | 37 |
| Recovered | 1 | 1.1 | 2.6 | 2.7 | 1.6 | * | Razi | AHV | **VVI7** | 38 |
| Not Recovered | DIS | 1.6 | 1.3 | - | 6.1 | 1.2 | SnaFab | AHV | **UUL5** | 39 |
| Not Recovered | DIS | 1.3 | 1.6 | 1.09 | 2.5 | 1.8 | Razi | AHV | **QQQ3** | 40 |
| Not Recovered | 1.4 | 1.8 | 1.2 | - | - | 2.1 | Razi | AHV | **UUN5** | 41 |
| - | DIS | DIS | - | - | - | 1 | SnaFab | AHV | **XXN0** | 42 |
| - | DIS | DIS | - | 1.5 | 1.3 | 1.1 | Razi | AHV | **RRA4** | 43 |
| - | DIS | DIS | - | - | - | 1 | SnaFab | AHV | **DDN5** | 44 |
| Recovered | DIS | 1.03 | 1.4 | 1.5 | * | 6* | Razi | AHV | **OOZ0** | 45 |
| - | 1.1 | 1 | 1.1 | - | - | -* | SnaFab | AHV | **UUT4** | 46 |
| Not Recovered | 1.1 | 1.3 | 1.1 | - | 1.3 | 1.1 | SnaFab | AHV | **GGM9** | 47 |
| Recovered | DIS | 1.1 | 1.6 | - | 7* | 1.6 | SnaFab | AHV | **SSH4** | 48 |
| Not Recovered | 1.3 | 1.4 | 1.3 | - | 1.3 | * | Razi | AHV | **FFW7** | 49 |
| Not Recovered | 1 | 1.3 | 1.1 | 1.04 | 1.6 | 6.1 | Razi | AHV | **MMM9** | 50 |
| Recovered | DIS | 1.1 | 1.3 | - | 1.1 | 1.1 | SnaFab | AHV | **KKZ6** | 51 |
| Recovered | DIS | DIS | 1.1 | 1.2 | 3.3 | 1.3 | Razi | AHV | **KKW6** | 52 |
| - | DIS | DIS | - | - | - | 1 | Razi | AHV | **EEP5** | 53 |
| Recovered | DIS | 1.1 | 1.2 | 1.29 | - | 1.6 | SnaFab | AHV | **FFR9** | 54 |
| Recovered | DIS | 1 | 1.1 | 1.5 | 6* | - | Razi | AHV | **UUN6** | 55 |
| - | DIS | DIS | 1.09 | - | - | 0.9 | SnaFab | MAS | **BBP7** | 56 |
| - | DIS | DIS | 1 | - | 1 | 1 | Razi | AHV | **OOE4** | 57 |
| Not Recovered | DIS | DIS | 1.4 | 1.4 | * | 6* | SnaFab | AHV | **QQA5** | 58 |
| Recovered | DIS | 0.92 | 1.33 | - | 1.75 | 1 | Razi | MAS | **FFT8** | 59 |
| Recovered | 1.2 | 1 | 1.4 | 2.9 | 1 | 1.1 | SnaFab | AHV | **JJY3** | 60 |
| - | DIS | DIS | 1.1 | - | - | 1 | Razi | AHV | **GGN0** | 61 |
| Recovered | 1 | DIS | 1.2 | - | 6 | 6 | SnaFab | AHV | **ZZQ0** | 62 |
| Not Recovered | DIS | 1.3 | 1.2 | - | 1 | - | Razi | AHV | **JJK9** | 63 |
| - | DIS | DIS | - | - | - | 1 | SnaFab | AHV | **YYE2** | 64 |
| - | DIS | DIS | 1.09 | - | 1.15 | 1.04 | Razi | MAS | **TTJ2** | 65 |
| Not Recovered | 1.3 | 1.3 | 1.1 | - | 7* | 6 | SnaFab | AHV | **GG0** | 66 |
| - | DIS | 1.08 | 1.13 | - | - | - | Razi | AHV | **NN5** | 67 |
| Recovered | DIS | DIS | 1 | - | 1 | 1.3 | Razi | AHV | **JX8** | 68 |
| Not Recovered | DIS | DIS | DIS | - | 1.28 | 6 | Razi | AHV | **QU4** | 69 |
| - | 1.1 | 1.1 | 1.1 | - | 1.2 | 1 | Razi | URM | **EEJ8** | 70 |
| Recovered | DIS | DIS | 1.1 | - | 1 | 6 | Razi | AHV | **WT3** | 71 |
| - | DIS | DIS | DIS | - | - | 1 | SnaFab | AHV | **DC7** | 72 |
| Not Recovered | 1 | 1.3 | 1.6 | - | - | 1.1 | Razi | AHV | **BK8** | 73 |
| Not Recovered | 1.4 | 3.8 | 1.4 | 6 | - | 6 | Razi | AHV | **ZK8** | 74 |
| Not Recovered | DIS | DIS | 1.37 | 2 | 6 | 6 | Razi | AHV | **FM4** | 75 |
| Not Recovered | DIS | 1.3 | 1.32 | 3.1 | 6 | 6 | SnaFab | AHV | **YP2** | 76 |
| - | DIS | DIS | DIS | - | 1 | 1 | Razi | AHV | **GK9** | 77 |
| - | DIS | 1 | 1.1 | - | 1.2 | 1 | Razi | AHV | **FC3** | 78 |
| Not Recovered | 1.3 | 1.3 | 1.2 | 1.78 | 1.8 | 6 | Razi | AHV | **UT8** | 79 |
| Not Recovered | 1.3 | 1.4 | 1.3 | 2 | 2.7 | 6* | Razi | AHV | **QS6** | 80 |
| Not Recovered | DIS | 1.3 | 1.6 | - | 1.58 | 1.8 | Razi | AHV | **RL9** | 81 |
| Not Recovered | DIS | DIS | 1.7 | - | 6 | 1.4 | SnaFab | AHV | **YF3** | 82 |
| - | DIS | DIS | - | - | 0.89 | - | SnaFab | MAS | **NNK8** | 83 |
| - | DIS | DIS | 1.1 | - | 1.1 | 1.16 | Razi | AHV | **KL5** | 84 |
| - | DIS | DIS | 1.1 | - | 1.28 | 1.2 | SnaFab | AHV | **RU5** | 85 |
| - | DIS | DIS | 1 | - | 1.5 | 1.2 | Razi | AHV | **OG6** | 86 |
| Not Recovered | DIS | 1.6 | 1.9 | 3.5 | 6* | 1.6 | Razi | AHV | **RU0** | 87 |
| Recovered | DIS | 1 | - | 1.5 | 6* | 2.4 | Razi | AHV | **CH8** | 88 |
| - | DIS | DIS | DIS | - | - | 1 | SnaFab | AHV | **VL2** | 89 |
| Recovered | 1 | 1.1 | 1.1 | 1.3 | - | 6 | Razi | AHV | **DC8** | 90 |
| Recovered | DIS | 1.3 | 1.1 | - | 1.04 | 1.42 | Razi | AHV | **HV6** | 91 |
| - | DIS | DIS | DIS | 1 | 1.42 | 1 | Razi | AHV | **FL2** | 92 |
| - | DIS | DIS | - | - | - | 6 | SnaFab | AHV | **RS1** | 93 |
| - | DIS | DIS | 1.3 | - | 1.2 | 1 | Razi | AHV | **AJ6** | 94 |
| Not Recovered | DIS | 1.56 | 1.3 | 1 | 1.95 | 1.1 | Razi | AHV | **ZY8** | 95 |
| - | DIS | DIS | DIS | - | 1 | 1 | SnaFab | AHV | **YB6** | 96 |
| - | DIS | DIS | 1.1 | - | 1 | 1.13 | Razi | AHV | **GS2** | 97 |
| Not Recovered | DIS | DIS | DIS | 6* | 6* | 6 | Razi | AHV | **WZ7** | 98 |

DIS: Discharged, N= normal, * = result disrupted

## Table 18 - Assessment of victims’ recovery over 48 hours (local and systemic symptoms)

| ID | Code | Center | Group | PT | PTT | PLT | INR | Local | Result  (Cuttoff-0) | Result (Cuttoff-1) | Result  (Cuttoff-2) |
| --- | --- | --- | --- | --- | --- | --- | --- | --- | --- | --- | --- |
| 1 | **YUAB** | AHV | SnaFab | Recovered | - | - | Not Recovered | Recovered | Not Meet | Meet | Meet |
| 2 | **MAGH** | AHV | SnaFab | - | - | - |  | Recovered | Meet | Meet | Meet |
| 3 | **ALSA** | AHV | SnaFab | - | - | - |  | Recovered | Meet | Meet | Meet |
| 4 | **GHMA** | AHV | SnaFab | Recovered | Recovered | - | Recovered | Recovered | Meet | Meet | Meet |
| 5 | **ABAR** | AHV | SnaFab | Recovered | - | - | Not Recovered | Recovered | Not Meet | Meet | Meet |
| 6 | **HABA** | AHV | SnaFab | - | - | - |  | Recovered | Meet | Meet | Meet |
| 7 | **FASO** | AHV | SnaFab | - | Recovered | - |  | Recovered | Meet | Meet | Meet |
| 8 | **RABA** | AHV | SnaFab | Recovered | Recovered | - | Not Recovered | Recovered | Not Meet | Meet | Meet |
| 9 | **HAHO** | AHV | SnaFab | - | Recovered | Not Recovered | Not Recovered | Recovered | Not Meet | Not Meet | Meet |
| 10 | **REAB** | AHV | SnaFab | - | - | - | Not Recovered | Recovered | Not Meet | Meet | Meet |
| 11 | **ALSE** | AHV | SnaFab | Recovered | - | Recovered | - | Recovered | Meet | Meet | Meet |
| 12 | **BAEB** | AHV | SnaFab | - | - | - | - | Recovered | Meet | Meet | Meet |
| 13 | **FAMA** | AHV | SnaFab | - | - | - | Not Recovered | Recovered | Not Meet | Meet | Meet |
| 14 | **ALHA** | AHV | SnaFab | - | - | - |  | Recovered | Meet | Meet | Meet |
| 15 | **SASH** | AHV | SnaFab | Recovered | Recovered | - | Not Recovered | Recovered | Not Meet | Meet | Meet |
| 16 | **EEZ9** | MAS | SnaFab | - | - | - | - | Recovered | Meet | Meet | Meet |
| 17 | **DDH8** | MAS | Razi | - | - | - | - | Not Recovered | Not Meet | Meet | Meet |
| 18 | **QQG5** | MAS | Razi | - | - | - | - | Recovered | Meet | Meet | Meet |
| 19 | **XXA1** | MAS | SnaFab | - | - | - | - | Recovered | Meet | Meet | Meet |
| 20 | **SSV7** | MAS | Razi | - | - | - | - | Recovered | Meet | Meet | Meet |
| 21 | **BBM6** | MAS | Razi | - | - | - | Recovered | Recovered | Meet | Meet | Meet |
| 22 | **YYY4** | MAS | SnaFab | Recovered | - | - | - | Recovered | Meet | Meet | Meet |
| 23 | **ZZI1** | URM | SnaFab | - | - | Not Recovered | - | Not Recovered | Not Meet | Not Meet | Meet |
| 24 | **SSU8** | MAS | SnaFab | - | - |  | - | Recovered | Meet | Meet | Meet |
| 25 | **NNZ3** | URM | Razi | - | - | Recovered | - | Not Recovered | Not Meet | Meet | Meet |
| 26 | **FFG3** | AHV | SnaFab | Recovered | Recovered | - | Not Recovered | Recovered | Not Meet | Meet | Meet |
| 27 | **IIE0** | AHV | Razi | Not Recovered | Not Recovered | - | Not Recovered | Recovered | Not Meet | Not Meet | Not Meet |
| 28 | **EEG0** | AHV | Razi | - | Recovered | Recovered | Not Recovered | Recovered | Not Meet | Meet | Meet |
| 29 | **JJW4** | AHV | SnaFab | - | - | - | Not Recovered | Recovered | Not Meet | Meet | Meet |
| 30 | **BBI6** | AHV | SnaFab | Recovered | Recovered | - | Not Recovered | Recovered | Not Meet | Meet | Meet |
| 31 | **AAH7** | AHV | SnaFab | Recovered | Recovered | - | Not Recovered | Recovered | Not Meet | Meet | Meet |
| 32 | **AAF9** | AHV | Razi | Recovered | - | - | Not Recovered | Recovered | Not Meet | Meet | Meet |
| 33 | **WWR1** | AHV | Razi | - | - | Recovered | - | Recovered | Meet | Meet | Meet |
| 34 | **RRS3** | AHV | SnaFab | - | - | - | - | Recovered | Meet | Meet | Meet |
| 35 | **JJI8** | AHV | Razi | Recovered | - | - | Recovered | Recovered | Meet | Meet | Meet |
| 36 | **QQW3** | AHV | SnaFab | Recovered | Recovered | - | Not Recovered | Recovered | Not Meet | Meet | Meet |
| 37 | **AAT1** | MAS | SnaFab | - |  | Not Recovered | - | Recovered | Not Meet | Meet | Meet |
| 38 | **VVI7** | AHV | Razi | Recovered |  | - | Recovered | Recovered | Meet | Meet | Meet |
| 39 | **UUL5** | AHV | SnaFab | Recovered | Recovered | - | Not Recovered | Recovered | Not Meet | Meet | Meet |
| 40 | **QQQ3** | AHV | Razi | Recovered | - | - | Not Recovered | Recovered | Not Meet | Meet | Meet |
| 41 | **UUN5** | AHV | Razi | - | - | - | Not Recovered | Recovered | Not Meet | Meet | Meet |
| 42 | **XXN0** | AHV | SnaFab | - | - | - | - | Recovered | Meet | Meet | Meet |
| 43 | **RRA4** | AHV | Razi | - | - | - | - | Recovered | Meet | Meet | Meet |
| 44 | **DDN5** | AHV | SnaFab | - | - | - | - | Recovered | Meet | Meet | Meet |
| 45 | **OOZ0** | AHV | Razi | Recovered | Recovered | - | Recovered | Recovered | Meet | Meet | Meet |
| 46 | **UUT4** | AHV | SnaFab | Recovered | Recovered | - | - | Recovered | Meet | Meet | Meet |
| 47 | **GGM9** | AHV | SnaFab | - | - | - | Not Recovered | Recovered | Not Meet | Meet | Meet |
| 48 | **SSH4** | AHV | SnaFab | Recovered | Recovered | - | Recovered | Recovered | Meet | Meet | Meet |
| 49 | **FFW7** | AHV | Razi | - | - | Recovered | Not Recovered | Recovered | Not Meet | Meet | Meet |
| 50 | **MMM9** | AHV | Razi | Recovered | Recovered | - | Not Recovered | Recovered | Not Meet | Meet | Meet |
| 51 | **KKZ6** | AHV | SnaFab | - | - | - | Recovered | Recovered | Meet | Meet | Meet |
| 52 | **KKW6** | AHV | Razi | Recovered | Recovered | - | Recovered | Recovered | Meet | Meet | Meet |
| 53 | **EEP5** | AHV | Razi | - | - | - | - | Recovered | Meet | Meet | Meet |
| 54 | **FFR9** | AHV | SnaFab | - | - | - | Recovered | Recovered | Meet | Meet | Meet |
| 55 | **UUN6** | AHV | Razi | Recovered | Recovered | - | Recovered | Recovered | Meet | Meet | Meet |
| 56 | **BBP7** | MAS | SnaFab | - | - | - | - | Recovered | Meet | Meet | Meet |
| 57 | **OOE4** | AHV | Razi | - | - | - | - | Recovered | Meet | Meet | Meet |
| 58 | **QQA5** | AHV | SnaFab | Recovered | Recovered | Not Recovered | Not Recovered | Recovered | Not Meet | Not Meet | Meet |
| 59 | **FFT8** | MAS | Razi | Recovered | - | Not Recovered | Recovered | Recovered | Not Meet | Meet | Meet |
| 60 | **JJY3** | AHV | SnaFab | Recovered | - | - | Recovered | Recovered | Meet | Meet | Meet |
| 61 | **GGN0** | AHV | Razi | - | - | - | - | Recovered | Meet | Meet | Meet |
| 62 | **ZZQ0** | AHV | SnaFab | Not Recovered | Recovered | Not Recovered | Recovered | Recovered | Not Meet | Not Meet | Meet |
| 63 | **JJK9** | AHV | Razi | - | - | - | Not Recovered | Recovered | Not Meet | Meet | Meet |
| 64 | **YYE2** | AHV | SnaFab | - | - | - | - | Recovered | Meet | Meet | Meet |
| 65 | **TTJ2** | MAS | Razi | - | - | - | - | Recovered | Meet | Meet | Meet |
| 66 | **GG0** | AHV | SnaFab | - | Recovered | - | Not Recovered | Recovered | Not Meet | Meet | Meet |
| 67 | **NN5** | AHV | Razi | - | - | - | - | Recovered | Meet | Meet | Meet |
| 68 | **JX8** | AHV | Razi | - | - | - | Recovered | Recovered | Meet | Meet | Meet |
| 69 | **QU4** | AHV | Razi | Rcovered | Rcovered | - | Not Recovered | Recovered | Not Meet | Meet | Meet |
| 70 | **EEJ8** | URM | Razi | - | - | - | - | Recovered | Meet | Meet | Meet |
| 71 | **WT3** | AHV | Razi | Not Recovered | Recovered | Not Recovered | Recovered | Recovered | Not Meet | Not Meet | Meet |
| 72 | **DC7** | AHV | SnaFab | - | - | - | - | Recovered | Meet | Meet | Meet |
| 73 | **BK8** | AHV | Razi | - | - | - | Not Recovered | Recovered | Not Meet | Meet | Meet |
| 74 | **ZK8** | AHV | Razi | - | Not Recovered | - | Not Recovered | Recovered | Not Meet | Not Meet | Meet |
| 75 | **FM4** | AHV | Razi | - | Recovered | - | Not Recovered | Recovered | Not Meet | Meet | Meet |
| 76 | **YP2** | AHV | SnaFab | - | Recovered | - | Not Recovered | Recovered | Not Meet | Meet | Meet |
| 77 | **GK9** | AHV | Razi | - | - | - | - | Recovered | Meet | Meet | Meet |
| 78 | **FC3** | AHV | Razi | - | - | - | - | Recovered | Meet | Meet | Meet |
| 79 | **UT8** | AHV | Razi | - | Recovered | - | Not Recovered | Recovered | Not Meet | Meet | Meet |
| 80 | **QS6** | AHV | Razi | - | Recovered | - | Not Recovered | Recovered | Not Meet | Meet | Meet |
| 81 | **RL9** | AHV | Razi | - | Recovered | - | Not Recovered | Recovered | Not Meet | Meet | Meet |
| 82 | **YF3** | AHV | SnaFab | - | Recovered | - | Not Recovered | Recovered | Not Meet | Meet | Meet |
| 83 | **NNK8** | MAS | SnaFab | - | - | - | - | Recovered | Meet | Meet | Meet |
| 84 | **KL5** | AHV | Razi | - | - | - | - | Recovered | Meet | Meet | Meet |
| 85 | **RU5** | AHV | SnaFab | Recovered | - | Recovered | - | Recovered | Meet | Meet | Meet |
| 86 | **OG6** | AHV | Razi | - | - | - | - | Recovered | Meet | Meet | Meet |
| 87 | **RU0** | AHV | Razi | - | Recovered | - | Not Recovered | Recovered | Not Meet | Meet | Meet |
| 88 | **CH8** | AHV | Razi | Recovered | Recovered | - | Recovered | Recovered | Meet | Meet | Meet |
| 89 | **VL2** | AHV | SnaFab | - | - | - | - | Recovered | Meet | Meet | Meet |
| 90 | **DC8** | AHV | Razi | - | Recovered | - | Recovered | Recovered | Meet | Meet | Meet |
| 91 | HV6 | AHV | Razi | - | - | - | Recovered | Recovered | Meet | Meet | Meet |
| 92 | FL2 | AHV | Razi | - | - | Recovered | - | Recovered | Meet | Meet | Meet |
| 93 | RS1 | AHV | SnaFab | - | - | - | - | Recovered | Meet | Meet | Meet |
| 94 | AJ6 | AHV | Razi | - | - | - | - | Recovered | Meet | Meet | Meet |
| 95 | ZY8 | AHV | Razi | - | - | - | Not Recovered | Recovered | Not Meet | Meet | Meet |
| 96 | YB6 | AHV | SnaFab | - | - | - | - | Recovered | Meet | Meet | Meet |
| 97 | GS2 | AHV | Razi | - | - | - | - | Recovered | Meet | Meet | Meet |
| 98 | WZ7 | AHV | Razi | - | - | - | Not Recovered | Recovered | Not Meet | Meet | Meet |

## Table 19 – PT tests of victims at the Ahvaz center

| Result | Visit 8 | Visit 7 | Visit 6 | Visit 5 | Visit 4 | Visit 1 | Group | Center | Code | ID |
| --- | --- | --- | --- | --- | --- | --- | --- | --- | --- | --- |
| Recovered | 16 | 17 | 15 | 13.7 | 16 | 36* | SnaFab | AHV | **YUAB** | 1 |
| - | DIS | - | - | - | - | - | SnaFab | AHV | **MAGH** | 2 |
| - | DIS | DIS | DIS | - | DIS | - | SnaFab | AHV | **ALSA** | 3 |
| Recovered | 13.7 | 13.9 | 12.8 | - | 25.1 | 36* | SnaFab | AHV | **GHMA** | 4 |
| Recovered | DIS | DIS | DIS | - | - | 23.7 | SnaFab | AHV | **ABAR** | 5 |
| - | DIS | DIS | - | - | - | - | SnaFab | AHV | **HABA** | 6 |
| - | DIS | - | - | - | 18 | - | SnaFab | AHV | **FASO** | 7 |
| Recovered | 16.9 | 16.9 | 17.8 | 14 | 20.4 | 13.7 | SnaFab | AHV | **RABA** | 8 |
| - | 13.3 | 12.2 | 13.7 | 14 | 14.7 | 13.3 | SnaFab | AHV | **HAHO** | 9 |
| - | DIS | DIS | - | 36* | - | 36 | SnaFab | AHV | **REAB** | 10 |
| - | 12.9 | 16 | 17.0 | - | 18 | 15 | SnaFab | AHV | **FAMA** | 13 |
| - | DIS | DIS | - | - | - | 14.7 | SnaFab | AHV | **ALHA** | 14 |
| Recovered | DIS | 19.7 | 18.7 | 22 | 17.2 | 36* | SnaFab | AHV | **SASH** | 15 |
| Recovered | 12.4 | 17 | 16 | - | 37* | 108 | SnaFab | AHV | **FFG3** | 26 |
| Not Recovered | 15.2 | 28 | 12 | 14.3 | 36* | 36* | Razi | AHV | **IIE0** | 27 |
| - | DIS | 14 | 15 | 15 | 26 | 36* | Razi | AHV | **EEG0** | 28 |
| - | DIS | 15 | 13 | - | 14.8 | 14 | SnaFab | AHV | **JJW4** | 29 |
| Recovered | DIS | 15 | 19 | 28 | 37 | 36* | SnaFab | AHV | **BBI6** | 30 |
| Recovered | 14 | 14 | 18 | - | 12 | 36* | SnaFab | AHV | **AAH7** | 31 |
| Recovered | DIS | DIS | 15 | 14 | 16 | 21 | Razi | AHV | **AAF9** | 32 |
| - | DIS | 14 | 12.4 | - | 13 | 13 | Razi | AHV | **WWR1** | 33 |
| - | DIS | DIS | 14 | 17 | 18 | 12.4 | SnaFab | AHV | **RRS3** | 34 |
| Recovered | 13 | 13 | 13 | - | 15 | 37* | Razi | AHV | **JJI8** | 35 |
| Recovered | DIS | 18 | 15 | 14 | 12 | 36* | SnaFab | AHV | **QQW3** | 36 |
| Recovered | 12 | 13 | 21 | 22 | 16 | * | Razi | AHV | **VVI7** | 38 |
| Recovered | DIS | 16 | 14 | - | 37 | 14 | SnaFab | AHV | **UUL5** | 39 |
| Recovered | DIS | 14 | 16 | 12.9 | 21 | 18 | Razi | AHV | **QQQ3** | 40 |
| - | 15 | 17 | 14 | - | - | 19 | Razi | AHV | **UUN5** | 41 |
| - | DIS | DIS | - | - | - | 12 | SnaFab | AHV | **XXN0** | 42 |
| - | DIS | DIS | - | 16 | 14 | 13 | Razi | AHV | **RRA4** | 43 |
| - | DIS | DIS | - | - | - | 12 | SnaFab | AHV | **DDN5** | 44 |
| Recovered | DIS | 12.3 | 15 | 16 | * | 37* | Razi | AHV | **OOZ0** | 45 |
| Recovered | 13 | 12 | 13 | - | 37 | 36* | SnaFab | AHV | **UUT4** | 46 |
| - | 13 | 14 | 13 | - | 14 | 13 | SnaFab | AHV | **GGM9** | 47 |
| Recovered | DIS | 13.1 | 16 | - | 37* | 16 | SnaFab | AHV | **SSH4** | 48 |
| - | 14 | 15 | 14 | - | 14 | * | Razi | AHV | **FFW7** | 49 |
| Recovered | 12 | 14 | 13 | 12.4 | 16 | 37 | Razi | AHV | **MMM9** | 50 |
| - | DIS | 13 | 14 | - | 13 | 13 | SnaFab | AHV | **KKZ6** | 51 |
| Recovered | DIS | DIS | 13 | 14 | 25 | 14 | Razi | AHV | **KKW6** | 52 |
| - | DIS | DIS | - | - | - | 12 | Razi | AHV | **EEP5** | 53 |
| - | DIS | 13 | 14 | 15 | - | 16 | SnaFab | AHV | **FFR9** | 54 |
| Recovered | DIS | 13 | 14 | 16.5 | 60* | - | Razi | AHV | **UUN6** | 55 |
| - | DIS | DIS | 13 | - | 13 | 12 | Razi | AHV | **OOE4** | 57 |
| Recovered | DIS | DIS | 15 | 15 | 36 | 36* | SnaFab | AHV | **QQA5** | 58 |
| Recovered | 14.3 | 12 | 15 | 20 | 12 | 13 | SnaFab | AHV | **JJY3** | 60 |
| - | - | - | 13 | - | - | 12 | Razi | AHV | **GGN0** | 61 |
| Recovered | 12 | - | 14 | - | 36 | 36 | SnaFab | AHV | **ZZQ0** | 62 |
| - | - | 14 | 14 | - | 12 | - | Razi | AHV | **JJK9** | 63 |
| - | DIS | DIS | - | - | - | 12 | SnaFab | AHV | **YYE2** | 64 |
| Recovered | 14 | 14 | 13 | - | 36* | 37 | SnaFab | AHV | **GG0** | 66 |
| Recovered | DIS | 12.8 | 13.3 | - | - | 36 | Razi | AHV | **NN5** | 67 |
| - | DIS | DIS | 12 | - | 12 | 14 | Razi | AHV | **JX8** | 68 |
| Recovered | DIS | DIS | DIS | - | 14.9 | 37 | Razi | AHV | **QU4** | 69 |
| Recovered | DIS | DIS | 14 | - | 13 | 38 | Razi | AHV | **WT3** | 71 |
| - | DIS | DIS | DIS | - | - | 12 | SnaFab | AHV | **DC7** | 72 |
| - | 12 | 14 | 17 | - | - | 13 | Razi | AHV | **BK8** | 73 |
| Not Recovered | 15 | 27 | 15 | 36 | - | 36 | Razi | AHV | **ZK8** | 74 |
| Recovered | DIS | DIS | 14.4 | 19 | 36 | 36 | Razi | AHV | **FM4** | 75 |
| Recovered | DIS | 12 | 14.1 | 24 | 36 | 37 | SnaFab | AHV | **YP2** | 76 |
| - | DIS | DIS | DIS | - | 12 | 12 | Razi | AHV | **GK9** | 77 |
| - | DIS | 12 | 13 | - | 13.3 | 12 | Razi | AHV | **FC3** | 78 |
| Recovered | 14 | 14 | 14 | 16.7 | 17 | 60 | Razi | AHV | **UT8** | 79 |
| Recovered | 14 | 15 | 14 | 19 | 23 | 37* | Razi | AHV | **QS6** | 80 |
| - | DIS | 13 | 14 | - | 15.6 | 17 | Razi | AHV | **RL9** | 81 |
| - | DIS | DIS | 17 | - | 36 | 15 | SnaFab | AHV | **YF3** | 82 |
| - | DIS | 13.6 | 13 | - | 13 | 13.1 | Razi | AHV | **KL5** | 84 |
| - | DIS | DIS | 13 | - | 13.8 | 14 | SnaFab | AHV | **RU5** | 85 |
| - | DIS | DIS | 12 | - | 16 | 14 | Razi | AHV | **OG6** | 86 |
| Recovered | DIS | 16 | 18 | 25 | 36 | 12 | Razi | AHV | **RU0** | 87 |
| Recovered | - | 13 | - | 17.5 | 60* | 23 | Razi | AHV | **CH8** | 88 |
| - | - | - | DIS | - | - | 12 | SnaFab | AHV | **VL2** | 89 |
| Recovered | 12.5 | 13.6 | 12.7 | 15.4 | - | 60 | Razi | AHV | **DC8** | 90 |
| - | DIS | 14 | 13 | - | 12.3 | 15 | Razi | AHV | **HV6** | 91 |
| - | DIS | DIS | DIS | 12 | 14.7 | 13 | Razi | AHV | **FL2** | 92 |
| - | - | - | - | - | - | 36 | SnaFab | AHV | **RS1** | 93 |
| - | DIS | DIS | 14 | - | 14 | 12 | Razi | AHV | **AJ6** | 94 |
| - | DIS | 15.5 | 14 | 12 | 17.6 | 13 | Razi | AHV | **ZY8** | 95 |
| - | DIS | - | DIS | - | 12 | 12 | SnaFab | AHV | **YB6** | 96 |
| - | 13 | 13 | 13 | - | 12 | 12.9 | Razi | AHV | **GS2** | 97 |
| Not Recovered | DIS | DIS | DIS | 36* | 36* | 37* | Razi | AHV | **WZ7** | 98 |

## Table 20 – PT tests of victims at the Mashhad center

| ID | Code | Center | Group | Visit 1 | Visit 4 | Visit 5 | Visit 6 | Visit 7 | Visit 8 | Result |
| --- | --- | --- | --- | --- | --- | --- | --- | --- | --- | --- |
| 11 | **ALSE** | MAS | SnaFab | 13.3 | 15 | - | 12.9 | 12.6 | DIS | Recovered |
| 16 | **EEZ9** | MAS | SnaFab | 15.8 | 16.1 | - | 14.3 | 14.1 | DIS | - |
| 17 | **DDH8** | MAS | Razi | 13.8 | 13.2 | 11.6 | 11.6 | 13.5 | - | - |
| 18 | **QQG5** | MAS | Razi | 13 | 12.9 | - | 13.6 | 13.2 | DIS | - |
| 19 | **XXA1** | MAS | SnaFab | 13.4 | 13.4 | - | 12.5 | DIS | DIS | - |
| 20 | **SSV7** | MAS | Razi | 11.9 | 11 | - | 11.4 | DIS | DIS | - |
| 21 | **BBM6** | MAS | Razi | 20.4 | 14.3 | - | - | DIS | DIS | - |
| 22 | **YYY4** | MAS | SnaFab | 33.2 | 12.9 | - | - | DIS | DIS | Recovered |
| 24 | **SSU8** | MAS | SnaFab | 13.3 | 13.1 | - | 13.4 | DIS | DIS | - |
| 37 | **AAT1** | MAS | SnaFab | 12 | 11.5 | - | - | DIS | DIS | - |
| 56 | **BBP7** | MAS | SnaFab | 11 | - | - | 13.2 | DIS | DIS | - |
| 59 | **FFT8** | MAS | Razi | 12 | 17 | - | 14.3 | 11 | DIS | Recovered |
| 65 | **TTJ2** | MAS | Razi | 11.4 | 12.7 | - | 12 | - | - | - |
| 83 | **NNK8** | MAS | SnaFab | - | 8.9 | - | - | - | - | - |

## Table 21 – PT tests of victims at the Urmia center

| ID | Code | Center | Group | Visit 1 | Visit 4 | Visit 5 | Visit 6 | Visit 7 | Visit 8 | Result |
| --- | --- | --- | --- | --- | --- | --- | --- | --- | --- | --- |
| 12 | **BAEB** | URM | SnaFab | 12 | - | - | 13 | DIS | DIS | **-** |
| 23 | **ZZI1** | URM | SnaFab | 13.5 | - | - | 17 | - | DIS | **-** |
| 25 | **NNZ3** | URM | Razi | 13 | 13 | - | - | DIS | DIS | **-** |
| 70 | **EEJ8** | URM | Razi | 13 | 13 | - | 13 | 13 | 13 | **-** |

## Table 22 – PTT tests of victims at the Ahvaz center

| Result | Visit 8 | Visit 7 | Visit 6 | Visit 5 | Visit 4 | Visit 1 | Group | Center | Code | ID |
| --- | --- | --- | --- | --- | --- | --- | --- | --- | --- | --- |
| - | 29 | 30 | 30 | 1.17 | 37 | 47 | SnaFab | AHV | **YUAB** | 1 |
| - | DIS | - | - | - | - | - | SnaFab | AHV | **MAGH** | 2 |
| - | DIS | DIS | DIS | - | DIS | - | SnaFab | AHV | **ALSA** | 3 |
| Recovered | 25 | 31 | 30 | - | 64.6 | 120 | SnaFab | AHV | **GHMA** | 4 |
| - | DIS | DIS | DIS | - | - | 37 | SnaFab | AHV | **ABAR** | 5 |
| - | DIS | DIS | - | - | - | - | SnaFab | AHV | **HABA** | 6 |
| Recovered | DIS | - | - | - | 55 | - | SnaFab | AHV | **FASO** | 7 |
| Recovered | 28 | 33 | 39 | 27 | 55 | 47 | SnaFab | AHV | **RABA** | 8 |
| Recovered | 36 | 24 | 38 | 27 | 39 | 37 | SnaFab | AHV | **HAHO** | 9 |
| - | DIS | DIS | - | 121* | - | 121 | SnaFab | AHV | **REAB** | 10 |
| - | 24 | 30 | 25 | - | 37 | 25 | SnaFab | AHV | **FAMA** | 13 |
| - | DIS | DIS | - | - | - | 37 | SnaFab | AHV | **ALHA** | 14 |
| Recovered | DIS | 37 | 39 | 35 | 31 | 54 | SnaFab | AHV | **SASH** | 15 |
| Recovered | 32 | 25 | 30 | - | 42 | 121* | SnaFab | AHV | **FFG3** | 26 |
| Not Recovered | 40 | 100 | 48 | 35 | 120* | 120* | Razi | AHV | **IIE0** | 27 |
| Recovered | DIS | 36 | 34 | 35 | 36 | 121* | Razi | AHV | **EEG0** | 28 |
| - | DIS | 35 | 30 | - | 25 | 25 | SnaFab | AHV | **JJW4** | 29 |
| Recovered | DIS | 31 | 31 | 39 | 66 | 121* | SnaFab | AHV | **BBI6** | 30 |
| Recovered | 37 | 25 | 30 | - | 121* | 120* | SnaFab | AHV | **AAH7** | 31 |
| - | DIS | DIS | 33 | 28 | 30 | 32 | Razi | AHV | **AAF9** | 32 |
| - | DIS | 30 | 32 | - | 30 | 25 | Razi | AHV | **WWR1** | 33 |
| - | DIS | DIS | 27 | 30 | 25 | 29 | SnaFab | AHV | **RRS3** | 34 |
| - | 28 | 25 | 29 | - | 31 | 41 | Razi | AHV | **JJI8** | 35 |
| Recovered | DIS | 32 | 35 | 31 | 25 | 121* | SnaFab | AHV | **QQW3** | 36 |
| - | 35 | 28 | 30 | 44 | 33 | * | Razi | AHV | **VVI7** | 38 |
| Recovered | DIS | 25 | 27 | - | 121 | 121 | SnaFab | AHV | **UUL5** | 39 |
| - | DIS | 25 | 34 | 38 | 36 | 32 | Razi | AHV | **QQQ3** | 40 |
| - | 31 | 30 | 34 | - | - | 37 | Razi | AHV | **UUN5** | 41 |
| - | DIS | DIS | - | - | - | 25 | SnaFab | AHV | **XXN0** | 42 |
| - | DIS | DIS | - | 25 | 37 | 25 | Razi | AHV | **RRA4** | 43 |
| - | DIS | DIS | - | - | - | 26 | SnaFab | AHV | **DDN5** | 44 |
| Recovered | DIS | 25 | 25 | 35 | * | 121* | Razi | AHV | **OOZ0** | 45 |
| Recovered | 25 | 25 | 25 | - | 121 | 121* | SnaFab | AHV | **UUT4** | 46 |
| - | 33 | 27 | 25 | - | 32 | 25 | SnaFab | AHV | **GGM9** | 47 |
| Recovered | DIS | 24 | 33 | - | 55* | 40 | SnaFab | AHV | **SSH4** | 48 |
| - | 32 | 31 | 35 | - | 28 | * | Razi | AHV | **FFW7** | 49 |
| Recovered | 25 | 45 | 32 | 25 | 34 | 50 | Razi | AHV | **MMM9** | 50 |
| - | DIS | 25 | 25 | - | 25 | 25 | SnaFab | AHV | **KKZ6** | 51 |
| Recovered | DIS | DIS | 27 | 27 | 50 | 121 | Razi | AHV | **KKW6** | 52 |
| - | DIS | DIS | - | - | - | 24 | Razi | AHV | **EEP5** | 53 |
| - | DIS | 39 | 43 | 38 | - | 30 | SnaFab | AHV | **FFR9** | 54 |
| Recovered | DIS | 26 | 29 | 22 | 120* | - | Razi | AHV | **UUN6** | 55 |
| - | DIS | DIS | 32 | - | 32 | 32 | Razi | AHV | **OOE4** | 57 |
| Recovered | DIS | DIS | 34 | 27 | 121 | 121* | SnaFab | AHV | **QQA5** | 58 |
| - | 25 | 39 | 30 | 34 | 50 | 25 | SnaFab | AHV | **JJY3** | 60 |
| - | - | - | 35 | - | - | 24 | Razi | AHV | **GGN0** | 61 |
| Recovered | 33 | - | 26 | - | 65 | 121 | SnaFab | AHV | **ZZQ0** | 62 |
| - | - | 32 | 30 | - | 43 | - | Razi | AHV | **JJK9** | 63 |
| - | DIS | DIS | 24 | - | - | 25 | SnaFab | AHV | **YYE2** | 64 |
| Recovered | 28 | 28 | 27 | - | 121* | 121 | SnaFab | AHV | **GG0** | 66 |
| - | DIS | 26 | 33 | - | - | 32 | Razi | AHV | **NN5** | 67 |
| - | DIS | DIS | 28 | - | 24 | 34 | Razi | AHV | **JX8** | 68 |
| Recovered | DIS | DIS | DIS | - | 46 | 121 | Razi | AHV | **QU4** | 69 |
| Recovered | DIS | DIS | 35 | - | 25 | 120 | Razi | AHV | **WT3** | 71 |
| - | DIS | DIS | DIS | - | - | 27 | SnaFab | AHV | **DC7** | 72 |
| - | 47 | 33 | 55 | - | - | 38 | Razi | AHV | **BK8** | 73 |
| Not Recovered | 35 | 72 | 50 | 121 | - | 121 | Razi | AHV | **ZK8** | 74 |
| Recovered | DIS | DIS | 46 | 90 | 121 | 120 | Razi | AHV | **FM4** | 75 |
| Recovered | DIS | 33 | 36 | 34 | 121 | 121 | SnaFab | AHV | **YP2** | 76 |
| - | DIS | DIS | DIS | - | 35 | 35 | Razi | AHV | **GK9** | 77 |
| - | DIS | 24 | 25 | - | 28 | 25 | Razi | AHV | **FC3** | 78 |
| Recovered | 28 | 25 | 30 | 30 | 31 | 120 | Razi | AHV | **UT8** | 79 |
| Recovered | 30 | 33 | 36 | 35 | 60 | 121* | Razi | AHV | **QS6** | 80 |
| Recovered | DIS | 30 | 39 | - | 32 | 120 | Razi | AHV | **RL9** | 81 |
| Recovered | DIS | DIS | 40 | - | 121 | 121 | SnaFab | AHV | **YF3** | 82 |
| - | DIS | 24 | 32 | - | 25 | 27 | Razi | AHV | **KL5** | 84 |
| - | DIS | DIS | 28 | - | 24 | 25 | SnaFab | AHV | **RU5** | 85 |
| - | DIS | DIS | 26 | - | 38 | 30 | Razi | AHV | **OG6** | 86 |
| Recovered | DIS | 40 | 32 | 48 | 36* | 121 | Razi | AHV | **RU0** | 87 |
| Recovered | - | 30 | - | 25 | 120* | 28 | Razi | AHV | **CH8** | 88 |
| - | - | - | DIS | - | - | 31 | SnaFab | AHV | **VL2** | 89 |
| Recovered | 32 | 40 | 35 | 35 | - | 120 | Razi | AHV | **DC8** | 90 |
| - | DIS | 38 | 36 | - | 24 | 35 | Razi | AHV | **HV6** | 91 |
| - | DIS | DIS | DIS | 30 | 29 | 28 | Razi | AHV | **FL2** | 92 |
| - | - | - | - | - | - | 121 | SnaFab | AHV | **RS1** | 93 |
| - | DIS | DIS | 31 | - | 30 | 26 | Razi | AHV | **AJ6** | 94 |
| - | DIS | 24 | 35 | 25 | 27 | 31 | Razi | AHV | **ZY8** | 95 |
| - | DIS | - | DIS | - | 41 | 25 | SnaFab | AHV | **YB6** | 96 |
| - | 30 | 30 | 25 | - | 30 | 30 | Razi | AHV | **GS2** | 97 |
| - | DIS | DIS | DIS | 36* | 83 | 121* | Razi | AHV | **WZ7** | 98 |

## Table 23 – PTT tests of victims at the Mashhad center

| ID | Code | Center | Group | Visit 1 | Visit 4 | Visit 5 | Visit 6 | Visit 7 | Visit 8 | Result |
| --- | --- | --- | --- | --- | --- | --- | --- | --- | --- | --- |
| 11 | **ALSE** | MAS | SnaFab | 28.5 | 30 | - | 28.7 | 30 | DIS | **-** |
| 16 | **EEZ9** | MAS | SnaFab | 45.4 | 27.1 | - | 28.3 | 30 | DIS | **-** |
| 17 | **DDH8** | MAS | Razi | 29.9 | 27 | 25.1 | 25.1 | 33 | - | **-** |
| 18 | **QQG5** | MAS | Razi | 29 | 30 | - | 30 | 30 | DIS | **-** |
| 19 | **XXA1** | MAS | SnaFab | 37.2 | 38.9 | - | 33.2 | DIS | DIS | **-** |
| 20 | **SSV7** | MAS | Razi | 30 | 24.2 | - | 23.9 | DIS | DIS | **-** |
| 21 | **BBM6** | MAS | Razi | 31.1 | 34 | - | - | DIS | DIS | **-** |
| 22 | **YYY4** | MAS | SnaFab | 28.9 | 28 | - | - | DIS | DIS | **-** |
| 24 | **SSU8** | MAS | SnaFab | 29.9 | 32 | - | 31 | DIS | DIS | **-** |
| 37 | **AAT1** | MAS | SnaFab | 29 | 27 | - | - | DIS | DIS | **-** |
| 56 | **BBP7** | MAS | SnaFab | 30 | - | - | 32 | DIS | DIS | **-** |
| 59 | **FFT8** | MAS | Razi | 23 | 25.5 | - | 32.6 | 40 | DIS | **-** |
| 65 | **TTJ2** | MAS | Razi | 47 | 44 | - | 30 | - | - | **-** |
| 83 | **NNK8** | MAS | SnaFab | - | 27.6 | - | - | - | - | **-** |

## Table 24 – PTT tests of victims at the Urmia center

| ID | Code | Center | Group | Visit 1 | Visit 4 | Visit 5 | Visit 6 | Visit 7 | Visit 8 | Result |
| --- | --- | --- | --- | --- | --- | --- | --- | --- | --- | --- |
| 12 | **BAEB** | URM | SnaFab | 28 | - | - | 25.3 | DIS | DIS | **-** |
| 23 | **ZZI1** | URM | SnaFab | 28 | - | - | 34 | - | DIS | **-** |
| 25 | **NNZ3** | URM | Razi | 34.5 | 38 | - | - | DIS | DIS | **-** |
| 70 | **EEJ8** | URM | Razi | 40 | 42 | - | 35 | 31 | 31 | **-** |

## Table 25 – Platelet tests of victims at the Ahvaz center

| Result | Visit 8 | Visit 7 | Visit 6 | Visit 5 | Visit 4 | Visit 1 | Group | Center | Code | ID |
| --- | --- | --- | --- | --- | --- | --- | --- | --- | --- | --- |
| - | - | - | - | - | - | 208 | SnaFab | AHV | **YUAB** | 1 |
| - | DIS | - | - | - | - | - | SnaFab | AHV | **MAGH** | 2 |
| - | DIS | DIS | DIS | - | DIS | - | SnaFab | AHV | **ALSA** | 3 |
| - | 162 | 199 | - | - | 225 | 261 | SnaFab | AHV | **GHMA** | 4 |
| - | DIS | DIS | DIS | - | - | 185 | SnaFab | AHV | **ABAR** | 5 |
| - | DIS | DIS | - | - | - | - | SnaFab | AHV | **HABA** | 6 |
| - | DIS | - | - | - | 183 | - | SnaFab | AHV | **FASO** | 7 |
| - | - | 228 | 252 | - | 256 | 291 | SnaFab | AHV | **RABA** | 8 |
| Not Recovered | 120 | 133 | 163 | - | - | 101 | SnaFab | AHV | **HAHO** | 9 |
| - | DIS | DIS | 161 | - | 163 | 193 | SnaFab | AHV | **REAB** | 10 |
| - | - | DIS | - | - | - | 266 | SnaFab | AHV | **FAMA** | 13 |
| - | DIS | DIS | - | - | 236 | 247 | SnaFab | AHV | **ALHA** | 14 |
| - | DIS | 188 | - | - | - | 193 | SnaFab | AHV | **SASH** | 15 |
| - | - | 206 | 220 | - | 227 | 222 | SnaFab | AHV | **FFG3** | 26 |
| - | 231 | 202 | - | - | 229 | 264 | Razi | AHV | **IIE0** | 27 |
| Recovered | DIS | 200 | 202 | - | 125 | 222 | Razi | AHV | **EEG0** | 28 |
| - | DIS | - | - | - | 198 | 165 | SnaFab | AHV | **JJW4** | 29 |
| - | DIS | - | 224 | - | 186 | 177 | SnaFab | AHV | **BBI6** | 30 |
| - | 212 | 221 | - | - | 238 | 234 | SnaFab | AHV | **AAH7** | 31 |
| - | DIS | DIS | 199 | - | - | 193 | Razi | AHV | **AAF9** | 32 |
| Recovered | DIS | - | - | - | 259 | 110 | Razi | AHV | **WWR1** | 33 |
| - | DIS | DIS | - | 271 | 386 | 336 | SnaFab | AHV | **RRS3** | 34 |
| - | - | - | - | - | - | 358 | Razi | AHV | **JJI8** | 35 |
| - | DIS | - | 303 | 314 | 265 | 313 | SnaFab | AHV | **QQW3** | 36 |
| - | 190 | 240 | 278 | - | 247 | 236 | Razi | AHV | **VVI7** | 38 |
| - | DIS | 186 | - | 206 | 206 | 237 | SnaFab | AHV | **UUL5** | 39 |
| - | DIS | 303 | 267 | 228 | 285 | 304 | Razi | AHV | **QQQ3** | 40 |
| - | 238 | 220 | 247 | - | - | 262 | Razi | AHV | **UUN5** | 41 |
| - | DIS | DIS | - | - | - | 246 | SnaFab | AHV | **XXN0** | 42 |
| - | DIS | DIS | - | - | - | 279 | Razi | AHV | **RRA4** | 43 |
| - | DIS | DIS | - | - | - | 205 | SnaFab | AHV | **DDN5** | 44 |
| - | DIS | 202 | 156 | - | 166 | 181 | Razi | AHV | **OOZ0** | 45 |
| - | - | - | - | - | 211 | 223 | SnaFab | AHV | **UUT4** | 46 |
| - | - | - | - | - | - | 260 | SnaFab | AHV | **GGM9** | 47 |
| - | DIS | - | 234 | - | 212 | - | SnaFab | AHV | **SSH4** | 48 |
| Recovered | 196 | 183 | 240 | - | - | 103 | Razi | AHV | **FFW7** | 49 |
| - | 206 | - | 169 | - | 196 | 217 | Razi | AHV | **MMM9** | 50 |
| - | DIS | - | - | - | - | 313 | SnaFab | AHV | **KKZ6** | 51 |
| - | DIS | DIS | - | - | - | - | Razi | AHV | **KKW6** | 52 |
| - | DIS | DIS | - | - | - | 190 | Razi | AHV | **EEP5** | 53 |
| - | DIS | - | 219 | - | - | 246 | SnaFab | AHV | **FFR9** | 54 |
| - | DIS | - | - | 319 | 321 | - | Razi | AHV | **UUN6** | 55 |
| - | DIS | DIS | - | - | - | 197 | Razi | AHV | **OOE4** | 57 |
| Not Recovered | DIS | DIS | - | - | 60* | 60* | SnaFab | AHV | **QQA5** | 58 |
| - | DIS | - | 258 | - | 297 | 301 | SnaFab | AHV | **JJY3** | 60 |
| - | - | - | - | - | - | 287 | Razi | AHV | **GGN0** | 61 |
| Not Recovered | - | 114 | 133 | - | 167 | 129 | SnaFab | AHV | **ZZQ0** | 62 |
| - | - | 217 | - | - | 200 | - | Razi | AHV | **JJK9** | 63 |
| - | DIS | DIS | 240 | - | - | 323 | SnaFab | AHV | **YYE2** | 64 |
| - | - | - | - | - | 314 | 361 | SnaFab | AHV | **GG0** | 66 |
| - | DIS | - | - | - | 189 | 190 | Razi | AHV | **NN5** | 67 |
| - | DIS | DIS | 175 | - | - | 234 | Razi | AHV | **JX8** | 68 |
| - | DIS | DIS | DIS | - | 234 | 259 | Razi | AHV | **QU4** | 69 |
| Not Recovered | DIS | DIS | 139 | - | 145 | 215 | Razi | AHV | **WT3** | 71 |
| - | DIS | DIS | DIS | - | - | 219 | SnaFab | AHV | **DC7** | 72 |
| - | 145 | 174 | 176 | - | - | 180 | Razi | AHV | **BK8** | 73 |
| - | 174 | 210 | 238 | 236 | - | 219 | Razi | AHV | **ZK8** | 74 |
| - | DIS | DIS | 219 | 222 | 258 | 279 | Razi | AHV | **FM4** | 75 |
| - | DIS | 190 | - | - | - | 221 | SnaFab | AHV | **YP2** | 76 |
| - | DIS | DIS | DIS | - | 101 | 259 | Razi | AHV | **GK9** | 77 |
| - | DIS | 239 | - | - | 250 | 264 | Razi | AHV | **FC3** | 78 |
| - | 236 | 274 | 263 | 224 | - | 283 | Razi | AHV | **UT8** | 79 |
| - | 224 | 224 | 205 | 216 | 242 | 290 | Razi | AHV | **QS6** | 80 |
| - | DIS | 318 | 304 | - | 283 | - | Razi | AHV | **RL9** | 81 |
| - | DIS | DIS | 202 | - | 198 | 266 | SnaFab | AHV | **YF3** | 82 |
| - | DIS | - | 328 | - | 334 | 357 | Razi | AHV | **KL5** | 84 |
| Recovered | DIS | DIS | 165 | - | 150 | 122 | SnaFab | AHV | **RU5** | 85 |
| - | DIS | DIS | 237 | - | 199 | 238 | Razi | AHV | **OG6** | 86 |
| - | DIS | 156 | 192 | 198 | 184 | 205 | Razi | AHV | **RU0** | 87 |
| - | - | - | 280 | 264 | 274 | 337 | Razi | AHV | **CH8** | 88 |
| - | - | - | DIS | - | - | 330 | SnaFab | AHV | **VL2** | 89 |
| - | 213 | 195 | 198 | - | - | 228 | Razi | AHV | **DC8** | 90 |
| - | DIS | 191 | 242 | - | - | 245 | Razi | AHV | HV6 | 91 |
| Recovered | DIS | DIS | DIS | 213 | 210 | 71 | Razi | AHV | FL2 | 92 |
| - | - | - | - | - | - | 309 | SnaFab | AHV | RS1 | 93 |
| - | DIS | DIS | 259 | - | 223 | 177 | Razi | AHV | AJ6 | 94 |
| - | DIS | 287 | 277 | 247 | 265 | 314 | Razi | AHV | ZY8 | 95 |
| - | DIS | - | DIS | - | 223 | 224 | SnaFab | AHV | YB6 | 96 |
| - | 318 | 318 | 209 | - | 177 | 155 | Razi | AHV | GS2 | 97 |
| - | DIS | DIS | DIS | - | 216 | 270 | Razi | AHV | WZ7 | 98 |

## Table 26 – Platelet tests of victims at the Mashhad center

| Result | Visit 8 | Visit 7 | Visit 6 | Visit 5 | Visit 4 | Visit 1 | Group | Center | Code | ID |
| --- | --- | --- | --- | --- | --- | --- | --- | --- | --- | --- |
| Recovered | DIS | 198 | 225 | - | 221 | 143 | SnaFab | MAS | **ALSE** | 11 |
| - | DIS | 71` | 74 | - | 82 | 96 | SnaFab | MAS | **EEZ9** | 16 |
| - | 172 | 166 | 173 | 173 | 193 | 218 | Razi | MAS | **DDH8** | 17 |
| - | DIS | 190 | 178 | - | 180 | 206 | Razi | MAS | **QQG5** | 18 |
| - | DIS | DIS | 235 | - | 233 | 222 | SnaFab | MAS | **XXA1** | 19 |
| - | DIS | DIS | - | - | 152 | 237 | Razi | MAS | **SSV7** | 20 |
| - | DIS | DIS | - | - | 166 | 159 | Razi | MAS | **BBM6** | 21 |
| - | DIS | DIS | 189 | - | 188 | 143 | SnaFab | MAS | **YYY4** | 22 |
| - | DIS | DIS | 205 | - | 189 | 181 | SnaFab | MAS | **SSU8** | 24 |
| Not Recovered | DIS | DIS | - | - | 141 | 228 | SnaFab | MAS | **AAT1** | 37 |
| - | DIS | DIS | 192 | - | - | 182 | SnaFab | MAS | **BBP7** | 56 |
| Not Recovered | DIS | - | - | - | 119 | 34 | Razi | MAS | **FFT8** | 59 |
| - | - | - | 214 | - | 253 | 262 | Razi | MAS | **TTJ2** | 65 |
| - | - | - | - | - | 192 | 174 | SnaFab | MAS | **NNK8** | 83 |

## Table 27 – Platelet tests of victims at the Urmia center

| ID | Code | Center | Group | Visit 1 | Visit 4 | Visit 5 | Visit 6 | Visit 7 | Visit 8 | Result |
| --- | --- | --- | --- | --- | --- | --- | --- | --- | --- | --- |
| 12 | **BAEB** | URM | SnaFab | 199 | - | - | 211 | DIS | DIS | **-** |
| 23 | **ZZI1** | URM | SnaFab | 107 | - | - | 136 | 15 | DIS | **Not Recovered** |
| 25 | **NNZ3** | URM | Razi | 128 | 295 | - | - | DIS | DIS | **Recovered** |
| 70 | **EEJ8** | URM | Razi | 350 | 280 | - | 200 | 306 | 306 | **-** |

## Table 28 – INR tests of victims at the Ahvaz center

| Result | Visit 8 | Visit 7 | Visit 6 | Visit 5 | Visit 4 | Visit 1 | Group | Center | Code | ID |
| --- | --- | --- | --- | --- | --- | --- | --- | --- | --- | --- |
| Not Recovered | 1.6 | 1.6 | 1.4 | 29 | 1.6 | 7* | SnaFab | AHV | **YUAB** | 1 |
| - | DIS | DIS | - | - | - | - | SnaFab | AHV | **MAGH** | 2 |
| - | DIS | DIS | DIS | - | DIS | - | SnaFab | AHV | **ALSA** | 3 |
| Recovered | 1.2 | 1.2 | 1.08 | - | 3.9 | 6* | SnaFab | AHV | **GHMA** | 4 |
| Not Recovered | DIS | DIS | DIS | - | - | 3.5 | SnaFab | AHV | **ABAR** | 5 |
| - | DIS | DIS | - | - | - | - | SnaFab | AHV | **HABA** | 6 |
| - | DIS | DIS | - | - | 2.05 | - | SnaFab | AHV | **FASO** | 7 |
| Not Recovered | 1.81 | 1.81 | 2.01 | 1.2 | 2.62 | 1.2 | SnaFab | AHV | **RABA** | 8 |
| Not Recovered | 1.13 | 1.13 | 1.2 | 1.2 | 1.38 | 1.13 | SnaFab | AHV | **HAHO** | 9 |
| Not Recovered | DIS | DIS | - | 6* | - | 6 | SnaFab | AHV | **REAB** | 10 |
| Not Recovered | 1.09 | 1.09 | 1.6 | - | 1.84 | 1.4 | SnaFab | AHV | **FAMA** | 13 |
| - | DIS | DIS | - | - | - | 1.3 | SnaFab | AHV | **ALHA** | 14 |
| Not Recovered | DIS | DIS | 2.21 | 1.6 | 1.5 | 6* | SnaFab | AHV | **SASH** | 15 |
| Not Recovered | 1.04 | 1.8 | 1.6 | - | 6* | 11.4 | SnaFab | AHV | **FFG3** | 26 |
| Not Recovered | 1.31 | 4 | 1 | 1.2 | 6* | 6* | Razi | AHV | **IIE0** | 27 |
| Not Recovered | DIS | 1.3 | 1.4 | 1.4 | 3.4 | 6* | Razi | AHV | **EEG0** | 28 |
| Not Recovered | DIS | 1.4 | 1.1 | - | 1.27 | 1.2 | SnaFab | AHV | **JJW4** | 29 |
| Not Recovered | DIS | 1.4 | 2.1 | 4 | 7 | - | SnaFab | AHV | **BBI6** | 30 |
| Not Recovered | 1.3 | 1.3 | 1.9 | - | 1 | 6* | SnaFab | AHV | **AAH7** | 31 |
| Not Recovered | DIS | DIS | 1.4 | 1.2 | 1.5 | 2.5 | Razi | AHV | **AAF9** | 32 |
| - | DIS | 1.2 | 1.04 | - | 1.1 | 1.1 | Razi | AHV | **WWR1** | 33 |
| - | DIS | DIS | 1.3 | 1.8 | 1.9 | 1.04 | SnaFab | AHV | **RRS3** | 34 |
| Recovered | 1.1 | 1.1 | 1.15 | - | 1.4 | 6.1 | Razi | AHV | **JJI8** | 35 |
| Not Recovered | DIS | DIS | 1.4 | 1.3 | 1 | 6* | SnaFab | AHV | **QQW3** | 36 |
| Recovered | 1 | 1.1 | 2.6 | 2.7 | 1.6 | * | Razi | AHV | **VVI7** | 38 |
| Not Recovered | DIS | 1.6 | 1.3 | - | 6.1 | 1.2 | SnaFab | AHV | **UUL5** | 39 |
| Not Recovered | DIS | 1.3 | 1.6 | 1.09 | 2.5 | 1.8 | Razi | AHV | **QQQ3** | 40 |
| Not Recovered | 1.4 | 1.8 | 1.2 | - | - | 2.1 | Razi | AHV | **UUN5** | 41 |
| - | DIS | DIS | - | - | - | 1 | SnaFab | AHV | **XXN0** | 42 |
| - | DIS | DIS | - | 1.5 | 1.3 | 1.1 | Razi | AHV | **RRA4** | 43 |
| - | DIS | DIS | - | - | - | 1 | SnaFab | AHV | **DDN5** | 44 |
| Recovered | DIS | 1.03 | 1.4 | 1.5 | * | 6* | Razi | AHV | **OOZ0** | 45 |
| - | 1.1 | 1 | 1.1 | - | - | -* | SnaFab | AHV | **UUT4** | 46 |
| Not Recovered | 1.1 | 1.3 | 1.1 | - | 1.3 | 1.1 | SnaFab | AHV | **GGM9** | 47 |
| Recovered | DIS | 1.1 | 1.6 | - | 7* | 1.6 | SnaFab | AHV | **SSH4** | 48 |
| Not Recovered | 1.3 | 1.4 | 1.3 | - | 1.3 | * | Razi | AHV | **FFW7** | 49 |
| Not Recovered | 1 | 1.3 | 1.1 | 1.04 | 1.6 | 6.1 | Razi | AHV | **MMM9** | 50 |
| Recovered | DIS | 1.1 | 1.3 | - | 1.1 | 1.1 | SnaFab | AHV | **KKZ6** | 51 |
| Recovered | DIS | DIS | 1.1 | 1.2 | 3.3 | 1.3 | Razi | AHV | **KKW6** | 52 |
| - | DIS | DIS | - | - | - | 1 | Razi | AHV | **EEP5** | 53 |
| Recovered | DIS | 1.1 | 1.2 | 1.29 | - | 1.6 | SnaFab | AHV | **FFR9** | 54 |
| Recovered | DIS | 1 | 1.1 | 1.5 | 6* | - | Razi | AHV | **UUN6** | 55 |
| - | DIS | DIS | 1 | - | 1 | 1 | Razi | AHV | **OOE4** | 57 |
| Not Recovered | DIS | DIS | 1.4 | 1.4 | * | 6* | SnaFab | AHV | **QQA5** | 58 |
| Recovered | 1.2 | 1 | 1.4 | 2.9 | 1 | 1.1 | SnaFab | AHV | **JJY3** | 60 |
| - | DIS | DIS | 1.1 | - | - | 1 | Razi | AHV | **GGN0** | 61 |
| Recovered | 1 | DIS | 1.2 | - | 6 | 6 | SnaFab | AHV | **ZZQ0** | 62 |
| Not Recovered | DIS | 1.3 | 1.2 | - | 1 | - | Razi | AHV | **JJK9** | 63 |
| - | DIS | DIS | - | - | - | 1 | SnaFab | AHV | **YYE2** | 64 |
| Not Recovered | 1.3 | 1.3 | 1.1 | - | 7* | 6 | SnaFab | AHV | **GG0** | 66 |
| - | DIS | 1.08 | 1.13 | - | - | - | Razi | AHV | **NN5** | 67 |
| Recovered | DIS | DIS | 1 | - | 1 | 1.3 | Razi | AHV | **JX8** | 68 |
| Not Recovered | DIS | DIS | DIS | - | 1.28 | 6 | Razi | AHV | **QU4** | 69 |
| Recovered | DIS | DIS | 1.1 | - | 1 | 6 | Razi | AHV | **WT3** | 71 |
| - | DIS | DIS | DIS | - | - | 1 | SnaFab | AHV | **DC7** | 72 |
| Not Recovered | 1 | 1.3 | 1.6 | - | - | 1.1 | Razi | AHV | **BK8** | 73 |
| Not Recovered | 1.4 | 3.8 | 1.4 | 6 | - | 6 | Razi | AHV | **ZK8** | 74 |
| Not Recovered | DIS | DIS | 1.37 | 2 | 6 | 6 | Razi | AHV | **FM4** | 75 |
| Not Recovered | DIS | 1.3 | 1.32 | 3.1 | 6 | 6 | SnaFab | AHV | **YP2** | 76 |
| - | DIS | DIS | DIS | - | 1 | 1 | Razi | AHV | **GK9** | 77 |
| - | DIS | 1 | 1.1 | - | 1.2 | 1 | Razi | AHV | **FC3** | 78 |
| Not Recovered | 1.3 | 1.3 | 1.2 | 1.78 | 1.8 | 6 | Razi | AHV | **UT8** | 79 |
| Not Recovered | 1.3 | 1.4 | 1.3 | 2 | 2.7 | 6* | Razi | AHV | **QS6** | 80 |
| Not Recovered | DIS | 1.3 | 1.6 | - | 1.58 | 1.8 | Razi | AHV | **RL9** | 81 |
| Not Recovered | DIS | DIS | 1.7 | - | 6 | 1.4 | SnaFab | AHV | **YF3** | 82 |
| - | DIS | DIS | 1.1 | - | 1.1 | 1.16 | Razi | AHV | **KL5** | 84 |
| - | DIS | DIS | 1.1 | - | 1.28 | 1.2 | SnaFab | AHV | **RU5** | 85 |
| - | DIS | DIS | 1 | - | 1.5 | 1.2 | Razi | AHV | **OG6** | 86 |
| Not Recovered | DIS | 1.6 | 1.9 | 3.5 | 6* | 1.6 | Razi | AHV | **RU0** | 87 |
| Recovered | DIS | 1 | - | 1.5 | 6* | 2.4 | Razi | AHV | **CH8** | 88 |
| - | DIS | DIS | DIS | - | - | 1 | SnaFab | AHV | **VL2** | 89 |
| Recovered | 1 | 1.1 | 1.1 | 1.3 | - | 6 | Razi | AHV | **DC8** | 90 |
| Recovered | DIS | 1.3 | 1.1 | - | 1.04 | 1.42 | Razi | AHV | **HV6** | 91 |
| - | DIS | DIS | DIS | 1 | 1.42 | 1 | Razi | AHV | **FL2** | 92 |
| - | DIS | DIS | - | - | - | 6 | SnaFab | AHV | **RS1** | 93 |
| - | DIS | DIS | 1.3 | - | 1.2 | 1 | Razi | AHV | **AJ6** | 94 |
| Not Recovered | DIS | 1.56 | 1.3 | 1 | 1.95 | 1.1 | Razi | AHV | **ZY8** | 95 |
| - | DIS | DIS | DIS | - | 1 | 1 | SnaFab | AHV | **YB6** | 96 |
| - | DIS | DIS | 1.1 | - | 1 | 1.13 | Razi | AHV | **GS2** | 97 |
| Not Recovered | DIS | DIS | DIS | 6* | 6* | 6 | Razi | AHV | **WZ7** | 98 |

## Table 29 – INR tests of victims at the Mashhad center

| ID | Code | Center | Group | Visit 1 | Visit 4 | Visit 5 | Visit 6 | Visit 7 | Visit 8 | Result |
| --- | --- | --- | --- | --- | --- | --- | --- | --- | --- | --- |
| 11 | **ALSE** | MAS | SnaFab | 1.13 | 1.31 | - | 1.09 | DIS | DIS | **-** |
| 16 | **EEZ9** | MAS | SnaFab | 1.4 | 1.43 | - | 1.24 | DIS | DIS | **-** |
| 17 | **DDH8** | MAS | Razi | 1.25 | 1.17 | 0.95 | 0.95 | 1.21 | - | **-** |
| 18 | **QQG5** | MAS | Razi | 1.14 | 1.12 | - | 1.22 | 1.16 | DIS | **-** |
| 19 | **XXA1** | MAS | SnaFab | 1.19 | 1.19 | - | 1.07 | DIS | DIS | **-** |
| 20 | **SSV7** | MAS | Razi | 0.99 | 0.83 | - | 0.92 | DIS | DIS | **-** |
| 21 | **BBM6** | MAS | Razi | 2.35 | 1.33 | - | - | DIS | DIS | **Recovered** |
| 22 | **YYY4** | MAS | SnaFab | 1 | 1.12 | - | - | DIS | DIS | **-** |
| 24 | **SSU8** | MAS | SnaFab | 1.18 | 1.15 | - | 1.19 | DIS | DIS | **-** |
| 37 | **AAT1** | MAS | SnaFab | 1 | 0.93 | - | - | DIS | DIS | **-** |
| 56 | **BBP7** | MAS | SnaFab | 0.9 | - | - | 1.09 | DIS | DIS | **-** |
| 59 | **FFT8** | MAS | Razi | 1 | 1.75 | - | 1.33 | DIS | DIS | **Recovered** |
| 65 | **TTJ2** | MAS | Razi | 1.04 | 1.15 | - | 1.09 | - | - | **-** |
| 83 | **NNK8** | MAS | SnaFab | - | 0.89 | - | - | - | - | **-** |

## Table 30 – INR tests of victims at the Urmia center

| ID | Code | Center | Group | Visit 1 | Visit 4 | Visit 5 | Visit 6 | Visit 7 | Visit 8 | Result |
| --- | --- | --- | --- | --- | --- | --- | --- | --- | --- | --- |
| 12 | **BAEB** | URM | SnaFab | 0.88 | - | - | 1 | DIS | DIS | **-** |
| 23 | **ZZI1** | URM | SnaFab | 1.06 | - | - | 1.48 | DIS | DIS | **-** |
| 25 | **NNZ3** | URM | Razi | 1 | 1 | - | - | DIS | DIS | **-** |
| 70 | **EEJ8** | URM | Razi | 1 | 1.2 | - | 1.1 | 1.1 | 1.1 | **-** |

## Table 31 - Assessment of victims’ recovery over 48 hours (local and systemic symptoms) at the Ahvaz center

| ID | Code | Center | Group | PT | PTT | PLT | INR | Local | Result  (Cuttoff-0) | Result (Cuttoff-1) | Result  (Cuttoff-2) |
| --- | --- | --- | --- | --- | --- | --- | --- | --- | --- | --- | --- |
| 1 | **YUAB** | AHV | SnaFab | Recovered | - | - | Not Recovered | Recovered | Not Meet | Meet | Meet |
| 2 | **MAGH** | AHV | SnaFab | - | - | - |  | Recovered | Meet | Meet | Meet |
| 3 | **ALSA** | AHV | SnaFab | - | - | - |  | Recovered | Meet | Meet | Meet |
| 4 | **GHMA** | AHV | SnaFab | Recovered | Recovered | - | Recovered | Recovered | Meet | Meet | Meet |
| 5 | **ABAR** | AHV | SnaFab | Recovered | - | - | Not Recovered | Recovered | Not Meet | Meet | Meet |
| 6 | **HABA** | AHV | SnaFab | - | - | - |  | Recovered | Meet | Meet | Meet |
| 7 | **FASO** | AHV | SnaFab | - | Recovered | - |  | Recovered | Meet | Meet | Meet |
| 8 | **RABA** | AHV | SnaFab | Recovered | Recovered | - | Not Recovered | Recovered | Not Meet | Meet | Meet |
| 9 | **HAHO** | AHV | SnaFab | - | Recovered | Not Recovered | Not Recovered | Recovered | Not Meet | Not Meet | Meet |
| 10 | **REAB** | AHV | SnaFab | - | - | - | Not Recovered | Recovered | Not Meet | Meet | Meet |
| 13 | **FAMA** | AHV | SnaFab | - | - | - | Not Recovered | Recovered | Not Meet | Meet | Meet |
| 14 | **ALHA** | AHV | SnaFab | - | - | - |  | Recovered | Meet | Meet | Meet |
| 15 | **SASH** | AHV | SnaFab | Recovered | Recovered | - | Not Recovered | Recovered | Not Meet | Meet | Meet |
| 26 | **FFG3** | AHV | SnaFab | Recovered | Recovered | - | Not Recovered | Recovered | Not Meet | Meet | Meet |
| 27 | **IIE0** | AHV | Razi | Not Recovered | Not Recovered | - | Not Recovered | Recovered | Not Meet | Not Meet | Not Meet |
| 28 | **EEG0** | AHV | Razi | - | Recovered | Recovered | Not Recovered | Recovered | Not Meet | Meet | Meet |
| 29 | **JJW4** | AHV | SnaFab | - | - | - | Not Recovered | Recovered | Not Meet | Meet | Meet |
| 30 | **BBI6** | AHV | SnaFab | Recovered | Recovered | - | Not Recovered | Recovered | Not Meet | Meet | Meet |
| 31 | **AAH7** | AHV | SnaFab | Recovered | Recovered | - | Not Recovered | Recovered | Not Meet | Meet | Meet |
| 32 | **AAF9** | AHV | Razi | Recovered | - | - | Not Recovered | Recovered | Not Meet | Meet | Meet |
| 33 | **WWR1** | AHV | Razi | - | - | Recovered | - | Recovered | Meet | Meet | Meet |
| 34 | **RRS3** | AHV | SnaFab | - | - | - | - | Recovered | Meet | Meet | Meet |
| 35 | **JJI8** | AHV | Razi | Recovered | - | - | Recovered | Recovered | Meet | Meet | Meet |
| 36 | **QQW3** | AHV | SnaFab | Recovered | Recovered | - | Not Recovered | Recovered | Not Meet | Meet | Meet |
| 38 | **VVI7** | AHV | Razi | Recovered |  | - | Recovered | Recovered | Meet | Meet | Meet |
| 39 | **UUL5** | AHV | SnaFab | Recovered | Recovered | - | Not Recovered | Recovered | Not Meet | Meet | Meet |
| 40 | **QQQ3** | AHV | Razi | Recovered | - | - | Not Recovered | Recovered | Not Meet | Meet | Meet |
| 41 | **UUN5** | AHV | Razi | - | - | - | Not Recovered | Recovered | Not Meet | Meet | Meet |
| 42 | **XXN0** | AHV | SnaFab | - | - | - | - | Recovered | Meet | Meet | Meet |
| 43 | **RRA4** | AHV | Razi | - | - | - | - | Recovered | Meet | Meet | Meet |
| 44 | **DDN5** | AHV | SnaFab | - | - | - | - | Recovered | Meet | Meet | Meet |
| 45 | **OOZ0** | AHV | Razi | Recovered | Recovered | - | Recovered | Recovered | Meet | Meet | Meet |
| 46 | **UUT4** | AHV | SnaFab | Recovered | Recovered | - | - | Recovered | Meet | Meet | Meet |
| 47 | **GGM9** | AHV | SnaFab | - | - | - | Not Recovered | Recovered | Not Meet | Meet | Meet |
| 48 | **SSH4** | AHV | SnaFab | Recovered | Recovered | - | Recovered | Recovered | Meet | Meet | Meet |
| 49 | **FFW7** | AHV | Razi | - | - | Recovered | Not Recovered | Recovered | Not Meet | Meet | Meet |
| 50 | **MMM9** | AHV | Razi | Recovered | Recovered | - | Not Recovered | Recovered | Not Meet | Meet | Meet |
| 51 | **KKZ6** | AHV | SnaFab | - | - | - | Recovered | Recovered | Meet | Meet | Meet |
| 52 | **KKW6** | AHV | Razi | Recovered | Recovered | - | Recovered | Recovered | Meet | Meet | Meet |
| 53 | **EEP5** | AHV | Razi | - | - | - | - | Recovered | Meet | Meet | Meet |
| 54 | **FFR9** | AHV | SnaFab | - | - | - | Recovered | Recovered | Meet | Meet | Meet |
| 55 | **UUN6** | AHV | Razi | Recovered | Recovered | - | Recovered | Recovered | Meet | Meet | Meet |
| 57 | **OOE4** | AHV | Razi | - | - | - | - | Recovered | Meet | Meet | Meet |
| 58 | **QQA5** | AHV | SnaFab | Recovered | Recovered | Not Recovered | Not Recovered | Recovered | Not Meet | Not Meet | Meet |
| 60 | **JJY3** | AHV | SnaFab | Recovered | - | - | Recovered | Recovered | Meet | Meet | Meet |
| 61 | **GGN0** | AHV | Razi | - | - | - | - | Recovered | Meet | Meet | Meet |
| 62 | **ZZQ0** | AHV | SnaFab | Not Recovered | Recovered | Not Recovered | Recovered | Recovered | Not Meet | Not Meet | Meet |
| 63 | **JJK9** | AHV | Razi | - | - | - | Not Recovered | Recovered | Not Meet | Meet | Meet |
| 64 | **YYE2** | AHV | SnaFab | - | - | - | - | Recovered | Meet | Meet | Meet |
| 66 | **GG0** | AHV | SnaFab | - | Recovered | - | Not Recovered | Recovered | Not Meet | Meet | Meet |
| 67 | **NN5** | AHV | Razi | - | - | - | - | Recovered | Meet | Meet | Meet |
| 68 | **JX8** | AHV | Razi | - | - | - | Recovered | Recovered | Meet | Meet | Meet |
| 69 | **QU4** | AHV | Razi | Rcovered | Rcovered | - | Not Recovered | Recovered | Not Meet | Meet | Meet |
| 71 | **WT3** | AHV | Razi | Not Recovered | Recovered | Not Recovered | Recovered | Recovered | Not Meet | Not Meet | Meet |
| 72 | **DC7** | AHV | SnaFab | - | - | - | - | Recovered | Meet | Meet | Meet |
| 73 | **BK8** | AHV | Razi | - | - | - | Not Recovered | Recovered | Not Meet | Meet | Meet |
| 74 | **ZK8** | AHV | Razi | - | Not Recovered | - | Not Recovered | Recovered | Not Meet | Not Meet | Meet |
| 75 | **FM4** | AHV | Razi | - | Recovered | - | Not Recovered | Recovered | Not Meet | Meet | Meet |
| 76 | **YP2** | AHV | SnaFab | - | Recovered | - | Not Recovered | Recovered | Not Meet | Meet | Meet |
| 77 | **GK9** | AHV | Razi | - | - | - | - | Recovered | Meet | Meet | Meet |
| 78 | **FC3** | AHV | Razi | - | - | - | - | Recovered | Meet | Meet | Meet |
| 79 | **UT8** | AHV | Razi | - | Recovered | - | Not Recovered | Recovered | Not Meet | Meet | Meet |
| 80 | **QS6** | AHV | Razi | - | Recovered | - | Not Recovered | Recovered | Not Meet | Meet | Meet |
| 81 | **RL9** | AHV | Razi | - | Recovered | - | Not Recovered | Recovered | Not Meet | Meet | Meet |
| 82 | **YF3** | AHV | SnaFab | - | Recovered | - | Not Recovered | Recovered | Not Meet | Meet | Meet |
| 84 | **KL5** | AHV | Razi | - | - | - | - | Recovered | Meet | Meet | Meet |
| 85 | **RU5** | AHV | SnaFab | Recovered | - | Recovered | - | Recovered | Meet | Meet | Meet |
| 86 | **OG6** | AHV | Razi | - | - | - | - | Recovered | Meet | Meet | Meet |
| 87 | **RU0** | AHV | Razi | - | Recovered | - | Not Recovered | Recovered | Not Meet | Meet | Meet |
| 88 | **CH8** | AHV | Razi | Recovered | Recovered | - | Recovered | Recovered | Meet | Meet | Meet |
| 89 | **VL2** | AHV | SnaFab | - | - | - | - | Recovered | Meet | Meet | Meet |
| 90 | **DC8** | AHV | Razi | - | Recovered | - | Recovered | Recovered | Meet | Meet | Meet |
| 91 | HV6 | AHV | Razi | - | - | - | Recovered | Recovered | Meet | Meet | Meet |
| 92 | FL2 | AHV | Razi | - | - | Recovered | - | Recovered | Meet | Meet | Meet |
| 93 | RS1 | AHV | SnaFab | - | - | - | - | Recovered | Meet | Meet | Meet |
| 94 | AJ6 | AHV | Razi | - | - | - | - | Recovered | Meet | Meet | Meet |
| 95 | ZY8 | AHV | Razi | - | - | - | Not Recovered | Recovered | Not Meet | Meet | Meet |
| 96 | YB6 | AHV | SnaFab | - | - | - | - | Recovered | Meet | Meet | Meet |
| 97 | GS2 | AHV | Razi | - | - | - | - | Recovered | Meet | Meet | Meet |
| 98 | WZ7 | AHV | Razi | - | - | - | Not Recovered | Recovered | Not Meet | Meet | Meet |

## Table 32 - Assessment of victims’ recovery over 48 hours (local and systemic symptoms) at the Mashhad center

| ID | Code | Center | Group | PT | PTT | PLT | INR | Local | Result  (Cuttoff-0) | Result (Cuttoff-1) | Result  (Cuttoff-2) |
| --- | --- | --- | --- | --- | --- | --- | --- | --- | --- | --- | --- |
| 11 | **ALSE** | MAS | SnaFab | Recovered | - | Recovered | - | Recovered | Meet | Meet | Meet |
| 16 | **EEZ9** | MAS | SnaFab | - | - | - | - | Recovered | Meet | Meet | Meet |
| 17 | **DDH8** | MAS | Razi | - | - | - | - | Not Recovered | Not Meet | Meet | Meet |
| 18 | **QQG5** | MAS | Razi | - | - | - | - | Recovered | Meet | Meet | Meet |
| 19 | **XXA1** | MAS | SnaFab | - | - | - | - | Recovered | Meet | Meet | Meet |
| 20 | **SSV7** | MAS | Razi | - | - | - | - | Recovered | Meet | Meet | Meet |
| 21 | **BBM6** | MAS | Razi | - | - | - | Recovered | Recovered | Meet | Meet | Meet |
| 22 | **YYY4** | MAS | SnaFab | Recovered | - | - | - | Recovered | Meet | Meet | Meet |
| 24 | **SSU8** | MAS | SnaFab | - | - |  | - | Recovered | Meet | Meet | Meet |
| 37 | **AAT1** | MAS | SnaFab | - |  | Not Recovered | - | Recovered | Not Meet | Meet | Meet |
| 56 | **BBP7** | MAS | SnaFab | - | - | - | - | Recovered | Meet | Meet | Meet |
| 59 | **FFT8** | MAS | Razi | Recovered | - | Not Recovered | Recovered | Recovered | Not Meet | Meet | Meet |
| 65 | **TTJ2** | MAS | Razi | - | - | - | - | Recovered | Meet | Meet | Meet |
| 83 | **NNK8** | MAS | SnaFab | - | - | - | - | Recovered | Meet | Meet | Meet |

## Table 33 - Assessment of victims’ recovery over 48 hours (local and systemic symptoms) at the Urmia center

| ID | Code | Center | Group | PT | PTT | PLT | INR | Local | Result  (Cuttoff-0) | Result (Cuttoff-1) | Result  (Cuttoff-2) |
| --- | --- | --- | --- | --- | --- | --- | --- | --- | --- | --- | --- |
| 12 | **BAEB** | URM | SnaFab | - | - | - | - | Recovered | Meet | Meet | Meet |
| 23 | **ZZI1** | URM | SnaFab | - | - | Not Recovered | - | Not Recovered | Not Meet | Not Meet | Meet |
| 25 | **NNZ3** | URM | Razi | - | - | Recovered | - | Not Recovered | Not Meet | Meet | Meet |
| 70 | **EEJ8** | URM | Razi | - | - | - | - | Recovered | Meet | Meet | Meet |

## Table 34- Delayed Adverse Reactions (During 7- and 14-day follow-up interval)

| ID | Code | Group | Start Day | End Day | Duration (Days) | Description | Severity | Treatment | Result |
| --- | --- | --- | --- | --- | --- | --- | --- | --- | --- |
| 1 | **YUAB** | **SnaFab** | **1399.02.03** | **1399.02.10** | **8** | **Vertigo** | **Mild** | **None** | **Recovered** |
| 6 | **HABA** | **SnaFab** | **1399.02.25** | **-** | **-** | **Malaise** | **Mild** | **None** | **Recovered** |
| 10 | **REAB** | **SnaFab** | **1399.03.13** | **1399.03.13** | **1** | **Bite site pain** | **Mild** | **None** | **Recovered** |
| 10 | **REAB** | **SnaFab** | **1399.03.13** | **1399.03.16** | **4** | **Dermatitis** | **Mild** | **None** | **Recovered** |
| 20 | **SSV7** | **Razi** | **1399.05.13** | **1399.05.16** | **4** | **Dermatitis** | **Mild** | **None** | **Un known** |
| 20 | **SSV7** | **Razi** | **1399.05.13** | **1399.05.16** | **4** | **Malaise** | **Mild** | **None** | **Recovered** |
| 27 | **IIE0** | **Razi** | **1399.06.16** | **1399.06.18** | **3** | **Infection bacterial** | **Moderate** | **Medication treatment (ceftriaxone 1g q12 hours), Debridment** | **Recovered** |
| 46 | **UUT4** | **SnaFab** | **1399.07.05** | **-** | **-** | **Vertigo** | **Mild** | **None** | **Un known** |
| 46 | **UUT4** | **SnaFab** | **1399.07.05** | **-** | **-** | **Bite site necrosis** | **Moderate** | **Debridment** | **Un known** |
| 48 | **SSH4** | **SnaFab** | **1399.07.05** | **1399.07.14** | **10** | **Headache** | **Mild** | **-** | **Recovered** |
| 52 | **KKW6** | **Razi** | **1399.07.09** | **1399.07.11** | **3** | **Vertigo** | **Mild** | **None** | **Recovered** |
| 65 | **TTJ2** | **Razi** | **1399.07.17** | **1399.07.20** | **4** | **Dermatitis** | **Mild** | **None** | **Recovered** |
| 65 | **TTJ2** | **Razi** | **1399.07.17** | **1399.07.20** | **4** | **Fever** | **Mild** | **None** | **Recovered** |
| 67 | **NN5** | **Razi** | **1399.07.19** | **1399.07.27** | **9** | **Weakness** | **Mild** | **None** | **Recovered** |
| 68 | **JX8** | **Razi** | **1399.07.19** | **1399.07.21** | **3** | **Vertigo** | **Mild** | **None** | **Recovered** |
| 68 | **JX8** | **Razi** | **1399.07.19** | **1399.07.21** | **3** | **Weakness** | **Mild** | **None** | **Recovered** |
| 70 | **EEJ8** | **Razi** | **1399.07.23** | **1399.07.27** | **5** | **Swelling** | **Mild** | **None** | **Recovered** |
| 70 | **EEJ8** | **Razi** | **1399.07.23** | **1399.07.27** | **5** | **Bite site pain** | **Mild** | **None** | **Recovered** |
| 92 | **FL2** | **Razi** | **1400.02.04** | **1400.02.09** | **6** | **Weakness** | **Mild** | **None** | **Recovered** |
| 97 | **GS2** | **Razi** | **1400.02.11** | **1400.02.13** | **3** | **Weakness** | **Mild** | **None** | **Recovered** |
| 98 | **WZ7** | **Razi** | **1400.02.11** | **-** | **-** | **Vertigo** | **Mild** | **None** | **Not Recovered** |

## Table 35- Concurrent medications used by the victims

| ID | Code | Group | Center | Concomitant Medications | Type |
| --- | --- | --- | --- | --- | --- |
| 1 | YUAB | SnaFab | AHV | - | - |
| 2 | MAGH | SnaFab | AHV | - | - |
| 3 | ALSA | SnaFab | AHV | - | - |
| 4 | GHMA | SnaFab | AHV | - | - |
| 5 | ABAR | SnaFab | AHV | - | - |
| 6 | HABA | SnaFab | AHV | Chlorpheniramine | Antihistamine |
| 7 | FASO | SnaFab | AHV | - | - |
| 8 | RABA | SnaFab | AHV | - | - |
| 9 | HAHO | SnaFab | AHV | - | - |
| 10 | REAB | SnaFab | AHV | - | - |
| 11 | ALSE | SnaFab | MAS | - | - |
| 12 | BAEB | SnaFab | URM | - | - |
| 13 | FAMA | SnaFab | AHV | - | - |
| 14 | ALHA | SnaFab | AHV | - | - |
| 15 | SASH | SnaFab | AHV | - | - |
| 16 | EEZ9 | SnaFab | MAS | Ceftriaxone | Antibiotic |
| 17 | DDH8 | Razi | MAS | Hydrocortisone  Vancomycin  Ceftriaxone | Antihistamine, Antibiotic |
| 18 | QQG5 | Razi | MAS | - | - |
| 19 | XXA1 | SnaFab | MAS | - | - |
| 20 | SSV7 | Razi | MAS | - | - |
| 21 | BBM6 | Razi | MAS | Cefazolin | Antibiotic |
| 22 | YYY4 | SnaFab | MAS | - | - |
| 23 | ZZI1 | SnaFab | URM | - | - |
| 24 | SSU8 | SnaFab | MAS | - | - |
| 25 | NNZ3 | Razi | URM | Hydrocortisone  Chlorpheniramine  Acetaminophen | Corticosteroid  Antihistamine  Analgesic |
| 26 | FFG3 | SnaFab | AHV | - | - |
| 27 | IIE0 | Razi | AHV | - | - |
| 28 | EEG0 | Razi | AHV | - | - |
| 29 | JJW4 | SnaFab | AHV | - | - |
| 30 | BBI6 | SnaFab | AHV | - | - |
| 31 | AAH7 | SnaFab | AHV | - | - |
| 32 | AAF9 | Razi | AHV | - | - |
| 33 | WWR1 | Razi | AHV | - | - |
| 34 | RRS3 | SnaFab | AHV | - | - |
| 35 | JJI8 | Razi | AHV | - | - |
| 36 | QQW3 | SnaFab | AHV | - | - |
| 37 | AAT1 | SnaFab | MAS | - | - |
| 38 | VVI7 | Razi | AHV | - | - |
| 39 | UUL5 | SnaFab | AHV | - | - |
| 40 | QQQ3 | Razi | AHV | - | - |
| 41 | UUN5 | Razi | AHV | Dexamethasone | Corticosteroid |
| 42 | XXN0 | SnaFab | AHV | - | - |
| 43 | RRA4 | Razi | AHV | - | - |
| 44 | DDN5 | SnaFab | AHV | - | - |
| 45 | OOZ0 | Razi | AHV | - | - |
| 46 | UUT4 | SnaFab | AHV | - | - |
| 47 | GGM9 | SnaFab | AHV | - | - |
| 48 | SSH4 | SnaFab | AHV | - | - |
| 49 | FFW7 | Razi | AHV | - | - |
| 50 | MMM9 | Razi | AHV | - | - |
| 51 | KKZ6 | SnaFab | AHV | - | - |
| 52 | KKW6 | Razi | AHV | - | - |
| 53 | EEP5 | Razi | AHV | - | - |
| 54 | FFR9 | SnaFab | AHV | - | - |
| 55 | UUN6 | Razi | AHV | - | - |
| 56 | BBP7 | SnaFab | MAS | - | - |
| 57 | OOE4 | Razi | AHV | - | - |
| 58 | QQA5 | SnaFab | AHV | - | - |
| 59 | FFT8 | Razi | MAS | - | - |
| 60 | JJY3 | SnaFab | AHV | - | - |
| 61 | GGN0 | Razi | AHV |  |  |
| 62 | ZZQ0 | SnaFab | AHV | Dexamethasone | Corticosteroid |
| 63 | JJK9 | Razi | AHV | - | - |
| 64 | YYE2 | SnaFab | AHV | - | - |
| 65 | TTJ2 | Razi | MAS | - | - |
| 66 | GG0 | SnaFab | AHV | - | - |
| 67 | NN5 | Razi | AHV | - | - |
| 68 | JX8 | Razi | AHV | - | - |
| 69 | QU4 | Razi | AHV | - | - |
| 70 | EEJ8 | Razi | URM | - | - |
| 71 | WT3 | Razi | AHV | - | - |
| 72 | DC7 | SnaFab | AHV | - | - |
| 73 | BK8 | Razi | AHV | - | - |
| 74 | ZK8 | Razi | AHV | - | - |
| 75 | FM4 | Razi | AHV | - | - |
| 76 | YP2 | SnaFab | AHV | - | - |
| 77 | GK9 | Razi | AHV | - | - |
| 78 | FC3 | Razi | AHV | - | - |
| 79 | UT8 | Razi | AHV | - | - |
| 80 | QS6 | Razi | AHV | - | - |
| 81 | RL9 | Razi | AHV | - | - |
| 82 | YF3 | SnaFab | AHV | - | - |
| 83 | NNK8 | SnaFab | MAS | - | - |
| 84 | KL5 | Razi | AHV | - | - |
| 85 | RU5 | SnaFab | AHV | - | - |
| 86 | OG6 | Razi | AHV | - | - |
| 87 | RU0 | Razi | AHV | - | - |
| 88 | CH8 | Razi | AHV | - | - |
| 89 | VL2 | SnaFab | AHV | - | - |
| 90 | DC8 | Razi | AHV | - | - |
| 91 | HV6 | Razi | AHV | - | - |
| 92 | FL2 | Razi | AHV | - | - |
| 93 | RS1 | SnaFab | AHV | - | - |
| 94 | AJ6 | Razi | AHV | - | - |
| 95 | ZY8 | Razi | AHV | - | - |
| 96 | YB6 | SnaFab | AHV | - | - |
| 97 | GS2 | Razi | AHV | Hydrocortisone | Corticosteroid |
| 98 | WZ7 | Razi | AHV | - | - |
