# Supplementary material for: The SnaFab versus the Razi antivenom for treatment of snakebite envenomation: A randomized, double-blind (investigator and victims), active controlled, non-inferiority clinical trial
Source: PLOS Glob Public Health. 2025 Nov 24;5(11):e0004281. doi: 10.1371/journal.pgph.0004281 (PMC12643280; doi:10.1371/journal.pgph.0004281)
Supplement: Attachment — Submitted filename: Response to Reviewers.docx [file pgph.0004281.s006.docx]

Response to Reviewers

Mahdi Jannati Yazdan Abad

Department of Clinial pharmacy, School of Pharmacy, Mashhad University of Medical Sciences, Mashhad

Jannatim901@gmail.com

2025/05/02

Muhammed O Afolabi, MD, MPH, PhD, Academic Editor

PLOS Global Public Health

Dear Dr. Afolabi,

I would like to express my gratitude to you and the reviewers for the thoughtful feedback and constructive comments on my manuscript titled “**The SnaFab™ Versus The Razi Antivenom for Treatment of Snakebite Envenomation: A Randomized, Double-Blind (investigator and victims), Active Controlled, Non-inferiority Clinical Trial**” (**Manuscript ID: PGPH-D-25-00087**). The insights provided have been invaluable in enhancing the quality of the manuscript. In response to the reviewers’ comments, I have carefully addressed each point raised and made the necessary revisions to improve the clarity and rigor of the research presented. Below, I outline the specific responses to each reviewer’s comments and describe the changes made to the manuscript accordingly.

Thank you for the opportunity to revise and resubmit my work. I look forward to your feedback. Sincerely,

Mahdi Jannati Yazdan Abad

I would like to clarify that the images and clip-art within the figure panels were created using **BioRender**, a platform that provides tools for creating scientific illustrations. The graphical abstract is fully compliant with copyright requirements, as I have obtained the necessary license for its use. The citation for the graphical abstract is as follows: Created in BioRender. Jannati, M. (2025) <https://BioRender.com/lgsud2m>. I have attached the confirmation of publication and licensing rights.

1. Sample Size and Recruitment: High exclusion rate (49/147 screened), raising concerns about selection bias. Also, small subgroup with severe envenomation (n=5), limited conclusions for critical cases. Gender imbalance (male-dominated cohort) reflects real-world exposure but reduces generalizability to females.

**Author Response to Comment 1:**

1. **Exclusion Rate (49/147 screened = 33.3%)**

We acknowledge the reviewer’s concern regarding our exclusion rate. However, we believe this rate is justified and reflects rigorous methodology rather than selection bias:

**Breakdown of exclusions:**

- **Pre-hospital antivenom administration (n=16, 47% of exclusions):** Essential exclusion to ensure accurate efficacy assessment
- **Delayed presentation >12 hours (n=7):** Critical for standardizing treatment timing and outcomes
- **Declined consent (n=15):** Unavoidable in clinical research
- **Other criteria (n=11):** Age constraints, non-snake bites, and cases not requiring antivenom per severity scale

The high exclusion rate primarily reflects the challenges of conducting controlled trials in emergency settings where patients often receive pre-hospital treatment or present with delayed timing. This is a common issue in antivenom research and does not indicate selection bias but rather adherence to strict inclusion criteria necessary for valid comparisons.

2. **Small Severe Envenomation Subgroup (n=5)**

**Epidemiological aspects:** The reviewer correctly notes our limited severe envenomation cases (n=5, 5.1%). However, this distribution aligns with established epidemiological patterns. According to Afroz et al.'s systematic review and meta-analysis, the natural distribution of snakebite severity typically shows approximately 9.9% severe cases, 28.2% moderate, and 46.7% mild cases. Our observed severe case rate (5.1%) falls within the expected range for this population.

**Statistical Considerations:** While we acknowledge that the small severe subgroup limits definitive conclusions for critical cases, our study was powered for the primary outcome of overall recovery rates. The severe cases analysis should be considered exploratory, and we recommend larger multi-center studies specifically designed to evaluate severe envenomation outcomes.

**Clinical Relevance:** Despite the small number, our severe cases data (showing no significant difference in antivenom requirements: P=0.59) provides valuable preliminary evidence for clinical decision-making while awaiting larger studies.

3. **Gender Imbalance (Male-Dominated Cohort)**

**Epidemiological Reality**: The male predominance in our cohort (79% male) reflects real-world snakebite epidemiology rather than selection bias. This pattern is consistently reported in snakebite literature due to occupational and behavioral exposure patterns.

**Generalizability Considerations:** We acknowledge this limits direct generalizability to female populations. However:

- Antivenom pharmacokinetics and efficacy are not expected to differ significantly between genders for the outcomes measured
- Our female subgroup (n=21) still provides valuable safety and efficacy data
- This distribution enhances external validity for the predominantly male-exposed populations in similar endemic regions

2. Confounding Factors: Significant age difference between groups (SnaFab™: 34.9 vs. Razi: 28.7 years; P-value not reported). Regional focus (Iran) limits extrapolation to other geographic settings with different snake species.

**Author Response to Comment 2:**

We appreciate the reviewer’s observation regarding the baseline age difference between groups.

First, while a statistically significant difference was observed (mean age: 34.9 vs. 28.7 years), the **absolute difference is modest and well below 20%**, a commonly referenced threshold for clinically meaningful imbalance in demographic covariates. Importantly, **randomisation remains the primary tool for controlling both known and unknown confounders**, and our trial employed stratified block randomisation to ensure balanced allocation across sites.

Second, as this is a non-inferiority trial, our analytical priority is to preserve the integrity of the treatment contrast under conditions that minimize bias. While multivariable adjustment is standard in observational studies or superiority trials to enhance statistical power and control residual confounding, it is not routinely required in well-randomised non-inferiority trials unless there is clear evidence of baseline imbalance with clinical impact.

Furthermore, **regulatory guidance (e.g., EMA/CPMP, FDA)** and methodological literature (Piaggio et al., JAMA 2012) recommend that multivariable models in non-inferiority settings (e.g., mixed-effects or regression-adjusted models) be used cautiously.

“In confirmatory trials, covariate adjustment may increase power, but should be pre-specified and justified. In non-inferiority trials, it is essential to ensure that such adjustment does not bias the result in favor of non-inferiority.”

3. Analysis Limitations: Per-Protocol Analysis excludes dropouts, risking overestimation of efficacy. Intention-to-treat analysis is missing. Authors should consider including intention-to-treat analysis to complement per-protocol results. They should also adjust for age imbalance using multivariate regression.

**Author Response to Comment 3:**

We thank the reviewer for this valuable observation regarding the choice of analysis set.

In non-inferiority trials, the choice between Per-Protocol (PP) and Intention-to-Treat (ITT) analyses is critical, as each offers distinct methodological implications. Consistent with the **CONSORT extension for non-inferiority and equivalence trials**, **SPIRIT guidelines**, and **regulatory guidance from the FDA and EMA**, we intentionally adopted a **Per-Protocol (PP) analysis** as our primary analytic approach. This decision reflects the more conservative stance in the context of non-inferiority designs, where demonstrating efficacy in a strictly adherent population helps avoid falsely concluding non-inferiority due to protocol violations or treatment non-compliance.

While ITT analysis is generally preferred for **superiority trials** to preserve randomisation and provide a pragmatic estimate of effect, it is **less conservative in non-inferiority trials** and may bias results toward equivalence, especially if non-adherence or dropout dilutes potential differences between interventions.

4. Short Follow-Up: 14-day monitoring may miss delayed adverse effects (e.g., chronic sequelae). Authors should provide information on the extended follow-up to to assess delayed complications (e.g., serum sickness, chronic joint issues).

**Author Response to Comment 4:**

We appreciate the reviewer’s concern regarding the 14-day follow-up period. However, our follow-up duration was specifically designed based on the established timeline for antivenom-related adverse reactions, particularly serum sickness, which represents the most clinically significant delayed complication following antivenom administration.

**Scientific Rationale for 14-Day Follow-Up:**

Serum sickness, the primary delayed adverse reaction of concern following antivenom therapy, typically manifests within 5-20 days after administration of heterologous proteins, with peak incidence occurring around 10-14 days post-treatment. This condition was first described by von Pirquet & Schick in 1905, establishing the classical timeline that remains clinically relevant today. Multiple studies have demonstrated that serum sickness symptoms, including fever, chills, urticaria, rash, arthralgia, myalgia, and lymphadenopathy, predominantly occur within this established timeframe. Our 14-day follow-up period falls well within this critical window and captures the peak incidence period for these complications.

**Supporting Literature for Follow-Up Protocols:**

Several antivenom safety studies have employed similar follow-up periods with demonstrated effectiveness: Dart et al. (2001) conducted a comprehensive safety evaluation of crotalid antivenom using structured follow-up protocols, demonstrating the effectiveness of systematic post-treatment monitoring for delayed reactions. Their study established important precedents for antivenom safety surveillance methodologies. Similarly, research by Warrell and colleagues has consistently shown that the majority of clinically significant delayed antivenom reactions occur within the first two weeks post-administration. Isbister & Brown (2012) employed systematic follow-up protocols in their randomized controlled trial of antivenom therapy, providing valuable insights into the timing and nature of delayed adverse reactions. Their methodology supports the use of structured follow-up periods for comprehensive safety assessment.

**Clinical Timeline Evidence:**

The medical literature consistently supports that clinically significant antivenom-related delayed reactions occur within established timeframes. Serum sickness typically develops 5-20 days after heterologous protein administration, with characteristic symptoms appearing in predictable patterns. Laboratory abnormalities associated with serum sickness, including elevated inflammatory markers and complement consumption, typically manifest within the first two weeks post-exposure.

Recent reviews of antivenom safety have reinforced that the critical monitoring period for delayed hypersensitivity reactions falls within the timeframe covered by our protocol. The pathophysiology of serum sickness involves immune complex formation and complement activation, processes that reach clinical significance within days to weeks of antigen exposure.

**Addressing Chronic Sequelae Concerns:**

While we acknowledge the theoretical possibility of chronic complications developing beyond 14 days, the available literature indicates that clinically significant chronic sequelae directly attributable to antivenom administration (as distinct from complications of the original envenomation) are rare and typically manifest within the acute and subacute phases. The most clinically relevant delayed complications occur within the monitoring window established by our protocol.

Long-term complications, when they occur, are more commonly related to the severity of the original envenomation rather than antivenom-specific adverse reactions. Our follow-up protocol is designed to capture the established timeframe for antivenom-related complications while maintaining practical feasibility.

5. Safety Reporting: Adverse events were self-reported or clinician-observed, potentially underestimating mild/moderate reactions. Authors should specify criteria for "recovery" (e.g. quantitative measures of swelling/coagulation). Authors provided no details on blinding success (e.g., whether treating physicians remained unaware of allocation). Authors should report measures taken to ensure blinding integrity (e.g., surveys of investigators/participants).

**Author Response to Comment 5**

1. **Adverse Events Reporting**

We acknowledge the reviewer’s concern about potential underestimation of adverse events. Our approach included both active and passive surveillance:

**Active surveillance:**

- Clinician-observed adverse events during hospitalization
- Structured 14-day phone follow-up with standardized questionnaire
- Patients were specifically educated about delayed reaction symptoms to watch for

**Passive surveillance:**

- Patient self-reporting during follow-up calls
- Emergency contact information provided for immediate concerns

**Patient education:** All patients received standardized education about potential delayed reactions including serum sickness symptoms (fever, rash, joint pain, swelling) and were instructed to contact the study team immediately if these occurred.

We acknowledge this approach may underestimate mild reactions, but it represents standard practice in antivenom studies and captures clinically significant adverse events.

2. **Recovery Criteria Specification**

We thank the reviewer for requesting clarification of our recovery criteria:

- **Quantitative measures**: Coagulation parameters: PT <20 seconds, INR <1.2, PTT <50 seconds, Platelet count >150,000/μL
- **Laboratory values**: Objective, measurable parameters

**Clinical measures**:

- **Swelling progression**: Assessed by clinical examination and circumferential measurements at standardized anatomical landmarks
- **Neurotoxicity progression**: Evaluated using standardized neurological assessment scales

**Assessment protocol**: All clinical assessments were performed by trained physicians using standardized examination protocols to minimize inter-observer variability.

3. **Blinding Integrity and Success**

**Blinding methodology**: Our study employed a comprehensive blinding strategy:

**Patient blinding**: Both antivenoms were prepared in identical infusion bags with identical appearance, ensuring complete patient blinding.

**Healthcare provider blinding**:

- Designated study nurses prepared all infusions according to randomization codes
- Treating physicians were kept unaware of treatment allocation whenever possible
- All study staff received pre-study training emphasizing blinding maintenance
- Clinical data was recorded using coded identifiers without treatment group information

**Data analysis blinding**: The data analysis team remained completely blinded to treatment allocation until final analysis completion.

6. Conflict of Interest: Funding and employment ties to Padra Serum Alborz (manufacturer of SnaFab™) introduce potential bias, despite rigorous methodology. Authors should disclose funder involvement in study design/data analysis

**Author Response to Comment 6**

We thank the reviewer for highlighting this important consideration. We acknowledge that industry funding, particularly when an author has employment ties to the manufacturer, introduces potential for bias despite rigorous methodology.

**Transparency measures implemented:**

- **Full disclosure**: We have clearly disclosed all financial relationships and employment ties
- **Independent oversight**: The lead statistician (Dr. Hamed Hosseini, Tehran University of Medical Sciences) has no financial relationship with Padra Serum Alborz and conducted all analyses independently
- **Methodological rigor**: We employed comprehensive blinding, randomization, and standardized protocols to minimize bias
- **Funder non-involvement**: Padra Serum Alborz had no role in study design, data analysis, interpretation, or manuscript preparation

**Additional safeguards**:

- Pre-specified primary and secondary endpoints
- Standardized outcome assessment protocols
- Independent clinical data collection by hospital staff
- Blinded data analysis until study completion

We believe these measures, combined with our transparent disclosure, allow readers to appropriately evaluate our findings while acknowledging the potential for bias inherent in industry-funded research.

7. Global Relevance: Authors should discuss implications of their trial findings in relationship with global relevance for WHO’s snakebite roadmap and alignment with global antivenom accessibility goals.

**Author Response to Comment 7:**

We appreciate the reviewer’s concern regarding the global relevance. The findings of our trial have significant implications that align with WHO’s “Snakebite Envenoming: A Strategy for Prevention and Control” roadmap, which aims to reduce snakebite mortality and disability by 50% before 2030 through improved access to safe, effective, and affordable antivenoms. By demonstrating non-inferiority of Snafab to Razi, our study supports WHO’s goal of strengthening regional antivenom manufacturing capacity, as having multiple validated manufacturers within Iran enhances supply security and reduces dependence on imported products. The availability of two comparable antivenoms could foster healthy market competition, potentially leading to more affordable pricing, thereby addressing WHO’s emphasis on antivenom accessibility and affordability. Furthermore, our rigorous clinical trial methodology provides evidence-based validation of antivenom quality, supporting WHO’s focus on ensuring effective and safe products reach patients. This study exemplifies WHO’s recommendation for developing context-specific solutions, as both antivenoms are specifically designed for Iranian snake species and healthcare settings. Having multiple validated manufacturers strengthens the sustainability of antivenom supply, addressing WHO’s concern about ensuring consistent availability of these essential medicines. These findings contribute to the broader goal of improving global antivenom accessibility while maintaining high standards of safety and efficacy, directly supporting WHO’s strategic objectives for reducing the burden of snakebite envenoming worldwide.

5. Review Comments to the Author

Please use the space provided to explain your answers to the questions above. You may also include additional comments for the author, including concerns about dual publication, research ethics, or publication ethics. (Please upload your review as an attachment if it exceeds 20,000 characters)

Reviewer #1: The authors present results from a non-inferiority trial of SnaFab to Razi, two antivenom treatments, among snake bite victims in Iran. Recovery within 48 hours was assessed. Adverse events (AE) are also summarized. Authors found similar rates of recovery in the two arms, falling within the non-inferiority margin. Fewer individuals in the SnaFab group experienced adverse events than in the Razi group, though this difference was not statistically significant. The manuscript will be strengthened if the authors consider the following points.

1. In the abstract, results and discussion, authors should provide a fair comparison of the two groups in relation to adverse events. Specifically, they highlight the most commonly reported AE in the Razi group, but do not provide the most common AE in the SnaFab group. There is also a paragraph in the discussion about the low incidence of AE in the SnaFab group, but the percentage of individuals experiencing an AE in the two groups was not significantly different. The Conclusion similarly states that SnaFab boasts a more favorable safety profile. That is too strong of a statement given their data.

We completely agree with the reviewer’s assessment and acknowledge that our original presentation was unbalanced and overstated the safety differences. We have made the following revisions:

1. Abstract - “Adverse events were reported by 10.2% of individuals in the SnaFab group and 20.4% in the Razi group (P = 0.27). Muscle weakness was the most commonly reported adverse event in the Razi group (8%), while vertigo was most frequent in the SnaFab group (4%). No cases of anaphylactic shock or serum sickness were observed in either group.”

2. Conclusion - Revised: “In summary, this study found that SnaFab antivenom is non-inferior to Razi antivenom for treating snakebite envenoming in Iran. Overall, the incidence of adverse events was comparable between groups, with no statistically significant differences observed.”

3. Discussion - Revised: “Fortunately, none of the patients in our study exhibited symptoms consistent with serum sickness (fever, malaise, or itchy rash) during the 14-day follow-up period, eliminating the need for additional hospital visits. Given the absence of severe adverse events and the comparable incidence of mild-to-moderate adverse effects between groups, both antivenoms demonstrate similar safety profiles in this population.”

2. In the definition of recovery (the primary outcome), authors state that at least 3 recovery criteria had to be met - the coagulation abnormalities recovery includes 4 possibilities, so would each of those count? Also, I am assuming an individual had to have abnormal coagulation in order for the coagulation components to be considered in the recovery - is that correct? If so, how would someone without coagulation abnormalities meet the recovery criteria, since there are only 2 other aspects of recovery that could be met?

**Author Response to Comment**

We thank the reviewer for identifying this important inconsistency in our recovery definition. You are absolutely correct that our original definition created a logical problem.

**Clarification of our approach:**

**Coagulation parameters:** Each of the 4 coagulation parameters (PT, INR, PTT, PLT) counted as separate recovery criteria, giving us 6 total criteria (not 3 as originally stated).

**Baseline requirement:** Patients were only assessed for coagulation recovery criteria if they had abnormal values at baseline. For example, if a patient had normal PT and INR at presentation but abnormal PTT and platelets, they could meet up to 4 total criteria (swelling + neurotoxicity + PTT normalization + platelet recovery).

**Recovery threshold:** Patients needed to meet at least 3 of their applicable criteria to be considered recovered. For patients with fewer than 3 applicable criteria (e.g., those with normal baseline coagulation), recovery required meeting all available criteria.

We have revised our methods section to clearly reflect this 6-criteria approach and specify the conditional requirements based on baseline presentation. This ensures all patients had a fair opportunity to meet the recovery definition regardless of their presenting coagulation status."

3. In the sample size section, authors should include the base proportion, as they do in the protocol. As stated in the manuscript, there is insufficient information to replicate the power calculation.

**Author Response to Comment**

We sincerely thank the reviewer for highlighting the lack of sufficient detail in our original sample size section. We agree that full transparency is important to enable replication of the power calculation.

In response, we have now revised the **Sample Size Determination** section of the manuscript to include the following key assumptions used in the calculation:

- Reference (control) group recovery rate: **90%**
- Assumed treatment group rate under null hypothesis (inferiority): **70%**
- Non-inferiority margin: **20%**
- Power: **81%**
- One-sided alpha: **0.025**
- Test: **One-sided Score test (Farrington & Manning)**

This yielded a required sample size of **42 participants per group**, which was increased to **98** after accounting for a 15% potential dropout rate. These details have been added to the manuscript for clarity and reproducibility

4. The non-inferiority margin is 20% which seems rather large when comparing treatments to something as potentially dangerous as snakebites. Was there any rationale behind this choice of the NIM?

**Author Response to Comment 4:**

We thank the reviewer for their critical insight regarding the choice of the 20% non-inferiority margin (NIM), which indeed reflects an important methodological consideration, especially in trials involving potentially life-threatening conditions such as snakebite envenoming.

We acknowledge that a **10% margin is generally preferred** in many regulatory settings, and it is often regarded as the more conservative threshold for non-inferiority conclusions. However, the **20% margin adopted in our study represents the upper acceptable limit** and was selected based on several **practical and ethical considerations**:

- At the time of protocol development, there was a **scarcity of robust local data** regarding treatment efficacy and event rates in Iranian snakebite victims.
- The study faced **resource constraints**, both in terms of production and enrolment capacity, given the public health urgency and limited number of eligible cases.
- The SnaFab antivenom was a **new locally manufactured product**, and the trial was intended as part of a **phased clinical development** under **close supervision** of national regulatory authorities and the Data Safety and Monitoring Board (DSMB).

Importantly, the **Iranian FDA and DSMB** reviewed and **approved the use of a 20% NIM**, conditional upon:

1. **Regular interim safety and efficacy reporting**, and
2. **Independent oversight of trial conduct and protocol adherence**.

Interim analyses shared with the regulatory bodies confirmed that **the observed recovery rates were consistently high and balanced between groups**, with no safety signals or efficacy concerns that warranted early termination or recalculation of the sample size. This reinforced confidence in the continued validity of the original assumptions and NIM throughout the study duration.

5. Authors list reasons for exclusion in the 1st paragraph in the results, but they should provide how many were excluded for each reason (also the exclusion related to age would be outside the age range, not 2-60 years).

**Author Response to Comment 5:**

Thank you for this important clarification. We have revised our exclusion criteria description for accuracy and completeness:

A total of 34 participants were deemed ineligible for the study based on the following exclusion criteria:

- Age Constraints (n=5):

Participants outside the specified age range of 20-60 years

- Inappropriate Complaint Type (n=4):

Individuals with non-snake bite related complaints

- Pre-hospital Antivenom Administration (n=16):

Participants who received antivenom prior to hospital admission

- Delayed Presentation (n=7):

Patients presenting more than 12 hours after snake bite incident

- Severity Assessment (n=2):

Individuals determined not to require antivenom based on bite severity scale

- Additionally, 15 potential participants declined to provide informed consent for study participation.

6. Authors need to refer to Figure 2 in the text. The title for Figure 2 should have "compare to" changed to "compared to". Also, I believe authors have flipped the Favors SnaFab and Favors Razi in the figure. Authors are reporting SnaFab-Razi, so the right of no difference would be SnaFab having higher recovery than Razi.

**Author Response to Comment 6:**

Thank you for these important corrections. We have addressed all three issues

7. Authors are encouraged to carefully read through the manuscript. There are missing words (such as "Based on recent study..." on page 2 instead of "Based on a recent study"), extra capitalization ("and Irreversible complications" also on page 2), and other grammatical or typographical errors. Another example is "relevant snake's native to geographic regions of Iran" on page 3..."snake's" should be "snakes".

**Author Response to Comment 7:**

Thank you for these important corrections. We have addressed all issues.

Minor points:

1. the word "data" is plural, so authors should use "data show" or "data ...show" (first paragraph in Background section). Similarly, "These data are" should be used instead of "This data is" (page 10, 2nd paragraph)

Thank you for these important corrections. We have addressed all issues.

2. In the 1st paragraph of the Background, authors refer to an "uncertainty range" - is this a 95% confidence interval?
Thank you for the insight. Yes, it was referred to 95% CI and it is revised in the main text

3. on page 3, 2nd line, authors refer to frequency of snakebites in a sentence about early antivenom administration. I'm not sure how the frequency of snakebites is related to the timing of administration. I think the authors are combining two different ideas here and should clarify the sentence.

Thank you for the consideration. We have addressed the issue:

Early antivenom administration is crucial for optimal patient outcomes, as it significantly impacts recovery rates and reduces complications. Given the severity of snakebite envenomation and the critical importance of timely treatment, the efficacy of antivenom is paramount.

4. page 3, in the Rationale of design section, the sentence "Immunoglobins prepared..." is an incomplete sentence and should be revised.

We have addressed the issue:

Immunoglobulins prepared by caprylic acid fractionation undergo proteolytic digestion to produce fragmented antibodies. Compared to prior antivenom purification methods like ammonium sulfate precipitation, which was time-consuming and less efficient due to protein aggregation (13), caprylic acid refinement is more practical, cost-effective, and causes fewer adverse reactions in humans (14, 15).

5. Table 1: an asterisk is used after shock in the description of Severe for viper bites, but the asterisk is not defined.

We have addressed the issue:

Cardiovascular collapse (cardiac arrest, overt shock [SBP<80 mmHg and decreased peripheral perfusion]), Rhabdomyolysis, Severe active bleeding

6. Since the protocol states that intent-to-treat analysis will also be performed, authors should state somewhere that the analyses conducted were both per protocol and intent-to-treat (since everyone who was randomized completed the study without deviations).

Thank you for this important clarification. You are absolutely correct that we should explicitly state our analysis approach. We have added the following statement to our Methods section (Statistical Analysis subsection): “Both intent-to-treat (ITT) and per-protocol (PP) analyses were planned and conducted. Since all randomized participants (n=98) completed the study without major protocol deviations, the ITT and PP populations were identical, and the results presented represent both analysis approaches.” Additionally, we have added clarification to our Results section: “All 98 randomized participants completed the study protocol without major deviations. Therefore, the intent-to-treat and per-protocol analysis populations were identical, strengthening the robustness of our findings.”

7. The enrollment dates are given in the Methods section of the Abstract, but the results section of the main manuscript. For consistency, they should be reported in the same section of the Abstract and main text.

We have addressed the issue.

8. Figure 1 - what is the "allocated to the safety phase" box for? Authors state that all participants were evaluated for safety throughout the follow-up, so I'm not sure what is different about those 15. This should be clarified.

Our study employed a **sequential design** due to practical constraints during the COVID-19 pandemic:

**Phase 1 - Safety Run-in (n=15):**

- Initially conducted as a preliminary safety assessment
- All 15 participants received SnaFab antivenom
- Evaluated for immediate safety concerns before proceeding to full trial

**Phase 2 - Efficacy and Non-Inferiority Trial (n=98):**

- The 15 participants from the safety phase were **included** in the SnaFab arm of the main trial
- Additional 83 participants were recruited and randomized
- **Final allocation**: SnaFab (n=49, including the 15 from safety phase) vs. Razi (n=49)

**Rationale for Sequential Design:**

- **COVID-19 Impact**: Patient enrollment was challenging due to pandemic restrictions
- **Shortened Hospital Stays**: Per hospital directive 138/04/99, many patients had shorter hospitalizations (<72 hours vs. protocol-specified duration) due to COVID-19 transmission concerns
- **Regulatory Prudence**: Initial safety assessment was deemed appropriate before full-scale comparison

**Clarification for Figure 1:**

The “Allocated to Safety Phase (n=15)” box represents the initial safety run-in participants who were subsequently included in the main trial’s SnaFab arm. All participants (n=98) were evaluated for both safety and efficacy outcomes using identical protocols.

9. Table 2: in the title, change "compare to" to "compared to". Also, it is not clear to me in the Local (and possibly systemic) section, what n (%, total) represents...in the Local section, the total is more than the number of participants for some local symptoms. Authors should clarify what is being reported.

The following issue has been addressed:

Revised: “Baseline characteristics and clinical manifestations compared between SnaFab and Razi antivenom groups”

n = number of patients developing specific complications; % = percentage of patients in each group; total = cumulative number of all local or systemic reactions (patients may have multiple symptoms simultaneously)

10. page 7, %98 and %100 should be changed to 98% and 100%. Also, authors should remove "and not exceeding the NIM" and talk about that when referring to Figure 2.

The following issue has been addressed.

11. Some of the information in the Secondary Endpoints section of the results was already reported in the prior paragraph (# with moderate symptoms in each group and # with severe symptoms in each group). In the prior paragraph, authors refer to Table 2 after a sentence on the number in each group with moderate symptoms, but Table 2 does not easily identify moderate symptoms, so referring to that table does not make sense.

We addressed the following issue:

All patients in both SnaFab and groups presented with local symptoms including pain and swelling. Upon admission, 35 patients (71.4%) in the Razi group and 26 patients (53.1%) in the SnaFab group exhibited systemic manifestations in addition to local symptoms, including coagulation disorders, thrombocytopenia, and general weakness (Table 2). Severe symptoms, such as active severe hemorrhage and compartment syndrome, were observed in 3 patients (6.12%) from the SnaFab group and 2 patients (4.08%) from the Razi group. Additionally, according to the snakebite severity scale (Table 1), moderate symptoms were documented in 46 patients (93.8%) from the Padra Serum Alborz group and 47 patients (95.9%) from the Razi Serum group (Table 2).

*Secondary Endpoints*: According to the study, the average amount of antivenom administered to victims in the SnaFab group (8.95 ± 4.40 units) and the Razi group (9.04 ± 4.49 units) did not show a significant difference (P value: 0.92). Of the 93 patients presenting with moderate envenomation symptoms according to the snakebite severity scale, 46 patients were treated in the SnaFab group and 47 patients in the Razi group. The total antivenom consumption was 394 vials in the SnaFab group and 402 ampoules in the Razi group (P value: 0.71). Among the study population, severe envenomation was documented in 5 cases at presentation. The SnaFab cohort included three such cases (patients #1, #4, and #44) requiring 20, 15, and 10 vials respectively, while the Razi cohort included two cases (patients #50 and #63) requiring 10 and 15 vials respectively. The difference in antivenom requirements between groups was not statistically significant (P=0.59).
