# Supplementary material for: The SnaFab versus the Razi antivenom for treatment of snakebite envenomation: A randomized, double-blind (investigator and victims), active controlled, non-inferiority clinical trial
Source: PLOS Glob Public Health. 2025 Nov 24;5(11):e0004281. doi: 10.1371/journal.pgph.0004281 (PMC12643280; doi:10.1371/journal.pgph.0004281)
Supplement: Attachment — Submitted filename: Response_to_Reviewers_auresp_2.docx [file pgph.0004281.s007.docx]

**Response to Editorial Comment on References**

We thank the Editor for carefully reviewing our reference list and for pointing out the need to ensure accuracy and completeness. We have carefully reviewed and revised the references as follows:

1. Corrections: Three references contained corrections. The journal correction notices are as follows:

**Warrell DA. Snake bite. The lancet. 2010;375(9708):77-88.**: In this Seminar (Jan 2, 2010), the y-axis on figure 8 should have read: “Venom antigen (μg/L)”.

**Warrell D, Davidson NM, Omerod L, Pope HM, Watkins BJ, Greenwood B, et al. Bites by the saw-scaled or carpet viper (Echis carinatus): trial of two specific antivenoms. Br Med J. 1974;4(5942):437-40.**: We regret that the name of Dr. L. D. Ormerod was misspelt in the paper under this title (23 November, p. 437).

## **Afroz A, Siddiquea BN, Chowdhury HA, Jackson TNW, Watt AD. Snakebite envenoming: A systematic review and meta-analysis of global morbidity and mortality. PLOS Neglected Tropical Diseases. 2024;18(4):e0012080**.: Early Version *PLOS Neglected Tropical Diseases* will publish an uncorrected page proof of your manuscript as an early version of your manuscript in advance of the final article soon after the time that you receive your author proof. The date the early version is posted will be your article’s publication date. The final article, which will include any changes made during the proof stage, will be published to the same URL and have the same DOI. The early version will remain accessible to readers. If you do not want to have this early version published, you will have a chance to opt out.

2. Duplicates: Three duplicate references were identified in the preliminary submission. These have been removed, and the numbering has been adjusted accordingly.

**Warrell DA. Snake bite. The lancet. 2010;375(9708):77-88.**

**de Silva HA, Ryan NM, de Silva HJ. Adverse reactions to snake antivenom, and their prevention and treatment. British journal of clinical pharmacology. 2016;81(3):446-52.**

**Habib AG, Brown NI. The snakebite problem and antivenom crisis from a health-economic perspective. Toxicon. 2018;150:115-23.**

3. Formatting: The entire reference list has been revised and reorganized in accordance with the Vancouver style requirements.

We confirm that the revised reference list contains no retracted articles. We greatly appreciate the Editor’s guidance, which helped us ensure accuracy and compliance in our reference section.

**Response to Reviewer Comment**

Reviewer comment:

- In Figure 1, authors show that participants were randomized and then there were the 15 SnaFab assessed for safety, which does not seem to match the sequential design described in their response. The sequential design would suggest those first 15 were not actually randomized, but instead were just given SnaFab. Authors need to describe their design within the manuscript and ensure that Figure 1 matches that design.

Author response:

We sincerely thank the reviewer for the opportunity to clarify this important point. We recognize that our **earlier explanation and diagram** in the previous revision inadvertently created ambiguity about the nature of the “Allocated to Safety Phase (n=15)” group. In our earlier reply, our wording unintentionally suggested a *sequential, non-randomized* start to the trial. This was **incorrectly phrased** and does not reflect the actual conduct of the study. We take full responsibility for that lack of clarity.

**Actual Trial Conduct (as per protocol, ethics approval, and trial registration):**

- **All 98 participants** in the trial were randomized **at enrollment** 1:1 to either SnaFab or Razi antivenom using a computer-generated allocation sequence (Sealed Envelope) managed by an independent contract research organization (CRO), with full allocation concealment.
- Per **Iranian FDA regulatory requirement**, the *first 15 participants randomized to the SnaFab arm* were designated as a regulatory-mandated **safety run-in subgroup**.
- These participants received the same intervention, follow-up, and monitoring as all other SnaFab patients, but their safety data were reviewed by the Data Safety Monitoring Board (DSMB) before recruitment of the remaining participants was allowed to proceed.
- Once DSMB safety confirmation was obtained, recruitment for the remaining participants continued under the same randomized, double-blind, parallel-arm non-inferiority design.
- All 15 safety-phase participants were **pre-specified** to be included in the main analysis of both efficacy and safety outcomes.

**What “Allocated to Safety Phase” Means in Figure 1:**
This box simply identifies the first 15 participants randomized into the SnaFab arm who were observed under regulatory safety monitoring before continuing full recruitment. It does not represent a separate, non-randomized preliminary cohort. All participants — both safety-run-in and the remainder — were evaluated for both safety and efficacy outcomes using identical criteria and procedures.

**Rationale for the Safety Run‑in:**

- **Regulatory prudence:** IFDA mandated an initial safety assessment before large‑scale comparison in humans.
- **Scientific & methodological:** Seamless phase 2/3 and roll‑over designs are routinely accepted in high‑risk or low‑incidence conditions where early safety verification is necessary, but all participants follow the same protocol.
- **Ethical/pandemic context:** During COVID‑19, excluding or re‑randomizing treated participants would have been unethical and would have undermined statistical power.
- **Baseline comparability:** Safety‑phase and main‑phase participants met identical eligibility criteria and had comparable baseline demographics (now shown in Results).
- **FDA guidance alignment:** U.S. FDA’s March 18, 2020 guidance on clinical trials during COVID‑19 supported adaptive measures that preserved participant safety, GCP compliance, and study integrity.

**Addressing the Confusion:**
The initial CONSORT flow diagram we provided in a prior revision was a **preliminary draft** that did not clearly show that the safety run‑in occurred *within* the randomized SnaFab arm. The revised diagram now in the manuscript is drawn directly from the final trial database and matches both the ethics‑approved protocol and regulatory documentation.

For transparency, we have:

Clarified the safety run‑in process in the **Methods** section.

Explained potential interpretive implications in the **Limitations** section.

Offered the IFDA approval letter and DSMB documentation to the Editor for independent verification.

We thank the reviewer for prompting us to provide this clarification — we believe this updated description will prevent similar misinterpretation for readers.

For further clarity, we are providing to the Editor (confidentially) two official letters from the Iranian Food and Drug Administration (IFDA):

- **Letter 1 (March 14, 2020)** – Initial clinical trial authorization specifying that the first 15 participants in the SnaFab arm would constitute a regulatory‑mandated safety run‑in phase, with DSMB oversight, before proceeding to the main trial phase.
- **Letter 2 (August 9, 2020)** – Authorization to proceed to the full non‑inferiority phase of the trial after IFDA’s review and acceptance of the DSMB safety report from these first 15 participants.

These letters confirm that the “Allocated to Safety Phase” group in Figure 1 was not a design change, but a **pre‑approved, regulator‑mandated component embedded within the randomized trial**. This requirement was documented before trial initiation and strictly followed without modification. The full Persian originals and certified translations are available to the Editor for verification.

In a few places, authors have the "%" sign before the number (for example, twice in the Abstract (%8 and %4) and %60 and %95 in the Discussion) - authors should ensure when they report percentages, the "%" sign follows the number.

We sincerely thank the reviewer for precise observations. All the signs have been reconsidered and ordered accordingly

Table 2 has the incorrect percentage for the SnaFab group for male (41/49=83.67% as stated in the text, not 86.7% in the table. Also, Table 2 has a superscript "1" above each of the sample sizes, but I'm not sure what that refers to (there is no note corresponding to a superscript 1). Also, the font for front right limb is different from the rest of the table.

We sincerely thank the reviewer for precise observations. Table 2 has been revised and all the percentages recalculated.
